# Evolutionary Histories of Type III Polyketide Synthases in Fungi

**DOI:** 10.3389/fmicb.2019.03018

**Published:** 2020-01-21

**Authors:** Jorge Carlos Navarro-Muñoz, Jérôme Collemare

**Affiliations:** Westerdijk Fungal Biodiversity Institute, Utrecht, Netherlands

**Keywords:** gene duplication, gene loss, horizontal gene transfer, secondary metabolite, gene cluster, resorcinol, pyrone, comparative genomics

## Abstract

Type III polyketide synthases (PKSs) produce secondary metabolites with diverse biological activities, including antimicrobials. While they have been extensively studied in plants and bacteria, only a handful of type III PKSs from fungi has been characterized in the last 15 years. The exploitation of fungal type III PKSs to produce novel bioactive compounds requires understanding the diversity of these enzymes, as well as of their biosynthetic pathways. Here, phylogenetic and reconciliation analyses of 522 type III PKSs from 1,193 fungal genomes revealed complex evolutionary histories with massive gene duplications and losses, explaining their discontinuous distribution in the fungal tree of life. In addition, horizontal gene transfer events from bacteria to fungi and, to a lower extent, between fungi, could be inferred. Ancestral gene duplication events have resulted in the divergence of eight phylogenetic clades. Especially, two clades show ancestral linkage and functional co-evolution between a type III PKS and a reducing PKS genes. Investigation of the occurrence of protein domains in fungal type III PKS predicted gene clusters highlighted the diversity of biosynthetic pathways, likely reflecting a large chemical landscape. Type III PKS genes are most often located next to genes encoding cytochrome P450s, MFS transporters and transcription factors, defining ancestral core gene clusters. This analysis also allowed predicting gene clusters for the characterized fungal type III PKSs and provides working hypotheses for the elucidation of the full biosynthetic pathways. Altogether, our analyses provide the fundamental knowledge to motivate further characterization and exploitation of fungal type III PKS biosynthetic pathways.

## Introduction

The genomic era has revealed that fungal genomes carry many more biosynthetic pathways than known compounds, demonstrating that the fungal kingdom has been an underexploited resource of secondary metabolites (SMs). Access to this hidden biodiversity is hampered by the strict regulation of SM biosynthetic pathways under specific conditions that are difficult to reproduce in the laboratory (Keller, 2019). Assessing the diversity of biosynthetic pathways encoded in fungal genomes is therefore the first step to prioritize the activation of candidate gene clusters toward exploiting fungal natural products and engineering novel compounds.

Fungi possess a high potential to produce SMs of the polyketide family (Collemare et al., 2008), some of them are well characterized because of their biological activities or toxicity (Keller, 2019). Fungal polyketides are synthesized through different routes, each involving a specific class of polyketide synthase (PKS). Type I iterative PKSs are multidomain mega-enzymes that are responsible for the production of most fungal polyketide compounds (Herbst et al., 2018). A second route relies on type III PKSs, which are enzymes consisting of a single keto-synthase (KS) domain (Yu et al., 2012). While type I PKSs have been well characterized and found to be abundant in fungal genomes, only a handful of fungal type III PKSs have been characterized so far (Hashimoto et al., 2014; Sun et al., 2016; Ramakrishnan et al., 2018; Yan et al., 2018; Kaneko et al., 2019; Manoharan et al., 2019).

Type III PKSs were initially found in the 1970s in plants, with the best representative enzyme being chalcone synthases, which catalyze the precursors of plant flavonoids, isoflavonoids and anthocyanins (Ferrer et al., 1999; Austin and Noel, 2003). Until the 1990s, their occurrence was thought to be restricted to plants, but type III PKSs were then identified and characterized in bacteria. The first bacterial representative is RppA from *Streptomyces griseus*, which catalyzes 1,3,6,8-tetrahydroxynaphthalene, a precursor of hexahydroxyperylenequinone melanin (Funa et al., 1999). Genome analysis of the fungus *Aspergillus oryzae* at the beginning of the 21st century revealed that this fungus contains four type III PKS genes (*CsyA*, *CsyB*, *CsyC*, and *CsyD*), and that other fungi also possess type III PKS genes (Seshime et al., 2005). Since then, type III PKS genes are regularly reported in fungal genomes (for examples, see Lackner et al., 2012; Bertrand et al., 2018; Sayari et al., 2018). Phylogenetic analyses of type III PKSs from plants, bacteria and fungi consistently revealed a unique origin for the fungal clade (Seshime et al., 2005; Goyal et al., 2008 Hashimoto et al., 2014; Shimizu et al., 2017; Yan et al., 2018). Although three distinct phylogenetic clades have been reported (Shimizu et al., 2017), a detailed phylogenetic analysis of fungal type III PKSs is lacking.

Polyketides produced by type III PKSs are typically grouped into α-pyrones (or 2-pyrones), resorcylic acids/resorcinols, and chalcones, according to the cyclization type (Shimizu et al., 2017). These molecules exhibit diverse biological activities and functions, including antimicrobial activities, raising interest in understanding their biosynthesis (Austin and Noel, 2003; Lim et al., 2016; Sun et al., 2016). In addition, α-pyrones are building blocks for many SMs and are thus of great interest in synthetic chemistry to produce new compounds with diverse biological activities (Lee, 2015). Type III PKSs catalyze the iterative condensation of a starter fatty acyl-CoA and of several extender units, mostly malonyl-CoA, as well as intramolecular lactone, aldol or Claisen cyclization (Lim et al., 2016; Shimizu et al., 2017). Although they can accept a wide range of fatty acyl-CoA starter units, from short to long linear (e.g., acetyl-CoA, steraoyl-CoA), branched (e.g., isobutyryl-CoA) or cyclic (e.g., *p*-coumaryl-CoA, benzoyl-CoA) molecules (Shimizu et al., 2017), they always show higher affinity for specific substrates (Funa et al., 2007; Rubin-Pitel et al., 2008; Li et al., 2011; Jeya et al., 2012; Ramakrishnan et al., 2018; Manoharan et al., 2019). The different affinity for diverse starter units is explained by changes in the structure of type III PKSs (Goyal et al., 2008; Rubin-Pitel et al., 2008; Seshime et al., 2010b; Mori et al., 2015). The diversity of substrates and enzymatic reactions result in a high diversity of compounds produced by type III PKSs. A recent review suggested a functional classification of type III PKSs to take into account the starter unit, number of elongation units and cyclization type (Shimizu et al., 2017).

Only eleven type III PKSs have been characterized in the fungal kingdom, ten from Ascomycota and one from Basidiomycota. The first fungal type III PKS to be functionally characterized is the pentaketide resorcylic acid synthase ORAS from *Neurospora crassa*. *In vitro* characterization of a recombinant ORAS protein showed that this enzyme can accommodate C4 to C20 fatty acyl-CoA starter units, but it exhibits a clear preference for longer chains to produce tetra and pentaketide resorcylic acids (Funa et al., 2007). Similar results were obtained with recombinant *Aspergillus niger* AnPKS and An-CsyA (Li et al., 2011; Kirimura et al., 2016), *Botrytis cinerea* BPKS (Jeya et al., 2012), *Sporotrichum laxum* Sl-PKS2 (Sun et al., 2016), *Sordaria macrospora* SmPKS and *Chaetomium thermophilum* CtPKS (Ramakrishnan et al., 2018), and *Fusarium incarnatum* FiPKS (Manoharan et al., 2019). However, fungal type III PKSs expressed in a fungal host yield compounds that are different from the recombinant proteins. For example, overexpression of the *A. niger* type III PKS yields four products only, of which the major one is protocatechuic acid (Lv et al., 2014). The type III PKS SsArs from *Sharaia* sp. Slf14 produces six alkylresorcinols from long starter units when heterologously expressed in *Saccharomyces cerevisiae* (Yan et al., 2018). Similarly, CsyA produces in *A. oryzae* three related compounds, with the major product being the pentaketide 3,5-dihydroxybenzoic acid (Seshime et al., 2010b), while recombinant CsyA yields tri and tetraketide pyrones from C4 to C18 starter units, with a preference for C6 and C7 fatty acyl-CoAs (Yu et al., 2010). In *A. oryzae*, CsyB produces csypyrones from acetoacetyl-CoA starter unit and a ketoacyl diketide unit (Seshime et al., 2010a; Hashimoto et al., 2013; Mori et al., 2015). Recently, the type III PKS PspB from *Penicillium soppi* was shown to accept as starter unit an unsaturated linear polyketide produced by a reducing type I PKS, PspA, yielding the alkylresorcinol soppiline B (Kaneko et al., 2019). Type III PKSs can also accommodate long fatty acids as shown with SsArs which can use unsaturated fatty acids from soybean oil to produce 5-(8′Z,11′Z-heptadecadienyl)resorcinol (Yan et al., 2018). These findings extend the diversity of starter units fungal type III PKSs can accommodate. In addition to these characterized enzymes, several type III PKS genes have been reported in fungal genomes (Muggia and Grube, 2010; Lackner et al., 2012; Bertrand et al., 2018; Sayari et al., 2018). In other fungi, newly identified SMs are predicted to be synthesized by a type III PKS (Rusman et al., 2018), suggesting that these fungi also contain type III PKS genes.

Despite interesting and diverse biological activities, polyketides produced by type III PKSs have been neglected in fungi. In order to fully exploit these compounds, it is timely to obtain a comprehensive overview of their occurrence and diversity in fungal genomes. In the present study, we report the first evolutionary analysis of fungal type III PKSs at the whole kingdom level. Phylogenetic analyses identified distinct evolutionary histories that have likely resulted in biosynthetic pathway diversification. Analysis of the type III PKS gene loci identified different putative gene clusters that likely contribute to the diversity of compounds produced by these pathways. Our results establish a reliable foundation for directing the future identification of novel polyketides with interesting biological activities.

## Materials and Methods

### Retrieval of PKS Sequences and Gene Clusters in Fungal Genomes

A total of 1,193 genomes (on 2019-04-17; genomes from *Saccharomycotina* spp. were omitted because an initial search did not retrieve any type III PKS gene; Supplementary Table S1) were retrieved from the Joint Genome Institute (JGI) Mycocosm repository (Grigoriev et al., 2014), and were analyzed with antiSMASH 4 (parameters: –minimal) (Blin et al., 2017), which reported 38,525 regions potentially containing biosynthetic gene clusters (BGCs). A total of 557 sequences were identified as type III PKSs and were analyzed for conserved domains with HMMER v3.2.1^1^. The sequences that contain both the chalcone and stilbene synthase N- and C- terminal domains (PF00195 and PF02797, from version 32 of the Pfam database (El-Gebali et al., 2019) were selected from the antiSMASH results. Both domains are specific of type III PKSs. We did not include sequences that contain only one of these conserved domain because these sequences likely correspond to pseudogenes or wrongly predicted genes. Additionally, a query in JGI Mycocosm with both Pfam terms reported three sequences that were below the default cut-off of antiSMASH, yielding a total of 522 type III PKS sequences (Supplementary Material S1). The automated gene structure prediction of 19 sequences were manually curated (Supplementary Material S1). Forty characterized sequences of fungal, bacterial and plant origins were added as reference from the Minimum Information about a Biosynthetic Gene Cluster (MIBiG) database (Medema et al., 2015) and from literature for a total of 74 sequences, including 10 characterized fungal type III PKS (Supplementary Material S2).

All BGCs predicted by antiSMASH to contain a type III PKS of fungal origin were searched for reducing PKS sequences as characterized by the presence of either PF08659 (KR keto-reductase) or PF14765 (PS-DH dehydratase) domains; and the absence of any signature domain from non-reducing PKSs (either PF16073, SAT starter unit:ACP transacylase or TIGR04532, PT product template). This search produced a list of 46 sequences, which were complemented with all reducing PKS sequences from the MIBiG database, as well as the characterized PspA PKS (Kaneko et al., 2019), for a total of 110 sequences (Supplementary Material S3).

For fungal sequences that were found to be closely related to bacterial type III PKSs, a blastp search was performed on the NCBI server using the -nr database and the best hit sequence was retrieved (Supplementary Material S4).

### Phylogenetic Tree Construction

Amino acid sequences were aligned using Clustal Omega v1.2.4 (parameters –hmm –in using PF00195.19 as a guide for type III PKSs and PF00109.25 as a guide for reducing PKSs; no model was used to build the tree to assign bacteria-to-fungi HGT events) (Sievers and Higgins, 2018). Poorly aligned regions were removed using Trimal v1.4.rev15 (build[2013-12-17]; parameters: -automated1) (Capella-Gutiérrez et al., 2009). Maximum-likelikhood phylogenetic trees were built using IQ-TREE v1.6.11-he860b03_0 bioconda (Nguyen et al., 2015) with model finder (Kalyaanamoorthy et al., 2017) and ultrafast bootstrap (parameters: -bb 1000 -alrt 1000 -nt AUTO) (Hoang et al., 2018). Phylogenetic trees were displayed using iTOL (Letunic and Bork, 2019). All curated alignments and phylogenetic tree files are provided in Supplementary Material S5. Sequence logos were generated using WebLogo 3 (Crooks et al., 2004).

### Species Tree Construction

The core eukaryotic genes KOG1753 (Ribosomal Protein S9 RPS9), KOG0189 (phosphoadenylphosphosulfate reductase PAP) and KOG2314 (translation initiation factor 3, subunit b eIF-3b) were retrieved from the JGI Mycocosm repository for all genomes that contain a type III PKS. Nucleotide sequences were aligned using MAFFT (Katoh and Standley, 2013) and phylogenetic trees were built using FastTree (Price et al., 2010). These preliminary trees were used to identify paralogs and exclude them from the species tree construction. Orthologous sequences of *RPS9*, *PAP* and *eIF-3b* genes were independently aligned with MAFFT, poorly aligned regions were removed using Trimal v1.4.rev15 (build[2013-12-17]; parameters: -automated1) (Capella-Gutiérrez et al., 2009), and the phylogenetic tree was built with IQ-TREE (parameters: -nt AUTO -m MFP + MERGE -alrt 1000 -bb 1000 -bspec GENESITE) (Nguyen et al., 2015) with ultrafast bootstrap (Hoang et al., 2018). The tree was rooted using the single early diverging chytrid species, *Geranomyces variabilis* (Gervar1), and displayed using iTOL (Letunic and Bork, 2019). The final curated alignment and phylogenetic tree files are provided in Supplementary Material S5. The obtained phylogenetic species tree is consistent with previously published ones that were built with genomic datasets (Robbertse et al., 2006; Wang et al., 2009; Ebersberger et al., 2012; Spatafora et al., 2017).

### Reconciliation Analysis

Reconciliation between the gene trees and rooted species tree was performed using NOTUNG 2.9 (Stolzer et al., 2012). Rearrangements in the gene trees were performed when weak edges were present, using an edge weight threshold of 98 (ultrafast bootstrap value). The reconciliation was then performed using the DTL (Duplication-Transfer-Loss) model with duplication, loss, transfer and co-divergence costs of 1.5, 1, 6 and 0, according to a previously published analysis of metabolic enzymes (Wisecaver et al., 2014). Reconciliation without inferring transfer with the DL mode was also performed.

### Protein Domain Analysis

All proteins from type III PKS gene clusters as predicted by antiSMASH were scanned with HMMER using the Pfam database (v32). Predicted BGCs from each defined monophyletic clade were analyzed with BiG-SCAPE (Navarro-Muñoz et al., 2019) in order to identify BGC families. When calculating distances between BGCs to build families, BiG-SCAPE assigns weights to the different distance components. By default, these weights differ between the “type I PKS” class and “PKS Other biosynthetic” class that includes type III PKSs and other types of (bacterial) PKSs. We assumed that fungal type III PKS BGCs do not fundamentally differ from type I PKS BGCs. Therefore, we manually assigned the same weights as the “type I PKS” class (J = 0.22, DSS = 0.76 and AI = 0.02) to the “PKS Other biosynthetic” class and we doubled the domain sequence similarity subcomponent of the anchor type III PKS domains. We did not apply the last step of the BiG-SCAPE algorithm which calls an affinity propagation clustering algorithm to separate high-density networks of mostly similar BGCs. Instead, we considered each network (cutoff = 0.5) as a representation of a gene cluster family. For each monophyletic clade *C*, the occurrence *O* of domain *d* is calculated as the normalized sum of all the occurrences of that domain within each gene cluster family *n*, counting domain *d* only once per BGC *B*:

$$O_{C,d}=\sum_{n\in C}\frac{\sum_{B\in n}1\,i\,f\,d\in B}{\left|n\right|}$$

Domains from proteins marked as biosynthetic (e.g., KS, KR, etc.) were not considered.

For each phylogenetic clade, the occurrence of frequently observed domains (as shown in Figure 6 and Supplementary Tables S15–S23) was measured in antiSMASH predicted type III PKS BGCs, type I PKS BGCs and other type BGCs. The occurrence was also measured in the rest of the predicted proteomes (i.e., not predicted to be located in a BGC). The total number of genes predicted in the four abovementioned groups was also measured for each clade. Chi-square tests with Yates’ estimation was performed to identify domains significantly enriched in either type III PKS, type I PKS or other type BGCs compared to the predicted “non-BGC” proteome (Supplementary Table S24).

## Results

### Widespread Occurrence of Fungal Type III PKSs in the Fungal Kingdom

**FIGURE 1:**
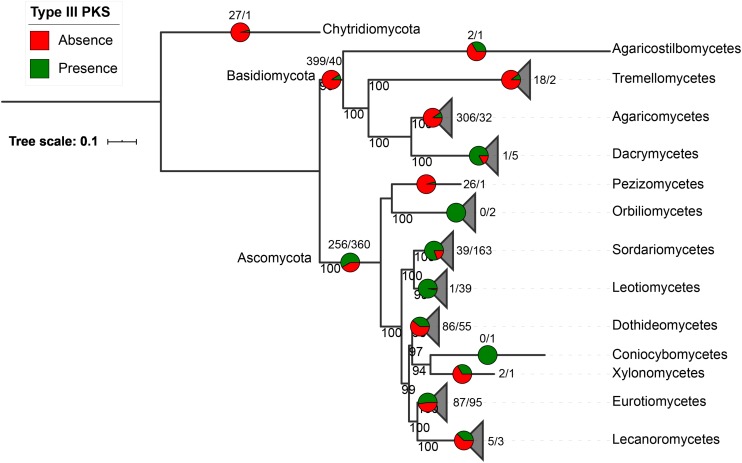
Occurrence of type III polyketide synthases (PKSs) in the fungal kingdom. Presence/absence of fungal type III PKSs in the fungal tree of life. The species tree was built with fungal classes that contain type III PKSs only. The pie charts and numbers at the end of the branches or above the pies indicate, for each phylum and class in which at least one fungal type III PKS was identified, the number of genomes that do not contain a type III PKS (red, first number) or contain at least one type III PKS (green, second number). Bootstrap values above 90 are indicated at the nodes of the tree.

Fungal type III PKS protein sequences were retrieved from 1,193 fungal genomes available at the JGI Mycocosm repository (Grigoriev et al., 2014). A total of 522 type III PKSs were found in only 407 fungal species (34% of all included genomes), 318 of these species carrying a single sequence and 89 having between two and five copies (Supplementary Tables S1–S3). As expected for SM genes, type III PKSs show a discontinuous distribution in the fungal tree of life (Figure 1). They are mostly present in Ascomycota (360 species representing 58% of the available Ascomycota genomes) and they are found to a lower extent in Basidiomycota (40 species representing 9% of the Basidiomycota genomes), with a single early divergent fungal genome belonging to the Chytridiomycota (Figure 1). The presence-absence pattern of type III PKSs in fungal classes is very diverse. Classes like the Dacrymycetes, Sordariomycetes, and Leotiomycetes exhibit conserved type III PKSs in 83, 81, and 97%, respectively, of fungal genomes in these respective classes (Figure 1). In contrast, in most other fungal classes, type III PKs are found in less than 50% of the genomes available in the respective classes. Considering the strict criteria we used to only retrieve likely functional type III PKSs in fungal genomes, these results underestimate the occurrence of type III PKSs in the fungal kingdom.

### The Phylogeny of Type III PKSs Reveals Bacteria-to-Fungus Horizontal Gene Transfers

**FIGURE 2:**
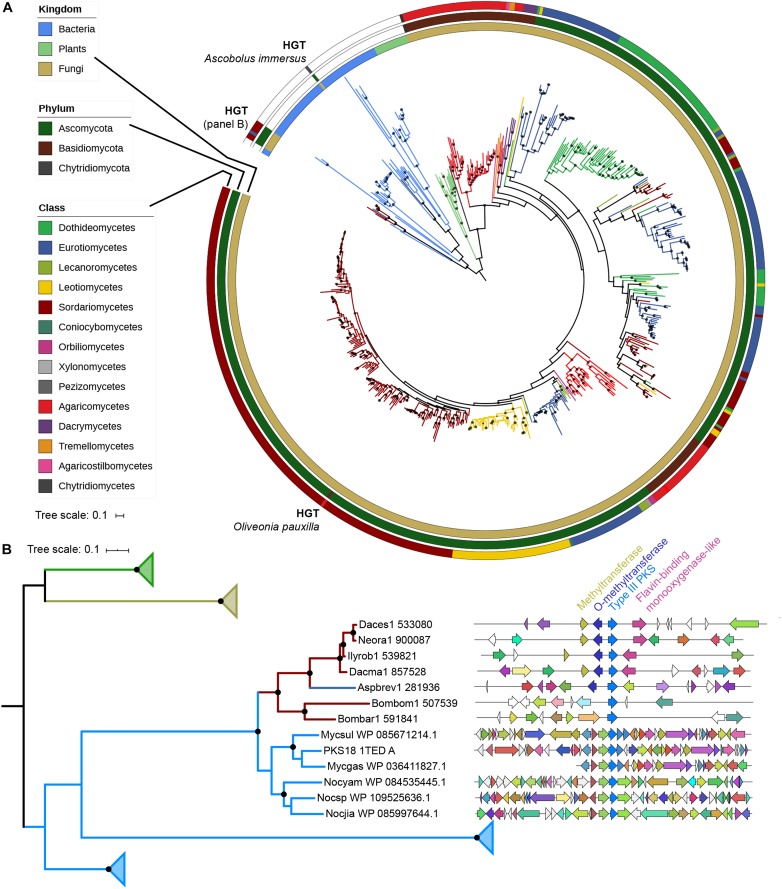
Phylogenetic analysis of type III polyketide synthases (PKSs). **(A)** Maximum-likelihood phylogenetic tree of type III PKSs identified in fungal genomes. Published and characterized type III PKSs from bacteria, plants and fungi are included. The taxonomy of each branch is indicated with colors (kingdom for bacteria and plants; class for fungi) on the three outer circles. Inferred horizontal gene transfers (HGTs) are indicated. The tree was rooted with the bacterial clade. Bootstrap values over 90 are indicated with black dots at the nodes. **(B)** Maximum-likelihood phylogenetic tree of fungal sequences putatively acquired from bacteria with their most homologous bacterial sequences (Supplementary Tables S4–S11) and characterized plant, bacterial and fungal type III PKS sequences. Only the clade with the HGT from bacteria to Sordariomycetes/Eurotiomycetes is not collapsed. The taxonomy color coding indicates the kingdom as in the **(A)** panel. Bootstrap values over 90 are indicated with black dots at the nodes. Synteny between loci of HGT-acquired type III PKSs in Sordariomycetes and Eurotiomycetes. Homologous genes are represented with the same color. A putative gene cluster is predicted in four Sordariomycetes (Daces1: *Dactylonectria estremocensis*; Neora1: *Neonectria radicicola*; Ilyrob1: *Ilyonectria robusta*; Dacma1: *Dactylonectria macrodidyma*) and *Aspergillus brevijanus* (Aspbrev1), while the locus is not conserved in *Bombardia bombarda* (Bombom1) and *Lasiosphaeriaceae sp. AZ0830* (Bombar1). The closest characterized bacterial type III PKS is the tri- and tetraketide pyrone PKS18 1TED_A from *Mycobacterium tuberculosis* (Genbank accession number: YP_177803.1). Mycsul: *Mycobacterium szulgai*; Mycgas: *Mycobacterium gastri*; Nocyam: *Nocardia yamanashiensis*; Nocsp: *Nocardia* sp. SYSU K10002; Nocjia: *Nocardia jiangxiensis*.

A phylogenetic tree with all 522 fungal type III PKSs, together with characterized ones from plants and bacteria, was built (Figure 2A). This tree confirms that plant, bacterial and fungal type III PKSs have evolved independently, forming distinct monophyletic clades. However, eight type III PKSs form two fungal clades within the bacterial clade, indicative of independent bacteria-to-fungi horizontal gene transfers (HGTs) (Figure 2A). The *Ascobolus immersus* type III PKS (Ascim1| 416225) shares 72% identity with a type III PKS from the Actinobacteria *Nocardioides lianchengensis* (Table 1). The locus of this gene in *A. immersus* genome does not contain any other gene of bacterial origin (Supplementary Table S4), ruling out a contamination during genome sequencing. This HGT is consistent with the observation that *A. immersus* is the single genome with a type III PKS within the Pezizomycetes (Figure 1). The other clade corresponds to another HGT event from Actinobacteria. Type III PKSs from the Sordariomycetes *Dactylonectria estremocensis* (Daces1| 533080), *Neonectria radicicola* (Neora1| 900087), *Ilyonectria robusta* (Ilyrob1| 539821) and *Dactylonectria macrodidyma* (Dacma1| 857528) are closely related to PKSs from the *Mycobacterium* genus. The locus in these four species is conserved, the type III PKS gene belonging to a predicted gene cluster consisting of two methyltransferases and a flavin-containing monooxygenase (Figure 2B). The three other fungal species (one Eurotiomycetes and two Sordariomycetes) are closely related to PKSs from the *Nocardia* genus (Table 1). However, a phylogenetic tree that includes all these Actinobacteria sequences supports a single HGT event (Figure 2B). The type III PKS gene in *Aspergillus brevijanus* (Aspbrev1| 281936) is downstream of an O-methyltransferase as found in *Dactylonectria* and related species (Figure 2B and Supplementary Tables S5–S9). Similarly to *A. immersus*, no other bacterial gene can be found at the locus in these fungal species (Supplementary Tables S5–S11). The type III PKS locus in the *Mycobacterium* and *Nocardia* species is conserved for a few genes only, but it does not show any similarity to the fungal locus, suggesting that only the type III PKS gene was horizontally acquired (Figure 2B).

**TABLE 1:** Fungal type III polyketides synthases putatively originating from horizontal gene transfer events.

Species	Protein ID^a^	Best blastp hit^b^	*e*-value	Identity %
*Ascobolus immersus*	416225	Type III polyketide synthase *Nocardioides lianchengensis* (WP_090860924.1)	8e-174	72
*Dactylonectria estremocensis*	533080	Polyketide synthase *Mycobacterium gastri* (WP_036411827.1)	2e-141	56
*Neonectria radicicola*	900087	Type III polyketide synthase *Mycobacterium szulgai* (WP_085671214.1)	3e-140	58
*Ilyonectria robusta*	539821	Type III polyketide synthase *Mycobacterium szulgai* (WP_085671214.1)	2e-144	60
*Dactylonectria macrodidyma*	857528	Type III polyketide synthase *Mycobacterium szulgai* (WP_085671214.1)	1e-142	58
*Aspergillus brevijanus*	281936	Type III polyketide synthase *Nocardia* sp. *SYSU K10002* (WP_109525636.1)	2e-147	59
*Bombardia bombarda*	507539	Type III polyketide synthase *Nocardia jiangxiensis* (WP_085997644.1)	2e-131	61
*Lasiosphaeriaceae* sp. AZ0830	591841	Type III polyketide synthase *Nocardia yamanashiensis* (WP_084535445.1)	4e-150	54
*Oliveonia pauxilla*	744902	Hypothetical protein AK830_g3657 *Neonectria ditissima* (KPM42895.1)	0.0	67

*^*a*^From the Joint Genome Institute Mycocosm repository. ^*b*^Performed with the NCBI nr database. The best bacterial hit is indicated. Genbank accession numbers are indicated in between parentheses.*

The phylogenetic tree also reveals a clear fungus-to-fungus HGT event (Figure 2A). The Basidiomycota *Oliveonia pauxilla* type III PKS (Olipa1| 744902) shares 67% identity with a type III PKS from the Sordariomycetes *Neonectria ditissima* (Table 1). Other genes at this locus in *O. pauxilla* genome share homology with Basidiomycota sequences (Supplementary Table S12), suggesting that this HGT event involved the type III PKS gene only. Other inconsistencies between the gene tree and species tree cannot be reliably assigned to HGTs because of low node support or very mixed topologies.

### The Phylogeny of Fungal Type III PKSs Shows Patterns of Gains Through Gene Duplications, and of Massive Losses

**FIGURE 3:**
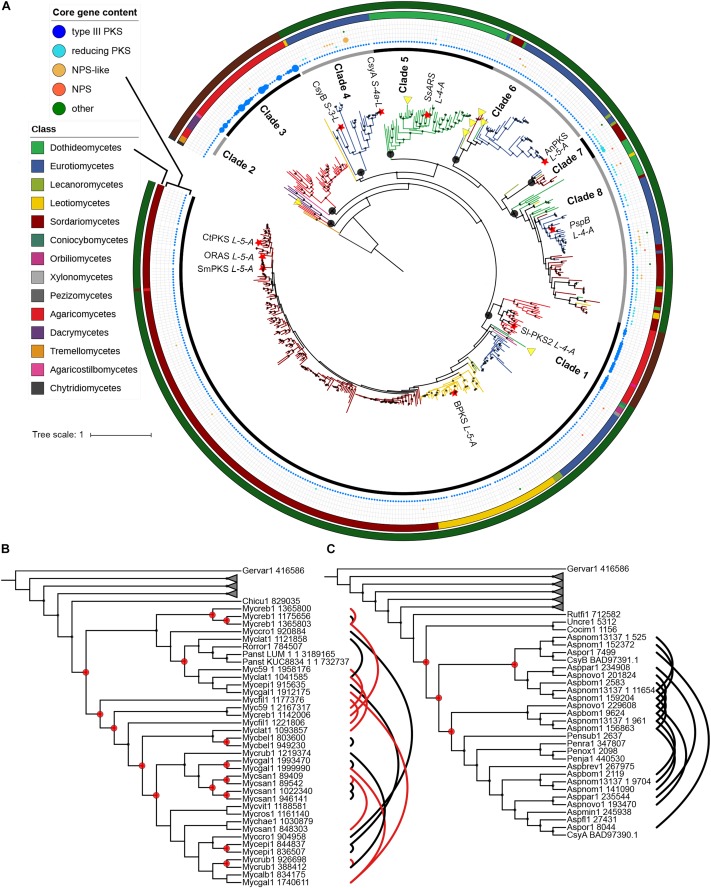
Phylogeny of type III polyketide synthases (PKSs) in the fungal kingdom. **(A)** Maximum-likelihood phylogenetic tree of type III PKSs on fungal origin. Published and characterized fungal type III PKSs are included. The fungal taxonomic class of each branch is indicated with colors on the outer circle. Red stars indicate fungal type III PKSs that have been functionally characterized. The functional classification of characterized type III PKS according to Shimizu et al. (2017) is indicated next to the star. Black circles indicate the nodes that define fungal phylogenetic clades, which are indicated on the inner circle. The middle ring indicates the core gene content in the predicted gene clusters. The size of the circles is proportional to the number of core gene present at the locus. Yellow triangles indicate potential horizontal gene transfers according to the reconciliation analysis; transfers within clades 7 and 8 are not shown because too many events were inferred. The outer circle indicates the phylum: Ascomycota, Basidiomycota and Chytridiomycota are indicated with dark green, dark red and dark gray colors, respectively. Phylogenetic clades 3 **(B)** and 4 **(C)** as defined in **(A)** show four and 15 duplication events, respectively, according to the reconciliation analysis. Other phylogenetic clades were collapsed. Paralogs are linked by black and red lines, the latter linking paralogs located at the same locus. Duplication events are indicated with red circles. Only sub-tree topology is shown. Bootstrap values over 90 are indicated with black dots at the nodes.

A phylogenetic tree with sequences of fungal origin only was built in order to investigate the evolutionary relationships of fungal type III PKSs in more details. Several paralogs due to duplication events can be deduced from this phylogenetic tree, defining eight monophyletic clades with strong bootstrap support (Figure 3A). Within each clade, gene duplication, loss, transfer and co-evolution events were inferred by reconciliation with the rooted species tree using the DTL model (and compared to the DL model) (Stolzer et al., 2012), and manual inspection (Table 2). Clade 1 is conserved from Basidiomycota to Ascomycota and seems to have followed a relatively simple evolutionary route as the gene tree follow the species tree at the class taxonomic level (Figure 3A). Reconciliation with inferring horizontal transfers failed to find temporally feasible solutions and thus could not detect any HGT (Table 2). In addition to the above mentioned HGT from a Sordariomycete to *O. pauxilla*, the phylogenetic tree suggests another HGT to *Zasmidium cellare* (Zasce1| 25004), the only *Dothideomycete* fungus in clade 1 (Figure 3A). *Z. cellare* type III PKS shows highest similarity with the Lecanoromycete *Umbilicaria pustulata* (49% amino acid identity; Supplementary Table S13), suggesting that the donor could have belonged to this class. However, *Z. cellare* type III PKS does not belong to the Lecanoromycetes branch in clade 1 (Figure 3A). In addition, the two upstream genes in *Z. cellare* show highest similarity with genes from another class, the Leotiomycetes (Supplementary Table S13). Both observations suggest an accelerated evolutionary rate at the *Z. cellare* locus that might explain the position of this PKS in the phylogenetic tree (Gabaldón and Koonin, 2013). When transfers are not inferred, the reconciliation analysis reports 34 gains and 126 losses (Table 2 and Supplementary Material S6).

**TABLE 2:** Evolutionary events inferred in the type III PKS phylogenetic tree from reconciliation with the fungal species tree.

Clade	Duplications	Transfers	Losses	Number of optimal solutions
1	-(34)	-(0)	-(126)	-(1)
2	0 (1)	1 (0)	3 (10)	2 (1)
3	15 (15)	0 (0)	25 (25)	1 (1)
4	4 (4)	0 (0)	31 (31)	1 (1)
5	3 (5)	1 (0)	23 (30)	1 (1)
6	6 (8)	4 (0)	38 (72)	16 (1)
7	0 (1)	5 (0)	3 (41)	16 (1)
8	3 (19)	26 (0)	46 (290)	1,382,400 (1)
*Total*	*31 (87)*	*37 (0)*	*169 (625)*	*–*

*Reconciliation was performed using Notung 2.9 with the DTL model for each phylogenetic clade. No co-divergence was detected. Numbers between parentheses are results obtained with the DL model.*

Clades 3, 4, and 5 are specific to the Agaricomycetes, Eurotiomycetes and Dothideomycetes, respectively (Figure 3A). No transfer was inferred in the first two clades, while a single transfer was inferred in the latter in *Paraconiothyrium sporulosum* (Parsp1| 1186637; Table 2 and Supplementary Material S6). However, this transfer is not strongly supported because of a lower bootstrap support and longer distance for this branch (Figure 3A). Although these three clades comprise a limited number of homologs, up to 15 duplication events were inferred in clade 3 and up to 31 losses were inferred in clade 4 (Table 2 and Supplementary Material S6). Especially in clades 3 and 4, recent duplication events were identified (Figures 3B,C). Paralogs in clade 3 are mostly present in the genus *Mycena*. As the Agaricomycetes *Trametopsis cervina* (Trace1) genome contains a paralog in both clades 1 and 3, an ancestral duplication event followed by losses is likely at the origin of the divergence between these Basidiomycota-containing clades.

Clades 2 and 6 comprise homologs from different classes of Basidiomycota and Ascomycota, respectively (Figure 3A). One transfer is inferred within the Basidiomycota clade 2, but the alternative inference of one duplication and 10 losses with the DL model appears as likely when the frequency of duplications in other Basidiomycota clades is considered (Table 2 and Supplementary Material S6). Four transfers are inferred in clade 6, likely due to the position of the Eurotiomycetes *Exophiala sideris* (Exosi1| 115876), the Lecanoromycetes *Cladonia grayi* (Clagr3| 8243) and the Dothideomycetes *Trypethelium eluteriae* (Tryvi1| 523642) type III PKSs (Figure 3A and Supplementary Material S6). The position of these homologs in clade 6 is similar to the *Z. cellare* homolog in clade 1 and thus an accelerated evolutionary rate could also be invoked to explain this topology (Gabaldón and Koonin, 2013), especially because the alternative inference of 8 duplications and 72 losses seems plausible (Table 2 and Supplementary Material S6).

Finally, clades 7 and 8 show a mosaic patterns of homologs from diverse taxonomic classes (Figure 3A). As expected, the reconciliation analysis favors transfer events, inferring 5 and 26 HGTs in clades 7 and 8, respectively (Table 2 and Supplementary Material S6). Reconciliation without inferring transfers in clade 8 results in a scenario of 19 duplications and 290 losses, while a single duplication and 41 losses are invoked in clade 7 (Table 2 and Supplementary Material S6). The observation that the other clades appear to be restricted to a few fungal taxa only or follow the species tree suggests that different selection pressures are acting on sequences in clades 7 and 8. Sequences in the other clades seem to be under purifying selection. Overall, the evolutionary history of fungal type III PKSs has been marked by a remarkable number of gene duplication and gene loss events, as well as potential HGT events.

### Genetic Linkage Between Type III Polyketides Synthase Genes and Other SM Core Genes

The vast majority (474) of fungal type III PKS genes are located in regions that do not contain any other core SM gene. Thirty loci contain two to four type III PKS genes (Figure 3A, Supplementary Table S14, and Supplementary Material S7). These loci are restricted to Basidiomycota and correspond to tandem gene duplications as indicated by the close phylogenetic relationship of paralogs found at the same locus (Figure 3B, Supplementary Materials S7, S8). In *Phanerochaete carnosa* (Phaca1), the two type III PKS copies are located next to each other in opposite directions (Supplementary Material S8). The deletion of the orthologs of each of these copies in the closely related fungus *S. laxum*, showed that only one copy is responsible for the production of resorcinols, while the other appears non-functional (Sun et al., 2016). Experiments are needed to determine whether all duplicated copies in that clade have been inactivated after duplication or some have remained functional.

**FIGURE 4:**
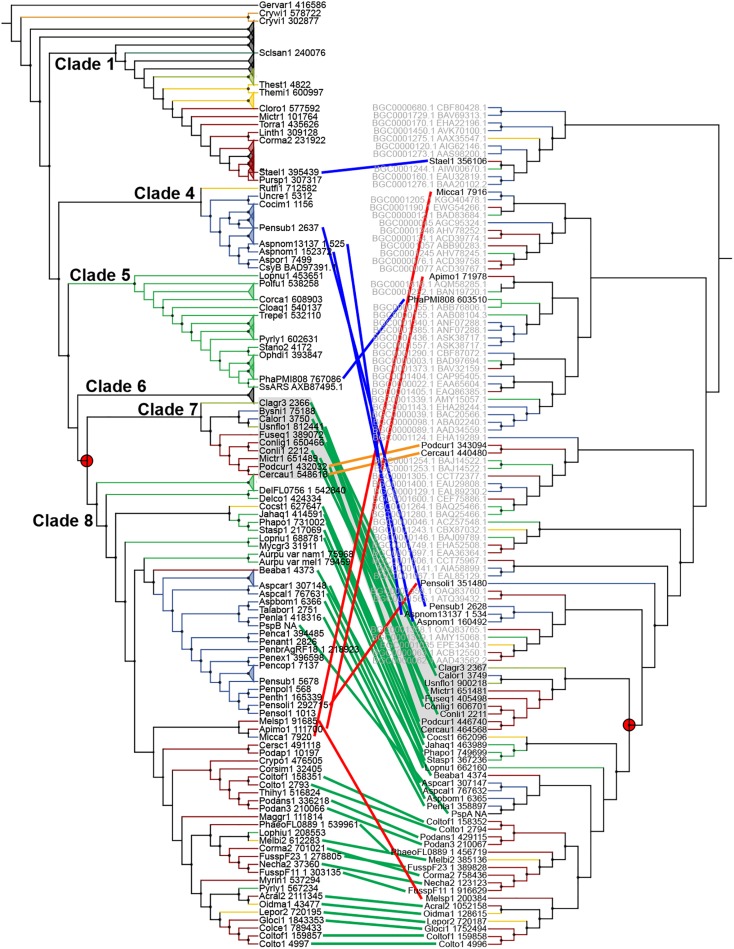
Co-evolution of type III and reducing polyketide synthases (PKSs). Maximum-likelihood phylogenetic trees of genetically linked type III PKSs **(left)** and reducing PKSs **(right)**. The rPKS tree comprises characterized fungal enzymes from the Minimum Information about a Biosynthetic Gene cluster (MIBiG) database (name written in gray with GenBank accession number) (Medema et al., 2015). Light green lines connect genes that co-evolved according to the common gene tree topologies. Red lines indicate linked genes that do not follow the expected tree topology. The orange lines indicate additional linkage with a second rPKS. Blue lines indicate independent linkage events. The red circles show the duplication event that resulted in the divergence of clades 7 and 8.

**FIGURE 5:**
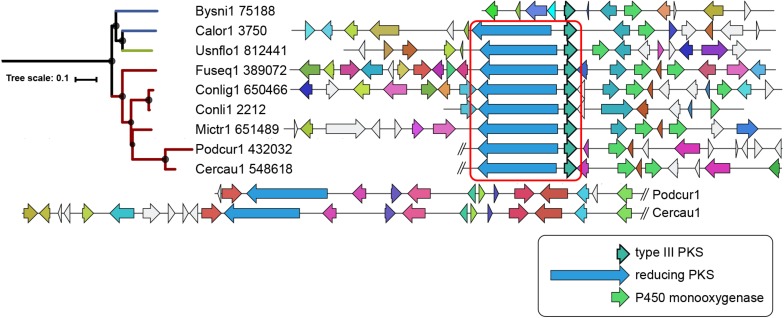
Locus organization of predicted mixed type III and reducing polyketide synthases (PKSs) gene clusters. The regions are shown as reported by antiSMASH 4.0 (Blin et al., 2017) for clade 7. The core genes in opposite direction are indicated within the red square. Homologs in the different species are indicated with the same color code. The loci in Podcur1 and Cercau1 genomes were longer and thus split as indicated with the double slash. The maximum-likelihood phylogenetic tree shown on the left is the clade 7 branch from the large tree in Figure 3A.

Sixty-seven type III PKS genes are located at a genomic locus that contains another type of SM core gene encoding either a reducing PKS (rPKS), non-reducing PKS (nrPKS), non-ribosomal peptide synthetase (NPS), NPS-like or terpene cyclase (TC) (Figure 3A). Fungal type III PKSs are most frequently found associated with rPKSs. Remarkably, type III PKS genes located next to a rPKS gene mostly belong to clades 7 and 8, suggesting ancestral linkage between both genes (Figure 3A). To address this hypothesis, a phylogenetic tree of rPKSs associated with type III PKSs was built. Both phylogenetic trees harbor the same topology (Figure 4), confirming the co-evolution of both genes. Both type III PKSs and rPKSs phylogenetic trees are consistent with a duplication event at the origin of the divergence between clades 7 and 8 (Figure 4). Examination of the loci revealed the two PKS genes are located next to each other, often in opposite directions (Figure 5 and Supplementary Material S8). In *Paecilomyces niveus* (Bysni1), remnants of the rPKS are found upstream of the type III PKS, explaining that it was not predicted as a full rPKS (Figure 5). In *Podospora curvicolla* (Podcur1) and *Cercophora caudata* (Cercau1), a second rPKS is found at the locus (Figure 5) and likely corresponds to the merging of another rPKS gene cluster with the type III PKS gene cluster, consistent with the distant phylogenetic relationship between both rPKSs (Figure 4). *Penicillium solitum* (Pensoli1) is the sole species with a locus harboring a type III PKS and rPKS in a *Penicillium*-specific branch of clade 8, suggesting a loss in the ancestor of these species because no close homolog could be retrieved using BlastP search (Figure 4). Both genes at the locus are not consecutive like in other fungal species, suggesting rearrangements between two PKS loci in this species (Supplementary Material S8). Consistently, the rPKS from this locus is not closely related to other rPKSs associated with type III PKS clades 7 and 8 (Figure 4). Three similar independent rearrangements that merged type III PKS and rPKS genes have occurred in *Stachybotrys elegans* (Stael1), *Phaeosphaeriaceae* sp. PMI_808 (PhaPMI808) and in an ancestor of *Penicillium subrubescens* (Pensub1) and *Aspergillus nomius* (Aspnom1 and Aspnom13137_1) (Figure 4). Finally, independent rearrangements might have occurred in *Melanconium* sp. NRRL 54901 (Melsp1), *Apiospora montagnei* (Apimo1) and *Microsporum canis* (Micca1) because the three rPKSs do not seem closely related (Figure 4). Most of the loci with linked type III PKS and rPKS also contain genes that encode cytochrome P450 monooxygenases, also suggesting an ancestral linkage. However, although the two PKSs may function together to produce the same intermediate in all fungal species, the diversity of tailoring genes predicted at these loci suggests that different compounds are likely produced (Figure 5 and Supplementary Material S8).

Twenty-six fungal type III PKS genes are also found next to NPS and NPS-like genes (Figure 3A and Supplementary Table S14). This type of mixed gene cluster has been functionally characterized in bacteria and it was suggested that the type III PKS, with other tailoring enzymes, is involved in synthesizing a non-proteogenic amino acid for one of the NPS modules (Chen et al., 2017). The single predicted gene cluster comprising type III PKS and TC genes in *Spathularia flavida* (Spafl1) could be responsible for the production of a prenylated polyketide (Figure 3A). The prediction of mixed gene clusters require further experimental investigation in order to validate the hypothesis that fungal type III PKSs can provide/accept precursors to/from other core SM enzymes, increasing the diversity of compounds fungi can produce.

### Fungal Type III PKS Predicted Biosynthetic Gene Clusters

**FIGURE 6:**
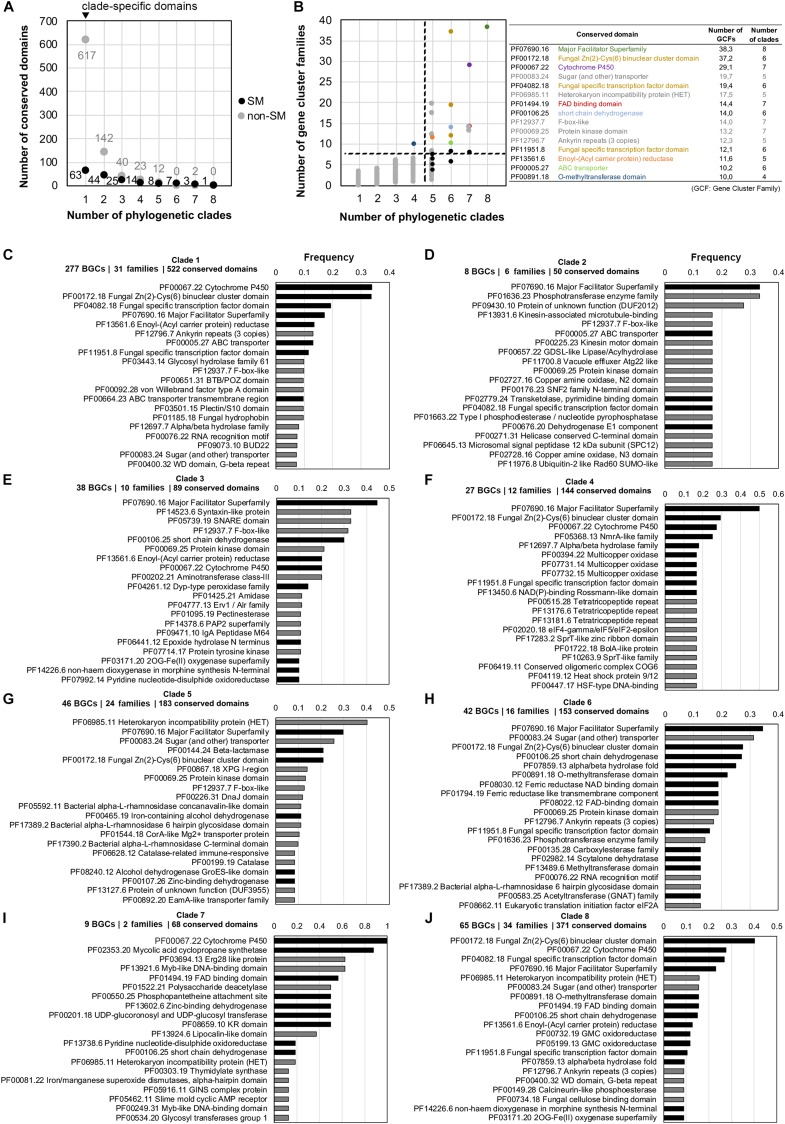
Occurrence of conserved protein domains at fungal type III polyketide synthase (PKS) loci. For each phylogenetic clade, regions that contain a type III PKS as reported by antiSMASH 4.0 (Blin et al., 2017) were processed using BIG-SCAPE (Navarro-Muñoz et al., 2019) in order to build gene cluster families and avoid bias of closely related fungal species. Conserved domains identified using the Pfam database (El-Gebali et al., 2019) were counted in each region (excluding domains found in core enzymes) and normalized to the total number of gene cluster within each gene cluster family. **(A)** Number of conserved domains associated or not with fungal secondary metabolism (SM) and which are found in one to eight type III PKS phylogenetic clades. The black dots indicate SM domains and the gray dots indicate domains that are not associated with fungal SM (non-SM). **(B)** Occurrence in gene cluster families of the 1,001 conserved domains found in 1–8 type III PKS phylogenetic clades. Black dots indicate the most frequent conserved domains typically associated with fungal SM and the gray dots indicate non-SM domains. The name and occurrence of the most frequent conserved domains are indicated on the right with the non-SM domains written in gray. The colored dots correspond to the colored text. The dashed lines separate domains that occur frequently in gene cluster families and in many phylogenetic clades (upper right corner) from low frequency domains (lower left corner). **(C–J)** Frequency of the top 20 most frequent conserved domains in each clade, calculated as domain occurrence divided by the number of gene cluster families in the corresponding clade: black bars indicate conserved domains reported to play a role in fungal SM and gray bars indicate conserved domains that are not commonly found in SM biosynthetic pathways. BGC: Biosynthetic Gene Cluster.

Apart from the soppiline BGC in *P. soppi* (Kaneko et al., 2019), only single type III PKSs have been characterized in fungi. In *P. soppi*, the BGC comprises a cytochrome P450 monooxygenase gene in addition to the two core type III and reducing PKS genes (Kaneko et al., 2019). As mentioned above in clades 7 and 8, fungal type III PKS genes are located at loci that comprise genes encoding diverse tailoring functions (Figure 5 and Supplementary Material S8). In order to gain more insights about the diversity of putative type III PKS BGCs in fungi, the composition in conserved protein domains of the BGC regions identified by antiSMASH was analyzed in each phylogenetic clade. BGC families were determined using BiG-SCAPE in order to avoid any bias in frequencies due to very closely related species. The normalized occurrence of conserved domains in each clade thus represents the number of BGC families in which they are found (Supplementary Table S15). In total, 1,001 different conserved domains were identified, but only 165 could manually be assigned to fungal secondary metabolism, suggesting that antiSMASH reports large regions with many genes unlikely to belong to BGCs. Consistently, the vast majority of protein domains is specific to a single phylogenetic clade (Figure 6A), and occurs in less than five families (Figure 6B). In contrast, most of the protein domains present in higher frequency (in more than 10 families) and found in several clades are typical of SM BGCs (Figure 6B). The three most frequent conserved domains found at type III PKS loci correspond to major facilitator superfamily (MFS) transporters, fungal specific transcription factors and cytochrome P450 monooxygenases. Indeed, MFS transporter (PF07690) is the only conserved domain found in all eight clades; cytochrome P450 (PF00067), FAD-binding (PF01494) and alcohol dehydrogenase GroES-like (PF08240) domains are found in seven clades in relatively high frequencies; conserved domains for transcription factors (PF00172 and PF04082), dehydrogenases (PF00106 and PF00107), ABC transporters (PF00005) and methyltransferases (PF13489) are all found in six different clades (Figure 6B and Supplementary Table S15). Remarkably, five conserved domains that have not been linked to fungal SM are frequently found in several clades (Figure 6B): F-box-like (PF12937), protein kinase (PF00069), sugar (and other) transporter (PF00083), HET heterokaryon incompatibility protein (PF06985) and ankyrin repeats (PF12796) conserved domains. All these domains are also found in other types of BGCs (Supplementary Table S24). The HET and protein kinase domains are actually found in high frequency and are not statistically enriched in any of the predicted type III PKS BGCs (Supplementary Table S24). In contrast, F-box-like, sugar transporter and ankyrin repeats domains are statistically enriched in certain BGCs, suggesting that they may play a role in these biosynthetic pathways.

At the phylogenetic clade level, the composition of predicted BGCs significantly differ (Supplementary Tables S16–S23 and Supplementary Material S8). Cytochrome P450 and transcription factor conserved domains are frequently found next to the type III PKS in clade 1, while MFS transporter, ABC transporter and enoyl reductase domains are found in about 10% of the BGCs (Figure 6C). In clades 2 and 3, the MFS transporter domain is frequently found, but a common BGC is not detected (Figures 6D,E and Supplementary Material S8). The MFS transporter domain shows also highest frequency in clades 4 and 6, but other conserved domains related to fungal SM define sub-BGCs in these two clades (Figures 6F,H and Supplementary Material S8). In the related clades 7 and 8, although both share an rPKS, the loci comprise different conserved domains. Cytochrome P450 and mycolic acid cyclopropane synthetase domains are shared by nearly all loci in clade 7 (Figure 6I), while transcription factor, cytochrome P450 and MFS transporter domains are found in 20–40% of loci in clade 8 (Figure 6J). Finally, the most frequent domain in clade 5, the HET domain, is not known to be involved in fungal SM, but about 30% of the loci also encode an MFS transporter domain (Figure 6G).

**FIGURE 7:**
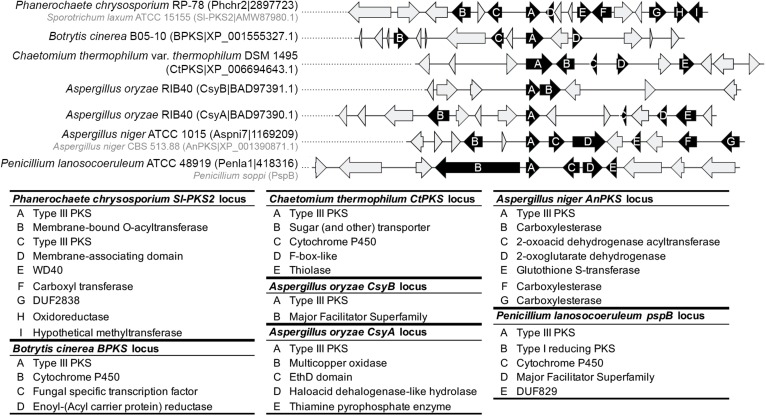
Predicted gene clusters of characterized fungal type III polyketides synthases (PKSs). The locus of type III PKSs was manually inspected for the presence of conserved protein domains that are associated with fungal secondary metabolism. Genes predicted to belong to a putative gene cluster are represented as black arrows and the lower panel indicates the predicted functions. Gray arrows indicate genes that are not expected to belong to the gene cluster. The genomes that contain *Sl-PKS2*, *AnPKS* and *PspB* were either not included in this study or were not available. For these three genes, the closest orthologous locus was inspected as the locus composition is likely conserved (see Supplementary Material S7).

No obvious BGC could be determined at the orthologous locus of the ORAS, SmPKS and SsArs type III PKS genes. The other characterized type III PKS genes seem to belong to BGCs (Figure 7 and Supplementary Material S8). In *A. oryzae*, CsyB appears to be located next to an MFS transporter gene only, suggesting that the csypyrone compounds reported for this PKS might be the final compounds (Seshime et al., 2010a; Hashimoto et al., 2013). In contrast, CsyA seems to belong to a BGC that comprises four putative tailoring genes encoding a multicopper oxidase, an EthD domain-containing decarboxylase (Griffiths et al., 2016), an haloacid dehalogenase-like hydrolase and a thiamine pyrophosphate enzyme (Figure 7). Further analyses are needed to validate the role of these tailoring genes in the modification of the polyketide 3,5-dihydroxybenzoic acid released by CsyA (Seshime et al., 2010b). Similarly, while the activity of AnPKS and An-CsyA is known from recombinant proteins only (Li et al., 2011; Kirimura et al., 2016), the corresponding gene is located at a well conserved locus in *Aspergillus* species (Supplementary Material S8), which comprises putative tailoring genes encoding carboxylesterases, a 2-oxoacid dehydrogenase acetyltransferase, a 2-oxoglutarate dehydrogenase and a glutathione-S-transferase, which could be involved in detoxification (Dasari et al., 2018) (Figure 7). Thus, it is likely that AnPKS produces a polyketide backbone that is further modified. The characterized type III PKS genes within clade 1 appears to belong to different BGCs. The CtPKS gene is located next to P450 and thiolase genes, as well as a sugar transporter and F-box-like genes, which function in fungal SM remains to be demonstrated (Figure 7). *B. cinerea* BPKS gene is located next to P450, enoyl(-acyl carrier protein) reductase, and transcription factor genes (Figure 7). Here again, the compounds produced by these biosynthetic pathways are likely different from the compounds reported from the expression of recombinant proteins. The Basidiomycota Sl-Pks2 type III PKS was shown to be responsible for the production of spirolaxine. A biosynthetic route has been proposed based on the activity of the recombinant protein and includes three hydroxylation, one methylation and three ring formation steps (Sun et al., 2016). Sl-Pks2 closest orthologs belong to a putative BGC that is not fully consistent with these predicted steps: genes encoding putative membrane-bound *O*-acetyl transferase, membrane-associating domain (MARVEL) protein, WD domain protein, carboxyl transferase, DUF2838 protein of unknown function, oxidoreductase and hypothetical methyltransferase are found at this locus (Figure 7). Finally, the soppiline BGC in *P. soppi* was shown to contain an rPKS and a P450 gene (Kaneko et al., 2019), but the BGC might also comprise genes encoding an MFS transporter and a protein of unknown function (DUF829) (Figure 7).

## Discussion

Polyketides produced by type III PKSs exhibit a diverse range of biological activities and are interesting chemical backbone for the synthesis of active compounds (Austin and Noel, 2003; Lee, 2015; Lim et al., 2016). They have been particularly studied in plants, especially flavonoid compounds that protect them from biotic and abiotic stresses (Ferrer et al., 1999; Austin and Noel, 2003). Type III PKSs have been discovered in fungi only at the beginning of the 21st century thanks to genome analyses (Seshime et al., 2005) and the first fungal type III PKS was functionally characterized in 2007 (Funa et al., 2007). The number of characterized fungal type III PKSs has doubled in the last couple of years (Ramakrishnan et al., 2018; Yan et al., 2018; Kaneko et al., 2019; Manoharan et al., 2019). However, most of these studies result from the random selection of fungal species. It is therefore timely to provide a reliable basis for the further characterization of new fungal type III PKSs and their BGCs, which will ultimately allow the rational exploitation of these biosynthetic pathways to produce new active molecules.

In this work, we present the first evolutionary study of fungal type III PKSs at the kingdom level. The phylogeny of these enzymes allowed the definition of eight phylogenetic clades that have likely originated from ancestral gene duplications as clearly shown for clades 1 and 3, and for clades 7 and 8 (Figures 3A, 4). This phylogeny is rather complex, making the inference of evolutionary events a difficult task. However, it appears that the discontinuous distribution of type III PKS genes in the fungal tree of life (Figure 1) is mainly due to duplications (between 31 and 87 events depending on the inference of transfers) and massive losses (between 169 and 625 events depending in the inference of transfers) (Table 2). Especially, Basidiomycota have experienced recent gene tandem duplication events, resulting in loci with multiple type III PKS paralogs (Figures 3A,B). Duplication has previously been reported as the major mechanism that drives gene cluster evolution (Wisecaver et al., 2014; Marcet-Houben and Gabaldón, 2019). In our analysis, gene losses appear to occur 5–7 times more frequently than gene duplications. This ratio is consistent with a previous reconstruction of gene duplication and loss in Pezizomycotina (Wapinski et al., 2007) and with the observation in *S. cerevisiae* that duplicated gene copies can be lost as soon as after tenth of generations (Naseeb et al., 2017). The reconciliation analysis inferred 37 HGT events in clades 2, 5, 6, 7, and 8, a low number considering that evolutionary events could not be inferred with transfers in clade 1 (Table 2). However, these predictions would indicate a minimum HGT frequency of 7% for fungal type III PKSs. This number is nearly the double of the reported HGT frequency of 4% for metabolic enzymes (Wisecaver et al., 2014), but is much lower than the 25–35% reported for SM gene clusters by Marcet-Houben and Gabaldón (2019). Manual inspection of inferred HGTs in our study suggests that the total number of events is actually overestimated by reconciliation software like NOTUNG, which has been recently reported (Villani et al., 2019). The weak support of certain branches is likely due to accelerated evolution in certain fungal species, as exemplified with *Z. cellare* (Zasce1) in clade 1 and *P. niveus* (Bysni1) in clade 7. Genes do not necessarily evolve at a gene-specific rate, but their evolution speed can vary between orthologs and evolutionary rates in duplicated genes can also vary greatly (Gabaldón and Koonin, 2013). Newly acquired genes seem to evolved faster than core conserved genes that are under purifying selection (Wolf et al., 2009). It was shown that orthologous genes can experience accelerated evolution in certain organisms only (Ely et al., 2019). Similarly, recently duplicated paralogs appear to evolve faster than orthologs with the same level of divergence and the duplicated genes will thus evolve toward different fates (Kondrashov et al., 2002; Innan and Kondrashov, 2010). In addition, the difficulties to infer co-divergence events (Xu and Yang, 2016), especially in ancestors, also contribute to favoring HGT events. The reconciliation analysis, although using no cost for co-divergence, did not infer any event of this kind. However, absence of co-divergence appears very unlikely considering that gene clusters are polymorphic within fungal populations, including ancestral ones (Ward et al., 2002; Lind et al., 2017). These different aspects in gene evolution are difficult to estimate and are likely to yield inconsistencies between gene and species trees.

Independently from the reconciliation analysis, several obvious HGT events were detected, one between a Sordariomycete fungus and the Basidiomycota *O. paxilla*, and three transfers from Actinobacteria to Sordariomycetes and Eurotiomycetes species (Figure 2). HGT of type III PKSs from Actinobacteria to Proteobacteria were previously reported (Gross et al., 2006), suggesting that Actinobacteria could be a recurrent source of new type III PKS genes in diverse organisms. To the best of our knowledge, bacteria-to-fungi HGTs were suggested only for 6-methylsalicylic acid synthase (MSAS)-type I reducing PKSs and for a hybrid non-ribosomal peptide synthetase-PKS enzyme from *Cochliobolus heterostrophus* (Kroken et al., 2003). MSAS-like rPKSs form the outgroup branch in the rPKS phylogenetic tree (Figure 4), consistent with an ancestral HGT event from bacteria (Kroken et al., 2003). The MSAS-like rPKS in *S. elegans* (Stael1) is the only example of linkage between this type of rPKS genes and type III PKS genes. Based on similarity, only transfer of the single type III PKS gene was detected. Remarkably, one of these transferred type III PKS genes is located next to three other genes of fungal origin and which encode enzymes with activities commonly found in SM biosynthetic pathways, namely two methyltransferases and one flavin-binding monooxygenase (Figure 2). The type III PKS gene could have inserted in between these genes after transfer, or these so-called tailoring genes could have been recruited after transfer to form a functional BGC. Experimental validation is needed to determine whether a bacterial type III PKS acquired through HGT has evolved to function with eukaryotic tailoring enzymes.

In most Ascomycota classes, type III PKS genes are present in at most 50% of the genomes included in this study. However, the Pezizomycetes have experienced nearly complete loss while nearly all Orbiliomycetes and Leotiomycetes genomes contain a type III PKS gene (Figure 1). Similarly, while the majority of Basidiomycota genomes do not contain a type III PKS gene, nearly all genomes from the Dacrymycetes class contain at least one (Figure 1). Fungal species within these three classes are not very closely related, meaning that a bias in the sampling for sequencing does not explain the conserved presence of type III PKSs. The evolutionary forces that contribute to the discontinuous distribution of type III PKSs in the fungal tree of life and their complete loss or conservation in different taxonomic classes are difficult to determine. The large diversity of lifestyles within each fungal class makes it difficult to correlate the presence of type III PKSs to specific lifestyles or ecological niches. Further studies are needed, ideally at the population level, to obtain insights in the evolutionary forces that are acting on these biosynthetic pathways. It must be noted that our search for type III PKS genes in fungal genomes was rather strict and they are likely present as wrongly predicted genes in a number of genomes.

Our phylogenetic and comparative genomics analyses have identified clades 7 and 8 which comprise type III PKS genes that are linked to an rPKS. As expected from the co-evolution of both genes, their recent functional analysis in *P. soppi* showed that the rPKS provides the starter unit to the type III PKS (Kaneko et al., 2019). The P450 monoxygenase gene found at this locus is involved in the oxidation of the polyketide released by the type III PKS (Kaneko et al., 2019). This P450 gene is conserved in nearly a third of BGCs predicted in clade 8, suggesting that similar compounds are likely produced in these species (Figure 6). However, many other genes encoding enzymes with activities commonly found in SM biosynthetic pathways are found and are expected to contribute to the diversification of polyketides produced by pairs of rPKSs and type III PKSs. Similarly, the sister clade 7 comprises a P450 gene in all species, but most predicted BGCs also appear to contain a gene that encodes a predicted mycolic acid cyclopropane synthetase (Figure 6). Functional analyses are needed to illuminate the diversity of compounds produced by BGCs from these two particular clades. In other phylogenetic clades, P450, MFS and transcription factor genes are frequent and likely form a core BGC in many cases (Figure 6). In each clade, sub-BGCs have likely emerged by recruiting additional tailoring genes encoding diverse functions. Functional analyses based on these predictions are likely to provide a large diversity of polyketides with potentially interesting biological activities.

In addition to the conserved domains that are commonly found in fungal BGCs, our analyses highlighted the high frequency of conserved domains with no known function in fungal SM, especially sugar transporters, HET proteins, F-box like proteins, protein kinases and ankyrin repeats (Figure 6). In clade 2, a protein of unknown function is also frequently found and several other proteins of unknown functions could be found at the locus of characterized type III PKS genes. These observations could be due to the localization of type III PKSs at conserved loci and these genes could indicate BGC borders. For example, the HET proteins and protein kinases are not enriched in fungal BGCs, suggesting that they are not involved in fungal secondary metabolism (Supplementary Table S24). Alternatively, these domains could be involved in biosynthetic pathways and their roles need to be determined experimentally. For example, F-box and ankyrin conserved domains are structural domains involved in protein dimerization (Bai et al., 1996; Michaely et al., 2002) and these protein may interact with enzymes from a given pathway to modify or enhance their enzymatic activities. The ankyrin repeats, F-box-like and sugar transporter domains are significantly enriched in BGCs from clades 1 and 6, 3 and 5, and 1 and 5, respectively (Supplementary Table S24). These domains are present in other BGC clades, but they are not significantly enriched, suggesting that these proteins may be involved in certain biosynthetic pathways only. Certain non-SM domains like phosphotransferase enzyme family (PF01636.23), syntaxin-like protein (PF14523.6) and SNARE domain (PF05739.19), and Erg28-like protein (PF03694.13) are found and significantly enriched in single clades 2, 3, and 7, respectively (Figure 6 and Supplementary Table S24). These proteins could play a different role in self-protection against a toxic compound as decoy, a function that has been found encoded within a few fungal BGCs (Yeh et al., 2016; Yang et al., 2018; Kjærbølling et al., 2019). Some proteins with unknown functions could also play a biosynthetic role, as shown for DUF1772 proteins in emodin-like BGCs, which was found to exhibit an anthrone oxidase activity (Lim et al., 2012a, b).

Our analysis is certainly not showing the complete picture because it is restricted to conserved domains from the PFAM database and many genes were not reported to contain any conserved domains. It is likely that many of these genes actually harbor a functional conserved domain either not in the PFAM database, divergent enough to not be detected with the detection thresholds we used, or need to be functionally characterized. A recent example showed that the Orf3 protein from the *ACE1* BGC in *Pyricularia oryzae*, while not harboring any known conserved domain, is likely a Dies-Alderase enzyme (Wang et al., 2019).

The phylogeny of fungal type III PKSs matches the enzymatic activities of the characterized type III PKSs. Consistent with their characterized common enzymatic activity, SmPKS, CtPKS and ORAS are close orthologs in the phylogenetic tree (Figure 3A). BPKS shares the same specificity and belong to the same phylogenetic clade 1, suggesting that most type III PKS in this clade will preferentially produce resorcinols from long starter units. The characterized Basidiomycota ortholog Sl-PKS2 also produce resorcinols from long starter unit, but incorporate fewer malonyl-CoA units (Sun et al., 2016), an activity that is shared with the distant *Shiraia* SsArs and *P. soppi* PspB enzymes from clade 5 and clade 8, respectively (Yan et al., 2018; Kaneko et al., 2019) (Figure 3A). Although the characterized type III PKS from *A. niger*, AnPKS, belongs to the different clade 6 (Figure 3A), it exhibits the same specificity and flexibility than ORAS, SmPKS and CtPKS (Funa et al., 2007; Li et al., 2011; Ramakrishnan et al., 2018). Yet, it was reported that AnPKS can also accept aromatic and branched starter units, which was also reported for FiPKS (Li et al., 2011; Manoharan et al., 2019). CsyA and CsyB from *A. oryzae* are paralogs belonging to clade 3 (Figure 3A). These enzymes appear to produce preferentially pyrones from short starter units (Seshime et al., 2010a, b). Divergence after duplication seems to have modified the processivity of these type III PKSs because CsyB incorporate fewer units than CsyA, and CsyA also uses acetoacetyl extender units (Seshime et al., 2010b). Finally, PspB from clade 8 accepts a linear polyketide produced by the rPKS located in the corresponding BGC (Kaneko et al., 2019). Considering the complex evolution observed in clade 8, it remains to determine whether all rPKSs are producing the same starter units and, if so, how the production of different precursors could have impacted the specificity of the corresponding type III PKSs. No type III PKS from clades 2, 3, and 7 has been characterized so far. Comparison of the sequence logos (Supplementary Material S9) between each fungal clade, plant and bacterial clades (Figures 2, 3A) highlights the highly conserved Cys-His-Asn catalytic triad, as well as the Phe residue that was reported to control product specificity (Rubin-Pitel et al., 2008; Mori et al., 2015). These sequence logos also highlight clade specific sequences, conserved motifs, and conserved amino acids, which require further functional analyses to understand their role in the specificity and activity of fungal type III PKSs.

## Conclusion

Our study provides important information to further characterize fungal type III PKSs and investigate their diversity. In contrast to previous studies, it is now possible to select specific candidate PKSs with likely different activities for characterization. In addition, the domain analysis provides a basis to further study the diversification of chemical structures produced by type III PKS biosynthetic pathways. Thus, our work is expected to promote research on these overlooked enzymes and pathways, and might lead to the production and engineering of novel bioactive compounds.

## Data Availability Statement

All datasets generated for this study are included in the article/Supplementary Material.

## Author Contributions

JC designed the analyses, performed the phylogenetic analyses, analyzed all data, and wrote the manuscript. JN-M performed the domain analyses, built phylogenetic trees, analyzed the data, and revised the manuscript.

## Conflict of Interest

The authors declare that the research was conducted in the absence of any commercial or financial relationships that could be construed as a potential conflict of interest.

## Footnotes

1 hmmer.org

## Supplementary Material

TABLE S1 Genomes used in this study.

TABLE S2 Distribution of type III PKSs in fungal genomes at the phylum level.

TABLE S3 Distribution of type III PKSs in fungal genomes at the class level.

TABLE S4 Best sequence similarity of proteins encoded at the locus of the type III PKS in *Ascobolus immersus*.

TABLE S5 Best sequence similarity of proteins encoded at the locus of the type III PKS in *Dactylonectria estremocensis*.

TABLE S6 Best sequence similarity of proteins encoded at the locus of the type III PKS in *Neonectria radicicola*.

TABLE S7 Best sequence similarity of proteins encoded at the locus of the type III PKS in *Ilyonectria robusta*.

TABLE S8 Best sequence similarity of proteins encoded at the locus of the type III PKS in *Dactylonectria macrodidyma*.

TABLE S9 Best sequence similarity of proteins encoded at the locus of the type III PKS in *Aspergillus brevijanus*.

TABLE S10 Best sequence similarity of proteins encoded at the locus of the type III PKS in *Bombardia bombarda*.

TABLE S11 Best sequence similarity of proteins encoded at the locus of the type III PKS in Lasiosphaeriaceae sp. AZ0830.

TABLE S12 Best sequence similarity of proteins encoded at the locus of the type III PKS in *Oliveonia pauxilla*.

TABLE S13 Best sequence similarity of proteins encoded at the locus of the type III PKS in *Zasmidium cellare*.

TABLE S14 Number of core secondary metabolism genes at type III PKS loci.

TABLE S15 Normalized occurrence of protein conserved domains in predicted type III PKS gene clusters.

TABLE S16 Normalized occurrence of protein conserved domains in predicted type III PKS gene clusters in clade 1.

TABLE S17 Normalized occurrence of protein conserved domains in predicted type III PKS gene clusters in clade 2.

TABLE S18 Normalized occurrence of protein conserved domains in predicted type III PKS gene clusters in clade 3.

TABLE S19 Normalized occurrence of protein conserved domains in predicted type III PKS gene clusters in clade 4.

TABLE S20 Normalized occurrence of protein conserved domains in predicted type III PKS gene clusters in clade 5.

TABLE S21 Normalized occurrence of protein conserved domains in predicted type III PKS gene clusters in clade 6.

TABLE S22 Normalized occurrence of protein conserved domains in predicted type III PKS gene clusters in clade 7.

TABLE S23 Normalized occurrence of protein conserved domains in predicted type III PKS gene clusters in clade 8.

TABLE S24 Enrichment analysis of frequent conserved domains in predicted type III PKS gene clusters.

MATERIAL S1 Protein sequences of type III polyketide synthases retrieved from the Joint Genome Institute genomes.

MATERIAL S2 Protein sequences of characterized type III polyketide synthases used in this study.

MATERIAL S3 Protein sequences of reducing polyketide synthases used in this study.

MATERIAL S4 Protein sequences of bacterial type III polyketide synthases that are closely related to fungal sequences.

MATERIAL S5 Curated protein alignments used to build phylogenetic trees.

MATERIAL S6 Reconciliation analyses performed with DTL and DL models using NOTUNG.

MATERIAL S7 Paralogy in the fungal type III polyketide synthase phylogenetic tree.

MATERIAL S8 Description of fungal type III polyketide synthase loci. Color blocks indicate conserved domains.

MATERIAL S9 Sequence conservation within fungal, plant and bacterial type III polyketide synthases (PKS) phylogenetic clades.
