# Supplementary material for: Evolutionary Histories of Type III Polyketide Synthases in Fungi
Source: Front Microbiol. 2020 Jan 21;10:3018. doi: 10.3389/fmicb.2019.03018 (PMC6985275; doi:10.3389/fmicb.2019.03018)
Supplement: MATERIAL S3 — Protein sequences of reducing polyketide synthases used in this study. [file Data_Sheet_3.docx]

>Jahaq1|463989

MAPHAIEPTTEDSHPFNGHIDVTALTGELNGSNGTTTTAPTNGHLNGTDESKFPEPIAICGMAMRLPGGVRDAEGYWDLL

YNKRSGQCKVPADRYNVEAFYGPGKIGHVNSMHGYFLEGYDLSNIDTTFWTMTKQEIEAMDPQQRMNLEIVYECLQNAGQ

KPSELRGRKIGVYVGTFDGDWLELDGRDTQHYNSYRLTGYADYMSANRVHYEYNFTGPSMTVRTACSSSMSALYDACRSL

LAGDCESAVVATSNLMLSPRTTSTMADQGVMSPTGSCKTFSADADGYARGEAVSAVYIKKLSDAIRDGDTVRSVIRSSVV

NAGGRASTLTAPNTVAHEALIRRGHEAAGITDFSQTAMIECHGTGTAVGDPIETKAVANIFGEHGIYIGSVKPNIGHGES

ASGLSSIIKMTLALEKKIIPPNILFNKPNPKIPFEECKLVVPTEPMPWPVDRAERVGVNSFGVGGSNAFMLLESAEYHGV

GRKEELLEPTWLEPQPKLLVFSAKHPESLKRSAADHESYINSRPESINDMSYTLGVKREVLAHRAFCVTDGQGNFELSRI

QKSTLKSPPSIVFCFTGQGAQSAQMGKELIQNVPSFRKSIEHLDHVLTCLPDPPKWKLLDEIMASKKSSRLGQAELSQPC

CTALQIALVDLLTSWNIHPAAVTGHSSGEIGAAYACGSLSAEDAIISAYYRGQVTVRLSPELVGGMAAIGLGAQDVAKYL

QPGVSIGCENSLESTTLTGDKVILQKVMDVIKEDRPETFVRALAVDKAYHSAHMETVADEYTVLLGKMHAGAPKVPFFSS

VTEKVITDPHILNASYWVKNLISPVKFTGAIGNALDTINAPKIFLEVGPHTALAGPIRQILRAKASQDPYVGTLARGHDA

HEQLLKTAGEVWLNDHAIAFESINGKGNFLTDLPLYPWNYESVWGESRLSRDWRFRKHPHHDLIGSRVLESTDQNPSWRN

ILRSDEIRWVKEHEVTGDVVLPGVGYVCMAGEAVRQLTGTADYTVRKVNIKAACVIQSGQDIELITHLSRARVTDSLESP

WYDFTVSSLNKDTWIQHAFGQIRPGSEIERENVDIKPLPRQLSKRTWYRKMQQMGLNYGTRFRAINNPTSHPTIKSAVAS

VKSELVEGESYYAVHPSAMDSLLQLLMLAVSNGLERRFTVLAVPTYIEELYVRPPVGEMTVQATADFPPKGGAMSGDVVA

IDSEGNTVVQLTTLQTSPLGDGKTDDNKGFNDLHAATELEWKPDLTFMKASDLFSISMERGEIHNNLDKLGALAMLEAEE

RLEGQATEQGHLTIFYNWLKKINAQTDPALTQFDSPQRNQLMDELNDWLQGSTAKAGATAIMRILNKCRGIFQGEVDPLE

LLMDDGILTQLYDFMQMQNSKYTSFIDLLSHKTPTLRILEIGAGTGGTTNTLLPFLKSQYGERTYFSYTYTDISSGFFIQ

ARERFREYPGMEYAVLDISKDPLAQGFEAHAFDMIIATNVLHATPSIHETLSNCHTLLHPRGRLFLQELDPVSKWINFVM

GVLSGWWLGAVDGRPDEPYIPAERWDKELKAAGFDGIDAVHHDGYLDNNIIAMPTRPALASCRITVLCVDPEASKVQTVA

MGLRAKGFELDFCTVGQTPIAEQDAISLLDLDHPFLYSANEAQFLWFVNVVDCIKGSGENHNGILWVTGACQVNCTEPKY

ALITGVSRVCRTERQMDFGTFELDSFDEETLKVVAEVYDEFAQRSHEESMTPDVEWAYDRGKVMVSRYHFIDVAEGMKNT

TVTPEVSTKKLAINKPGVLSTLAWEELELPVLGPNDVECEVKAVGLNFKDVLIAMGIITDLPAIGDGLGLESCSVVTRVG

SEVSSLKIGDRVIGCKTGSFTTKMVLSEKLCVKIPENASLSDEEAATMPVVVCTALYALQDLARLDSSKSILIQSAAGGV

GIAAIQIAKMTGAEIYCTVSTQEKIDFLTQTYGIQADHIFNSRDANFVSGIMAKTEGRGVDVVLNSLSGELLHASWKCVA

EFGIMVEIGRRDFIGHGQLGMEMFDGNRTFVGFDLSTIAAKKQAVMMSIMERALHYNAEGHIKPITPIHEFGAAQIGEAM

RYLQRGTHIGKVVVTMPEDPAALPLKKVRGELRLNPEKAYLFVGGLGGIGRAVASWLVERGARRIVFLSRSAGKDPKTQI

FLHELAAQGCNATTFAADVCNYEDVLKAVESIDKPIGGVLQAAMALADVSLADMTFAQWQYAMLPKVQGTLNLHKALENH

KSSVDIFFTFSSAGSTMGNWGQANYNAGNCFLDAFIQYRHSIGLPASSLDVGVIEDVGYVAENPSMLDTLRATGQYLIQE

RELLESIELCMSRSQPPSLKRKREDNYNGDPDELPRYANPSQVAIGYRSVLPITAPNNRCIWRKDRRMTIYRNLERQETA

AVDSGNGALTTFLKDIISNSTLLKAPESAELLAQAIGKTLFGFLMQEEEDIDLKAPLATIGIDSLISIELRNWIRANIGV

ELTVLEIVRADDIASLGEQAQAKLVEKLVT

>Apimo1|71978

MESNTPLAVVGYAYRAPGVGRKGLWEFLADAKSAWSEVPADRFNKDAFYHAGDKPGFFPSEGGHFLPDDVYAFDASFFNI

KAEEARSLDPQTRLVLECAFEAAESAGLTLPELAGANIGVFAQAGFSEYSMRLVEDLQTINKYAGLGISQSLIPNRLSYF

FGLTGPSVAVDAACAGTTYALHQACQSLRNGECSAALVAAGSIISGPEMWVGLASLGAISPEGKCFSYDSRAAGFGRGEG

GGCLIIKPLADAIACGDPVRAVIRGTACNHSGRSKGITMPSQPAQERLLTTLHDNAKLDPRDTIFVEGHGTGTKAGDPID

AGAIAAVVAKEASSSRPTYIGSAKSNFGHLEGASGMVSVIKAMMMLEQECMLPNANFEEFNPGIVNDGRLKVLTKPLPWP

SDAIRRVCVTNFGFGGSNAAVLLERAPQAQTSGFHAPLVNGISKKAVEVITNGDATPDHALPSRTSNEKLFVVSAKSATS

LEAYISSLAAYLEHQPTSTQFLQDLSYTLSQRRTLFPRHRLAISAESMGSLVEQLRSARSGNKTPETLDSNVAFIFTGQG

AQYFQMASGLEQYEAFYSAIKRAELFLTELGAPWSLTEELSRPQGESRVDDAEISQPACTAIQLALIVLLQSWGISPAAV

VGHSSGEIAAAFAARLISFEAAIAISYFRGVAVNDIILDGLEQGAMLAVGTSAEEAMKLIQEEDAYTTIAAINSHNSVTI

SGDIDAIENIQEKCQERDIFARRLKVDIAYHSRHMEAAATSYLASIEPFCLSSRESPERESSTKPSFISSVTGKQEHADT

VADGSYWVRNLVGTVQFLKALERLQSYDVSGEEKPDTAGSKRLLAIEIGPHSALKNPTMQTMSVLATQNGQAGSNVAYLP

SLIRGKPAPAALMDLAGKLFVSGSDIKLSEVNQLDHVGAKVIHDLPPYEWNKSSRYIHQPRIATKKLFGGEAHSSLLGSK

SPYAEGNEHVYRNVFTLDDIPWIRDHNVAGDVLFPFTGFFSLAVEAFRRVHQSSTSVSNVQVREFHVSKGLRIEEDEQVD

LTTKIRPADIGTQVASNTAWVFEVMSWSNAEGWTVHCRGLVERDHGETFEQSPGVKAAALLLENPASQAMDVEAEYAIQR

QNGIVYGSSFFNTVDMRRLPGAVIHTIKVRDIGAAGPNPDASVLTVDPPTLDSCLQTIGAIQELEGPRPVHVPTHTRRWR

ISNRIVADADHELTIVTRKLSHDVKSGNLHLSYVVFDLSSGSPKPVMEIDNMALKTITQVKDETLMDGLPKTYFLRHVPH

TDHVDGSILAKTITPPAADASELQCRRDWNEVSIIFMKRMVDSAKSHDVTNLPSHLSKFLAWSQRLVAATQPSPGSSLAD

DEQALISRIANSGPAGEMLCKVGDKLPQIIRGEVETLEIMLEGGLLTRYYEQDLATKRSAKALAEYVGLVHECHPEIRIL

EIGGGTGSATLPVLEAMSRWSVASTASAARFQYTFTDISAGFFENARAKIGRWSENITYKKLDIGLDPLEQGFVAEDYDL

IIAANVLHATPDITRTIRNTTTLLKPGGKLVLMELTKTSNPMAFPFASLPGWWLSEDDYRSADGPLLTKDSWRGLLKANG

FPLGIDGFVDDYPGEPERMNTTMWSTKQSPVRDSTERLTVCQASSGMTNDTTYAEMISQTLRTSTKVEPIVSTLLELDLG

SMNCCIVVDNPLRSCFSNPSPEMFSALQKAFTESAAHVLWVIPVNAHPDCALVKGLLRTLRIEDDGKAFVILENATFDKE

GLEAIAQLACRLGDRSILNEQEYSMVDGVLHVPRFCSHEPADQAFAMDAGVSVKEEQSIWSDTADDGYGQALEMTVDTVG

SPDSIYFRDNTEAVFGQPLTGDEVIIQVNAIGVNFRDLLLVLGSLPWHAPGFDGAGVVVRAGPGVKDLHVGDRVFYTVPK

AGMTNYLRTSSSQVHGLPDGLDMTDAASMPLAYCTALLSIVDTARLQKGESILIHAATGALGQACIMLAQHLGAEVYATA

GTPEKREFLTSTFGIPPDHIFSSRTPEFRAGLLLATDNRGVDVIVNSLSGALLQQSWELIAPHGRFIELGKKDLLQNSHL

AMRPFLQNASFHALDVRKIEGARPGAVRGWLAEIVRLYQSGAIRPIQPVTQVPISQLAAGLRRLQKGHNIGKVVLTLGAD

EKVLAERFSPLNLPSTIASVRSTLLAPDATYIITGGTGGIGRALGEWMVKQGAKYIILLGRSGSSTPKMAELLKKYEGTN

VCMRAIACDVGYRGSLLQVKEEIKDMPPVRGVVHGALYLRTFLDAFSEHRIKQGKPAVSISLPVVEGVGYVADRGISERL

KGSLGLSLSEEHLYTLVKGAILGPASGLNVKGRSFSFVTNPMEADADLPWEHFHFLRAIAPPRRGGDMESGAGKGKALKS

AGPDAKRGVDTSPEAVMEALRTKISNVTMMDRDEITPERNLAHYGLDSLVSVELRNWIRREYGADLALKDIVAARHLNAL

SKDILAHIK

>Pensoli1|351480

MTINGTNGVNGHGANDYTVNNYLINGHSTNGVHTNATNGAGENGPSGVSASEVHYNGQIPIAICGMACRLPGGLATPDEL

WDFLLAKKDARCRVPESRYSIDSYYSDTKKPGTVSTEYGYFLNESVEIGALDMSFFTMARSEVERADPQQRLMLEVAREA

FEDAGVTQWRGKKIGTYIGNFGEDWLEMLGRETQPLAHAIRDGNPIQAVVRSTSHNVDGKTPTLSQPSTDVQEALMRRAY

EFGGITDFSKTAMVECHGTGTPTGDPIETRAVARVFGDKGVHIGSVKPNLGHTEAASGLVSLLKMVKALQYRTIPPNIKF

TTPNPHIPFEEGKLMVPTEATPWPEDRLERVSINSFGIGGANAHVILESAALSHCPPAIPRTSESPQLLLFSANTQNSLT

RQIDNYKTWVEQNPNKVSDLAYTLARKRTHLPFRAFAIVKNSVIDSVSQPTDTKSSTPPSVIMVFTGQGAQWPQMGRELL

QSNKVFKSSIRSLDRTLQAISVEKPHYSLEEELKKSGKKSRLSSAEFSQPVCTAIQIALVDALKAAGVAPKPVVGHSSGE

ISAAYAVGALTAAEAITVAHNRGAVTIKQERIGTMAAIGMGWGDAKKYLVPNVTIACDNSPKSVTISGDVDAVKSVIAAI

KKEEPQMLARQLQVDKAYHSYHMKEIGENYQSLISEVTGKAPSAPFFSSVTGKILDLDETTGSKYWRDNLESPVRFREAI

TAILKHDVGRNAIFLEVGPHGALAGPLRQIFAQNSSPTPYVSAMARNQDCTVSLLAAIGGLHSHNVQVDLEAIFPIGSCL

SSLPRYPWNHEGSYWYESRLSKEWRNRQFPHHDLLGARVAESSDNEPVWRNLFHVTNAPWISDHKVGDNIVFPFCGYIAL

AGEAMRQLNNSKEGFTIRNIHVTTALIINEGRPTEIMATFRPRRLTNSLNSTWWEFTVSAYNGRNWTKHCIGEASVLTSL

PEEAQDPVALPRKLNVRKWFEKMAKGGLNLGGSFQTLDTMSTSTSEQRATGHVVNGRQKDEANYFIHPTVLDSTLQILGA

AAVNGQARRTKTWLPTSIDKIVVYKCASDMTTNVSAKLSSNYSVVGDGSCTSEGRKVVEAVGIRMSLADGYGSGDAAEEH

AASRCEWQPDIDFLDVNKLFSAPASRTENARLLEELGDICLLLSQSHLSETPGKVALPHLEKHAAWIKLRAKSINMRLPC

TWTGLDKQAISARIDDLLRQLADTPAAPVASVIHQVCNNMDSLLSGKSLQEILVEGALTRVYDYLGQLERSEFIGKLIHS

KPNLRILELGTGRGEPLHPHILDTLTRPDGEILCAKYTLTTPGYLAAEAQEALFPNMDFKSLDINEDLLEQGFEESQYDL

VISVNALHEANNAQESLNNLKKLITPDGRIFLQELSPSSGWVDYVLGLLPTWRSSAENGSTQSSYMTQADLESKLAAAGF

GSPEAVVLDADASHNVTVTMVARLNCEAPIKKITVLFEEAGPSVTHILARLDKNGYAVSKCKLSDTPPAGQDVLSLLDID

KPFFHAIDEARFLSFKTFLLGLQERRSGMLWATHLIDIGCRDPRYAQVLGLARTIRTEQLADMAICQVDAFENPDSIDSL

LRVLAKFQLRRGDEELNPDYEWAIFNDRVQVARFHPFTLTDDLLVSEASNDMITLNVRTPGRVNSLYYARHERKELDKDE

VEVQVYSAGLNFRDILVALGIVELPVRLFGIEAAGIVTRVGADVSPDDLQVGDRVVCFCRKDAFSTYTTTLAAVCVRIPD

TLTFDQAGTMLIPYFTAIHSMVNVGRVTKGQTVLIHSACGGVGLAAIQIAQMLETELYVTVGSEDKVKYLMDKCGIPRNR

IFNSRDKSFVDGVMRETQGRGIDFVLNSLSGELLHATWSCVAEFGTLLEIGKRDLIGDGKLDMKPFLANRNYCCVDIDGL

WKRIHIARALIFSILDFYNKGHISPLPTTVFPVTQTQDAFRFMEKGQHIGRVGVSIKRTEKDTESQLGASKRALNVAFNG

SASYLMVGGLGGIGRAVSTWMVDHGARELVYMSRSAGLTNKDDDFIQELESVGCAVKLVSGDITKYEDVERAISAASLPL

RGLVQMSMVVANENFTKMSHVEWVTSTAPKVEGTWNLHNASTAAGLDLDFFLMFSSVSGIVGQAGQANYASGLPASVVDM

GAVEDVGWISEHQGMMGKMSRSGFKPVLEQEVIDAMTISMLVYNKPQQADKSALAVVSKNASYFVHKNTFLVGLALLIPL

NDPSNYVIWKKDRRMASYHNNSKIAATTASTDVLKSYLSNAKADPSVLKSAEAASLFAVEIGKKLLDLLLKPQEAVKTSL

PLADLGLDSLVALELRAWIKQVFSFDMPMLEMMSIGSLDILGSHIANEVFRIATAENES

>Podan3|210067

MADLRFEDQPVSQEGRNGRTNGTGIVNGDHHPTSERTTNGDSVNGHPHGNGLSTGSGLPNGSKTQTQPPVEPIAICGMGM

RLPGGITDAAGFWDMLYNGRSGRCEVPEDRYNAEGWYGPGKIGHTASKFGYFLDNVNLANMDSSFWSMTKKEIEAMDPQQ

RLTLEVVYECLQNAGQKPDQLRGKKVGVYLGTFEGDWLELDGRDPQHYHMYRLTGYGDYMSANRIHYEFGFMGPSVTIRT

ACSSSLTALHDACHAIFSGECESAIVACANVICSPRTTITMQEQGVMSPSALCKTFDADADGYARGEAVSAIYVKKLSDA

IRDGDPIRSVVRSTAVNAGGKSSTLTAPNTAAHEALIRRGHQLAGISDFSKTAMIECHGTGTAVGDPIETLAVANIFGDY

GIYIGSVKTNLGHSEGASGLSSLIKMTLALENETIPPNLNFTTPNPKIPFKECKLTVPTEPRPWPKDRDHVVGVNSFGIG

GSNAHVLLSSASSFGAANATAHKGDVEAFDADPGLRLLLFSAKHPKALQSMVSQHQAYHLSHPSQLRDMSFSLALKRDAL

SYRAFCVTDGLDDWTPVVSPRPASREPSKLVFVFSGQGAQWAQMGMALIKNVPEFRQSLRDMDKFLQTLPDGPQWNEIQA

PKSRSRISSAELSQPCCTAIQMALVDLLTSYNVTPGAVVGHSSGEIAAAYASGAITANEGIAIAYYRGKVMLSVDSTKKP

GGMAAVGLGRKEVEPYLSSGVLIGCENSPESTTLTGDKDALEGVMLNIKEANPDILVRALQVDRAYHSHHMRQVAPLYEE

LLSNMINANDPNIPFYSTVSCKTVKSGRELGPEYWVNNLVSPVRFSTAVSQILREPGRKTFVEIGPHSALAGPLRQILKS

AKSTDEYMNILTRGNNSHSDLLHAVGHLWSANQPLHLAPVVGEGKFLVDLPLYPWHYEEPMWYESRLAREWRHRKFPHHD

VLGSRILESTDSSPAWRNLLRLESVPWIKEHEVAGDIVFPGVGYVTMAGEAVRQLTGATDFTVRRVHIKAAMILVQETAT

ETITQLQRVPLTNSADSVWYNFTISSYQNGSWLKHAFGQVSAGSEFPHEAPDLAPLPRVVSRKAWYRKLRSLGLEYGPRF

LGLRDITANPVEPTLLTHMTNDIRDGESLYAIHPVTLDLVPQAIAPALTNGLTRRFDRVAIPTYIDEMYIGPPATPDMAM

EVRITEERKNARIADAVAVSDGKVVISVKGMQVSVISDAEGDGGQDPHAAVELEWKEDVNLMDITTLIRPAKDRHDVHKL

LDRFSALCMLDAAERLRVVEPSRPHLAHFRQWLDGLCVDILGGRYGCLSSQQSDVDMSPARRQETVDTLYAQLLDTEAHA

AATAVYRIASNCDSIFSGTTDELALLLEDNVLHQLYDFMQNSEYSAFLDLVAHRKPNLRVLEIGAGTGGTTATVLPALKS

AYGERMYLSYTYTDVSPGFFPAAKERFKEYEGLQYAVLDISKDPLEQGFDPESFDLIIACNVMHATPNIHETLSNVRKLV

HPRGRLFLQELSPETKWINFVMGVLPGWWLGGADLRFPEPYMDSARWDTELRAAGFAGAEAVAYDGYLNNNIITTPAAPQ

PPPSKRVTLLHSGETTTTTTIAVIASLRAQLQLSGYQIDLHTLDTPSLPENQDILVALDLTEPFFHNLTPRRLASFQELV

RQARDRGCGVLWLTGASQVGCVDPRFAPVVGVARVLRTETGLDFATLELDCLGQVDVEFGAVPAVLAEFQRRGPEEEDVR

SEAEWACVGGRVLIGRYHFVDVAKGITTAGDGEGGPAEKTVLKLEQHRPGLVNTLFWERRAETALGENDVRVEVQAVGLN

FKDVLVSLGIVAEPYSIGRGMGYECSGTVTAVGSSVTEHRVGDRVIASSSGSFTTSLQVSETLCVKMPDTLTFEEGASML

AVYCTAIYCLLDVGRLARGMSVLIHSATGGVGIAAIEVAKMIGAEIYCTVGNESKAEYIVDRFHIPRHHIFNSRDASFLP

ALLKETGGRGVDVVLNSLAGELLHASWKCVAKFGTFVEIGRRDLVGQGLLAMDVFEANRAFVGFDLLRFSTERPLMIKSL

MERALAFYTQGHLRPISPLTTLPATAISDAIRFMQKAQHMGKIVVTMPENRNELVSEATRNPIVLRSDGAYLLVGGLGGL

GRAITTWLAEKGARHFVFLSRSAASVSDHDPFVLELEALGCTTVRVSGDVSNYEDVLLAIKAAGRPIAGVLQASMVLRDN

SLVDMSWDDWVTASRPKIQGTWNLHNALAREQAEPLDLFFLFSSAGAMSGQWGQANYNAGNTFLDAFVQYRHSLGLPASV

LNIGVIGDVGYVSENTDVLDSLRATSQYIMEEPALLDCVELMLQRSAARATTPAAPGGDDAMYRYAQPSQMGIGMRSLLP

ITAPANRTVWRKDPRMLVYRNLEDTGGAGSGGGASSSDEELTRLLREISSNMTLLRSADTAALIAREIKNTLLGFMMRSE

DELDLDGPLASVGIDSLISIELRNWIRRRLGAEVTVLEIVRAPSLRELGATVQKKLVEKYEARVG

>Podans1|429115

MADLRFEDQPVSQEGRNGRTNGTGIVNGDHHPTSERTTNGDSVNGHPHGNGLSTGSGLPNGSKTQTQPPVEPIAICGMGM

RLPGGITDAAGFWDMLYNGRSGRCEVPEDRYNAEGWYGPGKIGHTASKFGYFLDNVNLANMDSSFWSMTKKEIEAMDPQQ

RLTLEVVYECLQNAGQKPDQLRGKKVGVYLGTFEGDWLELDGRDPQHYHMYRLTGYGDYMSANRIHYEFGFMGPSVTIRT

ACSSSLTALHDACHAIFSGECESAIVACANVICSPRTTITMQEQGVMSPSALCKTFDADADGYARGEAVSAIYVKKLSDA

IRDGDPIRSVVRSTAVNAGGKSSTLTAPNTAAHEALIRRGHQLAGISDFSKTAMIECHGTGTAVGDPIETLAVANIFGDY

GIYIGSVKTNLGHSEGASGLSSLIKMTLALENETIPPNLNFTTPNPKIPFKECKLTVPTEPRPWPKDRDHVVGVNSFGIG

GSNAHVLLSSASSFGAANATAHKGDVEAFDADPGLRLLLFSAKHPKALQSMVSQHQAYHLSHPSQLRDMSFSLALKRDAL

SYRAFCVTDGLDDWTPVVSPRPASREPSKLVFVFSGQGAQWAQMGMALIKNVPEFRQSLRDMDKFLQTLPDGPQWNEIQA

PKSRSRISSAELSQPCCTAIQMALVDLLTSYNVTPGAVVGHSSGEIAAAYASGAITANEGIAIAYYRGKVMLSVDSTKKP

GGMAAVGLGRKEVEPYLSSGVLIGCENSPESTTLTGDKDALEGVMLNIKEANPDILVRALQVDRAYHSHHMRQVAPLYEE

LLSNMINANDPNIPFYSTVSCKTVKSGRELGPEYWVNNLVSPVRFSTAVSQILREPGRKTFVEIGPHSALAGPLRQILKS

AKSTDEYMNILTRGNNSHSDLLHAVGHLWSANQPLHLAPVVGEGKFLVDLPLYPWHYEEPMWYESRLAREWRHRKFPHHD

VLGSRILESTDSSPAWRNLLRLESVPWIKEHEVAGDIVFPGVGYVTMAGEAVRQLTGATDFTVRRVHIKAAMILVQETAT

ETITQLQRVPLTNSADSVWYNFTISSYQNGSWLKHAFGQVSAGSEFPHEAPDLAPLPRVVSRKAWYRKLRSLGLEYGPRF

LGLRDITANPVEPTLLTHMTNDIRDGESLYAIHPVTLDLVPQAIAPALTNGLTRRFDRVAIPTYIDEMYIGPPATPDMAM

EVRITEERKNARIADAVAVSDGKVVISVKGMQVSVISDAEGDGGQDPHAAVELEWKEDVNLMDITTLIRPAKDRHDVHKL

LDRFSALCMLDAAERLRVVEPSRPHLAHFRQWLDGLCVDILGGRYGCLSSQQSDVDMSPARRQETVDTLYAQLLDTEAHA

AATAVYRIASNCDSIFSGTTDELALLLEDNVLHQLYDFMQNSEYSAFLDLVAHRKPNLRVLEIGAGTGGTTATVLPALKS

AYGERMYLSYTYTDVSPGFFPAAKERFKEYEGLQYAVLDISKDPLEQGFDPESFDLIIACNVMHATPNIHETLSNVRKLV

HPRGRLFLQELSPETKWINFVMGVLPGWWLGGADLRFPEPYMDSARWDTELRAAGFAGAEAVAYDGYLNNNIITTPAAPQ

PPPSKRVTLLHSGETTTTTTIAVIASLRAQLQLSGYQIDLHTLDTPSLPENQDILVALDLTEPFFHNLTPRRLASFQELV

RQARDRGCGVLWLTGASQVGCVDPRFAPVVGVARVLRTETGLDFATLELDCLGQVDVEFGAVPAVLAEFQRRGPEEEDVR

SEAEWACVGGRVLIGRYHFVDVAKGITTAGDGEGGPAEKTVLKLEQHRPGLVNTLFWERRAETALGENDVRVEVQAVGLN

FKDVLVSLGIVAEPYSIGRGMGYECSGTVTAVGSSVTEHRVGDRVIASSSGSFTTSLQVSETLCVKMPDTLTFEEGASML

AVYCTAIYCLLDVGRLARGMSVLIHSATGGVGIAAIEVAKMIGAEIYCTVGNESKAEYIVDRFHIPRHHIFNSRDASFLP

ALLKETGGRGVDVVLNSLAGELLHASWKCVAKFGTFVEIGRRDLVGQGLLAMDVFEANRAFVGFDLLRFSTERPLMIKSL

MERALAFYTQGHLRPISPLTTLPATAISDAIRFMQKAQHMGKIVVTMPENRNELVSEATRNPIVLRSDGAYLLVGGLGGL

GRAITTWLAEKGARHFVFLSRSAASVSDHDPFVLELEALGCTTVRVSGDVSNYEDVLLAIKAAGRPIAGVLQASMVLRDN

SLVDMSWDDWVTASRPKIQGTWNLHNALAREQAEPLDLFFLFSSAGAMSGQWGQANYNAGNTFLDAFVQYRHSLGLPASV

LNIGVIGDVGYVSENTDVLDSLRATSQYIMEEPALLDCVELMLQRSAARATTPAAPGGDDAMYRYAQPSQMGIGMRSLLP

ITAPANRTVWRKDPRMLVYRNLEDTGGAGSGGGASSSDEELTRLLREISSNMTLLRSADTAALIAREIKNTLLGFMMRSE

DELDLDGPLASVGIDSLISIELRNWIRRRLGAEVTVLEIVRAPSLRELGATVQKKLVEKYEARVG

>Cercau1|440480

MAYKLPISEPIAIVGSSCRFAGGATSPCKLWEILLNPTDLSRLVPKERFNIDGFYHKDGEYHGTTNAPKAYFLEQDHRVF

DATFFNITPKEAEAIDPQQRMLLECVYEALESAGYTLHQYAGKKVAVFAGLMTADYDTLSQRDDIYTSQYYATGNARSII

ANRVSYFFNFHGPSMTIDTACSSSLVALHQAVLSLRAGESEMACVTGANLIITPEQFIVESSLHMLSPTGHCRMWDANAD

GYARGEGVSAVFIKPLSRAIADGDRIEAIIRETGVNSDGRSKGITMPNWKAQANLIQDTYRQAALDPTAVEDRCQYFEAH

GTGTAAGDPNEARAIEHAFFGHGENTAPHPHPPALATKLVVGSVKTVIGHTEGAAGLAGLLKVVHAMNHDTVPPNLHLCN

LNPDVEQYCNSLIVPTKAIPWPQVPIGQPKRASVNSFGFGGTNSHAIVEQYVPSLHIEVAKRFHPGLEAPECHTFRECDH

EAHINLPLVISATSQASLAAMAKAYRDYLLRSQIPRLEYLAWHSLAHRTAFPFRTAVSSTSVGDLVSKLDALIEQSSAGA

SIGVRARPENEKPKILGIFTGQGAQWVTMSRGLLKSSKVYRDAIRALDAVLRSCPDPPTWSLEQEIEAEEGMSRVHIAKI

SQPLCTAVQIALIDLLQSLGITFHAVVGHSSGEIAAAYAAGKLCARDAILVSYYRGKFAHLAGGADGEKGGMLAAGLSRK

EAAELCARTEYRNSICMAASNAPSLVTLSGDIDVIHRVHDELKNANKFARILQVDTAYHSPHMDAPSKRYIEAVKACGVK

PLASSTGTSWISSVYGKGEPSDGEMTAAYWGANMVDPVLFCEAVESALDTLGPFDCAIEVGPHPSLKAPATQTIKAKIGN

TIPYAGLLDRTTDDRVAFSKFLGWMWTHFGSSSGLIRRFVLGSVQPDLVKVRLDDAPTYQWDHSQQYYRESRLSRRHHFR

THRPHELLGVRTRDDNDFELRWRNILKLDKLPWIEHHSFEGQALLPASAYLVMALDAARVALGSRAASLVELRDLTFASG

ISLDKNDTNGVEVLFSLIIDRASERTADSPIIDGSFTLTSAPADGTTSMKKNFEGKLRIFLGNPDSDALPVRPKRRAETL

NASPEAFYQMMAGLGLKYTGPFKGLTHLERRFAFASGAAQKLHSEDTTQLSISPATLDSCLQTAFVSVSSPGDGAIWTSF

LPERIEQIRFNLSICDIGCRDDTISVDTFLMQATPYTRRNRASFTADLELYNDNGNMEIQVQGLTVASWSPTKPEDDYEL

YLTSTFDVDPEDAIVTASIPDNHLPSPMLVESCDRVASYYYKDLPRHLSTRLLRSLQIGDHSVFPIPTKHWPAETEKSLD

DFIRASPYYYALDFIRQLGRGLPDVLPAVLPTAILEAHELAAYQQHISRVVGQIAHKYPRMNVLGLVRPEMGLSEHVLSG

LGDSFLSYVVGAEAEKNLEDRVFARSPHLRKKVQTEKVDFDADVFHSALAQRQDLVLVDTSLFEGRDKDHILRNIREMMR

PGGFLILLQNPLTSLRDRIRDFACCKEEQTKSITSEPGAWGRILRDCGFVSPIRNSEQEVYPGFSLMVRQSESAEKQLLA

HPRRHPAAIPHLTEKLLIIGGRQLWTSHIASRVQESLAPYCGTVTVIETLDHVEHDELTTCSAAILLSDIGDPVLSSLTK

VGMDHLRSLLRPEMTVLWITQNARFHNPDNAASFGFTRTLAAETPGLTLQMLDLDQVDPLPAANAISETFVRLAVRPFVG

SRTEASMLWVHEPEIHLEDGNRLVPRVVPWKEGIQRVNAGRRVVTNTVNTLENLLTPGMVFVHAHSSAREIKSWYPAGGA

CIFNFLPESSPVSHTLKRTHPRGCEYNQLTTPLDLSHDHGVEGNHFIESIWAEVVSTAVSKCLAWEPKSAVPTTTAARLM

AKLEPAHTFDILDWKADRWVEHVIQPVAGTALLDPRKTYVLVGITRDFGQSLCTLFIQQGARNIVLASRNPPSHPPVWQQ

ELLDEGYSVRFEPLDVTNLDQVLAFKSKLAETCPPVGGVVNGAMVLDDRVFSEMPLETFQRVMRPKTIGSKNLDTAFGSD

DLEFFIMTSSFAAIGGHAGQANYAAANMYMNGLAAARRARGKAGSVLNIGVIWGLGLLHREKEQLYQGLSREGYPPISER

DIHHMFIEAIAAGKPEPNQILNITTGLRRYPANHPTLFWQRDPRFSHFSFVDDEDMSGPSSGGEKKSLRELVVGADTKSE

IATIIQDAFIGSLQQLLQIPKGRLTGDDSIADIGVDSLVAVEIRGWFWKNVGQDVAVMKVLGVSSITALCQDVAASILDT

RITETKMAASLGLDGGPLSSQSSDSSSEGDLASEAPSGVTIPTPDAPGSPKQEA

>Cercau1|464568

MGMRLPGGVRNAESYWDLLVNGRDGRCEVPKDRYNIDTWFGPGRVTHVGTKYAHFLENLNYANIDASFWSFTKQEAELLD

PQQRLFLEVVYEAFESAGKTGWRGKDIGVYVGALGEDWSEMEAVDAQDLNPTRTYVYGDYIIANRASYEFDLKGPSMVVR

TACSSSLMALHLACQDIHSGDCSDGAIVGGINIIISPRTTVALTEQGVLSEDGRCKTFDAGADGYARGEGVSAIYIKKLS

DAIRDGDPIRAVIRSTCVAADGKTAGFTLPNDESHEKLMRRGHQLAGITDFSKTAMVECHGTGTSAGDPREVSAVARVFG

EHGIYIGSVKPNIGHGEGASGLSSIMKMTLALEHKTIPPNINFKTPNPKIPWKEAKLTVPTKPLPWPEDRLERVSVNSFG

IGGANAHVVLESAASWGVGRKTDTSASGEEDGVLKKQLLIFSAKHQKSLEGSAQKHEEYLTAHPESLADLAYTLNTKREV

HPVRAFTVTDGLDSFQLSRVVKSASGPEPSLIFTFTGQGAQWARMGRELITSEGVFKKTIDKLDAVLAELPDAPKWTLRD

EILAPKKTSRLAEAELSQPCCTAVQVALVDLLQSWGVKPSGVVGHSSGEIAAAYAAGSITAEDAIRIAYHRGQCTVALKE

THKGGMAAIGLGRESVEQYLKPGVAIGCENSPSSVTLTGELDVLETVMEDIRAAEPSALVRALRVECAYHSQHMKFVADS

YAARLGSIQAKTPQIPFYSSVTAKSNPDLSEGYWIQNLVSPVLFKSAVAAALEAHDNATFVEIGPHSALQGPIRQTIQAA

NKTAEYIGTLIRDVDALQAALNAAGNLWLAGVSRIDLHAVNGSPAGVKFLTNLPSYSWNYTELWLENRISKDFRLRKYDH

HELLGSRIIESSDAGPAWRCKLRVQDAPWLSDHDILGDIIFPGAGYLAMAGEAVKQLRPGTDDFSLRRVTLSSALVLHGD

PIELITTLVPVRLTTSIDSDWFTFTISSVNPTTNVWTKHITGQVRAGRDDAAVSETPEIEDFPRKVPTSSMYSVWRRFGL

NYGGRFRGLDDITAHTTEQRAAGTIYQKCSPDEAAVYSLHPGSIDAAFHLSNVCLCHGLGRNFRTPSVPKYIEEMYVGTP

DGPIRVVGDAEAKGRGGSNSNLTGVSNGKVVLNWKGLELSPLSDGSDVVDEDPHAAAVLEWKSDIDFMNPGSLLLSLNKD

INHPVRKLVDTLGLACIVESHHQLAGLETDAWHLIKFRDWLGLITQQAVEGNYSYVANCAEIAALSSPDRIKLIEDSLEA

SYGTEAEPVAVSLWRIYKNSADFFSGEEDPLEVLMADNLLMRMYDFTNNADSVAFLKLLGHKKPVMRVLEIGAGTGGTTA

TILAALASDQGERLYGTYVHTDISSGFAIGAKERFKDFEAIEYRTLDITLDPEEQGFELGSFDLICSSNCLHATPNLVHT

LKNVRKLLRPDGRLFLMELSPESSKSVNYVMGPLVGWWLSDDGRDHEPYVSADIWHERLLEAGFSGVEAWAFDGNTSNSI

VARPAVTLPPTLTSVSVVAQDAANPTVAAAIDHLRSNGLEVETFALGANLPADRPGVFLLDLEAPFLASITEEQFNAFKH

TLFSAPDMPVLYVTGACQIACADPRYALVNGISRSIRQETGLDFVTLELERFDTDGGWRGLSQILKTFPVRMAAARDQEE

RETDFESEYAFHDGTVQVGRMHWIKINDELKEKGDDVTTEQVKRLVIEKPGIIQTLKWRKAAAPVPRGGDWVRVDTRAVG

LNFKDVLIAMGIVDSGSEGNGDFGFEGAGVITSVGPDVKWLSVGDRVAFSSTGCFATSLIMAEMACVKLPNMLSFEGAAT

MPCVYGTALYGLEDLARLEEGQTVLIHSACGGVGQAAIQISQMIGAEIFCTVGNEEKIEYLGQTFGIPRDHIFNSRDSSF

LPGVLKATGGKGVDVVLNSLSGELLHESWKCVAKFGTMVEIGKRDFIGKAELSMDRFENNRTFVGLDHTELWAHKPKVAS

RVLRRVMLLCSEGKLAPITPIKTFESAKVEEAFRYMQKGQHIGKIVATVPKDTSDEVLNAVPARREIRLRGDRTYIFAGG

LGGLGQSIATYLAELGARHLIFFSRSAGRFAETNPSFGKELESLGCHVQFIGGSINELADVEKVIASAITPIGGVLQAAM

VLQDANFVDMTFQAWDTAVRPKVTGTWNFEEALKKQAEPLDFFFLFSSVSGTAGQIGQANYAAGNTFMDAYVQYRRAQGL

ACSTLAIGIMEDVGFLARERHLLEALRATSLHFLHEQDLLDAMELMLGPWAAAPLPPAQPAETGNRSIDIATRAYVNPGH

VILGLRSKLPLLSPMNRTGWKKNPRLLVYRNIEQHDTTTVSGGPAGTDGSLKEFLSSCGKTPEILTSETTANFLANEIGA

TLFNFMMRGDEEPDLNVPLANVGVDSLVSIELRNWFRQKVGVPFTVVEIVGASSIADLGRMTAEKLAEKHKRT

>Podcur1|343094

MAYKFPVSEPIAIVGSSCRFAGRVTSTTTLWQLLKSPSDLSRRIPAERFNIDGFFHPDAEYHGTTNAPNAYFLDQDHRVF

DANFFNITPKEAEAIDPQQRMLLECVYEALEAAGYTLGQYAGKNVAVFAGIMTADYDTLSQRDDIYTSQYYATGNARSIV

SNRISYFFNFAGPSMTIDTACSSSLVALHQAVLSLRSGESEMACVTGANLILTPEQFIAESNLHMLSPTGHCRMWDAAAD

GYARGEGIAAMFIKPLSKAIADGDRIEAIIRETGVNSDGRSKGITMPNWKAQANLIQDTYHRAGLDPRTPGDRCQYFEAH

GTGTAAGDPNEARAIEEAFFGRPDHRAGKIPKNTSHPAPVPMLLVGSVKTVIGHTEGAAGLAGIMKVVQALRHDTVPPNL

HLDKMNPVVEQYCTALFVPTQAMPWPRVADCQPKRASVNSFGFGGTNAHAIIEEFVPSLHVGVAKRFSPRLVIPQRPDSH

ECDHDARVNLPLLLSAASQGSLGAMAKTYRDYLDRGSPQRLEHLAWNSYAHRTALPFRVAVTGITVNALVSKLDKLVEQS

AGCVGTPLGTRARPDNEKPRILGIFTGQGAQWATMSRGLINSSKVYGDAIRNLDAVLRSCPDPPTWSLEQELNAEGSLSR

VHVAKISQPLCTAVQIALVDLLRSLGVDFHAVVGHSSGEIAAAYAAGRLCARDAMLISYYRGKFAHLAGGANGKKGGMLA

AGLSCEEAAELCARTEYGSSMCMAASNAPSLVTLSGDIDVIKRAHEELKAANKFSRLLQVDTAYHSPHMEVPSKQYLEKL

AACQIAPLPDGNGTTWISTVYGHGEPPASDIALSYWNANMLQPVFFREALESALDTAGPFSGAIEIGPHPSLQAPATQTI

KAKIGECFPYSGVLDRTTDDRVAFSQFLGWMWTHFGSATDHIRRFVQGSVQPDLVNLRLEGTPSYTWDHSHHYWRESRVS

RQYHFRTHAPHELLGVRARDDNDFEMRWRNILKLETIPWIEHHMFEGQSLLPASAYIVMAVDAARVALAGRPASVIELRD

LAFLGGITLDKNDTQGVEVLFSLAIDRASDSVFDQSTIDGSFALTSAIPGDTSAMKRNFEGKFTISLGDPYPDILPNRPA

ERPETLRASPKAFYEMMAGRGLGYTGPFQGLTSLDRRFCFASGTVKKLHSEDTTKLKMSPATLDCYLQTAFVSLSSPGDG

AIWTSFLPEAIDAIRVNLAICDSQTGDEISVVDTYLTKATPYTQRQKASFTADVQVYDNDGNMEIQVQGLTVASWSPTKP

EEDYELYLTSVFDLDPEDAIVAVKTPEDPLPNSILIESCERVASYYHKTNPPHSSRLLRRLEAVKYSQHPVPASHWPAET

DQSLDDFIRRSPYYSALDFIRQLGRKLPDLLPAVLPTVVIETHELIAYQHHVSRVVRQIAHKYPRMNVVGLTRAETGLSE

HVLAGLGDSFSSYVIGGESEKNMEDHVLGALPNKRSKIIINELNFDGNLSYSVPMTDIDLVLLDVSFLEGRDIEVALQKI

RDIMRLGGFLILLQIPRTSLRDRLRGFAGMGRDQEAAHTQSLSEWTEILQRSGFLTSIANSQQEVRPGFNILVSQANSIE

KQLLNHPHRYPEEIPHFTDRLLIIGSKEKWVSQIAARVQDCLSPQCGTVAVVDGLDDAEHVQLLSCTAVVFLSDICEPIL

SNLTKIGLEHLQLLLRPHMTLLWVTHNARYSNPDNAASFGFTRTVAAETPGIALQMLDLDRLDILPAADIISETFLRLAV

RSLVHGSSDSPILWVHEPEVYLEDSRRHVPRIKPWKEGIERVNAGRRVVASLVNTLETCVAISSYEQNNGDNGHQSIVQE

IRLQDLPELEKSRVLQVDYSTAEAIRFSSTHPKVHVCLGREVSTQSCIITLSSTNSSYISQDCTFTAEVPCSVQSQPLFL

MLVVGYVAAFSIDTARRSRPILFLDPDPALEECVRDVFCRRGIAFRIFCTDRTRCKRTPGMVFIHSNLTVREIRSWCPAG

ASIFNFLPGTHPVSQTLAKTPPRNCEFLTMESLFEFVVRDRPGINDLLQSIWTEAVETAISKSLSLASSCHAVTLSPAQL

LNRTEPAKIFEIIDWRADRWMQHIIQPCSTPKVLDSRKTYVLVGLTRDLGQSLCTLFVNEGARNIVLASRNPPVPAPLWQ

QELLNQGYNICFEPLDVTDLQQVRDFKEKLAQTCPPVGGVVNGAMVLDDRVFSQMSLETFHRVMGPKTIGSKNLDITFGS

EDLDFFIMMSSFAAVGGHAGQANYAAANMYMNGLAATRRSQGKAASVLNIGVIYGLGLLHREKENLYEGLSREGYPPISE

RDVHHMFMEAIAAGKPAPNQILDIITGLRRFPANHPTLSWHRDPRFSHFSYDEDVDDMESISGSDKKTLKELVVAAGTRE

EIVTVILSGLTSQLQRLLHIPAGRITGDDNIAELGVDSLVAVEIRAWFWKSVGQDVPVMKVMGVSSIFKLAQDIAAGILE

SRNVSMQAAESMIMAGGLIESLNSVTSSEDGVASGTSSAVTSTVPTPDAPRSPKVVT

>Podcur1|446740

MAVPETNDNSNSNSNSNGQAAGVNGHSNGANGHSNGANGHSDSSRNGTFEPIAIIGMGMRLPGGVRNAESFWDLLVNGRD

GRCEVPKNRYNIDTWYGPGRVTHVGTKHANFLEDLNYANIDASFWSFTKQEAELLDPQQRLFLEVVYEAFESAGKTGWRG

KDIGVYVGALGEDWSEMEALDAQDLNPTRTYVYGDYIIANRASYEFDLKGPSMMVRTACSSSLMALHLACQDIHSGDARD

GAIVGGINIIISPRTTVALTEQGVLSEDGRCKTFDAAADGYARGEGVSAIYIKKLSDAIRDGDPIRSVIRSTCIAADGKT

AGFTLPNDESHEKLMRRGHELAGITDFSKTAMVECHGTGTAAGDPREVSAVARVFGEHGVYIGSVKPNIGHGEGASGLSS

IMKMTLALEHKTIPPNINFKTPNPKIPWEEAKLTVPTKPLPWPIDRLERVSVNSFGIGGANAHVVLESAASWGVGKKAQA

PESDIDGVLKKQLLVFSAKHQKSLEAAAQRHEEYLTVHPDSLADMAYTLNNKREVYPIRAFTVTDGLDSFQLSRVVRAGT

STSPNLVFVFTGQGAQWARMGRELLGSEGIFKQTIEALDATLSSLPDSPQWTLREEILKPKKTSRLAEAELSQPCCTAIQ

VALVDLLRSWGVAPTAVVGHSSGEIAAAYSSGAISAEDAIRIAYYRGQCTLALKESYKGGMAAIGLGRESVKPYLRPGVI

VGCENSSSSVTLTGDLDVLETVMEDIRAGEPSALVRALRVECAYHSHHMQAVAKSYAARLGAVEAKTPAVPFYSSVTADS

TPELSTQYWVQNLVSPVLFSSAVDAALQAHDNVTFVELGPHSALQGPIRQTIQASQKTAEYMGTLVRDTDAYQAVLSTAG

NLWLSGYAGLDLHAANGSPDNVKFLTNLPNYSWNYTELWLENRISRDFRLRQHDHHELLGSRVLESADAGPAWRCKLRVQ

DAQWLSDHDILGDIIFPGAGYLAMAGEAVKQLRPGTDDFTLRRVTLSAALVLHGDPVELITTLTPVRLTTSIDSDWFTFT

ISSVHPTTNVWTKHVSGQVRAGRDDAMVPNVPGDIGDDLPRKVPTSSMYSVWRRFGLNYGESFRGLDNISAHTTEKRAAG

TIYQRCTSEEAAVYSLHPGSIDAAFHLSNVSLCHGLGRNFRTPSVPKYIDEMYVGTPDGPIRVVGNASPIGRGGSTSNIV

GVCNGKVVLSWKALELSPLSDGSDVVDDDPHAAAILEWKADIDFVDDMKSLLLSLNKDMNDPIRKLVDTMGLVCIVESHH

QLAGVQTDAWHLDRFRDWLGMITQQAVEGRYSYVENCEEIPAMSSADRIKLIEDNLAASYGTEAEPVAISLWREADSLDV

LMQDNILMKMYDFTNNADAVRFLKLLGHKKPVMKVLEIGAGTGGTTATILAALVSDQGERLYGTYVHTDISSGFAIGAKE

RFKDYEAIEYRTLDITLDPEEQGFELGSFDLICSSNCLHATPNLIHTLKNVRKLLRPDGRLFLMELSPEASKSVNYVMGP

LVGWWLSDDGRDHEPYVSADVWNQRLLEAGFSGVEAWAFDGNTSNSIVARPAISTPPSLESISIVTQNASHPTVASAIEH

LKSTGMGIDIYAPGAGLPIDRPAVFLLDLEAPFLASVTEAQFNAFKHTLFSAPDVPSLWVTGACQIACVNPNYALINGIS

RSVRQETGLDFITLELERFDAEGWRGLSRLLATFPARIAVTKAGEERETDFESEYAFHSSSMQVGRMHWIKINDELKEKS

EVSGEQVKRLVIEKPGIIQTLKWRMAPARVQQSGDWVTVEPRAVGLNFKDVLVAMGIVDSGSEGYGDFGFEGAGVVTSIG

PDVKTLSLGDRVAFSSTGCFATSLTMAEIACAKLPEGLGFDEAATMPCVYGTALYGLENLARLVEGQTVLIHSACGGVGQ

AAIQIAKMIGAEVYCTVGNEEKIEYLVKTFAIPRDHIFNSRDSSFLPGIFKATEGKGVDVVLNSLSGELLHHSWQCVAKF

GTMVEIGKRDFIGKAELAMDRFENNRTFVGLDHTELWAHKPKTAGRVLQRVMQLCSEGKLGPITPIKTFEAFKVEEAFRY

MQKGQHIGKIVVTVPEGAVDLLNAAPGRREIHLRGDRTYIFAGGLGGLGQSIATYLVQLGARHLIFFSRSASKFAEESPW

FGKELESMGCHVQFIGGSINEFADVEKVVASAITPIGGVLQAAMVLKDMNFVDMTYEAWETSVLPKVTGTWNFEEALKKQ

AEPLDFFFLFSSVSGTAGQIGQANYAAGNTFMDAYVQYRRSQGLACSTLAIGIMEDVGFLARERHLLEALRATSLHFLHE

QDLLDAMELMLGPWASAPAASPAESSTDTHTANKGYVNPGHVILGLRSKLPLLSPMNRTGWKKNPRLLVYRNIEQHDSTS

STEGGASGTDGSLKEFLTSCGRTPEMLTADATIDFLAGEIGATLFNFMMRGDEEPDLNVPLAQVGVDSLVSIELRNWFRQ

KVGVPFTVVEIVGAGSIKDLGKMTAEKLAEKYKRT

>Acral2|1052158

MHTNGTGSSSYAAPQSNGEKQPALEPIAICGIGLRLPGGVTDVPSFWDMLVNGKSGRCPVPKDRYNVDAWYHPDKDGHVP

SRYGYFLDHVDIRNLDTSFWSMTKREVEILDPQQRIALEVVYETLQSAGQKSSDLNGRKVGVFACNFGGDREELDARDTQ

TRHPYDLTSTFDFVLANRISYEFNLVGPSVTVHTACSSSLTGLHEACQALYSGECESAIVLGSSIIYAPTVTVAFRGHGV

LSPSGLVKTFSADADGFARGEAVLAVYVKKLSDAVRDGDTIRSVVLSTAIGADGKSSTMTAPNPAEQAELIRRTHALAGN

ADFSKTAMIECHGTGTAVGDPLEAASVASVFGPHGGVWIGSVKSNIGHTEGAAGLAGVVKMTLALENGIIPPNLNFSKPN

PKIPWEEAQLKVPVAPTPWPEGKDKVGATNGSSAPVPRLLVFSAKHQSALERMVQNHQSYCLANGDRLPDVAYTLAMKRD

TLSHRAFCVANGIDDWVPFYAPRSGHYEPGKLVFVFSGQGAQWATMGKALIKSVKTFRDSIEEMDNVLKQLSDGPEWDLQ

GQLLAPKKTSRVSEAEVSQPCCTAVQVALVDLLREHGVEAQAVVGHSSGEIAAAYASGAVTRKEAIIIAYYRGKVLRGMD

SSKGGMAAVGLGADQVQPYLKPGVLVGCENSPDSVTLTGDKQVLDEVLAKIKEDDPAMLARALQVDRAYHSHHMQAIAQD

YLDLIAPHLQARKPTIPFVSSVTNKDLAQGCELAASYWVDNLVSPVKFSQALAGSVHAFPGSSKVFLEVGPHSTLAGPVR

QILKAENSKDDYVSVLTRGKDSHEEVLRALGQLYLLSQPVDFAPLVAGGKLLTDLPLYPWHYGEPMWKESRLAREYRLRE

FIHHELLGARIVESTPFSPAWRNVMRLEDATWIKDHEIEGDIVVPGVSYLLMAGEAARQITGNADFTCEKVYMKNALVLN

GDDEKEVITQLNLVKSSSSSSSSGSTDAAWYEFSISSYDNDSNSWTEHAHGRVRGGGDKETHLKSKTPEPEGYARKCSSK

AWYRKFRSLGLEYGARFTPLYDVSADPLSDRLAASVKIGLRPGEEQYYSIFPGSLDGIPQALFSASSRGLTRNFNRLAII

QSVDEFYMRPPSPETEELRLQCEITQKKPNCMIGDVVAVSAQGGGGDAVVRSRGWQLSYIAEAVGGDGGGDDESGNPHGA

AELEWKDDIDFVDAASFIRQTGITSEGREPQPLDRLSLLCYAEIQDRLRDAPPPTREFLGRFRKWIDDRIDEVLSKGFAW

FGVPDARDLLGLDSEERNRLIEDIYARLADSPSYGPATALYRVARNCRGIFDGSISELEILLADGALQKVYDFLLEYADL

SGFLSLMAHKRPNLRVLEIGAGTGGATSTILPNLRSAHGETAYLSYTYTDISAGFFNDAKERFKDYTGIEFAVLDISKDP

LEQGFAAGSFDLVIAYNVLHATENLHRTLSHVRTLMHPEGRLLLQELAPRTMWAGVFGVLAGWWYGHEDGRTEAPYVEID

RWTEELTGAGFENVTSMHDGYMNNNIVCQPQRDFEPVKRVSLLRHESQAVPESVVDSLAAAGYQVDDITLEADPSAELRP

GQDIVAVLDLASPFFVSLDEGKLTRLQRFLTAAKEASCGILWLTGSCQVGHVSSPDFAPILGAARVWRNDMQVEMATLEL

EDFDAAVKVLPSVLGQFQQRTADEDIEPEHEWAHVGGRTLISRYSYLKVRQELESHLPADAVVRKVAQRKPGVINSSSLF

WKAQKPQPLQTGQVSVAVKAVGVNSRDVSIAQNNSASADAIEGLGVDCAGIVTQVGPGVDQFKVGDRVGCLALGSLATSN

NVAQDLCFSIPDGKSLEDAAQIPFPYCTAVHALVDRAHLGEGMSVLISSAAEDVGTAAVQVARMTGAEVFCTVGGEEQID

YVTTKLGVPRDHVFTADEFSVPDMVALTKGRGVDVVFNSSSGGLAILFSKCLAEFGTFIDTSDRESRGQDRPAVQSRSNQ

TYIGIDLAQLTIHRPQMVGDVLQRVLGSWKEGRIGPTVPAQEYSALQISEAFRDAHLSEYTGKLVLSMPTADEFPAEAVD

DPLQFSGDRSYLFVGGLGGLGSVISTWLAERGAGEVVFLSRSAGSSPEHVQFAQELATLGCEVKLVAGDVARYEDVVKAV

MAATRPFGGVLQASLVLRDASFSNMGYEDWVAASAPKVQGTWNLHNAILEKQPDTPLDFFLLFSSTAATGGWYGQANYHA

GNTFVEAFANYRQQMGLAASVLNVGFIRDAGFVAENAAAAEAARSMGQWFNTETELLECIELLLKRPPRGGQPAEPEDSS

SANNGLGPVQRHVQRSLLAMGMRSTTPVASSRMPWRKDRRMLAYRNFEAHQLKSSAVAGSGSSSNDELTQFIRDSRSNIS

VLQSPETPVYLGTEMGKALFDLIMRGDEQVNVEAPLASIGLDSLVSLELKSWIRRWMGVEVATLEILNCATLHGLGAVVQ

GKLLEKHQS

>Aspbom1|6365

MHAREVSDNETSSSHSNIVPTPSETSSRCSQQPSIPIPEGDKEPLVEPMAICGMAMRLPGGIHDAEGFWDLLYNKRSGRC

RVPKDRYNVENWYGPGKIGHVASEYGYFLDDVDLRNADASFWSMTKQEIEAMDPQQRLSLEVTYECLQNAGQRPQELRGR

KIGVYLGTFEGDWLELDGRDPQHYHMYRLTGYGDYMSANRIHYEFGLTGPSVTIRTACSSSLTGIYDACHAIAAGDCESA

IVACANIIYSPRTSITMQEQGVISPSGFCKTFDANADGYARGEAVSAVYIKKLSDAISGGDPICSVIRSTCINAGGKAST

LTAPNTTAHEALIRRGHELAGITDFSRTAMIECHGTGTAVGDPIETAAVANVFGKHGIYIGSVKTNLGHSEGASGLSSII

KMTLALERQTIPPNLNFTTPNPKIPFERCKLKVPTEPLPWPKDRAELVGVNSFGIGGSNAHVLLGSAASFGIESTQKKIA

ACEEAENLMADLTPHLLMFSAKHQQSLKRMITDHQAYFLSHPESLQDMAYSLAHKRDDLSHRSFCVTNGEDDWVQSRTHR

ISGRAPPMLVFTFTGQGAQWPQMGKSLIDQVPQFRHSIEKLDKVLQALSDPPQWKLIDEIRKPKKICQLSKAEFSQPCCT

AIQIALVDLLNHYGVHPDAVVGHSSGEISAAYASHAISAADAIQIAFYRGRVMCSLNPVERPGGMAAVGLGAEEVAPYLR

PGVLVGCENSPNSITLTGDKAPLDEALKAIKEANPESFVRALQVDRAYHSHHMETVAPQYVDLLSTQGVKAMNPSAQFFS

SVTGRQVTQSQDLGPVYWARNLVSPVRFLTAISELMQSLIGPKVFLEVGPHSALAGPIRQILQMHKSTDEYFNTLTRGSD

SHKDLLNAVGEMWLQNMPVDLSAISERGCFLPDLPLYPWHYEEPLWSESRLAKEWRLREFPHHDLLGSRVLESTDQNPSW

RNILRLDVVPWIKEHEVASDIVFPGVGYICMAGEAIRQLTRDSAFTARRVHIKAALVMHQGQDVEVITQLQRIPFTNTVE

SKWYNFTVHSYNKGVWVKHIFGQVSAGSDREHRIPSLEPLPRQLSRRGWYRKMKEMGLEYGSRFMGLTEMTAHPIERKTI

ATVVNDNREGESQYAVHPVSLDCLLQAIVPATFNGLTRRFQHLGIPTYMEEIYVCPPLQSEMVIEAHADEQPTAALSGNI

IAVANGQVTIDIKGLQMSAIGDAAYASGQNPHAAVELEWREDINLITDAHRLIRPAKDRREVHHSLDRFASACIIDTVTR

LQGVEPSRPHLCHYQKWIASTAGQIRLGKYPGLQRSDEISQSSESRRLEIIEGLYPILLRTEACAAAKAIYRIWKECQGI

FTGEVEELGLLLEDEVLHSLYDFMQNSEYKVFLELLAHRKPNLRVLEIGAGTGGTTATVLPALQSLYGERMYHSYTYTDI

SAGFFPAAKKRFESYPEMKFATLDISEDPLAQGFEAESFDLVIACNVLHATPVLQESLANVRKLLHPQGRLFLQELSPNT

KWINYVMGVLPGWWLGEQDDRYPEPYISIDRWESLLNKTGFFGVELVSHDGYLNNNIISRRVQEKERPKHITLLHSVKSP

SSAISTTNQILYSAGFEVDLLVIEDTTTLPPTGQDIVSILDLGGPFFHHLEQPEFENLKTLLSHLQETESGILWVTGACQ

VGCKDPRYAMVNGVARVIRTEMNLDFATLELESFEKDTLALMPKVLGEFQRRLSEQNINTTTEWAVVGQSPLISRYHYIK

VDEELKNKAAEEKPSVKKLVQTKAGLVDTLCWQDMPVSHGLDPDDVLVQVKAVGMNFKDVLISTGVITEKSSIGRGLGYE

GSGLVLEVGSAVDKLSPGDRVIMSSSGSLTTIQKLDQRLCVKMPDFLSYEEGATMSAVYCTAIHCLLDVGGLRKGKSVLI

HSASGGVGMAALYIARMVGAEIYATVGSEKKAQALMSSFNIPRNRIFNSRTKEFLPRLMEETNGVGVDVVLNSLSGELLH

ASWKCTAQFGTFVEIGRSDFVGQGLLDMQPFEPNRSFVGFDLLLFSEKRPERIESIMTRAMDYCSAGFIHPITPTTTFEA

VSIVDAIRHMQRGQHIGKIVITMPEISTDLPAEPSRQELSLRHDRAYLFVGGLGGLGRSIATWLVEHGARHLVFLSRSAG

SIPVDDPFVQELAVLGCKSYMVSGDVSKHEDVVRAIRASGKPVGGVLQSSMVLRDNSLLEMKWDEWQAAVQPKVQGTWNL

HNALLSEQPEEILDFFLLFSSAGAMSGQWGQANYNAGNTFLDAFVSYRHSLGLPASTVNIGVIQDIGYVSQNPEILDSLR

STAQYLMREPELLESIELMLHRSSPTHSVADQASNIYVTRSQIGIGMRSTLPIEAPSNRTIWRKDPRMLVYRNLEVQSGP

VASSTGSGQALTQFLREIGSNMTMLKAPESAEMLAGEIGKTLFGFLMRAETEEVDLDAPLASVGIDSLISIELRNWIRRN

IGVEITVLEIVRADSVRALGLLAQKKLVEKYEARM

>Aspcal1|767632

MHTVNIDDSNLPLYTGTAPSNPNSFSNNATTSSFPPIAICGMATRLPGGVHTPSALWDLLTTKRSGRCRVPASRYTVSSF

LGPGKLGHVASEHGYFLDDVDLRDVDTSFWSGMTRKELEAMDPAQRLALEVVYECLQSAGQKARELREKDVGVFVGTFGG

DWSDLDSRDPQRYHMYRMTGQGDYMLANRVSYEFGFGGPSVTTRTACSSSLTALHSACQSLIAGDCSSAVVASANLILSP

APCIIMQEQGIISPSGSCKSFDAGADGYARGEAVSAIYVKSLADAIRDGDPVRSVIRSSCVNSACALASTPTSGSGAEGK

SITTPSALAQEVLIRRGLELAGVGDLKSVAMIECHGTGTQVGDPIETTAVGNVFGEHGIYIGSVKSNLGHCEGASGLTSI

IKMTLALEKKIIPPNINFSTPNPKIPWQRFKLKVPTEPIPWPADRAELVGVNSFGIGGSNAHVLLASAASSGLTPSAASK

ETATSTSSLTPHLLLFSATHPSSVSRSISAHQAYALSHPDSLADMAYSLALKREVLSHRAFCVTDGEDNWAPSRTRRTRA

TAGQVAAAPTLVFVFTGQGAQWPRMGRELIKTVARFRERIEELDRVLESLEDPPGWRLLDEILAPKRKSRLSTAEFSQPC

TTALQIALVDLLSSYGIHPAAVIGHSSGEIAAAYAARAITAADAIKIAYYRGKVLATPSPSRTGASAGSEPEPASREGGM

AAVGLGVEQITPYLKPGVRVGCENSPESTTLTGEKKVLEEVVGAIESENEDVFVRWLSVDRAYHSHLMDPVAPAYVDLLT

KARVCTANIGMDPSIDFFSSVTGCLHDKTKLLDPAYWAQNLVSPVLFSTAMTSLCQTHPGPKAFLELGPHSALSAPIRQI

LNHHQAPSASASAPAPADEYIPTLIRNNNSHRDLLTALGELWLRNTPIDLDAIFNTAKTKAEFLPDLPLYPWHYTREENA

EKLWSETRLAHEWRFRQFPHHELLGVRVLESTEDNPSWRNVLRVESVPWVKDHVVGGQVVFPAAGFVGMLGEAMRQVTGG

SLEGGFTVRRLGIRAALVLSTEAEGDEGVEMVTQLMRTDASPGPAGDQTGYTFTIHSYQPKPNGKKGTWIKHVVGRVSSG

PGNLPALPPAAPSPSALPRALSRRAWYRKMREMGLEYGPRFMGLNDMSAHPTEKRLVATVVNEIPEAGKQKPGESVYAVH

PASLDCLIQAIIPATFNGLTRRFRSLGLPTYIDEIYVCPPSDSSMTIEARIDQHKTTGTTTTDISRTITATANNQLAVRI

SGLQLSTTSITQSSDPFPYGPHAAVELEWREDINLVRDFGFLFRPARIEDAADERRRAEVYALLDRFGAACIFDTARRLD

GVEVSPAKPGLVHFKRWVVRAKAMMESGAYPGLRAEDIGALTQAQDVGGTERTQRTTLIEDLYNSLLNPPTPASAPATAL

YRIHQSALSLLSGEASALDLLSTDNTFHAVNELVQSHADYSAFLSLLAHRKPNLRILEIGGGTGATTRRVLDALRAASTY

AERMYYSYTWTDLSPGFVAAAKRTFAGVPGMEFAVLDIERDPLGQGQGQGFEAGSFDLVIACNVLHATTSLHTSLTNIRP

LLHPEGRLFIQELSPATNAQWINYIMGVLPGWWSGENDGRYPEPYVSVERWDGLLRAARFGGVMGSGFDGYSCNCIVAGL

ARDREREGRRVTLLHSGGAVSDAAEGMGRILVEAGVAVDYYALDAVLPPRSGQDVVSVLDLEAPFFHDVDEASFENLKRL

LSHLHQQQSTDGNNNSGILWLTGASQVACKDPRYGMVNGVARVIRTEMNIDFATVELEDFAAETLAHVPAIMEEFTRRTS

IDKNGIRANMEWAVAGGKALIGRYHFIRVEDELERTGQRNESRDIVKNVQQTSPGRLDTLCWREKPASLSSSQALLGETK

VLVQVRAVGLNSTDLLMTMVTNPLLTDASSTTHPPGHEGTGLILATGSAVHDLAVGDRVMVTGPGCLTTTLQLDQRLCVR

MPDSLTYEDGATMALAYCTAIHCLLDIGHLRKGMTVLIHAEGGGSSIAIAAVDVARMMGAEIFAAVCSDEETEFLMQTFD

IPRHRIFPSRTTAFYAGIMEKTNGVGVDVVLSPDSASGQLLHSSWNCTAEFGTFVDISQGQSRGQSQGLLGMNPSDANRT

FVHFDILRMIEKQPQRIESLMARTIEYYRAGLVHPVTPTTFPAEALVDAFRHMQTSEQHQHMARTVITIPKDTTLLHAEP

SRRHLVLRPDRAYLFVGGLGGLGRSVSTWLAEHGARHLVFLSPSAGTVPDDDSLLVELAVAGCTTARVSGSVSNIQDVHT

AIQAAGIPIAGVLNACMVLADTSFADMTLPTWQKATDPKVRGTWNLHSALQTLQPEEALDFFLLFSSISAAGGQWGQANY

NAGNTFLDAFVSYRHSLGLPAAAVNVGVMQDVGYLARNENAELLEALKATAQWLNTEGELLDVLELAMLRSWPGQSGTGS

HVSTGVSESQGSMPTSATATATAAGTASDKSGTRCSYVNHSQICMGMRSTLPMDAPGNRTVWRGDPRMLVYMNVENHEQS

LSGSSTSTDSEQVLAQFLREASSDLAVLRASKTAEVLAAAIGRTLAGFSGSGQSTTDEPRAKAGQPAEIDIDIDASLSTS

GIDSLVSMELRNWIRRKIGVEISVLEIVRAECVRELGVVAQRRLVDKYEACL

>Aspcar1|307147

MHTVNIDDSMLPLYTGAASATSKPNPSDSSNSSNSNSNALFPPIAICGMATRLPGGIHTPSALWDLLSTKRSGRCRVPAS

RYNVSSFVGAGKLGHVASEYGYFLDDIDLRDFDTAFWSGMTRKELEAMDPGQRLALEVVYECLQSAGVKGDEVRGKNVGV

FVGTFGGDWGDLDSRDPQGYHMYRMTGQGDYMLANRVSYEFGFGGPSVTTRTACSSSLTALHAACQSLIAGDCTSAVVAS

ANLILSPAPCIIMQEQGIISPSGSCRSFDAAADGYARGEAVSAIYVKKLVDAVRDGDPVRSVIRSSCVNSASASASKSVS

RSGVEARSITTPGAVAQEALIRRGLELAGVTDLKSVAMVECHGTGTQVGDPIEATAVGNVFGEHGVYIGSVKSNLGHSEG

ASGLTSLIKMTLALEKRVIPPNISFSTPNPQIPWVRFKLKVPTEPIPWPADRAELVGVNSFGIGGSNAHVLLASAASFNL

NQPPPYKETASASASATPHLLLFSAQHPTSVSRTISNHEAYALSNPDSLPSMSYSLALRREILSHRAFCVTDGEDSWTPS

RTRRVPPGKAAPTLVFVFTGQGAQWPRMGRDLIKTVPRFKEGIEELDRVLNTLPDPPGWKLLDEILAPKRKSRLAAAEFS

QPCTTALQIALIDLLSSYGVRPAAVVGHSSGEIAAAYAAQAITAADAIKIAYYRGKALSTPNLLDSSDETLPAGGMAAVG

LGVEQVTPYLKPGVRVGCENSPGSTTLTGDKEVLEEALRTIESETEVIVRWLRVDRAYHSRMLGLILSQTAVPALDRDTD

YVCMTADHMHPFAPAYIDLLTTAGIREAHPTTSVDFFSSVTGRLVDETHRLDPAFWAQNLVSPVLFSTAMTSIRGTHPGP

KIFLELGPHSTLSAPIRQILNGNHDTAASPVPTDEYIATLTRNNNSHRDLLAALGELWLRNIPMDLPALFTPSQTQTQIQ

KQTHPPRFLPDLPFYPWHYATEANENEEKLWSETRLSREWRFRAYAHHELLGVRVLESTDENPAWRNVLRLDSVPWVKDH

VVAGRVVFPAAGFVGMAGEASRQVSDAHRGSGEGVGFTVRKLRISAALVLPVEAEGDEGVEIITQLVRVDSDSVSGSASG

PAGDPTWYSFTIHSYQPKPNGKKGTWLKHVTGQVSATPSAAAPPAVPSPSPLPLPRPLSRRAWYRKMREMGLEYGPRFMG

LTDMSAHPIEKRLVATVVNDIPEAGKRRPGDSVYAVHPASLDCLIQAIIPASFNGLTRRFQSLGLPTYIDEISIYPPSNR

TMTIEARIDETPGTAISRTITATSNNNLAIRISGLQLSSTAIAASRDPFPYGPHAAVELTWREDLNLVPKIGSLFRAARI

ENAADERRRAEVYALLDRFGAACVFDTARRIEHIGVSSARPDLVHFKKWVVRAREMMMSGEYPGLEAADIVTLTCTTQDS

DTSTDQNGDALIEDLYSSLLKPPTPASAPATAIYRIHQLSLSLLSGETSALDLLSADNTFHAVNELVQSHADYTAFLALL

AHRRPNLRILEIGGGTGATTRRVLDALQAVSGSGSGSTSASKTHAERMYYSYTWTDLSPAFVAAARRTFAGVPGMQFAVL

DIERDPLSQGQGNMFEAGSFDLVIACNVLHATSSLHTSLSNIRPLLHAQGRLFIQELSPATNARWINYIMGVLPGWWSGE

NDGRYPEPYIDVDRWDGLLRQVGFGGVSGYAFDGYSCNCIVAGLAGGIEGCIEGEGRRVTLLHSRGARSDAVQGVRRLLS

EAGVVVDEYALGSADAPPAGQDVVSVLDLERPFFHDVDESSFENLKRLLSHLHLQATDDTSNTNNRGILWLTGASQIACK

DPRYGLVNGVARVIRTEMTIDFATVELEDFDADTMAHVPRILGQFLDRISIDKRGDRADMEWAIVHGKVLLGRYHFIRVD

EELERTAHRDEGRDVVKKVQQTSPGRIETLSWSEMPASPSPSQSLGETEVLVQVRAVGLNPTDLLTTGANPLVTNAPSTT

PNLGLEATGSILATGSSVHNLAVGDRVMVTSPAGCLMTTLKVDHRLCVRMPDTLAYEEGATMALAYATAIHCLLDIARLQ

KGMSMLIHCANDAGGVAAVDVARMIGAEIFATTDTEEETQHLMQTFDIPRHRVFSSRATDLHARVMDATDGEGVDVVLSS

GSEELLHASWKCTAEFGTLVDVTRSQSQGVLDLQLSQANRTFVRFDIRRLIEKQPQRIESLMARTVDYYRAGLIHPITPA

VFPAGSLVDAFRHLQNSEQQKQHLTKTVIAIPEDTTLLRASPSRRHLILRPDRAYLFVGGLGGLGRSVSTWLAEHGARHL

IFLSPSAGTVRDDDPLLVELAAIGCKTTRVPGSVTNIADVHTAITAAGIPIAGVLNASMALADTSFTEMTFATWTKAVAP

KVRGTWNLHDALISLQPEQKLDFFFLFSSISAAGGQWGQANYNAGNTFLDAFVGYRRGLGLCAAAVNVGVMRDVGYLSQS

GNAELLEALRATAQYLNTEGELLDVLELAMLRSIPEQSGGAGCGGGSISQDGLGLHSMTTSGSQNTRYSYTNHSQICMGM

RSTLPMDAPGNRTVWRGDPRMLVYRNVEDHRDPSSSASSTGTGSEQVLAQFLREASADLALLKASKTAVVLANAIGRTLA

GFSGSGSGSGSTASDPATDDARGTANQEAIDIDASLSSSGIDSLVSMELRNWIRRKIGVEISVLEIVRADCVRELGVVAQ

RRLVEKYEGCL

>Aspnom1|160492

MEQCCSNVAFPEAAEVKDPNSVRRPTPVWGNRDRVTEYVVDNVREDLVPFPIAIVGMGMRLPGGVSSGTEFWNFLVNKRD

GLCRVPETRYNVDAFYDEAREGAVRTKHGYFLEQDIAQLDIGFFEISKPEAEKLDPQQRLLLEVVWECMENAGQTNWQGT

NIGCFVGVFGEDWLDLLSKDTQQHDRYRVMSAGDFALSNRVSYEYDLTGPSVTVRTGCSSSMVGLHEACQAIYTGECSSA

IVAGTSLIMSPTMTTTMSENLVISSSGLCRTFDAAADGYGRGEAINAVFVKPLADALANADPIRAIIRSTAVNCDGKTPS

ITTPDPKAQERLIRRAYKKAHIEGDDILKTAFFECHGTGTIAGDTAETTGVANIFGEKGIYIGAVKPNVGHSEGASGITS

IIKCVLALEKKTIPPNVHFHKPNPKIPFEDAKLQVPVEPTPWPSDRKERISVNSFGIGGTNAHVILDSASSVLRKTSPEA

RLASEPDYHLLVLSAKEKRSLDGQIERITRYIEASPSCLNDLAFTLARRRYHLPYRAFAVADKDGSLPTFQKAQSTAPCP

VFVFSGQGAQWPTMGVELMCRFPKFREDIHRMDKILTELREAPPWSIEEELSKDEATSRVAHAEFAQPLCTAIQIALVNL

LREWGIVPSAVVGHSSGEIAAAYASGAISDRVAIIISYLRGQAIKALSMSRSGAMAAVGISPDTAHAFLEEGVTIACENS

PISVTLSGDKQTLDRVLDRIHNADENVMCRRLAVDVAYHSHHMLRSRHIYESLISPHIYHNSSMLPLYSSVTSTPIIEPT

KLDAAYWSQNLSSTVLFRTAVQGSLDDNGPAQLFLEIGPHSTLAAPLRQIFQAQSSKEKSPLYVPTIRRGTGEWQSLLAT

GGQMFMHGIPVDFGAIISNGVALSDLPPYSWQHGERYWNESRLASHWRLRHEPHHELLGSRVLESSSVEPSWRNILQIDQ

VPWLGDHRIGRDVVFPCAGYVAMVGEAIRQITESDEYSVRNMFMRAALTLEASIATEIITTLRPARLADNIDSVWYDFTI

LAYQNGTWKKHCIGQVRPASDKTFKAKEIIPYPRLVRSERWYNALEKRGLEYGPQFRGLEHISASPSSYQAAATLRDEEC

LYTSEYVLHPIIIDESLQLLSVAASHGIPRQMTRLAIPTAIEELYIGSGRGTMSLDVSCDTSGGMMRGNAMLVTDGQVTL

NLYHGLFFSIQEPDIGNPKLPVAATPHWGPHIDFFPVEKLFSPSQSLLDGHRKTFRIVSLYGVEYYHQMRRSQPTQEHLR

KWQSWVSSNYEYMRDNAPMLVPELRDICSLSPAERAAEFETFKSMQPRDFGYPFYVLCKRILDSLHKLLEGQLEPIDLLV

EDGVLNSFYERSARIGSWHEFASLLGHSYPQLRVLEVGGGTGGDTLVALQGLTLDHSNRLYSTYTFTDISLGFLLEAREK

YKSYPAMEYATLDISRDPVEQGFEPESYELIIAANVIHATPRISDALRNVRRLLVPGGRLLLIELTCTIPIIDYIMGILP

GWWLGDGDGRKEQPYIPVDEWHDKLLDAGFTGVETFRYDDEPPYQLNARILSRVPAAKFPLKGEVSLLYHTVINDWARDL

ANALVEAGYSVHWCTLKQMPSPHSNIISVIDLEGPFFHELTPADFESFQSYVSHLVDGHLLWVTRSVQPECDDPRYALVL

GLARTIRHEVMLHFATIEIDQVEKTALQPVVHVFERLLSQRDGPGASPEYEFAVREGSVQVPRFRWNSFEGQVEASEIQA

PRVLDIESYAMLDSLTWTCADITPNDLQKEEIELDIKYVGLNFRDLMTVMGFMGDVAHVGLEASGIVRRVGSAVSRFSPG

DKIMVSQSGLMSTRKVVRVERCIPVPDNVSLEGAATILCVYATVVYSLINVGGLKPGQSVLIHSACGGVGLGAVQLCQLM

GAVIYATVGSEEKARHLIDNLNIPADHIFDSRSSSFLQGVLQKTEGRGVDLVLNSLSGDLLHASWQCVAKFGKMLELGKR

DFLGHGLLEMDRFLDNRSFVGIDLLQVLDGDIEVLHEMIGSVMEYFHQGKAGPISPVTVFDAADVAKAFRYMQSGQHMGK

IVVKMPDDPSTLPVARVHERVSYFPAHASYLLVGGLGGLGRAVATWMVEKGARHLVFLSRTAGNTIDGSSFIKTLECQGC

DAITVVGNVGNIDDVRRAVSAAKTPLAGVIQLSMVLKDQSFHNMVHEEWVAALFPKVKGTWNLHHVLKDKPLDFFLLLSS

MAGIMGWPGQANYGAANTFLDAFVKYRQSLNLPAHVIDLGLMGDIGYASEASLISALQASQSKSLQVLDERQFLQAVEAA

VLAQRLSRQNQVVVGLGTTRTLSSAEFASAWTKEARFDIWKNIIATMEQPVEGSKADELREHMEAIKNNPLLLDRPETEE

KILLELGKLVASYTSRPEDMTLEEFSNIPIDSLMTIEIRTWFRRHAGIDITLVEVSNSGTVGGLGKIAVQKLRDKHTHKG

HKGSEGQGGDPDGAEEPGYHDDLTLGKTMRPISNASPYWTSESEGNVFFTGATGFLGAFLLSELISLPQVKQIACLVRAS

TSDLGHTRIRQTFSKYGLPVDFKSKIIAIPGDVTKKNLGLRPETFSHLAQWSSVIFHFTGYANYTLPYSVHRGPNVLGLL

EILRFANTERLKPVHYCSSISACGITENLTGPVPEDVRPRVEAHNVTQSIGYTQSKFVAESIAWDAIENGFPIAIYRPTV

VTGDSRTGACKKEDMVNRFMANCIRLGCYPNPPQQRFHFVPVDFACSAISRISLNSTSLGHAFNVTQPDQDKVITLSEVY

KILSNYSPTPLISIPTVQFFKRFTKEPDSQTEVTSYILAERLPAHQIWWDDWEYMAAYGTENLRQAIADHPDVTELRPVP

ELLKVYYDFWSTLG

>Aspnom13137_1|534

MEQCCRNVAFPEAAEVKDPNSVRRPTPVWGNRDRVTEYVVDNVREDLVPFPIAIVGMGMRLPGGVSSGTEFWNFLVNKRD

GLCRVPETRYNVDAFYDEAREGAVWTKHGYFLEQDIAQLDIGFFGISKPEAEKLDPQQRLLLEVVWECMENAGQTNWQGT

NIGCFVGVFGEDWLDLLSKDTQQHDRYRVMSAGDFALSNRVSYEYDLTGPSVTVRTGCSSSMVGLHEACQAIYTGECSSA

IVAGTNLIMSPTMTTTMSENLVLSSSGLCRTFDAAADGYGRGEAINAVFVKPLADALANADPIRAIIRSTAVNCDGKTPS

ITTPDPKAQERLIRRAYKKAHIEGDDILKTAFFECHGTGTIAGDTAETTGVANIFGEKGIYIGAVKPNVGHSEGASGITS

IIKCVLALEKNIVPPNVHFHTPNPKIPFEDAKLQVPVEPTPWPSDRKERISVNSFGIGGTNAHVILDSASSVLRKTSPEA

RLASEPDYHLLVLSAKEKRSLDGQIERITRYIEASPSCLNDLAFTLARRRYHLPYRAFAVADKDGSLPTFQKAQSTAPCP

VFVFTGQGAQWPTMGMELMCRFPKFREDIHRMDKILTELREAPPWSIEEELSKDEAISRVGHAEFAQPLCTAIQIALVNL

LREWGIVPSAVVGHSSGEIAAAYASGAISDRVAIILSYLRGQAIKALSMSRSGAMAAVGISPDTAHAFLEEGVTIACENS

PISVTLSGDKEALDRVLDRIHNADGNVVCRRLAVDIAYHSHHMLRSRHIYESLISPHICHNSSMLPLYSSVTSTPIIEPT

KLDAAYWSHNLSSTVLFRTAVQGILDDNGLAQLFLEIGPHSTLAGPLRQIFQAQPNKAKSPLYVPTLRRGTGEWQSLLVT

GGQMFMYGIPVDLGAIITNGVALSDLPPYSWKHGERYWNESRLASHWRLRHEPHHELLGSRVLESSSVEPSWRNVLQIYQ

VPWLGDHRIGRDVVFPCAGYVAMVGEAIRQITESDEYSVKNMFMRAALTLEASIATEIITTLRPARLADNIDSVWYDFTI

SAYQNGTWKKHCIGQVRPASDKTFKAKEIIPYPRLVRSEKWYNALEKRGLEYGPQFRGLEHISASPSSYQAAATLQDEDC

LYTSHYVLHPIIIDESLQLLSVAATHGIPRQMTRLAIPTAIEELYIGSGRGKMSLDVSCDTSGGMMRGNALLVTDGQVIL

NLYHGLFFSIQEPDIGNPKPPIAATLHWGPHIDFFPVEKLFSPSESPSGGLCKTFRLVSLYGVEYYHRMRYSQPTQEHLR

KWQSWVSSNYEYMRANAPMLVPELRDICSLSPADRAAEFETFKFMQPRDLGYPFYLLCKRILDSLHKLLEGQLEPIDLLV

EDGVLNSFYEQSARIGSWHEFASLLGHSYPQLRVLEVGGGTGGDTLVALQGLTLDHSNRLYSTYTFTDISPGFLLEAKEK

YKSYPGMEYATLDISRDPVEQGFEPESYDLIIAANVIHATPRISDALRNIRTLLVPGGRLLLIELTCTIPIIDYVMGILP

GWWLGDGDGREERPYIPVEEWHDKLLDAGFTGVETFRYDDEPPYHMNARILSRVPPTKFPRKGEVSLLYHTVINDWGRDL

AEALVEAGYSVHWCTLKQMPSPHSNIISVIDLEGPFFHELTPADFESFQSYVSHLIDGHLLWVTRSVQPECDDPRYALVL

GLARTIRHEIMLHFATVEIDQVERSALQPVVHVFERLLSQLDDPGASPEYEFAVREGSIQVPRFRWNSFEGQVEASEIQA

PRVLDIESYAMLDSLIWTCADITPNDLQKEEIEIDIKYVGLNFRVRPDDRDGLHGGCGPCRLEASGIVRRVGSAVSRFSP

GDKIMVSQSGLMSTRKVVRVERCIPIPDNVSLEGAATILCVYATVVYSLINFGGLKPGQSVLIHSACGGVGLGAVQLCQL

MGAVIYATVGSEEKARHLIDNFNIPADHIFDSRSSSFLQGVLQKTEGRGVDLVLNSLSGDLLHASWQCVAKFGKMLELGK

RDFLGHGLLEMDRFLDNRSFVGIDLLQVLDGDIEVLHEMIGSVMEYFHQGKAGPISPVTVFDAADVAKAFRYMQSGQHMG

KIVVKMPDDPSTLPVARVHERVSYFPAHASYLLVGGFGGLGRAVATWMVEKGARHLVFLSRTAGNTIDGSSFVKTLECQG

CDAITVVGNVGNIDDVRRAVSAAKTPLAGVLQLSMVLKDQSFHNMVHEEWVAALYPKVKGTWNLHHVLKDKPLDFFLLLS

SMAGIMGWPGQANYGAANTFLDAFVKYRQSLNLPAHVIDLGLMGDIGYASEASLISALQASQSKSLQVLDERQFLQAVEV

AVLAQRLSRQNQVVVGLGTTRTLSSAEFASAWTKEARFDIWKNIIATMEQPVEGSRADELREHMEAIKNNPSLLDRPETE

EKILLELGKLVASYTSRPEDMTLEEFSNIPIDSLMTVEIRTWFRRHAGIDITLVEVSNSGTVGGLGKIAVQKLRDKHMHK

GHKGSGGQDGDSNGAEEEPRFHDDLTLGKTMRPISNASPYWTSESEGNVFFTGATGFLGAFLLSELLSLPQVKQIACLVR

ASTSDLGHLRIRQTFSKYGLPVDFRSKIIAIPGDVTKKNLGLRPETFSHLAQWSSVIFHFTGYANYTLPYSVHRGPNVLG

LLEILRFANTERLKPVHYCSSISACGITENLTGPVPEDVRPRVEAHNVTQSIGYTQSKFVAESIVWDAIENGFPIAIYRP

TIVTGDSRTGACKKQDMVNRFMANCIRLGCYPNPPQQRFHFIPVDFACSAISRISLNSTSLGHAFNITQPDQDKVITVGE

VYKILSNYSPTPLISIPTAQFFKRFTKEPNSQTEVTSYILAERLPAHQIWWDDWEYMAAYGTENLCQAIADHPDIIELRP

VPELLKVYYDFWSTLG

>Beaba1|4374

MSHANGHQSSFRPNTGNDALPTPLSDKPILMSPASSQASPPPGDTVQEPIAEPMAICGMGMRLPGGIRDAEGYWDLLYNK

RSGRCKVPASRYNVDAWYGPGKIGHVASKYGYFLQDVNLANMDASFWSMTRQEIEAMDPQQRLTLEVTYECLQNAGQIPE

QLRGKKVGVYLGTFEGDWQELDGRDPLHYHMYRLTGYGDYMSANRIHYEFGFMGPSVTIRTACSSSLTGLYDACRAISSG

ECESAVVACANIIYSPRTSVTMQEQGVISPTGICKTFDAKADGYARGEAVSAIYVKRLSDAIRDGDPVRSVIRSTCINAG

GKATTLTAPSTAAHEMLIRRGHELAGIHDFSKTAMIECHGTGTTVGDPIEAAAVANVFGEHGIYIGSVKPNLGHSEGASG

LSSIIKMTLALENKTIPPNIHFTTPNPKIRFDECKLKVPTEPLPWPQDRDELVGVNSFGIGGSNAHVLLGSAESFGIFQT

HPKPIDAADQALDELTPQLLLFSAKHPMALSRTISEHEAYNISHPDSLQDMCYSLALKREKLIHRAFVVTDGEDAWQPSR

VRQASVKAAPPKLVFVFNGQGAQWPQMAKKLVQQSNLFRQRLENMQAILQSLPDGPAWSLIDTLLAPKASSRLTEAEISQ

PCCTAVQVALVDLLGHYGVRPDAVVGHSSGEIAAAYASGAISTEAAMRIAYYRGLVMPLLDATSRLGGMAAIGLKPQDIK

PLLQSGVVVGCENSPSNTTITGDKDALDKTMDSIRAAYPGVLVRKLHVDRAYHSPHMQVIAPTYLRLLERQLISASAPAV

PFYSSVTGGQVTEAGTLGAAYWVQNLTCPVLFSTALGQAHSSSAALQTFLELGPHPALVGPVRETLGQFSSSFDHVKTLS

RGQDSYRDMLHCIGEMWLRQVPVDVAVAAGQGHFLTNLPLYPWHYDDGDDEQLWFESRLSKEWRLRQFPHHDVLGSRVLE

STNHSPSWRNIIRLDAVPWIKEHEVAGSIVFPGVGYVCMAGEAVRQLTGSESFTVRRVHIKSALVMHQGVDVEVLTELHR

APLTYALDSKWYSFSISSLHNGTWTKHSFGQISAGADKAHTAPKICPKARSLSSRGWYRKMRAMGLEYGPRFMGLKDMSA

HPVERTVVASLTNDMRDGESQYAVHPVTLDCFIQAIAPATFHGLTRRFNTLGLPTYIEEMYVAPPLHKDMQIEVRADKEP

KATLSGDITALSGGQVAIEMKGLQMSAIEDSSDLQDPNRHAAVELEWKEDLHLQASVEHLIEPASDRTAVYALLNEFAAV

CMQRTSDKMHTLAPCAAGVAHLQHYKTWLHDATQHASRKQLGLGATTLRSIDELYRELQSSEAKAAAEAMYRIHDDCASL

FSGQVDGLELLLRDDVLHQLYDFMQNSKYDALLDLAAHRKPNMRVLEIGAGTGGTTATVLPALRSRYGERMYSSYTYTDI

SAGFFPAARKRFEAYSAVEFAVLDIAKDPIEQGYEPNSFDLVIACNVLHATPSLRATLENVHTLLHPEGRLFLQELSPAT

KWINFIMGILPGWWLGEQDGRFPEPYVSAERWDQELLSAGFSGAALRSYDGFLNNNIVSTPCRVLDEAKSRTRLTVLYSA

SRPSPAQTVVDILTGHGYTTGLQAIESIEEANLPESGDVLSLLDLSEPFFHELDAVKFSNLQKLLAQVQNVGGGILWVTG

AAQVRCVDPRYAMVNGFARVMRTEMSLDFATLELDRFDYDGLAVVPRVLREFQLRLSEDAVNPTLEWAYDRGKMLISRYH

YIQVEEELKMEGTAQGDQARTSKLVQDRSGLIDTLCWRQELQAPLLDECDVLVQVKAVGLNFKDVLISTGVISAKSSIGR

GFGYEGSGIVVAVGANVAKLTPGDRVIMSSSGCLATTMSLDQRLCVKMPDNMTFLEGATMSAVNCTAIYGLLDSGKLTKG

STVLIHSAAGGVGIAAMQIAKMVGATIYATVSSEEKIDYLVKAFDQPRERIFHSRTGDFLQHVMAATDGVGVDVVLNSLS

GELLHASWKCVAEFGTFVEIGRRDFVGQAVLDMQLFEPNRRFIGFDLLLFATKRPHVIEGIMERVMSFCNLGFIQPIRPM

AIFPAHSVKEAFRFMQRAQHIGKIVLEMPEEHNQPSLCSSISPQPLALQPDGAYLFVGGLGGLGRAVATWLVEYGARHLV

FLSRSAESSADAADFREELNQLSCHATLVAGDVAKLQDVERAIQAAKMPIVGILQASMVLRDTNVSEMTWHEWQAAVQPK

VAGTWNLHKALIRCQPSQTLDFFFMFSSAGAISGQWGQSNYNAGNTFLDAFVSYRHSLKLPASVVNIGVIEDVGYVSENP

DILDSLRSTSQHLMQESELLESIELMLRRSSHRFSSHQQLGPPYVDRSRIGVGMRSTLPIMSPNNRTIWRKDPRMLVYRN

LEKAADAQTADDGDGDDQTLSNFLRGASTNVMVLKAPETALLLAGEIAKTLFGFLMKTSIDDACLDAPLATIGIDSLISI

ELRNWLRRKVGVEFAVLEIFQAPNIRALGVVAQGKLVDKFQALL

>Calor1|3749

MGNNADGPVPNGHVNGHRTNGDVPTNGESSAGTYEPIAIVGMGMRLPGGVHNGAAYWDLLVNGKCGRCRVPKDRYNVDTW

YGPGRVTHVGTEYAHFLEELNLANIDPTFWSLTKQEAELLDPQQRLFLEVVYEAFENAGLTNWRGKNIGMYVGALGDDWG

EMEMQDGQDLNPTRAYVYGDYIISNRASYEFDLKGPSLVVRTACSSSLVALHLACQDVLAGECTGAVVGGINLIITPRTT

VALTEQGVLAPDGLCKTFDADANGYGRGEGVSAIYIKKLSDAIRDGDPIRAVIRSTCVAADGKSQGFTMPNPESHEKLMR

RGHHLAGIYDFSKTAMVECHGTGTSVGDPREVGAVANVWGDHGIYIGSVKPNIGHGEGASGLSSIMKMVLALENKTIPPN

INFKNPNPKIPWEKAKLKVPTKATPWPADRLERVAVNSFGIGGSNAHVILESAASFGVGQKNVDSVPENGSSPLRAHLLT

FSAKHSQALEKSVQKHEDYLTTKPDSLNDLAYSLNVKREAHSNRGFVVTNGLDSFELSRISKASSTSPNVVFTFTGQGAQ

WARMGRDLIEYEPVFKDSIHTLDETLSKVAGHSAWKLTDELLASPKTSRLSEAEFSQPCCTAIQIALVDLLRSWGVSPNA

VVGHSSGEIAAAYACGAISAQDAILIAFHRGQATLPLKSSHNGGMAAIGLSRESVEQHLQPGVIIGCENSPSSVTLTGDS

EVLDKVMGDIRSAHPDTLVRALRVECAYHSDHMKVVASDYLASLGDIKAAKPQVPFYSSVTGEISTNLSAPYWTKNLTSP

VLYRSAVQTLLADYPSTSNLVFVEVGPHAALAGPTRQTLQGEGRSVEHIATLVRNAGGFDSILRTIGNLWLAGASVDLAT

VNPPGTFLTDLPTYSWHYDGDYWVENRMSKDWRLRPYDHHELLGARVLETSDAGLAWRCKLRIEDAPWLRDHDILGDIIF

PGAGYLCMAGEAVKQLHPGTPDYTLRRVTLSSALVLHDQPVELVTTLVPVRLTTTLNSEWYDFSISSFNGDAWIKHVSGQ

VRPGPELVQEVPEIETLPRKVANTSMYNVWKKYGLNYGGRFRGLSDISSHTTEQKAAGTIYDKCTPEESALYSVHPAAID

AAFHLSNVCVCRGLSRNFKVPSVPKYIEEMYVGNPKGAIRVMGDATEKGRGGSNSNLVGISNDKVVLSWKGLELSPLSDG

SEVVDDDPHAAAIVDWRTDVDSIDARRLLHSLNKDMADKEHRLVDKMGLTCIIESRAQVAGLKTSQWHLEKFRAWMDIPY

TEAVEGRYPNVPDCAEIAKMSSEERVKIIKDSLDASVGTVAHAVAIAIYQIFDNCVKFFTGEADPLEVLLTDNILMRMYD

FANNADHVQFLTLLSHKKSTLRVLEIGAGTGGTTATVIPALISEQGERMYSKYVYSDISAGFFKAAKERFKDYEAMEFST

LDVTQDPVPQGFEEGYFDLIVASNVLHATPNLVTTLTTVRKLLSPQGRLFLLELSPESSKSVNYVMGPLVGWWLSEDGRE

YEPYVSHEVWHEKLLQTGFTGVDAYAFDGNMSNSIIAKRVPAYDDKFTKLSVVYGDSKNPEVAEAIKFLQNRGLDLEFFA

AGQTLPAGQPAVFLLDLEKPFLVDVTAEQFKAFKESLFSIHDTSLLWVTGACQVEAKNPDYALVNGMSRSIRQETGIDFV

TLELESFDESAWTALYDLLKTFPARLRPGDPDTDLDSEYVFNAGTLQIGRMHWIKVTEELKDLEKKDRGKRLVIEKPGII

QSLHWKDSAPTPGNSDWVQVDTRAVGLNFKASDVLIAMGIVEAGNGSLGVGDFGFEGAGVVSSVGPDVKGLQVGERVAFS

STGCFSTSLSMPEIACAKIPDSLTFEEAATMPCVYGTAMYGLLNVARLEEGQTVLIHSACGGIGQAAIQLAKMVGAEIFC

TVGSEEKVKYLVDVYDIPQNHIFNSRDSTFLPGILKETNGKGVDVVLNSLSGELLHASWKCVATFGTMVEIGKRDFIGHA

ELEMDRFERNRTFVGLDHTELCAHRPRVANKLLTQMMEWCQEGKLRPITPLKTFEAAKVEEAFRYMQKGQHIGKIVVTFP

QEQQSLTADPARRETVLRRDRTYLFAGGLGGLGQSITTYLVEKGARQFIFFSRSATKFAESNPEFFKELESLGCEAQAIS

GSINNMRDVERAIAAAKTPIAGVLHASMVLQDANFVDMEFEQWQTAALPKVQGTWHLHHALSKQEESLDFFFLFSSVSGT

AGQIGQANYAAGNTFMDAFVQYRRSLGLPASTLDIGIMEDVGFLAREKHLLDALKATSLHMLHEQDLLDSLEMMIARDSE

FKKNPTSKETESPRLIDGYISPGHVIIGMRSKLPLLSPLNRTGWKKSPRLLVYRNIEFQDHTETGTSTDGGLKEFLQNCS

NTPAMLASDDAVTFLAHATGSTLFSFMMREDEELDVTIPLAHIGVDSLVSIELRNWFRQKVGVQLSVLEIGEAASLLELA

KTTTEKLMEKFKH

>Clagr3|2367

MHDRSGGPETTTNRVELNGHANGYTNSTFNGSENGSSNGLDAGSNGTVNGNAHQSHSYSTKSRILNGNTAESLQSPNGHR

IDPANLNGNSNITPDHVVEPVAIVGMAMRLPGGIHNAEDFWDLLINKRNGRCRVPKDRYNIDAWYGPGKSGHVGTQHGYF

LEDLNLSHIDASFWSLTKQEAELMDPQQRLILEVVYEALENAGEKNWRGKNIGCYVGIFGEDWLDMDSKDVQNMHMYRLT

GYGDYITANRVSYEFDFKGPSMTIRTACSSSLTGLHEACQALYNGDCTSAIIGGTNIIMTPRMTIAMTEQGVISPTGSCK

SFDASADGYARGEAVSALYIKKLSDAISDGDPIRAVIRSTCVNNDGKTVGLTNPSTEAHETLIRRGHELAGIKDLSKTAM

IECHGTGTKIGDPIETKAVANVFGQYGIYIGSVKPNLGHSEGASGISSVIKMVLALEHKTIPPNINFTKGNPQIPWDEAK

LKVPVTATPWPEDRAERVGVNSFGIGGANAHVVLESAASYGYDRSFERVEAISERPNLLVFSAKHPESLRRSAQNHASYL

GSHSDTFNDMSYTLSTKRQPLPYRAFCIASEGDSFELSRINKPGEPPHLIFTFTGQGAQWARMGKELFIQEALFSNSIKA

LDKVLSSLPEAPQWTLQDEILKSKSQSRLTEAEFSQPCCTAIQIALVDLLKAWSVKPAAVVGHSSGEIGAAYASGVLTAP

EAILVAYYRGLATLGLGEIGRGGMAAIGLGRDQVTPYLRTGVIIGCENSSSSTTLTGDRDTLEQVMLDIQTGNPEVLVRA

LKVECAYHSHHMKAVEAKYRALLGKSIHAKEPIVPFYSTVTGKLLRSGEILSTSYWVQNLVSPVLFLSAVSAITDSLPSP

KAFLEIGPHSALAGPIRQIIRNEAKEAHYIPTLVRNEDAMTALLKTAGELWISNLDLNFGAINPAGELLTDLPTYPWNYD

GEYWYESRLSKEWRQRKFPHHDILGARVTESSEVDPTWRNILRLDNVPWIQDHEIAHDILFPGAGYIAMVGEAIRQLTDL

RDYTVRAVNFMSALMLHEGKPVEIFTHVRKVRLTTTLDSEWYEFSIISLNGPTTTKHCVGQVRGGSDSRIEAPAIEPLQR

MVPSSTWYRVMARFGLNYGPRFRGLKEISAHVSERKAVATLTDGLTEKETPYQIHPTTIDCSFQLFSVSAFHGIGRLFNK

LSVPTYIEELYIRPAKDNILIQAEAASSFSGALSGNLVGISAGEVVINLVGLKLSPLGDNDQGQNEDPHAAVELEWKSDL

NLLDAAQLMRPAKDITKCHMLVEKLALACMIESHFQFMDLQPSLPHLLDFRTWLEIQHKKAIGSQYPNIQDCAVIAGMKH

SQRVALIRDLHTEALNTDAAAVATAIHRIFAHSSNIFLGKVDPLEILLENEVLTKVYDFMQLWEYGEFFELLGHYKPDMK

ILEIGAGTGGTTSTILPHLKSSYGERLYGSYKYTDISAGFFVAAQERFKDIQGMDYGILDISQDPIQQGYEAESFDLIVA

CNVLHATPKLNETLSNVRKLLHPRGRLLLQELSPSTKWINYVMGVLPGWWLGADNNRAIEPYIAPQRWAQELKSSGFEGI

DAIAYDGQLNNNIIAMPARDPRTKQLTVLCKRESEKHVQELSKLLRERSYKLDYCTLEQTPKPGQDIISLLDIETPFLYS

ATAKEFEAFKDFVTRIEGSGVLWVTGAAQIRCHNPNYSLILGMARTIRSELLMDFGTLELENFDLDGWKATANVLHEFGR

RVRDSDHDPVLEYAYSDGKVQVGKYHWISVSKELLDFKHDSHPRKLEIGRPGILHTLAWKQEEPVDLKSDWIEVETRAVG

LNFKDVLMSMGIVDLVGRGLGCERSGIVRKIGPQVKHLKVGDRVLCCTDGSFSTTLSTSELFCAKMSDNLSFIEAATIPC

VYGTVIYGLIDIARLSKGQTVLIHSACGGVGISAIQIAKMIGAEIYTTVGNQEKVDFLMQNFGIPRNRIFNSRNTSFLPD

VLRETKGRGVDVVLNSLSGELLHASWKCVADFGTMVEIGKRDFIGHGTLSMNLFEQNRTFAGLDLSQICADRPAVIHDLL

RRAMEYYEQGYIKPIAPIKEFQAVEVEPAMRYMQKGQHIGKIVVTFPEDATELEATTKTRDLVLRPDVSYLFIGGLGGLG

RSIASWLVEKGARHLIFLSRSAGSVTREDPYIKELAARGCSVQTFSGSVASGRDVKRVVAWAARPIAGVLQASMVLDDET

LGKMTFDQWQNATQPKIKGTWNLHEALQAQKQPLDFFFLFSSISGIGGQWGQANYAAANTFLDAFVQYRHSLGLPASVLD

IGAIDDVGYLSRNTSVLEALRATSLHILHEQDLLDSLQLMINRSHPPAPRTFLPSQLTTPYMNPSQVALGVRSNLPLSAP

NNRTIWKRDPRMSIYRNLESSNSTASTSSSSETLKQFLRDAAKNAALLDLPESVAYLAKETGTTLFGFMMRSEEELDLEA

PLASLGVDSLVSIELRNWFRQKVGAEFTVLEVVNSNSIMHLGQQAAERLKAKFQGRI

>Cocst1|662096

MANFQSQTATIPENLALNGNSQNGKISDGNTPNGNTSNGYSVTNGHLIGEHSNNGHSTNSFTPGESPPLTEPIAICGMAM

RLPGGIRDADSYWDLLYHKRSGRCKVPKDRYNIESFYGPGKIGHVTSKYGYFLEDHDLANIDPSFWSMTKQEIESMDPQQ

RMNLEIVYECLQNAGQKPNELRGRKVGVYVGTFDGDWLELDGRDTQHYHPYRLTGYADYMSANRIHYEFGFMGPSVTIRT

ACSSSLTGLYDACHSLLSGECEAAVVASSNLIFSPRTTSTMQEQGVMSPTGSCKTFDAHADGYARGEAVSAVYVKKLSDA

IKDGDPIRSVIRSTVINAGGRSSTITAPSSIAHEQLIRRGHKVAGISDFSKTAMIECHGTGTPVGDPIETAAVANIFGEY

GIYIGSVKPNIGHSESASGLSSVIKMTLALERKTIPPNINFETPNPKIPFERCKLVVPVEPMDWPADRLERVGVNSFGVG

GANAFVLLDSAASFGLGPKKVADTNRSLFEPTMRLLTFSAKHPEALRRSVADHESYLSSHPDSLRDISYSLTMKRDVLPH

RAFCVTNGQDSFEISRIHKPASKSPSSIVFCFSGQGAQWAQMGKELIQNETSFQKSIQGLDNVLANIHDPPGWKLFDEIM

APKKSSCLSQAELSQPCCTALQIALVDLLTAWNVKPDAVIGHSSGEIGAAYASGSITSREAILIAYYRGQVMLHLDSGLV

GGMAAVGLGAEAVTKYLQPGVNIGCENSPESTTLTGDKDVLKEVMETIRKENPETLVRLLVVDRAYHSEHMKRVEVEYVS

FLEKHIHALAPKVPFISSVTNTIITESGELGSTYWARNLVSPVLFSSAVETVIKTMPTPKVFLEIGPHSALAGPIRQIFR

HHGTTDEYLSTIKRGQNGHFEVLKAVGELWLSDHPVDFETINQGGDFLTDLPLYSWHYEEEPLWSESRLSKEWRFRKHSH

HDLLGSRVLESTDHNPTWRNIIRLDEISWIKEHEVTGDIVLPGVAYVCMAAEAIRQVTGSTDYTARKINIKAACIIQQGQ

DVEIITHLQRARLTNALDSSWFDFSVFSMNKGVWVQHAFGQIRPGSEIERNPIKLKPLPRELSRRGWYRKMEQMGLAYGP

RFRGLRNPTSHPTIKSAMAMVKNDLRPTDSTYAVHPASLDCLLQLLMIAASNGLTRRFNVLAVPTYIEEIYVRPPTGEMT

LQATADFPPKNGMVSGNVIATSDEVTIIQLKGLQTSSLGDVTDAVGHFHDLHAATELEWKPDLNYMDASDLFSIKRERGE

THDKLDKLGALCMLEAQERLDGFEPSQDHLNDFFQWLKSINGEFNDVQHSDSIRGSTVTETTPRQRLQIIQDLYRELQDS

DAASAATAIYRIMDQCKRIFSSDVDPLDLLLEDEVLTQLYNFMQMQNSEYTPFLELLAHRTPTLRILEIGAGTGGTTNTV

LPFLKSSYGERTYLSYTYTDISSGFFVQARERFKEYANMDYAILDISKDPIQQGFTAESFDLIIATNVLHATPFLKETLA

NVRKLLHPGGRLFLQELSPVTKWINFVMGVLPGWWLGKADGRYPEPYISAERWDKELIASGFNGINAVHHDGYLDNNIIA

MPSRPSPTSSRLTLLCVDHEAKSVTTIAAHFRGKGYEIDYCTLDELPPPKQDVVSLLDLDHPFLHDATEREFKSFVKFVD

HITDSGMLWVTGACQVACRDPRYALINGVSRVCRTESQMDFGTLELDSFEDDALSVVSNVFCEFITRIHEAEVSPNLEWA

YNNGFVLISRYHFIDVLEEMKSMKNPTASRKLEIHRPGLTNTLSWEQVDTPPLGDNEVEFDIKSVGLNFKDVLISMGIIT

DHRAIGSGLGLENSGVITAVGSKVSSMKVGDRVVGCRTGCFMTKMVLREDLCVKIPPVSKLTFAEAASMPVVCCTAIYAL

EDLAKLDSKKTILIHSAAGGVGIAAVQIAKMLGAEIFCTVSTQEKIDFLVQTYGIPESHIFNSRDSNFVLGIMEQTQTRG

VDVVLNSLSGELLHSSWTCVAEFGIMVEIGRRDFIGFGKLGMEMFDGNRTFTGFDLSLIAADRPQLVIQLMARCLKYHAQ

GFLTPITPIHEFPALQIQEGMRYIQRGQHIGKVVITMPERPEDLPSQSVRKNLTLNPDKAYLFVGGIGGLGRSIANWLIE

RGAKHIVFFSRSAGKTPQSTALFSELEVQGCIATAFAGDVCNYSDVQAVVNSIRIPIGGVFQAAMVLDDVSLSDMTFDQW

QTAMLPKVKGTWNLHSALESHNSTIDIFFLFSSAGCTMGQWGQANYNAGNTFLDAFVQYRHSLGMPASTVDIGVIEDVGY

VAENPSVLDSLRATGQYLLQERELLESIELAMNRSAAPDPKNSTLHSLVPRYANPSQIVIGLRSVLPLTAPNNRTIWRKD

PRMAIYRNLEQQDTTSSDSDNDELQRFMKDITTNMTLLRAPESALLLASAIGKTLFGFLMQDEAEVDLGSPLASIGIDSL

ISIELRNWIRRSIGVEFTVLEIVRADDIKNLGLQAQEKLIEKFEAKI

>Colto1|2794

MSDAEVENQPASKEGHDGFHRLPGPERCQERLVDTVNEFPKAIEFNANRHPASENTANEAPMNGASHGATSNAQCDISNC

SGSSHSSTTGSRPTVEPIAICGMAMRLPGGITDAAGFWDMLYNGRSGRCKVPEDRYNAETWYGPGKIGHTASKFGYFLDN

VDLANMDSSFWTMTKKEIGAMDPQQRLTLEVVYECLQSAGQKTDELRGKKVGVFIGTFEGDWLELDGRDPQHHHMYRLTG

YGDYMSANRINYEFDFVGPSVTIRTACSSSLTALHDACHSILTGECESAVVACANIICSPRTTMTMQEQGVMSPSGLCKT

FDAEADGYARGEAVSAIYVKKLSDAIRDGDPIRSVIRSTAINAGGKSSTLTAPKTAAHEALIRRAHQLAGISDFSKTAMI

ECHGTGTAVGDPIETLAVANIFGDYGIYIGSVKTNLGHSEGASGLSSVIKMTLALENETIPPNLNFTTPNPKIPFKKCKL

AVPIEPRPWPKDRDHVVGVNSFGIGGSNAHVLLSSAFSFGCGNGKVSGQQQGNVEEAYDASPRLRLLLFSAKHPKALQTM

LSQHQAYHLSHPSLLRDMSFSLALKRDVFNHRAFCVTDGVDDWAPVVSPRPATREPSKLIFVFSGQGAQWARMGMALIKQ

VPEFKQSLRDLDKFLHMLPDGPDWNLIGKSRNQTSTQYLLTTVRVDELLAPKSRSRISSAELSQPCCTAIQLALVDLLAS

YNVKPGAVVGHSSGEIAAAYASGAITAKQAIAIAYYRGKVMLALDPAKTRGGMAAVGLGRDKVEPYLSNGVMIGCENSPE

STTLTGEKKALEWVIQQIRETSPDVLKRNPRRTTLTLLTTLTDHMRQVAPLYKELLGGDMTNAKDPIIPFYSSVFCKSIK

SGRELGPKYWVDNLVSPVRFSTAVGQILQEPGHKTFVEIGPHAALAGPVRQMIAKSAKPADDYMSVLTRGNDSHADLLHV

VGQLWSTNQSVYLDPIVGEGEFLVDLPLYPWHYEEPLWYESRLAREWRRRQFPHHDVLGSRILESTDFSPGWRNLLRPET

VPWIKEHEVAGDIVFPGVGYVTMAGEAIRQLTASINFTVRRVHIKAALILLQDTATEVITQLQRVPLTSSADSAWYNFTI

SSHQNGGWQKHAFGQVSAGSEHPHDKVTDMAPLPRVVSPKAWYRKLRSLGLEYGPRFMGMRDMTAHPVEPTLMLHMTNDI

GDGESVYAIHPVTLDLVPQALAPALANGLTRRFDCVAIPTYIEEMYVRPPATADIVMQVHVTDRRKNTHIGDMVAVSDGE

VVISVKGLQVSVISEADNDGDGRHMQDPHAAVELEWKEDINLMDMTTLIRPAEDQYDVRRLLDSFSVLCMLEAAERLSAV

DVKPSKPHLAHFRHWLEDLCGEIREGRYGTGILPAGFHTDMPPARRTETVDALYSRLLETKASAAATAVYRIASSCESIF

CGTTDELTVLLEDNNLHRLYDFMQNTEYSAFLDLMAHRKPSMRVLEIGAGTGGTTATVLPVLESAYGERMYLSYTYTDVS

PGFFPAAKERFKEHDAVQYAVLDISKDPLGQGFDPESFDLIIACNVLHATPNIHDTLSNVRKLVHPRGKLLLQELSPETK

WINFVMGVLPGWWLGADDSRFPEPFIDSARWDMELRAAGFGGAEAVVYDGYLNNNIIATPAALQPPLSKRVTLLHSGEIG

AVGRHDTAVYKLHARLELSGYQIDLRAFNYDGNAAAPSLSLPEGQDVVAALDLTGPFFHDLTERRLAGFQELVRQARDRR

CGILWLTGTSQADCVDPRFAPVIGVARVLRTETGLDFATLELELDRLDKDGGSSIGAVPAVLAEFQRREADEDITPEAEW

ACVGGQVMIGRYHFVDVYKGMKTDDNNRDDGGTVLKLEQHRPGLVNTLFWERRAQPPLGDNDVRVQVKAVGLNFKDVLIS

LGVITEPYSIGRGLGYECSGTVTAVGPSVSEFRVGDRVIAGSSGSFTTVLQAPENLCCNIPDSMSFEEAATIMAVYCTAI

HCLLDVGRLTKGMSVLIHSAAGGVGIAAIQVAKMVGAVIYCTVSNNSKAEFLVDRFGIPRRHIFNSRDATFLPAVLEATE

GRGVDVVLNSLAGELLHASWKCVAMFGTFVEIGRRDFVGQGLLAMDTFEANRSFVGFDLLRFTTERPLTVKSLIRRALAF

YTEGHIRPISPSTTLPATEISEAIRFMQKGQHLGKIVVIMPENHNELVSEKPHSSIVLRNDAAYLLVGGLGGLGRSITTW

LAERGARHFVFLSRSAASVSDDDPFILELEALECMTVRVSGDVANYEDVLRSIKAAGRPIAGVLQASMVVRDNNLIDMSW

DEWVAASRPKIQGTWNLHNAFVREQAEPLDLFFLFSSAGAMSGHWGQANYNAGNTFLDAFVQYRHSLGLPASVLNIGIMG

DVGYVSENTGLLESLRSTSQYVMEESALLECIELMLKRCFVAQEVPAPAAVSSGGGGDNGGGSRWRYAQRSQMGIGLRSL

LPITAPANRTTWRKDPRFLVYRNLEEAGSGASGAAGALTSDEALAQLLREIGTNMMLLRSTETAVLLARALRNTLLGFMM

RTEDELDLDGSLTSMGIDSLISIELRNWIRRQLGAEVTVLEIVRAASLKDLGEMVQRRLVEKYEARAK

>Colto1|4996

MTFDFQKYHGGESLPSANGINGNGITLDGVDLNDTNGINNNFGTKTHADASGDCNTEPIEPVAICGMGMRLPGGVNDANA

FWDMLLNKRDGRCAVPKDRYNAEAWYAPGKKRHVPSEYGYFLDSNIDLKNVDASFWSMTKKELETLDPQQRLALEVVYET

LQSAGQKPSEIRGRKIGVWVGSFGGDRAELDARDPQAVHPYNLLNSFDFMPADRIHYEFGLMGPSVTVRTACSSSLLGLH

QACHALIHGDCEAAVVAGTSIIYSPTLTATMNEHNVLSPSGTCKAFDAEADGFVRGEAVLAVYVKRLRDAVRDGDPIRSV

VLATASNSSGKSSTMTAPNSDAQESLIRRAHELAGIKDYSRTAMMECHGTGTPVGDPIEAEAVGRVFGEHGGVYIGSVKT

NVGHVEGAAGLASVLKMTLALENDTIPPNANFKSPNPKIPFQRYQLRVPTEPMPWPEGRDRVVGVGSYGVGGSNAYALLA

SAGHLGIQSRDRGRDASPQVAVTSTVESPKLLLFSANHPQALETMVMRHQSYCLAHPDRLGDMAYTLAMRRETLSHRAVC

VTNGIDDLAPIKSTRHGPYDPATLVFVFTGQGAQWPQMGKSLIQELPSFRSSMIEMENILQQLPDAPDWKLTDHILAPKK

VSMINDGQISQVCCAALQIALVDILETLNIKPGAVLGHSSGEIAAAYACGSITQSEAIIIAYYRGKILAAVDGSVGGMAA

IGLGKAQVTPHLRPGVLVGCENSPNSITLTGDKQILQMVVNDIKGAYPDVLARTLQVDRAYHSHHMQAVALRYLDLLKPY

VKPRDPKTPFISSVSNQLISTAADLDSAYWAQNLVSPVLFSGAVTKAIHALKSDKVFLEIGPHSALAGPIRQILAAEKVN

AEYISVLTRGRDSHQDLLRAVGELWLHNQPVALNHVVEKGQILTDLPLYPWHYEESLWHESRLSEEYRLRKFRHHELLGS

RISESTSSNPAWRNLLRLEDVPWIAEHEVEGIIIAPGVSYLCMAGEAVRQLTGEVGFTCKQVHFNAALLMTYESQTEVIT

QLTRIGLTDSIDSDWYNFTISSYCDGDWVKHAFGKVRSSSEGLDGSLNMPSPQSTRSVFPRTCKSASWYRKFRSLGLEYG

PRFSGMMNITADPLTPKLTATLKLDMVLGEEKYYSIHPGALDRLVQSLYIASAHGLTRNCTTLALVAYVDEFTMTPPPNG

AKEMKFLANVTEQRPGAFLGSIVASVDGRNAVVNSRGWQLTRISESNETHSDLNSHGAAQLEWREDVEFINPRSLISPAI

TPAKAELYQLLDRFNLLSLARTLDNARRAPQPRRDHLAKYQKWLEDTVANLASGSLKCPGVPDSQNLVTMTAEQRDELLL

SMYKQLQGTEVDAPATAIHRVSSHCEGILNGDTSELELLLADGVLQHVYDCLLLDTESSTLFSLIGHKKPNLRVLEIGAG

TGGATASTLKGLISAQGEKMYQSYTYTDISPGFFADAKERFKEYSGLDFALLDVSKDPLEQGFNAESYDLIIAWNVIHAT

PDLHQSLTNIRKLIHPQGWFLLQEIAPLTTWINHCFGVIPGWWLGEANGRASAPHVDLDRWKKELFESGFRDVSNTYDGY

TNNNIVSRPAPPRFRPKRVTLLKRASQQVQHIQAGLQAAAYEVDEYVLDDRAARLPPGQAVISTLDICSPFLTGLDEQEF

ASFQRFIKAAKDGACGILWLTGPCQVGETVRPEYAPLLGVARVLRTELGLDFATLELDSFSRAEAAEVIQKVFSEFEQRI

SDDPDANPEYEWAYTDGKLLVSRYRHVKVEQESYELQADMTVLKLEQRKPGLTDTLCWKLLTPPTLKDDEVRIDVKAIGM

NYKDVLIAQGVITDTAAIDAGLGLECAGVVSEAGPDVDRLKVGDRVAVISSGSFTNSKAVSQQLCCKIPDETSFEEAAVI

PVAYCTAFYSIFNHGQATKGMSILIHSATGGLGLAALQLAQMLEANIYCTVGSQAKREFLVKECKIPPEHIFNSRDSSFL

PGIMAITEGRGVDLVLNQLSGELLHASWQCVAEFGIFVELGRRDFLGHAKLAMEQFESNRTFVGVDLTHLWIRKPSVVGA

MMKRVMQLWTQGHIKPRITSKFSAAEVCQAFRQMQKSQHIGKLVVTMPEDAAARGLPTEPTYKALKLRHDRSYLFTGGLG

SLGQVISTWLAEKGAKEIIFLSRSAGSLPEHATIAEELSALGCKATIVSGDVAMYEDVARALETATMPVGGVLHAAMALR

DVGFLGMRWADWLTASRPKIDGTTNLHEALLKQQPDFPVDFFLLFSSTAATAGWWGQANYHAGNTYMESFAAYRRNLGLA

ASVLNVGFISDAGYVADRPEAADSARATGQWFNTEIELLNCIERMFMESLAGFNPKDSPCWVQRSLLAMGMRSTIPLTSK

TCRVPWKRDRRMLALCNVEPSDSIGSSGSDSMSNEELGRSVRDLSSNIVKLQSDETTTFLATHIGKTLCRFLLRSDADLD

LQARLNDIGMDSLVSIEIRAWIRQWMGVDLATLEITGSENLHKLAVAVQGRMISKYNSKI

>Coltof1|158352

MSDAEVENQPASKEGHDGFHRLPGPERCQERLVDTVNEFPKAIEFNANRHPASENTANEAPMNGASHGATSNAQCDISNC

SGSSHSSTTGSRPTVEPIAICGMAMRLPGGITDAAGFWDMLYNGRSGRCKVPEDRYNAETWYGPGKIGHTASKFGYFLDN

VDLANMDSSFWTMTKKEIGAMDPQQRLTLEVVYECLQSAGQKTDELRGKKVGVFIGTFEGDWLELDGRDPQHHHMYRLTG

YGDYMSANRINYEFDFVGPSVTIRTACSSSLTALHDACHSILTGECESAVVACANIICSPRTTMTMQEQGVMSPSGLCKT

FDAEADGYARGEAVSAIYVKKLSDAIRDGDPIRSVIRSTAINAGGKSSTLTAPKTAAHEALIRRAHQLAGISDFSKTAMI

ECHGTGTAVGDPIETLAVANIFGDYGIYIGSVKTNLGHSEGASGLSSVIKMTLALENETIPPNLNFATPNPKIPFKKCKL

AVPIEPRPWPKDRDHVVGVNSFGIGGSNAHVLLSSAFSFGCGNGKVSGQQQGNVEEAYDASPRLRLLLFSAKHPKALQTM

LSQHQAYHLSHPSLLRDMSFSLALKRDVFNHRAFCVTDGVDDWAPVVSPRPATREPSKLIFVFSGQGAQWVRMGMALIKQ

VPEFKQNELLAPKSRSRISSAELSQPCCTAIQLALVDLLASYNVKPGAVVGHSSGEIAAAYASGAITAKQAIAIAYYRGK

VMLALDPAKTRGGMAAVGLGRGKVEPYLSNGVMIGCENSPESTTLTGEKKALEWVIQQIRETSPDVLVHHMRQVAPLYKE

LLGGDMTNAKDPIIPFYSSVFCKSIKSGRELGPKYWVDNLVSPVRFSTAVGQILQEPGHKTFVEIGPHAALAGPVRQMIA

KSAKPADDYMSVLTRGNDSHADLLHVVGQLWSTNQSVYLDPIVGEGEFLVDLPLYPWHYEEPLWYESRLAREWRRRQFPH

HDVLGSRILESIDSSPGWRNLLRPETVPWIKEHEVAGDIVFPGVGYVTMAGEAIRQLTASIDFTVRRVHIKAALILLQDT

ATEVITQLQRVPLTSSADSAWYNFTISSHQNGGWQKHAFGQVSAGSEHPHDKVTDMAPLPRVVSPKAWYRKLRSLGLEYG

PRFMGMRDMTAHPVEPTLMLHMTNDIGDGESVYAIHPVTLDLVPQALAPALANGLTRRFDCVAIPTYIEEMYVRPPATAD

IVMQVHVTDRRKNTHIGDMVAVSDGEVVISVKGLQVSVISEADNDGDGRHMQDPHAAVELEWKEDINLMDMTTLIRPAED

QYDVRRLLDSFSVLCMLEAAERLSAVDVKPSKPHLAHFRHWLEDLCGEIREGRYGTGILPAGFHTDMPPARRTETVDALY

SRLLETKASAAATAVYRIASSCESIFCGTTDELTVLLEDNILHRLYDFMQNTEYSAFLDLMAHRKPSMRVLEIGAGTGGT

TATVLPVLESAYGERMYLSYTYTDVSPGFFPAAKERFKEHDAVQYAVLDISKDPLGQGFDPESFDLIIACNVLHATPNIH

DTLSNVRKLVHPRGKLLLQELSPETKWINFVMGVLPGWWLGADDSRFPEPFIDSARWDMELRAAGFGGAEAVVYDGYLNN

NIIATPAALQPPLSKRVTLLHSGEIGAVGRHDTAVDRLHARLELLGYQIDLRAFNDDGNAAAPSLSLPEGQDVVAALDLT

GPFFHDLTERRLAGFQELVRQARDRRCGILWLTGTSQAGCMDPRFAPVIGVARVLRTETGLEFATLELELDRLDKYGGSS

IGAVPAVLAEFQRREADENITPEAEWACVGGQVMIGRYHFVDVYKGMKTDDNNRDDGGTVLKLEQHRPGLVNTLFWERRA

QPPLGDNDVRVQVKAVGLNFKDVLISLGVITEPYSIGRGLGYECSGTVTAVGPSVSEFRVGDRVIAGSSGSFTTVLQAPE

NLCCNIPDSMSFEEAATIMAVYCTAIHCLLDVGRLTKGMSVLIHSAAGGVGIAAIQVAKMVGAVIYCTVSNNSKAEFLVD

RFGIPRRHIFNSRDATFLPAVLEATEGRGVDVVLNSLAGELLHASWKCVAMFGTFVEIGRRDFVGQGLLAMDTFEANRSF

VGFDLLRFTTERPLTVKSLIRRALAFYTEGHIRPISPSTTLPATEISEAIRFMQKGQHLGKTVVIMPENHNELVSEKPHS

SIVLRNDAAYLLVGGLGGLGRSITTWLAERGARHFVFLSRSAASVSDDDPFILELEALECMTVRVSGDVANYEDVLRSIK

AAGRPIAGVLQASMVVRDNNLIDMSWDEWVAASRPKIQGTWNLHNAFVREQAEPLDLFFLFSSAGAMSGHWGQANYNAGN

TFLDAFVQYRHSLGLPASVLNIGIMGDVGYVSENTGLLESLRSTSQYVMEESALLECIELMLKRCFVAQEVPAPAAVSSG

GGGDNGGGSRWRYAQRSQMGIGLRSLLPITAPANRTTWRKDPRFLVYRNLEEAGSGASGAAGALTSDEALAQLLREIGTN

MMLLRSTETAVLLARALRNTLLGFMMRTEDELDLDGSLTSMGIDSLISIELRNWIRRQLGAEVTVLEIVRAASLKDLGEM

VQRRLVEKYEARAK

>Coltof1|159858

MTFDFQKYHGGESLPSANGVNGNGITLDGVDLNDTNGINNNFGTKTHADASGDCNTEPIEPVAICGMGMRLPGGVNDANA

FWDMLLNKRDGRCAVPKDRYNVEAWYAPGKKRHVPSEYGYFLDSNIDLKNVDASFWSMTKKELETLDPQQRLALEVVYET

LQSAGQKPSEIRGRKIGVWVGSFGGDRAELDARDPQAVHPYNLLNSFDFMPADRIHYEFGLMGPSVTVRTACSSSLLGLH

QACHALIHGDCEAAVVAGTSIIYSPTLTATMNEHNVLSPSGTCKAFDAEADGFVRGEAVLAVYVKRLRDAVRDGDPIRSV

VLATASNSSGKSSTMTAPNSVAQESLIRRAHELAGIKDYSRTAMMECHGTGTPVGDPIEAEAVGRVFGEHGGVYIGSVKT

NVGHVEGAAGLASVLKMTLALENDTIPPNANFKSPNPKIPFQRYQLRVPTEPMPWPEGRDRVVGVGSYGVGGSNAYALLA

SAGHLGIQSRDRGRDASLQVAVTSTVESPKLLLFSANHPQALETMVMRHQSYCLAHPQRLGDMAYTLAMRRETLSHRAVC

VTNGIDDLAPIKSTRHGPYDPATLVFVFTGQGAQWPQMGKSLIQELPSFRSSMIEMENILQQLPDAPDWKLTDHILAPKK

VSMINDGQISQVCCAALQIALVDILETLNIKPGAVLGHSSGEIAAAYACGSITQSEAIIIAYYRGKILAAVDGSVGGMAA

IGLGKAQVTPHLRPGVLVGCENSPNSITLTGDKQILQMVVNDIKGAYPDVLARTLQVDRAYHSHHMQAVALRYLDLLKPY

IKPRDPKTPFISSVSNQLISTAADLDSAYWAQNLVSPVLFSGAVTKAIHALKSDKVFLEIGPHSALAGPIRQILAAEKVN

AEYISVLTRGRDSHQDLLRAVGELWLHNQPVALNHVVEKGQILTDLPLYPWHYEESLWHESRLSEEYRLRKFRHHELLGS

RISESTSSNPAWRNLLRLEDVPWIAEHEVEGIIIAPGVSYLCMAGEAVRQLTGEVGFTCKQVHFNAALLMTYESQTEVIT

QLTRIGLTDSIDSDWYNFTISSYCDGDWVKHAFGKVRSSSEGLDGSLNMPSPQSTRSVFPRTCKSASWYRKFRSLGLEYG

PRFSGMMNITADPLTPKLTATLKLDMVLGEEKYYSIHPGALDRLVQSLYIASAHGLTRNCTTLALVAFVDEFTMTPPPNG

AKEMKFLANVTEQRPGAFLGSIVASVDGRNAVVNSRGWQLTRISESNETHSDLNSHGAAQLEWREDVEFINPRSLISPAI

TPAKAELYQLLDRFNLLSLARTLDNARRAPQPRRDHLAKYQKWLEDTVANLASGSLKCPGVPDSQNLVTMTAEQRDELLL

SMYKQLQGTEVDAPATAIHRVSSHCEGILNGDTSELELLLADGVLQHVYDCLLLDTESSTLFSLIGHKKPNLRVLEIGAG

TGGATASTLKGLISAQGEKMYQSYTYTDISPGFFADAKERFKEYSGLDFALLDVSKDPLEQGFNAESYDLIIAWNVIHAT

PDLHQSLTNIRKLIHPQGWFLLQEIAPLTTWINHCFGVIPGWWLGEANGRASAPHVDLDRWKKELFESGFRDVSNTYDGY

TNNNIVSRPAPPRFRPKRVTLLKRASQQVQHIQAGLQAAAYEVDEYVLDDRAARLPPGQAVISTLDICSPFLTGLDEQEF

ASFQRFIKAAKDGACGILWLTGPCQVGETVRPEYAPLLGVARVLRTELGLDFATLELDSFSRAEAAEVIQKVFSEFEQRI

SDDPDANPEYEWAYTDGKLLVSRYRHVKVEQESYELQADMTVLKLEQRKPGLTDTLCWKLLTPPTLKDDEVRIDVKAIGM

NYKDVLIAQGVITDTAAIDAGLGLECAGVVSEAGPDVDRLKVGDRVAVISSGSFTNSKAVSQQLCCKILDETSFEEAAVI

PVAYCTAFYSIFNHGQATKGMSILIHSATGGLGLAALQLAQMLEANIYCTVGSQAKREFLVKECKIPPEHIFNSRDSSFL

PGIMAITEGRGVDLVLNQLSGELLHASWQCVAEFGIFVELGRRDFLGHAKLAMEQFESNRTFVGVDLTHLWIRKPSVVGA

MMKRVMQLWTQGHIKPRITSKFSAAEVCQAFRQMQKSQHIGKLVVAMPEDAAARGLPTEPTYKALRLRHDRSYLFTGGLG

SLGQVISTWLAEKGAKEIIFLSRSAGSLPEHATIAEELSALGCKATVVSGDVAMYEDVVRALETATMPVGGVLHAAMALR

DVGFLGMRWADWLTASRPKIDGTTNLHEALLKQQPDFPVDFFLLFSSTAATAGWWGQANYHAGNTYMESFAAYRRNLGLA

ASVLNVGFISDAGYVADRPEAADSARATGQWFNTEIELLNCIERMFMESLAGFNPKDSACWVQRSLLAMGMRSTIPLTSK

TCRVPWKRDRRMLALRNVEPSDSIGSSGSDSMSNEELGRSVRDLSSNIVKLQSDETTTFLATHIGKTLCRFLLRSDADLD

LQARLNDIGMDSLVSIEIRAWIRQWMGVDLATLEITGSENLHKLAVAVQGRMISKYNSKI

>Conli1|2211

MAAPENHVHSNGISGSNGYINGDINGHTNGVTALDGKGQGTYEPIAIVGMGMRLPGGVHNAESYWDLLVNGKEGRCRVPK

DRYNIDTWFGPGRVTHVGTQYAHFLEELNLANIDPNFWSFTKQEAELLDPQQRLFLEVVYEALENSGKTGWRGKDIGVYV

GALGDDWGEMEMQDGQDLNPTRTYVYGDYIIANRASMVVRTACSSSLMALHLACQDIHSGDCSDGAIVGGINLIITPRTT

VALTEQGVLSEDGRCKTFDADADGYARGEGVSAIYIKKLSDAIRDGDPVRAVIRSTCVAADGKTAGFTLPNPASHEKLMR

RGHHLAGIYDFSKTAMVECHGTGTAVGDPREVGAVANVFGKHGVYIGSVKPNIGHGEGASGLSSIMKMTLALEHKIIPPN

INFKNPSPKIPWKEAKLTVPTKPLPWPTDRFERVAVNSFGIGGANAHVILESAASWGVGKAPELSSSVAEGAYKKHLLLF

SAKHQKSVEVSAQKHEEYLTTHPDSIADMAYTLNTKREVHPVRAFCVTDGLDSFQLSRIVKPTPGTENSLIFAFTGQGAQ

WARMGRELLLSEGLFKDTIDALDVVLSQLPGGPKWTLKDEILAPKKTSRLAEAELSQPCCTAIQIALVDLLQSWGITPTA

VIGHSSGEIAAAYACGAITAEDAVRIAYHRGQCTLALKDSHKGGMAAVGLGRETVEQYLRAGVTIGCENSPASVTLTGDS

HVLEKVMEDIRAAEPLALVRALRVECAYHSDHMKVVAKDYTERLGLFQAKQPQIPFYSSVTKQRNPDLSTSYWVLNLISP

VLFKSAVASALQSHENATFVEVGPHSALQGPIRQNIQGMGKSADYIATLVRDIDATQALLSTAGNLWLNGIKIDLHAVNG

SPKAVTLLTNLPTYSWRYDGDYWLENRLSRDWRFRKHDHHELLGARILESADAGPAWRCKLRMQDAPWLSDHDILGDIIF

PGAGYLAMAGEAVKQLRPGSDDFSLRRVTLSAALVLHGDPVELVTTLVPVRLTTTIESQWYTFTISSVNAVTNAWTKHVS

GQVRAGRDDASVPEMPEIEILPRKVPTSSMYSAWRRFGLNYGGRFRGLDDISAHTTEQKAVGTIHDKCTPKEAAVYSLHP

AGIDAAFHLSNVCLCHGLGRNFRTPSVPKYIEEMYVGKPDGPIRVVGDAASKGRGGSTSNLVGVSSGKIVLSWKGLELSP

LSDGSDVVDEDPHAAAILEWKADIDFLNPGGLLLSLKKDADDPMHRLVDTMGLACMIESRHQLAGLETSHWHLVKFRDWL

DIPYQEAVEGRYPHVPNCAEIATMSSPERVRLIEDYLAASEGTEANPVAISLWRIFSNAVGFFSGEADPLEVLMADNILM

RMYDFTNNADAVQFLGLLGHKKPVMRVLEIGAGTGGTTATILAALKSDQGERLYGTYVYSDISAGFFLGAKERFKDYTGI

EHHVLDITVDPMSQGFVPESFDLIVSSNCLHATPNLVQTLTNVRKLLHPDGRLFLMELSPEASKSVNYVMGPLVGWWLSD

DGRDHEPYVSAAVWHDRLLQSGFAGVEAYAFDGNMSNSIIARPAVPPPPRLTGVSVIATDANNPNVSAAVQHLRHIGLAV

EIFPVGATLPPSRPGVFMLDLEAPFLTGVTEAQFNAFKHTLFSVPDTPLLWVTGACQVACTNPQYALVNGMSRSIRQEMG

LDLVTLELEKYDTAAWQGMAELLATFPSRVNLTGEERETDWESEYAFHAGVIQVGRMHWIKINEELKEMEEHHHAKRLVI

EKPGIIQTLKWRRVAPPAEERGDWVNVDTRSVGLNFKDVLIAMGIVDPGGDSKGDFGFEGAGVITGVGPEVKHLSVGDRV

AFSSTGCFATSLTMAEITCTKIPDMMSFEEAATIPCVFGTAMYGLEDLARLEEGQTVLIHSACGGVGQAAIQIAKLIGAE

IYCTVGNEDKVEYLVKTFCLPRDHIFNSRDSSFLAGILNATNDKGVDVVLNSVSGELLHDSWRCVAKFGTMVEIGKRDFI

GKAELAMDRFENNRTFVGLDHTELWAHKPKAAHRVLDKIMRLCEEGKLAPISPIKTFEAAKIEEAFRYMQKGQHIGKIVV

NIPEGRADNVLEAVPARRELRLRGDRTYIFAGGLGGLGQSIATYLAELGARHLIFFSRSADKFAKSNPEFGRELESLGCH

VQFIGGSIIELQDVEKVISSAVNPIGGVLQAAMVLQDANFVDMTFEAWQTAVLPKVTGTWNFEKALKKQSEPLDFFFLFS

SVSGTAGQIGQANYAAGNTFMDAFVQYRRAQNLACSTLAIGIMEDVGFLARERHLLEALRATSLHFLYEQDLLDSLELSL

GPWASAPEDPGIPVKELSAINQATRGYVNPGHVILGLRSKLPLLSPMNRTGWKKNPRLLVYRNIELQGDTAAGPTNTDGG

LREFLASCGKTPELLETEATAGFLAKEIGATLFNFMMRGDEEPELTVPLANVGVDSLMSIELRNWFRQKIGVPFTVVEIV

GAASIAELGKMTAEKLAEKHKRR

>Conlig1|606701

MGMRLPGGVHNAESYWDLLVNGREGRCRVPKDRYNIDTWFDPGRVTHVGTQYAHFLEELNLANIDPNFWSFTKQEAELLD

PQQRLFLEVVYEALENSGTTGWRGKDIGVYVGALGDDWGEMEMQDGQDLNPTRTYVYGDYIIANRASYEFDLKGPSMVVR

TACSSSLMALHLACQDIHSGDCSDGAIVGGINLIITPRTTVALTEQGVLSEDGRCKTFDADADGYARGEGVSAIYIKKLS

DAIRDGDPVRAVIRSTCVAADGKTAGFTLPNPASHEKLMRRGHHLAGIYDFSKTAMVECHGTGTAVGDPREVSAVANVFG

KHGVYIGSVKPNIGHGGGASGLSSIMKMTLALEHRIIPPNINFKNPSPKIPWKEAKLTVPTKPLPWPTDRFERVAVNSFG

IGGANAHVILESAASWGVGKAPESSSSVAEGAYKKHLLLFSAKHQKSVEVSAQKHEEYLTTHPDSIANLAYTLNTNREVH

PVRAFCVTDGLDSFQLSRVVKPTPGTTNSLIFAFTGQGAQWARMGRELLLSEGLFKNTIDALDVTLSQLPDGPKWTLKDE

ILASKKTSRLAEAELSQPCCTAIQIALVDLLQSWGITPTAVIGHSSGEIAAAYACGAITAEDAVRIAYHRGQCTLALKDS

HKGGMAAVGLGRETVEQYLRTGVTIGCENSPASVTLTGDSDVLEKVMEDIRAAEPLALVRALRVECAYHSDHMKVVAKDY

TERLGLFQAKQPKIPFYSSVTELPNLDLSTSYWVLNLISPVLFKSAVASALQSQENATFVEIGPHSALQGPIRQTIQGMG

KSADYIATLVRDIDATQALLSTAGNLWLNGITIDFHAVNGSPKAVRLLTNLPTYSWRYDGDYWLENRLSRDWRFRKHDHH

ELLGARILESADAGPAWRCKLRVQDAPWLSDHDILGDIIFPGAGYLAMAGEAVKQLRPGSDDFSLRRVTLSAALLLHGDP

VELVTTLVPVRLTTTIASQWYTFTISSVNEVTNAWTKHVSGQVRAGRDDASVPEMPEIEILPRKVPTSSMYSVWRRFGLN

YGGRFRGLDDISAHTTEQKAVGTIHDKCTPKEAAVYSLHPAGIDAAFHLSNVCLCHGLGRNFRTPSVPKYIEEMYVGKPN

GPIRVVGDAASKGRGGSTSNLVGVSSGKIVLSWKGLELSPLSDGSDVVDEDPHAAAILEWKADIDFLNPGGLLLSLKKDA

DDPMHRLVDTMGLACMIESRHQLAGLETLHWHLVKFRDWLDIPYQEAVEGRYPHVPNCAEIATMSSPERVRLIEHSLAAS

EGTEANPVAISLWRIFANAVGFFSGEADPLEVLMADNILMRMYDFTNNADAVQFLGLLGHKKPVMRVLEIGAGTGGTTAT

ILAALKSDQGERLYGTYVYSDISAGFFLGAKERFKDYTGIEHHVLDITVDPISQGFAPESFDLIVSSNCLHATPNLVQTL

TNVRKLLRPDGRLFLMELSPEASKSVNYVMGPLVGWWLSDDGRDHEPYVSAAVWHDRLLESGFAGVEAYAFDGNMSNSII

ARPAAPPPPRLTAVSVIAADANNPNVSAAVQHLRHIGLAVEIFPVGATLPPSRPGVFMLDLEAPFLAGVTEAQFNAFKHT

LFSVPDTPLLWVTGACQVACTNPQYALVNGMSRSIRQETGLDLVTLELEKYDTAAWQGMAQLLATFPCRVNLTGEERETD

WESEYAFHAGVIQVGRMHWIKINEELKETEEHHPAKRLVIEKPGIIQTLKWRRVAPPAKEGGDWVNVETRSVGLNFKDVL

IAMGIVDPGVDGKGDFGFEGAGVITGVGPEVKHLSVGDRVAFSSTGCFATSLTMAEITCTKIPDMMSFEEAATIPCVFGT

AMYGLEDLARLEEGQTVLIHSACGGVGQAAIQIAKMMGAEIYCTVGNEDKVEYLVKTFCLPRDHVFNSRDSSFLTGILNA

TNGKGVDVVLNSVSGELLHDSWRCVAKFGTMVEIGKRDFIGKAELAMDRFENNRTFVGLDHTELWAHKPKAAHRVLDKIM

RLCDEGKLAPISPIKTFEAAKIEEAFRYMQKGQHIGKIVVNIPEGQADNALEALPARRELRLRGDRTYIFAGGLGGLGQS

IATYLAELGARYLIFFSRSADKFAKSHPEFGRELESLGCHVQFIGGSIIELQDVEKVISSAVNPVGGVLQAAMVLQDANF

VDMTFEAWQTAVLPKVTGTWNFEEALKKQSEPLDFFFLFSSVSGTAGQIGQANYAAGNTFMDAFVQYRRAQNLACSTLAI

GIMEDVGFLARERHLLEALRATSLHFLYEQDLLDSLELSLGPWASAPEDPGIPVKELSTISQATRGYVNPGHVILGLRSK

LPLLSPMNRTGWKKNPRLLVYRNIELRGDTAAGPTNTDGGLREFLASCGKTPELLETEATAGFLAREIGATLFNFMMRGD

EEPDLTVPLANVGVDSLMSIELRNWFRQKIGVPFTVVEIVGAASIAELGKMTAEKLAEKHKRR

>Corma2|758436

MEFSSQPNGHSRASIGKPLYEPIAICGMGMRLPGSINDSQAFWDLLQNKRNGRCKVPKERYDVDTWFGPGKSGHVASKFG

YFLDHVDLAKMDTSFWSATKDECASMDPQQRLLLEVAYEALQTAGQKSSELRGRKIGVFVGSFEGDWLELDQRDVENFSR

HRQSGSGDYMAANRIQYEFGFMGPSTVIRSACSSSMIALHDACTAIIAGECEGAIVGGCNLILSPRMTATMQELGVASPS

GYCHTFDADADGYARGEAVSAVYIKKLSDAIRDGDPVRSVIRSTCFNSGGRAATMSAPVAAAHEALMRRSHELAGITDFS

RTAMVECHGTGTPVGDPIEVQAVANVFGDHGIYIGSVKTNLGHGEGASAMSSLIKMTLALEHKIIPANLNFKTPNPSIPF

KSRKLAVPLESTPWPKDRDEVVGINSFGVGGSNAHAVLASADSFGAGMAQESAAEKTTVSWKPALLLFSAKHADALKKII

ENQQAYHVAHPNRLTNVAYSLAMKRDAFDHRAFAVADGVDDWSAKFSTRTSAPREPPKLVFVFSGQGAQWAQMGKELVQL

YPSFGESIKELNGYLSALGDDGPTWSLMEELVRGKKTSRLSQAEFAQPCTTALQIALVDLLRQSGLTPDAVVGHSSGEIA

GAYASGALTAREAILAAYYRGKAMPWVEKALVAEGTQGGMAAIGLGSEQVMPYLTKGVVVGCENSPDSTTLTGDKSALDI

VMSRINEAHAGVLVRELKVDRAYHSHHMRIAAPHYLASLKAQGIEPQAPSVPFHSSVTGEVISNAEDLGPQYWIDNLVSR

VRFSTAVNSVLSSSKSPKTFVEVGPHSTLAGPIRQILQAASAQADYVNVMKRGEDGSRAYLQAMGELWSLHVPLDLSAIV

DKGDFLTDLPLYPWHYEESLWAESRLSKDYRSRKFRHHELLGSRIIESTDHSPAWRNILRLESVPWIRDHEVFGDIVLPG

VGYIAMAGEAIRQLTGADDFSVQRVHIRVALALEHDSKIEVVTQLERVALTNSLDSPWYNFTISSFRKAADGGAGSWIKH

IFGQVRGGSDFQPGAAPDVSPLQRAVSEKGWYRKFRASGLDYGPRFQSLANITADPSTSEIVANITNDVRDAESYYPIHP

GALDCMAQALVLALCGGLTRNLGSMAVPTYIDELYCRPSAIKDMSIRVKATERRRNAYTGDLTAVCDGQIAVQAKGFRLT

VVDNTTEADASSNLGARFRHGAVHLEWKPDINFANVDMLFSPDLPPDQDFHLLDQYLALTSIEVAERTRNMKPAQDFMAL

FQKWTADFAAETQLEALFDASEKDILRNDSAYRNKVMDKTYVQLMDCQTRAAAEAIGRIVKDIEGLMNGTTDGLDILMDN

KLLHDVYDGMQLTEMGSFFDLVAHKKPNLRVLEIGAGTGGTTAKVLPLLQSAYGERMYLSYTYTDISPGFFPPAQERFRE

FPGLEYRVLDISQDPAGQGFENDLESYDLIIATNVLHATERILDTLKNVRKLCHPRGRLFLQELSPVSKWPNMIMGVLPG

WWLGAEDGRPDEPYMDEQRWDKELTSAGFCGAETVFFDGRLCLNIVTTPKEMDAKRPRVTLLTPGQVSESSEPKVRAVSE

QLQGAGYSVVVHPFGQAVLDLGMDEDVVALLDVDKPFFHDLSESDLHGFQAFLAHAKGRRCGLLWVTGASQAGCTDPRYA

PVIGVARVLRLELGMDFATLEMGGQTEDSSLKIDAPTVARVMGEFRRRDRNAMESDIDTTCEAEWVHSAGTTLAGRYHYL

DMDAELKKPAPSNGTETAVSAPEQKTYLKLDTWRPGLLDALYWKRLPVEELAPDEVRVEVRAVGLNFKDVLVAIGIIAEP

VKIARGMGYDCSGVVTAVGRDVTKHRIGDRVAVCESGTFSTSLNVLEQLCATLPDDMSFEEGATMPLVYATASYCLLDAA

QLAKGMTVLIHAAAGGVGLAAIQLCNMVGCEVYCTVGSDEKVQYLMEHCGIPRDHIFNSRDTSFLPGLMAMTDNRGVDVV

LNSLSGELLHASWKCVAEGGAFVEIGRRDFIGQAKLAMEPFETNRSFIGFDLGAMRNQRPDTIARLMDRMMKQAKEGRIK

PILPMKIFDAAQISDPFRLMQKAQQIGKMVISMPASADTELSSEAAHKPFCARSDAAYLLTGGLGGLGKSISTRLAERGA

RHFVFLSRSAEKPEHDAFFKEMESLDCTSTRVSGDVVNYDDVVRAVKAAGRPIAGALHAPLVLQDNSFLDMSFDEWNAPF

RPKVEGAWNLHKALLSEKLEFFFLFSSFGCMFGQWGQSNYSASNTFIDAFVGYRHSLGLPASTVNIGVMGDVGWVADNPE

ALEKLQAAASHVSQESDFLDCAELMFMRPGPTPQTDPAHFVQHSQVGFGLRTTLPILAPNNRVVFRKDPRMLVYRNLEAD

AGWSAAATTGAAGADPADEELTRFLQAASVNMAVLRGEEATRFIAQNVGRTLFGFMLRSEDDFDVLTPMADLGIDSLVSI

ELRNWITKRLVVEVNVLEITRAGSLLELGGLLQTRMVEKYQARR

>Fuseq1|405498

IDQKDVHEPIAIIGMGMRLPGGVHNAEAYWDLLVNGKDGRCQVPKDRYNIDTWYGPGRVTHMGTKYGHFLEELNLANIDP

NFWSFTKQEAELLDPQQRLFLEVVYEAFENSGTTKWRGKDVGVYVGALGDDWGEMEMQDGQDLNPTRTYVYGDYIIANRA

SYEFDLKGPSMVVRTACSSSMIALHLACQDILSGDCADGAIVGGINLIITPRTTVALTEQGVLSEDGRCKTFDADADGYA

RGEGVSAIYIKKLSDAVRDRDPIRAVIRSTCAAADGKTAGFTLPNPESHEKLMRRGHRLAGISDFSKTAMVECHGTGTAV

GDPREVSAVANVWGDHGIYIGSVKPNIGHGEGASGLSSIIKMTLALENKTIPPNINFKTPNPKSNVFHVILESAASFGLG

QKPESKLQGLGDEDFGWQLFTFSAKHPKALEMSAQRHEEYLTAHPDSLSNMAYTLNNKRAVHSHRAFCVTDGLDSFEISK

IVKPALGSKCDLVFVFTGQGAQWAGMGLELLRTDGIFKDTVDYLDAELSRLSDGPNWNMRDEILATEKTSRLSEAEISQP

CCTAIQIALVELLRSWGISPDAVVGHSSGEIAAAYACGAISAQDAIRVAYYRGNSTIALKSIRKGGMAAIGLGREDVEQY

LQPGVTIGCDNSPASVTLTGDADVLERVMETIRTSDPSVLVRPLRVECAYHSEHMKLVAQDYAARLGTVNASQPKVPFYS

SVTRGINSDLSTSYWTENLVSPVLFKGAIQTALQSHDNLTFVEIGPHSALAGPIRQTIQSEIKSNAGYISTLVRKKDAHL

SILSTAGNLWLAGTPHLDLSAVNLHGRPEGGELLTDLPTYSWNYDGEYWLENRLSRDWRFRKFDHHELLGARVLESADAG

PAWRCKLRVQDAPWLSDHDILGDIIFPGAAYLCMAGEAIKQLRLGSHDYSLRRVTLASALVLHGDPVELVTTLTPVRLTN

AADSDWYSFTISSFTPNTNTWTKHVSGQVRSGRTSVPDTPKIEPLPRKVPTSVMYNVWRRFGMNYGGRFRGLDDISAHTT

EKRAVGTIYEKCLPEEGELYSIHPASIDAAFHLSNVCLCHGLGRNFRTPSVPKYIEEMYVGTPEGPIRVVGDAANKGRGG

SMSNLVGVSNGKVVLSWTGLELSPLSDGSDVVDDDPHAAAVLEWKTDADFIDATRLLRSLKKDPDDEQHRLVDTMGLACI

IESRHQLAGLETSQWHLVKFRDWMDIPYSEAVEGRYPHVPNCVEIATMSSEERQKLIRDYLAASEGTEAEAVAISLYRIF

DNSVRFFTGEADPLEVLMADNILMRMYDFTNNADHLHFLTLLGHKKPTMRVLEIGAGTGGTTATILPALRSDQGERMYGT

YVYSDISAGFFMGAKERFRDYQAIEYSVLDITQDPISQGFEEGSFDLIVSSNCLHATPDLAKTLSNVRRLLHPEGRLFLM

ELSPESSKSVNYVMGPLIGWWLSEDGRENEPYVSAGIWHEKLLQAGFSGVEAYAFDGNMSNSIIARPVQPPCEQLKRVSI

IANDLDHPAVAEVTKYLQEIGLDLDLFPLGQVLTPGQPAVFLMDLEAPFLANITEEQFNSFKRTLFSVQDVPLCWVTGAC

QIGCKNPDYALVNGMARSIRQETGIDLVTFELEVFDGSAWRALSDLLETFPSRVTDGEVDTDFESEYAFHAGTIQVGRMH

WINVNSELQDKRPEVRMETLVIDKPGIIKTLHWKQKTSPVLKAEDWVQVDTRAVGLNFKDVLIAMGIVEATNDGLAADDF

GFEGAGVVTRVGSGVQHLVVGDRVAFSSTGCFSTSQTMPEIYCTKIHESLTFEEAATMPCVFGTAMYGLVDLARLEAEQS

VLIHSACGGVGQAAIQIAKMIGAEVFCTVGSEEKIEYLTSVFGIPRDHIFNSRDSSFRQGILKATYSRGVDVVLNSLSGE

LLHESWNCVAKFGTMVEIGKRDFIGKAELAMDRFENNRTFVGLDHTELWAHKPKVASRVLNKIMELCGQGKLGPIVPIKT

FEAARVEEAFRYMQKGQHIGKIVVTVPESRATHTLEAEPARRELILRDDRTYVFAGGLGGLGQSIATYLAEKGARHLLFF

SRSATQFAESNPAFFEELESLGCSTQVVSGNINNRADVEKAVASATNPVAGVLQAAMVLQDANFVDMTFDAWQTAVLPKV

LGTRNFEEALKKQEEPLDFFFLFSSVSGTAGQIGQANYAAGNTFMDAYVQYRHGQGLACSTLAIGIMEDVGFLARERHLL

EALRATSLHFLHEQDLLDSLELMLGPRASLANSTSDRSTTAAEKPDEYTRLTRGYINDSHVVVGLRSKLPLLSPMNRTGW

KKSPRLLVYRNIENQAEIKSGPATDGGLKEFLSSCGKTPELLEADATADFLAHEIGTTLFNFMMRSDEEPDLTVPLASAG

VDSLVSIELRNWFRQKVGVPFTVVEIVGAASIADLGKMTAEKLAEK

>FusspF11_1|916629

MEFSSQPNGHSRYTNGHTNGQSEYTNGYTNGHTNGHTNGRTKGYANGHASASIGKPLYEPIAICGMGMRLPGSINDSQAF

WDLLQNKRNGRCKVPKERYDVDTWFGPGKSGHVASKFGYFLDHVDLAKMDTSFWSATKDECASMDPQQRLLLEVAYEALQ

TAGQKSSELRGRKIGVFVGSFEGDWLELDQRDVENFSRHRQSGSGDYMAANRIHYEFGFMGPSTVIRSACSSSMIALHDA

CTAIIAGECEGAIVGGCNLILSPRMTATMQELGVASPSGYCHTFDADADGYARGEAVSAVYIKKLSDAIRDGDPVRSVIR

STCFNSGGRAATMSAPVAAAHEALMRRSHELAGITDFSRTAMVECHGTGTPVGDPIEVQAIANVFGDHGIYIGSVKTNLG

HGEGASAMSSLIKMTLALEHKIIPANLNFKTPNPSIPFKSRKLTVPLESTPWPKDRDEVVGINSFGVGGSNAHAVLASAD

SFGAGMAQESAAEKTIVSWKPALLLFSAKHADALKKIIENQQAYHVAHPNRLTNVAYSLAIKRDAFDHRAFAVADGVDDW

SAKFSTRTSAPREPPKLVFVFSGQGAQWAQMGKELVQLYPSFGESIKELDGYLSALGDDGPTWSLMEELMRGKKTSRLSQ

AEFAQPCTTALQIALVDLLRQSGLTPDAVVGHSSGEIAGAYASGALTAREAILAAYYRGKAMPWVEKALVAEGTQGGMAA

IGLGSEQVMPYLTKGVVVGCENSPDSTTLTGDKSALDIVMSRINEAHPSVLVRELKVDRAYHSHHMRIGAPHYLASLKAQ

GIEPQPPSVPFHSSVTGEVISNAEDLRPQYWIDNLVSRVRFSTAVNSVLSSSKSPKTFVEVGPHSTLAGPIRQILQAASA

QADYVNVMKRGEDGSRAYLQAMGELWSLHVPLDLSAIVDKGDFLTDLPLYPWHYEESLWAESRLSKDYRSRKFRHHELLG

SRIIESTDHSPAWRNILRLESVPWIRDHEVFGDVVLPGVGYIAMAGEAIRQLTGADDFSVQRVHIKVALALEHDSKIEVV

TQLERVALTNSLDSPWYNFTISSFRKAADGGAGSWIKHIFGQVRGGSDFQPGAAPDVSPLQRAVSEKGWYRKFRASGLDY

GPRFQSLANITADPSTSEIVANITNDVRDAESYYPIHPGALDCMAQALVLALCGGLTRNLGSMAVPTYIDELYCRPSAIK

DMSIRVKATECRRNAYTGDLTAVCDGQIAVQAKGFRLTVVDNTTKADASSNLGARFRHGAVHLEWKPDINFANVDMLFSP

DLPPDQDFHLLDQYLALTSIEVAERTRNMKPAQDFMALFQKWTADFAAETQLEALFDASEKDILRNDSAYRNKVMDKTYV

QLMDCQTRAAAEAIGRIVKDIEGLMNGTTDGLDILMDNKLLHDVYDGMQLTEMGSFFDLVAHKKPNLRVLEIGAGTGGTT

AKVLPLLQSAYGERMYLSYTYTDISPGFFPPAQERFREFPGLEYRVLDISQDPAGQGFENDLESYDLIIATNVLHATERI

LDTLKNVRKLCHPRGRLFLQELSPVSKWPNMIMGVLPGWWLGAEDGRPDEPYMDEQRWDKELTSAGFCGAETVFFDGRLC

LNIVTTPKEMDAKRPRVTLLTPGQVSESSEPKVRAVSEQLQGAGYPVVVHPFGQAVLDLGMDEDVVALLDVDKPFFHNLS

ESDLHGFQAFLAYAKGRRCGLLWVTGASQAGCTDPRYAPVIGVARVLRLELGMDFATLEMGGQTEDSSLKIDAPTVARVM

GEFRRRDSNAMESDIDTTCEAEWVYSAGTTLVGRYHYLDMDAELKKPAPSSGTETAVSAPEQKTYLKLDTWRPGLLDALY

WKRLPVEELAPDEVRVEVRAVGLNFKDVLVAIGIIAEPVKIARGMGYDCTGVVTAVGRDVTKHRIGDRVAVCESGTFSTS

LNVLEQLCATLPDDMSFEEGATMPLVYATASYCLLDAAQLAKGMTVLIHAAAGGVGLAAIQLCNMVGCEVYCTVGSDEKV

QYLMEHCGIPRDHIFNSRDTSFLPGLMAMTDNRGVDVVLNSLSGELLHASWKCVAEGGAFVEIGRRDFIGQAKLAMEPFE

TNRSFIGFDLGAMRNQRPDTIARLMDRMMKQAKEGRIKPILPMKIFDAAQISDPFRLMQKAQQIGKMVISMPASADTELS

SEAIHKPFRARSDAAYLLTGGLGGLGKSISTRLAERGARHFVFLSRSAEKPEHDAFFKEMESLDCTSTRVSGDVVNYDDV

VRAVKAAGRPIAGALHAPLVLQDNSFLDMSFDEWNAPFRPKVEGAWNLHKALLSEKLEFFFLFSSFGCMFGQWGQSNYSA

SNTFIDAFVGYRHSLGLPASTVNIGVMGDVGWVADNPEALEKLQAAASHVSQESDFLDCAELMFMRPGPTPQTDPAHFVQ

HSQVGFGLRTTLPILAPNNRVVFRKDPRMLVYRNLEADAGWSAAATTGAAGADPADEELTRFLQAASVNMAVLRGEEATR

FIAQNVGRTLFGFMLRSEDDFDVLTPMADLGIDSLVSIELRNWITKRLVVEVNVLEITRAGSLLELGGLLQTRMVERYQA

RR

>FusspF23_1|389828

MEFSSQPNGHSRASIGKPLYEPIAICGMGMRLPGSINDSQAFWDLLQNKRNGRCKVPKERYDVDTWFGPGKSGHVASKFG

YFLDHVDLAKMDTSFWSPTKDECASMDPQQRLLLEVAYEALQTAGQKSSELRGRKIGVFVGSFEGDWLELDQRDVENFSR

HRQSGSGDYMAANRIHYEFGFMGPSTVIRSACSSSMIALHDACTAIIAGECEGAIVGGCNLILSPRMTATMQELGVASPS

GYCHTFDADADGYARGEAVSAVYIKKLSDAIRDGDPVRSVIRSTCFNSGGRAATMSAPVAAAHEALMRRSHELAGITDFS

RTAMVECHGTGTPVGDPIEVEAVANVFGDHGIYIGSVKTNLGHGEGASAMSSLIKMTLALEHKIIPANLNFKTPNPSIPF

KSRKLTVPLESTPWPKDRDEVVGINSFGVGGSNAHAVLASADSFGAGMAQESAAEKTTVSLKPALLLFSAKHADALKKMI

ENQQAYHVAHPNRLTNVAYSLAMKRDAFDHRAFAVADGVDDWSAKFSTRTSAPREPPKLVFVFSGQGAQWAQMGKELVQL

YPSFGESIKELDGYLSALGDDGPTWSLMEELVRGKKTSRLSQAEFAQPCTTALQIALVDLLRQSGLTPDAVVGHSSGEIA

GAYASGALTAREAILAAYYRGKAMPWVEKALVAEGIQGGMAAIGLGSEQVMPYLTKGVVVGCENSPDSTTLTGDKSALDI

VMSRINEAHPGVLVRELKVDRAYHSHHMRIGAPHYLASLKAQGIEPQPPSVPFHSSVTGEVISNAEDLGPQYWIDNLVSR

VRFSTAVNSVLSSSKSPKTFVEVGPHSTLAGPIRQILQAASAQADYVNVMKRGEDGSRAYLQAMGELWSLHVPLDLSAIV

DKGDFLTDLPLYPWHYEESLWAESRLSKDYRSRKFRHHELLGSRIIESTDHSPAWRNILRLESVPWIRDHEVFGDVVLPG

VGYIAMAGEAIRQLTGADDFSVQRVHIKVALALEHDSKIEVVTQLERVALTNSLDSPWYNFTISSFRKAADGGAGSWIKH

IFGQVRGGSDFQPGAAPDVSPLQRAVSEKGWYRKFRASGLDYGPRFQSLANITADPSTSEIVANITNDVRDAESYYPIHP

GALDCMAQALVLALCGGLTRNLGSMAVPTYIDELYCRPSAIKDMSIRVKATERRRNAYTGDLTAVCDGQIAVQAKGFRLT

VVDNTTEADASSNLGARFRHGAVHLEWKPDINFANVDMLFSPDLPPDQDFHLLDQYLALTSIEVAERTRNMKPAQDFMAL

FQKWTADFAAETQLEALFDASEKDILRNDSAYRNKVMDKTYVQLMDCQTRAAAEAIGRIVKDIEGLMNGTTDGLDILMDN

KLLHDVYDGMQLTEMGSFFDLVAHKKPNLRVLEIGAGTGGTTAKVLPLLQSAYGERMYLSYTYTDISPGFFPPAQERFRE

FPGLEYRVLDISQDPAGQGFENDLESYDLIIATNVLHATERILDTLKNVRKLCHPRGRLFLQELSPVSKWPNMIMGVLPG

WWLGAEDGRPDEPYMDEQRWDKELTSAGFCGAETVFFDGRLCLNIVTTPKEMDAKRPRVTLLTPGQVSESSEPKVRAVSE

QLQGAGYPVVVHPFGQAVLDLGMDEDVVALLDVDKPFFHDLSESDLHGFQAFLAHAKGRRCGLLWVTGASQAGCTDPRYA

PVIGVARVLRLELGMDFATLEMGGQTEDSSLKIDAPTVARVMGEFRRRDRNAMESDIDTTCEAEWVHSAGTTLAGRYHYL

DMDAELKKPAPSSGTETAVSAPEQKTYLKLDTWRPGLLDALYWKRLPVEELAPDEVRVEVRAVGLNFKDVLVAIGIIAEP

VKIARGMGYDCSGVVTAVGRDVTKHRIGDRVAVCESGTFSTSLNVLEQLCATLPDDMSFEEGATMPLVYATASYCLLDAA

QLAKGMTVLIHAAAGGVGLAAIQLCDMVGCEVYCTVGSDEKVQYLMEHCGIPRDHIFNSRDTSFLPGLMAMTDNRGVDVV

LNSLSGELLHASWKCVAEGGAFVEIGRRDFIGQAKLAMEPFETNRSFIGFDLGAMRNQRPDTIARLMDRMMKQAKEGRIK

PILPMKIFDAAQISDPFRLMQKAQQIGKMVISMPASADTELSSEATHKPFRARSDAAYLLTGGLGGLGKSISTWLAERGA

RHFVFLSRSAEKPEHDAFFKEMESLDCTSTRVSGDVVNYDDVVRAVKAAGRPIAGALHAPLVLQDNSFLDMSFDEWNAPF

RPKVEGAWNLHKALLSEKLEFFFLFSSFGCMFGQWGQSNYSASNTFIDAFVGYRHSLGLPASTVNIGVMGDVGWVADNPE

ALEKLQAAASHVSQESDFLDCAELMFMRPGPTPQTDPAHFVQHSQVGFGLRTTLPILAPNNRVVFRKDPRMLVYRNLEAD

AGWSAAATTGAAGADPADEELTRFLQAASVNMAVLRGEEATRFIAQNVGRTLFGFMLRSEDDFDVLTPMADLGIDSLVSI

ELRNWITKRLVVEVNVLEITRAGSLLELGGLLQTRMVERYQARR

>Gloci1|1752494

MGMRLPGGVNDPDAFWDMLVNKRDGRCPVPKDRYNAETWHAPGKKRHVPSDHGYFLDSSIDIKNADASFWSMTKKELEIL

DPQQRLSLEVVYETLQRAGQKPSELRGRKIGVWVGSFGGDRAELDAADPQTVHPYNLLNGFDFMPADRIHYEFGFMGPSV

TVRTACSSSLLGLHQACYSLIHGDCEAAVVAGTSIIYSPTLTATMNDHNVLSPSGTCKTFDAEADGFVRGEAVVAVYVKK

LSDAVRDGDPICSVVLSTASNSSGKSSTMTAPNPDAQEDLIRRAHEVAGIKDYSRTAMMECHGTGTPVGDPLECEAVGRV

FGDHGGIYIGSVKTNIGHVEGAAGLASVLKMTLALQNDMIPPNANFKSPNPKIPFDRYQLKVPTQAIPWPQGKDRVVGVG

SYGVGGSNAYALLASVDHVGIKDDRKRTKTTKPLPIGLECPKLLLFSAKHPQALEKMVKRHQAYNLAHPDRLSDVAYTLA

KKRELLSYRACCVSNVIDNFPTLKPPRHGFYDPAHLVFVFTGQGAQWAQMGKSLVENIPSFKLSLEAMDSLLQQLPDSPT

WNLIDEMLAPKRTSRINNGQVSQVCCAALQLCLVDLLKTYNIKPAAVLGHSSGEIAAAYACGAITQREAIIIAYYRGKVL

GAVEDSVGGMAAIGMGKAQVTPYLRPGVLVGCENSPNSVTLTGDKNSLDLVVTEIKKAHPDVLARALQVDRAYHSHHMQA

VASQYVDLLRPYLQPRDPKTLFFSSVTNQLICKGTELGPDYWIQNLVSPVLFNGAVIKALHTLNSEKVFLEIGPHSALAG

PIRQILGAEKINAEYIATLIRGQDAHQEVVRAVGELWLRNHPVALDRVFKEGGILTDLPSYPWHYEGPLWHESRLSEEYR

LRQFRHHELLGSRVLQSTNTSPAWRNLLRLEDVPWIAEHEVEGTIILPGVSYLCMAGEAARQLTGKTNFICKQVHFSAAL

PMTYESQTEIITQLNQIRLTDSIDSGWYDFTISSYQDGGWVKHAYGKVRGDGELSNISLEQFDAKDFSSVFSRVCSSASW

YRKFRSLGLEYGPRFSGMDIAADPTKPQIKGTLTLDLLPGEEKYYSIHSGALDRLVQSLYVAVARGLTRNCNSLALVAYV

HEFAFTAPLVGAQELEFYAKVAEQRPGSFLGSVDAAPDGGSVVVSSKGWQLSRISGSDGANEVSNPHGAAELEWREDIEF

IDPSSLIRPAIDVSKAELYRLLDRFNILSLTRTLDSIRHIAHPTREHLNRYRKWLEDTVTGLASGSLKCPGVPDAEYLVS

LASKDRDVLLSSLFKDLEKSEVNAPAAAIHRISSSCEGIFNGDVSELELLLADGVLQNVYNCLLLDTDSSRLLSLIGHKK

PNLRVLEIGAGTGGATASTLRSLTSSHGQRIYQSYTYTDISSGFFADAKKRFEEYGGIEFAILDISKDPLEQGFQAESYD

LIIAWNVIHATPNLHESLSNIRKLIHPEGRFLLQEIFPITPWINHCFGVIPGWWLGEADDRVSTPHVSFERWKKELSDSG

FGDIANSFDGYTNNNIICRPSQPMPSSTRVTLLKHRGQDTKQLFETLRRASYEVDEYTLEDSEASLTPEQDVISVLDLLS

PCFAGLTEEKLAHFQRFVKNAHDGDCGILWLTGQCQVGEKIQPEYAPLFGLSRVLRTELGLDFATLELESFSPAAIAHVF

PKVYSQFGKRVKNDPDSNPEYEWAYMDDKILISRYHHINLTAESQVLPESIGVRKLSQRKPGLTDTLYWKPIITPALNKD

EVKIEVKAVGMNYKDVLIAQSIITDTAAIQDGLGLECAGIVLEVGPDVDRVKVGDRVVAISSGSFTNIKVTSQQLCCRIP

DPMSFEDAAVIPVAYCTAFHALFTQGKAIKGMSILIHSATGGVGLAALQLAQMLEADIYCTVGSQSKRDFLIEKLGIPPG

KIFNSRDSSFLPGIRAATKGLGVDLVLNQLSGELLHASWQCVAEFGTFVELGRRDFLGHAKLAMDQFESNRTFCGIDLAH

LWSRKPRLVGDMLERVIDLWTQGHIEPYITSTRSAAEIFQPFRQMQQSQHIGKIVVTLPEEAEYQLLPTEPIHKTVELRH

DRSYLFTGGLGSLGAVISSWLVEKGAKEIIFLSRSAGRLPEHVSLAKELACLGCNATFVSGNVAKYDDVVRAIKTAKMPI

GGVLHAAMALRDAGFLSMSWSEWLTASQPKIDGTRNIHEALLDRQPDVPLEFFFLFSSTAATGGWWGQANYHAGNSYMES

FAGYRRNLGLAASVLNVGFISDVGYVADRPEAADLAKATGQWFNTEAELLNCIERMLMEPPTGNGASAASCHVQPSLLAM

GMRSTMPLTSKSCRVPWKRDRRMLALRNLDSDDSNIPSGSDSMTKDELSHAIRELSSNIVSLQSGETTEFLAAHIGKTLC

KFQLRQDTDLDFHAPLTDLGLDSLVGIEVRAWIRQWMGVDLATLEITGSKNLEQLATAVQKRMIQRSTLRHEAQTLA

>Lepor2|720187

MPSNTNGYSIPTTNGSNAHSTHHGNGINGHSTHRTNGTNGVSTHNTSGTNGTNPHGVPFPQDPKYRAEPMAICGMGICLP

GGVTTGDAFWDMIVNKRSGRCVVPKDRYNVESWYAPGKKGHSMTKKELEAMDPQQRLMLEVVYETLQNAGQKPSELRGRN

IGICVGSFSGDRADLDSRDTQTVHPYNLLNSFNFVPADRVHYEFGFMGPSVTIRTACSSSLVGLHQACQALQAGDCKAAV

VAGSSIIYSPSLTIKMNEQNVLSPTGTCRTFSADADGFRLQDAIRDGDKIRSVVLSTATNSSGKSSTMTAPSAVAQEALI

RRAHDIAGISDLSRTAMMECHGTGTLIGDPIEAQAVANVWGDLDGIYIGSVKTNIGHMTLALENDTIPPNINFTSPNPKI

PFEKCKLTVPTDPKPWPKDKDRVVGVGSYGVGGSNAYALLASADHLKALRGNTQTKVVTDDYDIEIPKLLLFSAKHPKAL

DRMVQNHKAYYSVNSDRLGDIAYSLAIKRENLSHRLCAVANGINDWSLFDSSRHGTFEPGKSVFVFSGQGSQWAQMGREL

IKNVPSFKKSIEDMDRILHGLIDAPKWELKGKLTSQILAAKKSSFINEAEISQPCCTALQVALVDLLNTYNIKPEVVLGH

SSGEIAAAYACGAISSREAIIIAYYRGKVMLDIDASVGGMSAIGLGLKEVEPFLCPGVLVGCENSPKSVTLTGDKDALAE

VSKAIKEAHPDTLARALLVNRAYHSHHMETVASRYLELMSPHLKPCDPSLPFFSSVTNKAISNGQGFGAAYWVQNLISPV

KFNSALSKVINSSMGDNVFLEVGPHAALAGPIRQIMAAENIRAEYISVLTRGKDSHEEFLRTLGQLWMQNYPVSLDHLFA

EGQFLHDLPPYPWNYEEPLWYESRLSKEYRLREFPHHELLGTRIVESTSANPGWRNVLRLESVPWIAEHEIEGDIVLPGI

SDILMAGEAIRQITSRSDFTCQQVHIKSALLLAEDDEVEVITQLNRIDLTDAMKHASEQVRGGGKESANSMPREKASKTH

PRVCSSKAWYRKFRSFGLNYGPRFTSLKDISADPLEPQLAASLTVDLRPGEERYYSIHPGTLDGLVQGLFPAIACGQTKK

FDQLSLTTYVEEFYMQAPPSGIKDLRYLVNITEQRTTASLGDAAIVAIPEDGGKPVTVVKSRGWQVSRLNDSSETSDDQN

PHAAATLEWREDINLLAKPSSLIKSVRNKSKEDAHIILDRFNILCMVQSRERLRPELINMEADARNKLIEELYVQLKESP

VAAEAAAIHRVSFSCDKIFEGESELELLLEGRILHNLYDSLLVDSDSSGFVSLLAHKKPNLRVLEIGAGTGGATSTVLRA

LKSSSGEQVFASYTYTDISAGFFADAMERFKEYSGLEFVVLDITKDPLEQGLETGSFDLIVAWNVLHATHNLYQTLTNVR

KLIHPHGYLLLQELDPLTKWINHAFGVIAGWWLGGPDGRPSQPHVDFARWKEELTNADFKDINSMHDGYIDNNISKASDD

SSPFQAGLQKYRGSLQASGYEVDEYTLETQTSKLRPGQDVVSVLDIVSPFIANAHEESYAQFQRFISVAKENACGILWIT

GSCQVGHANPEYGPILGLARVLRTELELDFAVLELDDFRSGVDVIPKVFGDFQKRISDEDHVNPEYEWAHVDGKTLVSRY

HHTKVTRKPKALPTELTARKLEQKRPGLTDTLYWKAVAPQELKGDEVRAEIKAVGMNYKDILIAQNIIDETAAISTGLGS

ECSGIITEIGSDVSNSSIGDRVAIVSSGSFTTSKIVPASLCVKIPDEMNFEDAAVLPVAYCTAIHGIVDLGRLSEGMSVL

IHSAAGGVGIAAIQLAKMLGAEIFCTVSSQEKIDFLVSEFSIPKDHIFNSRSPSFLPSIMAATSNRGVDVILNSLSGELL

HASWKCLAEFGTFVEIGRRDFLGHGKLAMQNFESNRTFVGVDLTHLWLQKPKIVGKLLERAVKFWTEGFLRPIISSKLPA

KYVVGAFRLMQKSQHIGKFVVDMPVDPIGELPVETVYETLQLRDDRAYLFAGGLGSLGRAIATWLVEKGATEIVFLSRSA

GTIPSHLHFVEELAVLGCKAQLIAGDVGNYKDVLRAVESASTPIGGIFHAAMVLRDAQFLSMSYADWLAGSQPKIQGAWN

LHNALLSQQPSIPVDFFFLFSSTAATGGWWGQSNYQAGNSFLESFAAYREQLGFAASVLNVGFISDTGYVADRPEAADSA

RATGQKFNTESELLDCTEFQLMSKAPLPDENDTDENNNNNSLNANQGTTTSLVQHSLLAMGMRSTVPITSSTCRLPWRKD

RRMLAFRNAETSTAAGTSSGTSNGSTTNDELTRFIREVSSNGVLLQSPQTTTFLAHEIGTTLLQLLLRPETDLDLEEPLS

AIWIDSLVSLEVRAWIRKWMGVDLVTLEIMKARNLMALAVAVQGKMVARYNARA

>Lopnu1|662160

MTADSGKPETVTIAATEAVAPPRFSDMNGLPNGEHVTNGDEKLKPPVEPVAICGMAMRLPGGVRNAEDFWDMLYNKRSGR

CLVPKNRYNADAWYGPGKIGHVASKYGYFLDDVELHHTDASFWTMSKQEIEAMDPQQRLTLEMVYECLQNAGQKPSALRG

ARVGVYLGTFEGDWLELDGRDPQHYHMYRLTGYGDYMSANRIHYEFGFTGPSVTIRTACSSSLTGLHDACQALFSGECDS

AVVACANLIFSPRTTVTMQEQGVMSLTGYCKTFDADADGYARGEAVSAIYVKKLSDAIRDGDPIRSIVRSTAINAGGRAS

TLTAPNTAAHEYLIRRGHEVAGITDLSKTAMIECHGTGTAVGDPIETNAVANVFGDWGCYIGSVKTNLGHSEGASGLSSI

IKMTLALEKSTIPPNLNFKTPNPNIPFESRKLKVPTEPLPWPTDKKELVGVNSFGIGGSNAHVLLESAKSYGIPQAHLNT

GSEDNAELVPRLLYFSAKHPESLKTTIADHESYFLAHPESLDDMSYSLAAKRELLPWRAFSVTDGEDSWDTSPPSRAGDK

SPTQKLFVFTGQGAQYAQMGKALITRMPRFRNSIMALDEALLSAKGPPEWKLLTEILRPKKTSRLSRAEFSQPCTTAIQI

ALVDLLSSYGIHPSAVVGHSSGEIAAAYAAGALTASDAIITAYQRGRIMSLLDTDRPGGMAAVGLGRDEAVPFLKPGVLI

GCENSPESVTLTGDKKPLEEVLCSIRKAHPDVLARTLHVDRAYHSHHMETVADRYLASLPEVMQSAVPQISFYSTVYSKL

IRAKGELGSQYWVQNLISEVRFSSSISNIMRDIPQPKVFIELGPHSALAGPLRQILRHHKSNDEYVSTLTRGGNAYVDIL

KAAGSLWLRNVNFDVMATIPQGKFLTNLPLYPWHYDEVLWHESRLSKDWRLRPFPHHDLLGSRILESTDSSPSWRNILRL

DVVPWIKEHEVAGDIVFPGVGYLCMAGEAIRQLTGARDFTAHQVHIKNALVMHQGVDVELITTLHRESLTSTHDSDWYHF

TVCSLDRDNWIKHIFGRIRAGTEYTREASNPKQLPRILSRRSWYRKMRQQGLEYGPRFMGLRDMSADPVKRQLVASIESV

VPSDESLYAIHPTIMDCFMQALVPAHFNGLTRRFDMLGIPTYVEELYVGPARGEIQIQATADDSPKGALSGHVIGLSEGH

VVLEFKGVSTSAIADTNELEGDRCAAVELEWKKDINCLTDISKLMKLSADRTALHQILDEFAVLCMLECDAAMRFVEPHT

DYLCNYKAWLAETVSEIAQGQYHGVENAAQLSSLDPSARRLRIASLLSELERTDARAPSLAIHRIFDAAAGILSGDINEL

ELLLADNVLHQLYDFMQNTDYAELIELIGHNKPNMRVLEIGAGTGGTTATILPLLQSAYSERMYLSYVYSDVSAGFFMTA

KDRFKDYPGVQYQTLDISQDPLEQGFEPGTFDLVIACNVLHATPSLQQTLKNVRTLMHPRGRLLLQELHPATKWINFIMG

VLPGWWLGQVDGRTPEPYVTPERWDNELRSAGFDGTSVSFPDGYLINNIISSPTVADRNVPRVSILHNGSQESDLLRIQN

LLRSSGVDTGVCTLDDVPIAGQDIVSILDLAQPFLHDLDEQTFKRFRKYVSSLHKSGILWITGACQINCKDPRFSMVIGL

SRVLRSEMSLDFAVLEMDSFDDQAIETVPTVLDQFLGRLDEDHVNATSEWAVVNGEIFTSRYHFIHVEKELEATPSRSSH

KKLEQRRPGMLDTLYWKSSDDGPVPQEREVAIDVCAVGLNFKDVLIASGVVTEKSAIGRGFGFEGSGIVAAVGAGVSTCS

VGDRVLFCCSGSFATRTVIDERLACVMPDTLTFEEAATIPTVYGTAIHCLVTAGHLRQGESILIHAATGGVGIAALYIAR

MIGAEIYCTVGSDAKRQFLTDTFNIPTDRIFNSRDSSFLPAVLNATSGRGVDVVLNSLSGGLLHASWNCVAEFGRFVEIG

RRDFVGQGKLAMEQFLENRSFVGFDMSAVIDKRPQMVQDLMVTAMRFHTQGYIKPISPMHVYSTTNVQEPLRIMQKANHI

GKNVVLMPDNFETIEAEPRRSRLQLRQDRAYLFVGGFGGIGKAMSTWLAERGVKQLIFLSRLAGEISSEDPFRRELHALG

CKTTVISGSVTNYEDVLKAVHATELPIGGVLQASMVLQDGNFVDMGWDQWQAAVLPKVQGTWNLHNALLTQSEPLEQFFL

FSSAGAMGGQLGQSNYNSGNTFLDAFVQYRQSLGLAASTVNIGVVEDVGYVNDNPAVLDALRATSQYLLRETELLASVEL

MLQRSDSSNVCRLHDVPPAKTRSPRFVNHAQIGTGMRSTLPILADNNRTIWRKDPRMLVYHNIEEGAYAETTATDGSGDA

VFRQFIKEASSNMTVLKNPDTATTLSREIGRTLFRFLLRGEDFDPDTAIEEPLANIGIDSLISIEVRNWIRRTLGIELPV

LEIVKSENIRQLGGKAQLKLVEKLEARM

>Melbi2|385136

MNAMGFSSQPNGHSPATSRSTANGQSEYTNGHGSAATTNGQGRAPKPLVEPIALCGMGLRLPGAVNDAQAFWDLLQNKRN

GRCKVPTTRYDVDNWYGPGKSGHVSSKFGYFLDHVDLANMDTSFWSATKDECASMDPQQRLLLEVVYEALQTAGQRSTDL

RGRKVGVFVGSFEGDWLELDQRDVENFSRHRQSGSGDYMAANRIHYEFGFMGPSAVIRSACSSSMIALHDACTAINAGEC

EAAIVGCCNLILSPRMTATMQELGVSSPSGYCHTFDAAADGYARGEAVSAVYLKKLSDALRDGDPVRSVIRSTCLNAGGR

SSTLSAPVAAAHEALIRRCHELAGISDFSRTAMMECHGTGTPVGDPIEVQAVANVFGEHGIYIGSVKTNLGHGEGASGLS

SLVKMTLALENKTIPANLNFNTPNPNIPFESCKLRVPTEPMPWPKDRDFVVGINSFGVGGSNAHALLGSAASFGAGLAEE

SASEKTTVSSKPALLLFSAKHPNALKAMIESQQAYHLAHPNRLGNVSYSLAMKRDAFDHRAFAVTDGLDDWTAKFSTRTA

PPREPQTLVFVFSGQGAQWAQMGKELIQVYPSFEESIKALDRHLHTLEDDGPSWSLLEELLRGKKTSRLSQAEFAQPCTT

ALQIALVDLLRQCGLTPDAVVGHSSGEIAGAYASGALTAREAILVAYYRGRAMPWVEKALAVDGVQGGMAAVGLGPEQVT

PYLAKGVLVGCENSPESTTLTGDKDVLDSVLRRVKEAYPDVLVRELKVDRAYHSHHMGIAAPHYLADLKARGITPQAPSV

PFHSSVTGKVIRNAQDLGPQYWIDNLISRVRFSTAVASVLSSSTRPPKTFVEVGPHSTLAGPLRQILQSANSQADYVNVM

KRGQDASRAFLQAMGELWSLHVPLNLAAIIDKGEFLTDLPLYPWHYEEPLWAESRLSKDYRFRKFPHHELLGSRVVESTD

HSPSWRNVLRLESVPWIGDHEVFGDVILPGVGYVAMAGEAIRQLTGAPDFSVQRVHIKVALALKHDSAVELVTQLDRVAL

TNSLDAAWYNFSISSHRKAPDGGAGAGAWVKHAFGQVRAGSEFQPGSPPDVRPLQRVVSEKGWYRKFRAAGLDYGPRFQG

LANTTAHPPAPELVAAITNDVRLAESYYPVHPAALDCLAQALVLALCGGLTRNLAHMAVPTYIDELYCRPPATEDMAIRV

RATERRRNAYTGDMTAVSDGQVVVQARGFRLTVVDNNDTEAEGPSSSGARFRHGAVQLEWKPDINFADVDRLFSPDLPPD

QDFEMLDRYLALCAIEVAERVRNIEPAQDHMALFSAWTTKFAAGTQLDGLFGVEENHILREDGDRRKEVMDMLYSQLMDC

GTRAAAEALGRIVEASEALMRGTTDGLGVLMDGKLLHDVYDGMQLTDMTAFLDLVAHKKPNLRVLEIGAGTGGTTAKVLP

ALQSAYGERMYLSYTYTDISPGFFPPAQERFHESPGIEYRVLDISQDPTEQGFEADLESYDLVIATNVLHATPRIQDTLR

NVRKLVHPRGRLFLQELSPVSKWPNMIMGVLPGWWLGADDDRPEEPYMDAQRWDRELTSAGFCGAQTVFFDGRLCLNIVT

TPAAPTDVVSKEQKRARVMLLVPAQVSETSEPQVRAVTAQLRGAGYRVNVHHFAQAAADLDLGPDEDVVALLDVDKPFFH

DLNEAGLRAFQGFVAHAKERRCGILWVTGGSQAGCTDPRYAPVVGVARVLRLELGVDFATLEMGGHIQDGGTGMDSHGGL

EVDAQAVARVLDEFRRRDRGAIETETDTTCEAEWVYLAGHVLTGRYHYLDIDAELKKPQPCSASGSIETAISVPEREQQK

TYLKLDTWRPGLLDALYWKRLPEEELGPEEVRVEVRAVGLNFKDVLVAIGLVAEPEKIARGMGYDCSGVVTAVGPNVTKH

RIGDRVVVCESGTFATSLNVSQLLCVGMPDDMSFEEGATMPLVFATASYCLLDAASLTKDMSVLIHAAAGGVGLAAIQLC

KMVGADIYCTVGSEDKVRHLMEHCDIPRHHIFNSRDTSFLPGLMAMTDGRGADVVLNSLSGELLHASWKCVAEGGTFVEI

GRRDFIGQAKLAMEPFESNRSFIGFDLGAMRIQRPVVIARLLDRMMEFAREGHIKPLLPMRTFDAAYVSEPIRLMQKAQH

IGKMVVTMPASADAELPSEAAHEPFRARSDATYLLTGGLGGLGRSVSTWLAERGARHFVFLSRSAAKPEHEAFFQELACL

GCTSIGVSGDCANYDDVVRAVKAAGMPICGVLHASMVLEDNSFLDMSFDQWSAPFQPKVKGAWNLHKALSSENLDFFFLF

SSFGAMFGQWGQANYSAANTFVDAFVSYRHSLGLPASTINIGVMGDVGWVAENPEALEKLQAAASHVSQESDFLECAELM

LMRPGPMQQQEQKRFVQHSQIGFGLRSTLPIASPNNRVVFRKDPRLLVYRNLEADAGWSSAPTTHGQAGADPADEELTRF

LQGAAVNMAVLRSEEATALIARNVGRTLFGFMLRPEDEFDVHAPMADVGIDSLVSIELRNWIRKRLGVEVNVLEITRAGS

LQELGGLLQTRMVEKYQARR

>Melsp1|200384

MGMRLPGGITDAQGFWDLLYNKRSGRCKVPGDRYNVDTWYGPGKIGHIACQYGYFLNNVNLGNMDTSFWSMTKNEIEALD

PQQRLTLEVTYECLQSAGQKPGELRGRRVGVYMGSFEGDWQELDGRDPQHYHQYRITGYGDYMSANRINYEFGFMGPSVT

TRTACSSSMTGFHEACHAINSGECESAVVVCSNIIYSPRTTVTLQEQSVTSPTGYCKTFDADADGYVRAEAVSALYIKKL

SDAVRDGDPVRSVVVSTCINAGGRAATLTSPNEVAQEALIRRGHKLAGRSDFSKTAMMECHGTGTKVGDPIEVSAVANVF

GQWGIYIGSVKTNLGHSEAASGLSSIMKMTLALENNTIPPNIHFNTPNPKIPWDEGKLTVPTEPQPWPAGRDQVVGINSF

GIGGSNAHVLLASAASFGLGPKDRRTVESQEADVPHILLFSAKHPEALKKMIQDHESYCNSNPSKLKDVAYSLAMKRERL

SHLGFCVANGVDDWTPSSAPPHPDSYEPPGLVFTFSGQGAQWAQMGQSLIKNVPEFRRSIEDLDKVLSELHDGPTWKLLD

QLLAPRKRSRISEAEISQPCCTAIQIAQVDLLRHYGILPAAVVGHSSGEIAAAYCSGALNASEAITIAYYRGRIMTQVDP

NSTPGGMAAIGLGADDVEQFLRPGVRIGCENSPRSVTLTGDKDVLEDVMDSIKRAQPDILVRALQVDRAYHSHHMDQVAH

RYFQLLDQKVNPQELKIPFYSSVYSRKLSTGEELGPAYWVENLVSPVRFSGTVADIVSSVTGSKTFLEVGPHSALAGPIR

QILQPLNSSDGYINVMTRGKDSHAEILRAVGELWLARHDLDLRNISGTGEFLTDLPPYPWHYEEPLWFESRLAREYRLRE

FPHHDILGSRILESTAQSPAWRNLIRLESVPWIKEHEVAGDVVLPGVAFVAMAGEAIRQLTGSTDFMARQIHIKAPLVLS

DESASEVITQLHRIQLTDSLDSDFYDWSVSSYESGHWVRHAFGHVRAGPERARDAPEIGLQPRRLSTPAWYRQMRSLGLE

YGPRFRGLDDLTAHPVAPRVTASLTHKTHEGESLYAIHPETLDCLAQSLAPAAARGLTKYFTKLAIPTYIDEFYVCPPPE

THIKIQTDITEQRHSAQIGDIIAVCEGKVVAEIKGFQLSAISQGDDDHEEVDPHAAVELEWKEDLNLIDVSALISQAEDR

SIVHHLLDKFAALCMMETGDRLQGVQPTRHHLSQFYNWLVEIKDGMTRGYFAGLRSSEVQEISELDSTSRRVLREELYRK

LQDTIASAPATAMHRITEACQGIFDGSEEELGLLLQDDVLHRIYDFSQNSEYAGFLDLLAHRKPTLKILEIGAGTGGTTN

TILPVLKSASGERLYASYTYTDISSGFFPAAKERFKEYANIDFTILDISKDPAEQGFADALGSYDLVIACNVLHATPSLK

ETLTNVRKLVHPRGRIFLQELSPATKWINFVMGVLPGWWLGAEDLRASEPYIDSKRWNEELSNAGFSRFTAVHDGYLNNN

IVCMPTPVNLREKRITLLTGNDEPGQRLRELEAALDRSGYEVNRYALRSSAGIPVNQDVLSVLDLEDPFFVSMDETAFAD

FQCLTKRAKEGQCGILWVTGACQVGKPDPRYAPVIGLARVLRTEMSLDFGVLELEDFTASSLDAVPRVMEEFQQRIIEPD

VNPETEFAHVAGKTLISRYHFIVVGDELKTVGDAETCHRKLEQRKPGLASTLYWQPKQRKGLGARHVRVAVKAVGVNFKD

LLISLGIVAEPWAVGDGMGCECSGVITEVGDNVTGLRVGDRVVVTCMGCFTTELVIPQDVCAKVPDNLPFEDAAVMMCVY

CTAIYCLLDAGRLGRGMSVLIHSAAGGVGIAAIQLAQMVGAQIYCTVSNEEKISYLHNTYGIPREHIFYSRNDRFLSDIM

RATHGRGVDVVLNSLSGSLLHASWQCVAEFGTFVEIGRRDMIGRGKLALEQFESNRSFVGFDLTHVVAQRTQLLQSLMQR

AVEYYKLGHIKPVCARQFSAVNISEPFRYLQKAEHIGKFVVTMPDNIKDLPVEPSHDAISLRSDRAYLFVGGLGGLGRSI

SRWLVERGARHIVFLSRSAQDVPDDDPFIQELLALGCRPTRISGDVCKYEDVVRTIKSVDEPFAGVFQGSMVLRDGEFAS

MPWPDWLAASQPKIQGTWNLHNVFLKMQTEPLDHFFLFSSAGATCGHWGQANYNAGNTFLDCFVNYRHALGLPASSLNVG

IMGDVGYVSENREILDALRATASYISKEPELLDCIELMLKRSRPDSAEQAAVAAAAASTLPRGPGLGYRQHSQIAMGLRS

PIPLSSPANRVVWRKDPRMLLYRNFESHEASSSTSTDGSSDDEKLKQVLKDASTNMVLLTSPETGDLIAQHIGRTLKEFM

MKTDGDVDLEAPLSNIGMDSLISVELRSWIRRRIGIDITTLEILRSESLKALGEVVQGKLVEKYKARA

>Micca1|7916

MDEPIAIVGVACRLAGEVSSLDSLWDMISKVKTGHGPVPGDRWNSSRWYHPDPDRKGGISPRHGYFLQQDISQFDAPFFS

VTAREAAAMDPMKRLLLEVTYESIENAGILVEDLMNSRTGCYVGCMTNDYEMLSLHDIYDIGHPAATGLSEAMIANRVSW

FFGLKGPSLTLDTACSSSLYTLHLACQSLRLKETNMSLVAGVNLIINPNTMHQFNALHMLSPDGISHTFDDRANGYGRGD

GIGVLVVKRLSDAIRDGDTIRAVIRGTGANANGKTPSITQPSSEAQAELINQTYQDAGLDQSETDYFECHGTGTPVGDPL

ELAAIASTIGAARRTAGKTPLYIGGIKPSVGHTEGCAGLAGVFKAILLLEKGMLVPTYGVERVNPKLKLSEWHLALPHNI

LRWPSPGPRRVSVNSFGFGGANAHAILDDAYHYLQERGLAGRHNTVVHDDYSASDAGLLRDAPVEDDTAKQLFLFSSKDQ

TGANRVSGSLNAWLQSDLRDKPCPRLLENLAYTLALRRTHLEHRTFAIASSLSELAERLSQGLPTATRSQRHGDNLVMVF

TGQGAQWPAMGLELFENPIFRQSIHDSQSRLEALGCRWNAIEELTKAPNPNIDRPEYSQPLCTVVQVALVDLLRFWKISP

TATIGHSSGEIAAAYAASLLSHHDAIKLAYVRGQSSAVVSKQGAMMAAGISPHDAQVYLEKVSLGSAVVACVNSPSSVTF

SGDVDAINQLESLISSDGKFARKLKVTTAYHSPHMREVSSQYLEMIGEISPRQRDETNSSSRNSPVMYSSLTGSMVSSVK

ELNAQYWVNNMQNPVQFSQGLLALLKHKNVAPGGTTRPVAVHWGGFLEVGPHATLQGPLRQVIDASNNKFAKSAPYVSML

VRGKNAMETSLNAAGVLWAAGCKVDMRAVNQHPNTANMLCNLPSYPWNHTRRFWHESYSSRSYRFPSHERSDFLGMPEDS

QNSHEPRWRNYLRISENPWIEDHTITGTVLYPGAGMLVMALEGVLRTADSSKAVKGFQMSDVMFERGLMISLDDGSPVET

RISFHPHSIKPDSWTFTVFSMTKGSSWAKHCSGSVALIYEKASEVEDGRLDDALWQGQLDIHKSLLSEADSVAINVDEFY

KNLDTIGMRYGPTFRNAVSLTAVPSMKASYGSVAVPDTKSTMPKHHESSHVIHPATMDSIFHIMLASLNTGQPLKQAAVP

YSIEEIYIARDQPKESGTLYSGHSRLLSRGGHEITAELFVSDQEWAKPKLTVKNFVLRQVTSEDGPETADTTSSGDIKNC

ARITWIPDLAFLTSNKDLTKLVSSKETDTLMDALLDPVFLKTSNNTVLVAMFDESESSLDILLRLKSRTYGPKKIIALAS

SEPGGRLMRATLGESTENMTITYRWLPEKELLQTLAPEANDLIIGLGVPDIAKNAATLAKLAPLACLTYTRQPDEVDIVV

QGSKRVGHFLGDSTSVLFASSTNSPSAELPDSVVLLLPASPSDPLFTFAATIQTKLESSGVSVARTNLTPRRVASLNGQT

VISLLEFDNPLVYNWDEDQFNAFKKLVSTVGHLFWITHGSVLDSWLNGVEFAASQGLFRVMRNEYPIANLPSLDLSAAAD

VSSIQYIDLVLKVWRASLADDAETEYAEANGMVYIPRAVEDAGFDYELQLANNTAKPIWSTLGAPGKPLKASKSANVQGY

IWTPDKNTELPLQAGEIEVKVEYAGLGIGNTMHSVRHAVGVVTRRADSAKAFKLGQRVIIFSHDAARTHVRQSEALVAPL

PEGLGSQEAVVLVEPLITAQYTLVEMAHLKCGQALLLDNAASAIGQALILVAKAVGADVFALVHSIEGRDIISDKFEIPT

DHIFDSNLDNFVPLIRSATNNHGVDVAVSQQSGAHVTGAMSVLSEFGQFIDINSTSTKDLLLASKANMTLTRINMAAVMK

DRPSIISKLFQQAFNKYTLQGPSPLTVLPVTNLSASVNSIQAEHVAVSLNNASPVLMAAAPSQELKLDSEAIYVLAGGLG

ALGLDIASWMVDYGARNLVFLSRSGGSKNQGDLQKLTERSVHAEALKCNVNDAASVAIVFDNLKASGRRIAGVIQLAMVL

EDGIFENMSFAQWRRAIEPKTKGSRNLLANIWPGDKPFFILLSSITGIIGNTAQANYASGNTFEDALAQHARSHLGIHAT

SIDVGLVSDSSHFTTAGEFGDLAGYLSRYQHGWRGLQTNLKELGVVIRAIMRSSTTMERSAPAQVVLGLGDHIECNESTG

GFSRDKKFELRIVKAGNRASDDNVTQDVGIRLSNATSMAEATAVVEENIKDLVAAAMGVSVEEIDPQKPLYDYGVDSLQA

VEIRNRALKNMKSDISVFDILSAMPLAGVAAKIAANSQLFKAIVGED

>Mictr1|651481

MTNGYGSGVPAANGHTNGVSGSDVSDNHQDNIFEPIAIVGMGMRLPGGVHNAEAYWDLLVNGKDGRCRVPKDRYNIDTWF

GPGRVTHVGTKFGHFLEELNLANIDPNFWSFTKQEAELLDPQQRLFLEVVYEALENSGTTRWRGKDVGVYVGALGDDWGE

MEMQDGQDLNPTRTYVYGDYIIANRASYEFDLKGPSIVVRTACSSSLMALHVACQDIHSGDCSDGAIVGGINLIITPRTT

VALTEQGVLSEDGKCKTFDADADGYARGEGVSAIYIKKLSDAIRDGDPIRAVIKSTCVAADGKTAGFTLPNPESHEKLMR

RGHHLAGIYDFSKTAMVECHGTGTAVGDPREVSAVANVWGDHGIYIGSVKPNIGHGEGASGLSSIMKMVLALENKTIPPN

INFKTPNPKIPWKEAKLKVATKAMPWPADRLERVAVNSFGIGGANAHVILESAASFGFGQKPEPLSDETGTERLRRHLLV

FSAKHPKSLERSAQKHEEYMTISPGSLRDMAYTLNTKREVHPYRAFCVTDGLDSLELSRIAKPILGSHRALVFAFTGQGA

QWARMGRELLESDSVFKETVDSLDATLSQLPDGPKWKLRDEILAPKKTSRLVEAEFSQPCCTAIQVALVDLLRSWGISPN

AVVGHSSGEIAAAYACGAITAQDAIRIAYYRGQCTLALKPSHKGGMAAIGLGRESVEQYLRPGVAIGCENSPASVTLTGD

SDILEKVMEDIRSADPSVLVRALRVECAYHSDHMKVVASDYATRLGTIKAKEPRVPFYPSATDQVSPDLSTSYWIQNLVS

PVLFMGAVQAALRSQDNVTFVEVGPHSALAGPIRQTIQSVGKPGAEYIATFVRNTDAYQSILSTAGNLWLAGVSNINLGT

VNGEPEGGKLLTNLPPYSWNYDGEYWLENRLSRDWRFRQFDHHELLGARVLESADAGPAWRCKLRVQDAPWLSDHDILGD

IIFPGAGYLCMAGEAVKQLRPGSDDYSLRRVTLASALVLHGEPIELVTTLVPVRLTTTVDSEWYSFSISSVNPTTNVWTK

HVSGQVRPGRDGGLTPRTPEIESLPRKVPTSTMYSVWQRFGLNYGGRFRGLSDISAHTTEKKAVGTIYEMLSPEENAVYS

VHPAGIDAAFHLSNVCLCHGLGRNFSTPSVPKYIEEMYVGKPEGPIRVMGDAASKGRGGSTSNLVGVSSGKVVLSWNGLE

LSPLSDGSEVVDEDPHAAAILEWKTDVNFIDAGLLLRSLEKDPDDAQHRLVDTMGLACIIESRNQLAGQETSQWHLVKFR

DWMDIPYAEAVEGRYPHVPNCAEIAVMSSKERIKLIKDLLAASEGTEANAVAIALYRIFHNIVGFFTGDADPLEVLMADN

ILTRMYDFTNNADHVQFLSLLGHMKPNMRVLEIGAGTGGTTATMLSALKSDQGERMYGTYVYSDISAGFFLGAKERFRDY

QAIEYSVLDITQDPASQGFEEGSFDLVVSSNCLHATPNLEQTLTNVRKLLRPDGRLFLLELSPESSKSVNYVMGPLVGWW

LSEDGREHEPYVSHQVWHEKLLQSGFSGVEAYAFDGNMSNSIIARPVAPPVEKLTKVSIVCGDPAHPKVTEATHFLQETG

LDLEIVQVGQPLSPGRPAVFLMDLEGPFLANINEDRFNAFKQSLFSVQDIAFCWVTGACQVDCKDPNYALINGMARSIRQ

ETGIDFVTFELECFDENAWKGLSSLLATFPSRVTDGESDTDFESEYVFHAGTIQVGRMHWIKITEELQDREQKDQVKRLI

VDKPGIIHTLHWRQGAPPALEADDWVQVDTRAVGLNFKDVLIAMGIVEASNDGLAVGDFGFEGAGVVSSIGPDVQHLRVG

DRVAFSSTGCFATSLTMPGIACTKIPASLGFEEAATMPCVYGTAMYGLVDLARLEEGQTVLIHSACGGVGQAAIQIAKMI

GAEIFCTVGNEEKVEYLIKVFGIPRTRIFNSRDCSFLPGILKETDGKGVDVVLNSLSGELLHDSWQCVAKFGTMVEIGKR

DFIGKAELAMDRFENNRAFVGLDHTELWSHKPKVSSRVLNRIMELCDQGKLGPISPIKTFEAAKVEEAFRYMQKGQHIGK

IVVTVPKHQEDEALEAEPARRETLLREDRTYVFAGGLGGLGQSIATYLAEKGARHLVFFSRSATKFAESNPDFFKELQSL

GCEAQVISGSINNMNDVEKVVASAIHPIAGILQAAMVLQDANFIDMTFEAWQTAVLPKVLGTWNFDEALKKQEEPLDFFF

LFSSVSGTAGQIGQANYAAGNTFMDAFVQYRRSLGLACSTLAIGIMEGVGFLDRERHLLEALRATSLHFLHEQDLLDSLE

LMIGPRSSYDDKITSADTAAAGEVNGYSKLTSGYANPGHIVIGLRSKLPLLSPMNRTGWKKNPRLLVYRNIESQDERSSG

PTTDGGLKEFLSSCGKTPEMLETEATANFLAHEIGATLFSFMMRGDEEPNLTVPLASVGVDSLVSIELRNWFRQKIGVPF

NVVEIVGAASIADLGRMTAEKLAEKHKR

>Necha2|123123

MEFSSQLNGHSRASIGKPLYEPIAICGMGMRLPGSINDSQAFWDLLQNKRNGRCKVPKERYDVDTWFGPGKSGHVASKFG

YFLDHVDLAKMDTSFWSATKDECASMDPQQRLLLEVAYEALQTAGQKSSELRGRKIGVFVGSFEGDWLELDQRDVENFSR

HRQSGSGDYMAANRIHYEFGFMGPSTVIRSACSSSMIALHDACTAIIAGECEGAIVGGCNLILSPRMTATMQELGVASPS

GYCHTFDADADGYARGEAVSAVYIKKLSDAIRDGDPVRSVIRSTCFNSGGRAATMSAPVAAAHEALMRRSHELAGITDFS

RTAMVECHGTGTPVGDPIEVQAIANVFGDHGIYIGSVKTNLGHGEGASAMSSLIKMTLALEHKIIPANLNFKTPNPSIPF

KSRKLTVPLESTPWPKDRDEVVGINSFGVGGSNAHAVLASADSFGAGMAQESAAEKTIVSWKPALLLFSAKHADALKKII

ENQQAYHVAHPNRLTNVAYSLAIKRDAFDHRAFAVADGVDDWSAKFSTRTSAPREPPKLVFVFSGQGAQWAQMGKELVQL

YPSFGESIKELDGYLSALGDDGPTWSLMEELMRGKKTSRLSQAEFAQPCTTALQIALVDLLRQSGLTPDAVVGHSSGEIA

GAYASGALTAREAILAAYYRGKAMPWVEKALVAEGTQGGMAAIGLGSEQVMPYLTKGVVVGCENSPDSTTLTGDKSALDI

VMSRINEAHPSVLVRELKVDRAYHSHHMRIGAPHYLASLKAQGIEPQPPSVPFHSSVTGEVISNAEDLRPQYWIDNLVSR

VRFSTAVNSVLSSSKSPKTFVEVGPHSTLAGPIRQILQAASAQADYVNVMKRGEDGSRAYLQAMGELWSLHVPLDLSAIV

DKGDFLTDLPLYPWHYEESLWAESRLSKDYRSRKFRHHELLGSRIIESTDHSPAWRNILRLESVPWIRDHEVFGDVVLPG

VGYIAMAGEAIRQLTGADDFSVQRVHIKVALALEHDSKIEVVTQLERVALTNSLDSPWYNFTISSFRKAADGGAGSWIKH

IFGQVRGGSDFQPGAAPDVSPLQRAVSEKGWYRKFRASGLDYGPRFQSLANITADPSTSEIVANITNDVRDAESYYPIHP

GALDCMAQALVLALCGGLTRNLGSMAVPTYIDELYCRPSAIKDMSIRVKATECRRNAYTGDLTAVCDGQIAVQAKGFRLT

VVDNTTKADASSNLGARFRHGAVHLEWKPDINFANVDMLFSPDLPPDQDFHLLDQYLALTSIEVAERTRNMKPAQDFMAL

FQKWTADFAAETQLEALFDASEKDILRNDSAYRNKVMDKTYVQLMDCQTRAAAEAIGRIVKDIEGLMNGTTDGLDILMDN

KLLHDVYDGMQLTEMGSFFDLVAHKKPNLRVLEIGAGTGGTTAKVLPLLQSAYGERMYLSYTYTDISPGFFPPAQERFRE

FPGLEYRVLDISQDPAGQGFENDLESYDLIIATNVLHATERILDTLKNVRKLCHPRGRLFLQELSPVSKWPNMIMGVLPG

WWLGAEDGRPDEPYMDEQRWDKELTSAGFCGAETVFFDGRLCLNIVTTPKEMDAKRPRVTLLTPGQVSESSEPKVRAVSE

QLQGAGYPVVVHPFGQAVLDLGMDEDVVALLDVDKPFFHNLSESDLHGFQAFLAYAKGRRCGLLWVTGASQAGCTDPRYA

PVIGVARVLRLELGMDFATLEMGGQTEDSSLKIDAPTVARVMGEFRRRDSNAMESDIDTTCEAEWVYSAGTTLAGRYHYL

DMDAELKKPAPSSGTETALDTWRPGLLDALYWKRLPVEELAPDEVRVEVRAVGLNFKDVLVAIGIIAEPVKIARGMGYDC

TGVVTAVGRDVTKHRIGDRVAVCESGTFSTSLNVLEQLCATLPDDMSFEEGATMPLVYATASYCLLDAAQLAKGMTVLIH

AAAGGVGLAAIQLCNMVGCEVYCTVGSDEKVQYLMEHCGIPRDHIFNSRDTSFLPGLMAMTDNRGVDVVLNSLSGELLHA

SWKCVAEGGAFVEIGRRDFIGQAKLAMEPFETNRSFIGFDLGAMRNQRPDTIARLMDRMMKQAKEGRIKPILPMKIFDAA

QISDPFRLMQKAQQIGKMVISMPASADTELSSEAIHKPFRARSDAAYLLTGGLGGLGKSISTRLAERGARHFVFLSRSAE

KPEHDAFFKEMESLDCTSTRVSGDVVNYDDVVRAVKAAGRPIAGALHAPLVLQDNSFLDMSFDEWNAPFRPKVEGAWNLH

KALLSEKLEFFFLFSSFGCMFGQWGQSNYSASNTFIDAFVGYRHSLGLPASTVNIGVMGDVGWVADNPEALEKLQAAASH

VSQESDFLDCAELMFMRPGPTPQTDPAHFVQHSQVGFGLRTTLPILAPNNRVVFRKDPRMLVYRNLEADAGWSAAATTGA

AGADPADEELTRFLQAASVNMAVLRGEEATRFIAQNVGRTLFGFMLRSEDDFDVLTPMADLGIDSLVSIELRNWITKRLV

VEVNVLEITRAGSLLELGGLLQTRMVERYQARR

>Oidma1|128615

MTSDQNRAHSTHRVNVIKTNGISKVNGVKPHVEPIAICGMGLRLPGGVTDAQSFWDMLINKRHGRCSVPKDRYNVDSWYH

PDKDGHVPSRYGYFLNNLDLRNMDTSFWSMTKKEIEIMDPQQRLALEVVYETFQNAGQKPSDLRGRKIGVWACSFGGDRQ

ELDSRDTQTRHPYDLTNAFDFVLANRINYEFNLTGPSVTIRTACSSSLTGLHQACQALYNGDCEAAVVAGTSIIYSPSLT

VAFNEHGVLSNSGLSKTFSADADGFARGEAVLAIYVKKLSDAVRDGDTIRSVVLSTAIGADGKSSTLTAPNPVEQAELIR

RTHELANIKDFSKTAMVECHGTGTAVGDPLEAESVANVFGDCGGIYIGSVKTNIGHVEGAAGLAGLIKMVLALETGIIPP

NLNFAKPNPKIPWDASFGIGGSNSHALLASADYVGVVGERDMDSLTDDTRSFTPKLLLFSAKHPEALEKMITGHQAYYMG

NRNSLLDMAYTLAMKRETLSHRAFCVANGIDDWTPFFSTRHGSYDSGKLTFVFSGQGAQWAQMGKALIKSIQSFRETIEE

MDMVLQSLGDGPKWKLKDQLLAPKKTSRILDAELSQPCCTALQVALVDLLKTYNITPEAATGHSSGEIAAAYASGAITLK

EAIIIAYYRGKVLLDVDPSIGGMAAIGLGTEQVLPYLLPGVLVGCENSPNSVTLTGDKSTLEAIIQKIKSENPDVLARAL

QVDRAYHSHHMESLANQYLDLMSCHLNPRDPSVPFISSVTNQVINQGAELGPSYWVQNLVSPVKFSTAMSMAINFSTSKK

IFLEIGPHSTLAGPIRQILKAENSSDEYVSVLTRGKDAYEEVLRAVGQLQLQNYPVNLEQAVGRGTFLTDLPLYPWHYEE

PLWHESRLAKEYRLREFPHHELLGSRILESTVFNPGWRNVLRLEDVPWIKEHEIQGDVVLPGVSYLYMAGEAIRQLTGKA

DFTCQKIHMKVALVLNGEDETEVITQLSRVNLTNALDSEWYDFSISSYKNESWIKHAFGQVRGGGDEFELQDLSAKEAAT

RSYTRACSSKSWYRKFRSLGLEYGPRFTPLKDITADPLNDKLAASIKVGLRPGEEKYYPIFPGALDGVPQSLFPAVARGL

TRNFAKPAVITYIDEFYLRPPRVVTEELKVLAEVTEQRPNGFLGDVVALSLEDGGQVVVRSKGWQMSCLVDEADDGKNPH

GAAELEWMDDIDLVDAASFIRPTGIKTQVREYQLLDRLSLLCFAEAQNRLRSTPPPTREHLKHFHKWINSHIDDVSSGRS

TWFGVPDAVDLLALKTEERNNLIESIYSQLEGTPAYAPATALHRVTYNCEGIFDGTVSELEILLAGGALQKVYDFLLEDA

DLSGFLSLVSHKRPNLRVLEIGAGTGGTTATILPILKSAHGDKKYLSYTYTDISAGFFSDAKERFKEYSGLEFAVLDISK

DPLEQGFAAESFDLIIAYNVLHATENLHQTLSNVRKLIHPEGRLLLQELSPQTMWVGTFGVLAGWWYGQADGRTQAPYVA

LERWTDELAKAGFEGVSSMYDGYMNNNIISQPASATKPPERVTVLRHADQEVRSIRSALEAAGYHLDEVVLEDPAAKLPP

GQDVISALDLSAPFFSSLDQDKFSNFQRFVNAARDGACGIFWLTGSCQIGHTKPEYAPVLGLSRVLRNDLELEFAILELD

DLGAASKVVPSVFAQFQKRISEPDVDPEHEWAHVDGRTFISRYSYVKVIQEHRNTLPAGMTVKKLEQHKSGVLSSLYWKP

LTAKALKDDEVRIEVRAVAMNFRDVLVAQGIITDAEAIDDGLGLECAGVVSEVGRGVDKFKVGDRVGALTCGAFTTSHVV

SQTLCFEIPDGMSPEEAASIPVTYGTVIHGMIDRGRLTSGMTVLIHSAAGGVGIAAIQLAMIQGAEIYCTVSNQEKIDFL

MTQFNIPRDHIFNSRDASFLPAIMAATNNRGVDVVLNSLSGELLHASWKCVAEFGTFVDIGRRDFIGQGKLAMEQFESNR

TYTGLELLHLWVHRPLVAGAILQRAVDFWKQGNIKPVVAPTKYSASQISEPFRGMQGGQHIGKLVVSMPDLHELAEEPVY

EALQLGDDRAYLFIGGLGGLGRAVATWLVEKGATEIVFLSRSAGSSPAHDQFVNELATLGCTAKLVSGNVAKYDDVVQAI

KTAGKPIGGVLQASLILRDSNFLDMKWEDWLAASQSKIQGTWNLHNALLSEQSNMPLEFFFLFSSTAATGGWHGQANYHA

GNTFVESFATYRHQLGLAASALNVGFIKDAGFVAENAGASDVARAMGQSFNTEAELLDCIELMLKPARSKPSQDRKLGDG

DFARGLVQKALLAMGMRSTASVTSSTCRIPWRKDRRMLAYRNVEAHELESSSVSGSGSSSNELARFVREVRSNIVLLQSA

ETAIYLATEMGRALLEFIMRGDAEVDVQAPLAAIGLDSLVSLELRSWIRRWIGVELATLEITRCDNLQALGTVVQGKLVD

KYNIKAEE

>Penla1|358897

MAICGMAMRLPGGIRDADGFWDLLYNKRSGRCRVPKDRYNVENWYGPGKIGHVASEYGYFLDDVDLRNADASFWSMTKQE

IEAMDPQQRLSLEVTYECLQNAGQRPQELRGHKIGVYLGTFEGDWLELDGRDPQHYHMYRLTGYGDYMSANRIHYEFGFM

GPSVTIRTACSSSLTGLYDACHAISAGDCDSAIVACANIIYSPRTSITMQEQGVISPSGSCKTFDANADGYARGEAVSAV

YVKKLSDAIRDGDPIRSVIRSTCINAGGKASTLTAPNTAAHETLIRRGHELAGVTDFSKTAMIECHGTGTAVGDPIETAA

VANVFGEHGIYIGSVKTNLGHSEGASGLSSIIKMTLALEHRTIPPNINFTTPNPKIPFEQCKLKVPTEPLPWPKDRAELV

GVNSFGIGGSNAHVLLGSAASFGIVSAEQTLMASEQSTEVAMAELTPRLLLFSAKHQKSLVRMVANHQAYFLSHPESLGD

MAYSLALKREELSHRSFCVTNGEDDWVPSRTHRTTGRAPPMLIFTFTGQGAQWAQMGKSLIDQMPRFRRSIEKLDQVLQA

LPNPPQWKLIDEIRASKKNSRLSEAELSQPCCTAIQIALVDILEYYGIHPDAVIGHSSGEIAAAYASHAISGVDAIQIAF

YRGLVMCNLNPAKRPGGMAAVGLGAEELTPYLRPGVRVGCENSPNSTTLTGDKGPLEETMKAIKEANPDVLVRALQVDRA

YHSHHMETVAPEYVELLTNQRVQAMGPSVKFFSSVTGHRMDQSQEFGPLYWANNLVSTVRFSTAMGEAMQSLIGPKVFLE

IGPHSALAGPIRQILQHYKSTDEYFNILTRGSDSHKDLLKAVGEMWLQNIPVNLTAVFGQGRFLPALPLYPWHYEEPLWC

ESRLSKEWRLREFPHHDILGSRVLESTDQNPSWRNILRLDVVPWIKEHEVAGEIVFPGVGYICLAGEAIRQLTGENSFTA

RRVHIKTALVMHQGQDVEVITQLQRIPLTNVAESKWYNFSVHSYNKGTWVKHIFGQVCAGSDREHQAPSLESLPRQLSRR

GWYRKMKEMGLEYGSRFMGLADMTAHPIERKIIATVVNDLREGESKYAVHPVSLDCLLQAIVPATFNGLTRRFQHLGIPT

YMEEIYVCPPLHPEMTIEARADEQPTAALSGSIIAVSDGHVTVDITGLQMSAIGDAADASGQDPHAAVELEWREDINLID

DAAKLIHPAKNRTDLHNILDRFASASMVDTFTRIQEVEPTRLHLTHYRKWIESTAGLIKLGKYSGLQPEDEIAQVSDAER

VNIIESLYISLLETDAAATATAIYRIWKECQGIFTGETDELELFLDGEVLHSLYDFMQNSEYQVFLELLAHRKPNLRVLE

IGAGTGGTTATVLPALQSHYGERMYHSYTYTDISAGFFPQAKKRFGNYPGLEFATLDISQDPLAQGFEAESFDLIIACNV

LHATSTLQDTLTNVRKLLHPQGRLFLQELSPATKWINYVMGVLPGWWLGEKDGRYPEPYIDIHQWEALLSKTGFSGVDLV

SHDGYLNNNIIAKPVADTQRPKRITLLHWCEKSSSGTASISQLLSSAGFGIDLYAIENTNTPPPAEQDVVSILDLDRPFF

HDLDESSFENFKRLLSRLRDTDSGILWVTRACQVGCKDPRYAMINGVARVIRTELNLDFATLELEDLEQETIAHIPRVLG

EFQRRISEQNIHTTTEWAVVGQKPLISRYHYIQVAEELKNNTVTGSSTVKLDQSRPGLVDTLCWKSMPPSHGLDENNVLV

QVKCVGMNFKDVLISTGVITEKSSIGQGLGYEGSGLVLQVGSAVHKLSVGDRVIMSSSGSLASIQQLDQRLCVKMPDSMT

YEEGATMSAAYCTAIHCLLDVGGLRKGQSVLIHSASGGVGIAALYIAQMVGAEVYATVGSEEKIQLLTSTFNIPRHRIFN

SRTSEFLPRIMEETGGMGVDVVLNSLSGELLHASWKCTAEFGTFVEIGRRDFVGQGLLDMQPFEPNRSFVGFDLLLFSNK

RPERIESIMTRTMDYYQAGFIQPIKPSTTFDAVSIVDAIRYMQRGQHIGKIVITMPENSTDLSAEPLRQELILRQDRAYL

FVGGLGGLGRSITTWLVEHGARHIVFLSRSAGNVPDDDPFIQELAVLGCTTTRISGDVSKLDDVLRAIRASGKPVAGVLQ

SSMVLRDNSFVDMKWDEWLGAVQPKVQGTWNLHNALLSEQPEEPLDFFFLFSSAGAMSGQWGQANYNAGNTFLDAFVAYR

HSLGLPASTVNIGVIQDIGYVSQNPEILDSLRSTAQYLMRERELLESIELMLHRSSPAESVADQAFGRYVTRSQIGIGMR

STVPMDAPANRTIWRKDPRMLVYRNLEVQSGPGSSSTGSDQVLTQFLREIGSNMTMLKASETVELLAGEIGRTLFGFLMR

ADTEVVDLDAPLASVGIDSLISIELRNWIRRKIGVEVTVLEIVRADSVRDLGVLAQKKLAEKYEARM

>Pensub1|2628

MGELIDHTVNHQLPLVVDTAARIGVEETQNCQDDLDLEDKPVPFPIAIVGMAMRLPGGVSCEEEFWDFLINKRDGLCKVP

DTRYNIDAFYQGSRPGAIRTKHGYFLEHDIRHFDPEFFGLSKIEAAKLDPQQRMLLEITWECMENGGQVGWRGKNIGCYV

GVFGEDWLDVKIRDPQDHDRYRVVGAGPYALSNRVSYEYDLGGPSMTIQTGCSSSLVGLHEACQALYSGECSSALVAGTN

LIFTPTMTTSMSDNMVISESGVCRTFDAAADGYGRGEAVNAIFIKPLDKALRDGDPVRAVIRSSATNCDGKTSSITTPGS

AAQERLIRKAYKKAGLDVSSTGLFECHGTGTTVGDLAETSVVGKIFGDKGIHIGAVKPNVGHSEGASGITSVIHSVLALE

NRTIPPNAHFQDPNPNNGPQLLVVSAKSKYSLNRQIDRLQAYLESTKSPLSDIAYTLGLRREHLTHRAFAVTQSDGKISP

FERSVSVRAPTVFVFTGQGAQWPGMGRQLIEKAQGFREDIQMMDRVLQGLKSKPLWSLEDELLKCDDQSRVAEAEFAQPL

CTAVQIGLVNLLRAWGTTPDAVVGHSSGEIAAGYASGALSAEMAILIAYFRGQAMKDQSSRPGGMAAVGLGPEQVEPFLK

PGVVVGCYNSPESVTLSGDSEILTEVLDNIHGDSQDTFCRKLAVNVAYHSHHMADAGEVYEKMVAPNFYHQPSMIPMYST

VTGTIVTDPSILSPSYWRRNLQSPVLFHTAIERILKDDTLSKLFLEVGPHSALSGPIRQSIKLANTGEHRYIPTIIRDKE

SWRSLLAAAGNLHTHGASIDLRHIIPRGKVVTKLPPYCWQHTEKYWDEPRIVHDWRHRQYPHHELLGSQILESNHLEPCW

RNILKLENVLWLLDHKLGTDIVFPAAGYVAMAGEAVRRVTGSSDYSLRNVFIRSALIIEDSVEILTSLRPEKLTDHVDSL

WFDFTISTFQNGKWKKHIVGQVRGGAEQEYGVPSRQSYPRQVDSDKWYHALSKRGLNYGDHFRGLEQITASSTTQQASAV

LHSDEPPLDSYYALHPTCIDECFQLLSVAATQGISRRMTKMCIPTAIESLYVTEGRGPMDLNVSCETIGGTLEANSELFS

NNCVSLRMERGFFFSINDPESDDINTPLASNLHWTPHIDYLPFREQLPPHEPAFDGKTMARLTSTCVAEAYRLTKNSTPV

SDHLEKYHVWLQDLYLKIKEKSPEIIPEMREEDVSVLSVCGPYADALNKKGRESHPWLIPSLELLHRVCFNLHDVLEGRI

NPLELLMENAGMKEFFDAMCAISPCEDFIALLGQSYPGIRILEIGAGTGGLTSTALKTLNPESGRIYSRYTFTDISPGFI

ADAQERFHEYDAVEYATLDISRDPEEQGYVPESYDLILAGNVLHATAQISSTLKNVRKLLAPGGRLLMQELSGDNPMINF

ITGILPGWWLGENDGRSNSPALSVERWHDELTKANFTGVDAVRYANDRPLSLTTVILSRTKAADDNAIGGQIGLLYLSHI

TEWGRELEKALTLAGYTVTWYTLQEKAPTGCDLISLLDLEGPFFQYLSADEYDLFRNYLSTLAESHLLWITKSLQITCDD

PGFALVLGMARTLRNELSSKFATVEVDQFDDISIASVLKVLRALKVQSQRPWLDPDYEYALQDGKILLPRIQWSSLDQQL

TDVPHLSAARSLDIEFNGIFDSLTWAISMSPVSQSDLKEHEVEIDMKYVGMNFRKHIIPAGCA

>PhaPMI808|603510

MASHDDAYIHGYKEGYTSGFTKAHIELKHQPIAIIGMSCRLPGSVSTPDEFWELLARTRTGYSPVPASRFSANRFFHPNP

GKGGAMNAQGGYFLTHDLSAFDAPFFGFTQQEAISLDPQQRLLLECTFEALESAGVPKHEVVGTDVGVFVGGSLSEYDTD

LFRDPETMPMYQATGCAMAMQSNRISHFFDFRGPSFTVDTACSSSLVALHTACQSLRSGESTMALAAGVHLNMLPEVWIS

YSMSRLFGEAGRSYAFDQRGTGYGRGEGCGMILLKTLDQAIKDNDPIRAVITGSGINQDGKTPGITMPNGSAQEALIRSV

YHEGGMDPRHTGYIEAHGTGTRVGDPIEVAALHNVFGEDRSRRKPLYIGSVKSNIGHLEAAAGIAGVIKTALMMERGFIL

PNYDFKYPNEKIPFDEWGLKVPIRQQPWPLGKKWASVNGFGFGGTNAHVVLTRGPLERKTMKEEIDTQTQERLFVLSGND

KSTAEKTMKSLGIYLEQRPEVFQNDLLSNLAYTLGQRKSLHPWRTAITASSGVGLVESLSSGKVIPAKQELENVRLGWVF

TGQGAQWWAMGRELYQQYPVYAAALDKASSHLSSIGATFSLLEELGKDEKSTRINLAHISQPACTAVQLALVELLFSWKI

RPTAVVGHSSGEIAAAYSAGIINFEDAMTIAYHRGRLIPILKERYPELDGCMMAVGTGTTDILPLLNKIPSTLGEVRIAC

TNSPSSVTISGDTNAVLELQNLIEEAHPGMFARRLAVDTAYHSHHMNLVAKDYTESLLKLHPPRKSEVSFHSSLLGRIAT

YSDLDASYWVQNLTCTVRFDEAAQSMCKSTGEVSAGINFIVELGPHSALQGPIKQVLKHVGGLAAKTQYSSVLSRKKNAI

QTALALAGTLFVKGIMLDMGSINFPKPLERPPQVLTDMPRYSWNHLTSYWHESRLTKVHKFHDTPRNDLVGVVAPYSDGI

EPTWRNVLRLDDVPWLRHHQIQGVTIFPISGFVIMAIEVMAQHARANNLEFCTLEVKNLNVKTPVMLTEEETEMTTTLRP

NTESHQGSSFGFHIRSWSKSKGWTENCTGLVSIHLGDDNDVDGMHVNKKKQQRLDTRMATVKLEADQPIQTPLLYTSLSE

IGVSYGATLQNLSTCQASPKAAYGTITVADTVSEMPNHHESSYVLHPTILEQLITLYWPILSAAGPLGTVHLPSSIGKVT

VSWKAFEHFNSSGKVMQALCQASTKLSNSRSNKLSMLAMTDTNEHLLSVEDLMISPIIETNTGIEALGPRELCYKSEWEP

VNSGGEVNAVEESHFDAEIVIIHGETTPQSSLASVLSDQILALTGILPTMGSLATVADISKDKLCIVITELEKPVLASLD

ESAFSALQRLLTTVQGALWVVRGAYIDSRNPDTNMIIGLSRTLRSEGTLMKFVTLDLDARRDASDTEQASTILRVLTETL

GKISKTEETEFVERDRELFTPRIVNDVVLNDYVHYQIHPPSTEPTCFMDMQRPLRGELETPNVPDSLTFKDYKLSSLPDE

EVEVQIKAIGLNSEDIRADSVIGLECSGVVTAVGTNVPNFQVGDRVAGLAPNGSLSTVTRAHYPFLFKLPDDITFGLAAT

IPLAHSTASYALIEKARLCEGEVLLIHDAASAIGQAALQIAQMIGAEILVTVQTLVEKTMLMREFGVLENRILYVGSRYL

TETINDITSGLGVDVIFDNLTESHFDLTTYDTLASFGRHICVGAQRPLDIKHLKDNTQVLSLNMISLANHRPKIVQRSLA

NVARMLKHGKIQPLQDIKSYSISETCVALQAVQSAGLHGKAVIVLQEDQVVTAPRIEKHFDLLRSDATYILIGGTGGLGR

SMAKWMVSKGAKNIVLLSRSGALRGKAKEQVDRLNENGASIVVRSCDVANRDSVDKLVSDGLTDLPPVRGIIHGAMVLHD

VLFEKMTHHQYVSVIESKVQGAWNFHHALSAAGASLDFFIAISSAAGAVGNRGQAAYAAANTFLNGFAQHLIGQGISAAS

IDLTAVSDAGYLAEDAEKAAEVARNLGSDTICEAEVLALIQAAIEGKLTSCNGHPITGMRITPTMRPFWSNDAKFIHLLR

TAEAASASSATIAKVSWSAAFKAAPSRPEAEQVVCNALVEKIAEVISMEPEELDTSRAISHYPLDSLTAIEVRNFITRMF

EASLQVLELLASGSIESLARVVCTKTKVGLPEA

>PhaeoFL0889_1|456719

MSPSATSLNTNGHTTNTNGHANGHTNGAGALNGSGFVNGSGSMANGHGAAHHEVNGDGGIPKPLAEPIAICGIGLRLPGA

INTTEDFWDMLVNKRSGRCKVPKTRYDVDSWYGPGKIGHVGTKFGYFLDNIDLENMDQSFWSFTKAECEAMDPQQRLLLE

VVYEALQSAGQKSTDLWGKSVGVFVGTFEGDWLELDGRDVEYLHRHRQMGSGDFMAANRIHYEYGFTGPSAVIRTACSSS

MLALHDACTAINTGQCEAAIVGGTSIIFSPTTTASMAELGMTSPSGYCHSFDAAADGYARGEAVSAVYVKKLSDAIRDGD

PIRSVIRSTCLNAGGRSSTLTAPVTAAHEANIRRGLELAGIRDFSKTAMIECHGTGTPVGDPIEVQAVANVFGEHGIYIG

SVKTNLGHSEAASGLSSIVKMTLALENETIPPNLNFKTPNPHIPWESAKLRVATEPTKWPKDRDFVVGINSFGVGGSNAH

VVLGSAASFGADRNRDSRPAAEISAPTLLMFSAKHPEALRKMIEKHQAYHLTHPDRLRDMSYSLAMKRDAFVSHRAFCVT

DGLDDWAVKSAVRAASAPRQAAKVVMVFSGQGAQWPRMGKELYESYPVFRHSLEAMDEFLHTLEDGPTWSLVAEMLKAKK

ISRLSSAELAQPCTTALQIALVNLLRQSGIEPDMVVGHSSGEVAAAYASGVLTAKEAILVAYYRGKTMSLVDESIGGMAA

VGLGAKACLPHLPEGVLVGCENSPDSTTLTGDKEALQKALVQIKEAYPDAFVRELRVDRAYHSHHMRRVAPQYLALMKSH

IQPKGALVPFHSSVKGHIISAAEDFGPQYWVDNLVSPVRFSTAVSNIMGATADPKTFVEVGPHATLSGPIRQILKADQKS

ADYVNTMTRGEDCASAMLKALGELWLLQHKVDFSAVSGRGQFLTDLPLYPWHYEEPLWSESRISRDYRLRKFPHHELLGA

RVLESTDQSPCWRNLLRLESVPWIKDHEVFGDALVPGVGFVTMAGEAIRQLTGASDFTVQHVHIKAAMALEHDSALEVVT

QLDRAPLTNALDSAWYSFSISSLRSGTWVKHAFGQVCAGSEFPPGPLPDVRPLPRTVSEKAWYRKFRGAGLDYGPRFQGL

RNITAHPTKPELVASITNDVRPAESYYAVHPGALDCLAQALVPALCSGLTRHLGSIAVPTYIEEFYCRPSATEDLAIHVL

ATEQKRNAYTGDVTAISDGQVAVKAKGFRLTFVDSGSNNDARTEKYSHGATELEWKQDINLADMSGLFYNEEPPVEVCTA

LQRFFALCTIEVAQRVRNIKPAHDHLALFAAWATKVAAGTQLDGLLEGAEVLREDGARRAAMMETLYSRLEDSEAHAPAT

ALRRIVNNTEAIMRGTIEGLDILMDDNVLHEIYNYMQLTDISGFLSLLAHKKPNLRVLEIGAGTGGTTAMVLPALQSAYG

ERMYLTYTYTDISPGFLLPAQERFHDHEGIEYRVLDISQDPAEQGFENDLGSYDLVISTNCLHATPRIQETLRNVHKLMH

PHGRLLLQELSPPPDGKWVNLVMGVLPGWWLGLDDDRPEEPYMDAKRWDKELTGAGFQGAQTVFFDEQASNNIITTPAPS

TSAEGQEKRVTLLVPSHPSEASEPHVDAVTAQLRDAGYGIDVCYLAQAADLTPDQDVVALLDVERPFFHDLDEAGLRSFQ

DLVAHAKERRCGVLWVTGQSQAGTKDPRYAQVVGVARVLRLELGLDFATLEMGFGPEFDAQAVARVLGEFQRRDRDTEES

DTTCEAEWVHVAGPSAGAGQVLIGRWHYLDMNNELKAPQAPKTEQKTYLKLDDSRPGLIEALTWKRLAEEDLGPEEVRVQ

VRAVGLNAKDVSIASGVDVEPRSIGHGMGYECSGVVTAVGANVTKHRVGDSVAVFSSGLFATSTTVSQDTCVKMPDGMSF

EEGATMPVAFMTAIYCLLHTGRLTKGMSVLIHAAAGGVGLAAIQLSQMVGADIYCTVGSEDKAQHLVSHFGIPREHIFNS

RDTGFLPALMAKTNGRGADVVLNSLSGELLHASWKCVAEYGTFVEIGRRDVIGQANLSMQPFESNRSFVGFNLSYMGTQR

PDIIARLFNQMMDYVREGHIKPLLPMQTFGAAQLSEPIRLMQKGQHIGKMIITMPTSGDDELELPSEGLHDPFRARSDAA

YLFTGGLGGLGKSTATWLAGRGARHLVFMSRSAGTAEHEAFLRELECLGCTSVTVQGDVANYDDVVRAVKAAERPICGVF

HAPLILKDAAFLDMSFEQWSAVNRPKVDGAMNLHKALLGEKLDFFFLYSSIVTLSGQLGQANYCAANTFVEAFVSYRHSL

GLPASTLMVGAVGDVGWVADNPDALEKLRATAQYISNEVEYLDCVELMLKRPGPCPQDLEGQGRLVQHSQIAFGVRATVP

MAAPTNRATFRKDPRLLVYRNLEAAFFAGGSSTLAQAGSEDPAQEELSRFLAAAAVSMAFLRSAEATALLARQLGRTLCG

FMLKPDDEFDVQAPLADVGMDSLVSIELRNWIRKKIGVEVNVLEITRAASLEDLGGMVQNKIVEKYEARR

>Phapo1|749699

MNAHVNGTDFPEPIAICGMAMRLPGGVRDAEGYWDLLYNKRSGHCKVPADRYKVEAFYGPGKIGHVNSMDGYFLDGYDLS

HIDTSFWTMTRQEIEALDPQQRMILEIVYECLQNAGQKPSNLRGRKIGVYVGTFDGDWLELDGRDTQHYNSYRLTGYADY

MSANRVNYEFNFMGPSMTVRTACSSSMSALYDACRSIHIGDCEAAVVATSNLIFSPRTTSTMAGQGVMSPTGSCKTFSAD

ADGYARGEAVSAVYIKKLSDAIRDGDTIRSVIRSSVINSGGRASTLTAPNATAHEALIRRGHEAAGITDFSQTAMIECHG

TGTAVGDPIETKAVANIFGEHGVYIGSVKPNIGHGESASGLSSIIKMTLALEKKIIPPNILFNKPNPKIPFDECKLVVPT

EPMPWPAERAERVGVNSFGVGGSNAFILLESADHHGVRRPKETLEHTSLEPLPKLLAFSAKHPESLKRSAADHESYINSR

PESITDLSYTLGVKREKVANSTSFFARVLAHRAFCVTDGQGTFELSRVQKSTLKSPPSIVFAFTGQGAQSAQMGKELVQN

VPSFKQSIQYLDQVLASLATPPKWKLLDEIMAPKKSSRLGQAELSQPCCTALQIALVDLLALWNILPAAVVGHSSGEIGA

AYACGSLSAEDAIISAYYRGQVTLSLSPELVGGMAAIGLGAQDVTQYLQHGVTIGCENSPESTTLTGDKAVLQKVMEVIK

ENHPNTLVRALAVDKAYHSAQMYTIAHEYTALLGKLHAGASKVPFFSSVTESIITDAHVLNESYWVKNLISPVKFTGAIG

NIVDTINAPKVFLEIGPHKSLAGPIRQILRAKSSQEPYIGTLVRGKDAHEQLLKTAGELWLNDHAISFESINGKGNFLTD

LPLYSWNYESIWGESRLSRDWRFRKHPHHDLIGSRVLESTDQNPSWRNILRSDEILWVKEHEVTGDIVLPGVGYVCMAGE

AVRRLTGTSDYTVRKVNIKVACMIQSGKDVELITHLSKARVTDSLESPWYDFSVSSMNKDTWIQHAFGQIRPGSEIERKN

FTIKPLPRQLSKRTWYRKMQQMGLNYGTRFRAINNPTSHPTIKSAVASVKSELEEGDNGLERRFNVLAVPTYIEELYVRE

PVGEMTVQATANFPPKGGAMSGDVVAIDTEGNTVVQLTTLQTSLLGDGKTDDNKGFKGLHAATELEWKPDLTFMKASDLF

SISLERGDIHNNLDKLGALAMLEAEERLKCEDTEQGHLDIFFHWLRKINANIDPALAKLDAPQRNQIMVELHDWLQSTPA

KAGATAIMRILNKCKGIFKAEVDPLDLLMDDGVLTQLYDFMQMQNLKYTSFIDLLSHKTPNLRVLEIGAGTGGTTNTLLP

FLKSQYGERTYFSYTYTDISSGFFIQARERFKEYPGMEYAVLDISKDPLEQGFEANSFDMIVAANVLHATPSIHETLSNC

HKLLHPRGRLFLQELDPVSKWINFVMGVLSGWWLGAADGRPDEPYISAERWEKELRGAGFDGIDSINHDGYLDNNIIALP

MRPILSSYRITVLCIDPKAPKVQTIASGLESKGFKLDFCKVGQIPTFEQDAISLLDLDHPFLYSANEKEFLWFVTVVNRI

KGTSENHNGVLWVTGACQVNCTEPKYALITGVSRVCRTERQMDFGTFELDKFDEKALAVVSEVYAEFARRSHEDSMTPDV

EWAYDSGKVMKLAINKPSVLSTLAWEELGLPTLGPNDVRCDVKAVGLNFKDILIAMGIITDLPAIGDGLGLESCSVVTHV

GSNVSSLEVGDRVIGCKTGSFTTSMVLDEKLCVKIPEKALLSDEEASNMPVVVCTALYALQDLARLESSKTSVGIAAIQI

AKMTGAEIYCTVSTQEKIDFLTQTYGIKEDHIFNSRDANFVSGIMAKTGGRGVDVVLNSLSGELLHASWRCVAEFGIMVE

IGRRDFIGHGQLSMEMFDGNRTFVGFDLSTIAAKRRTVIISLIERALRYNAEGHIKPITPIHKFSAAQIGEAMRYLQRGT

HIGKVVITMPENTAALPLGKVRGELQLRPEKAYLFVGGLGGIGKAVASWLVERGAKRIVFLSRSAGKDPKMQTFLHELAV

QGCNATTFAADVCNYDDVLKAVNSIEKPIGGILHAAMALADVSLADMTFEQWQYAMLPKVQGTLNLHKALESHKSSVDIF

FTFSSAGSIMGNWGQANYNAGNCFLDAFVQYRHSVGLPASSLDVGVIEDIGYVAENPSLLDTLRATGQYLIQERELLESI

ELCMSRSQPPSLKLKRDGDADKLPRYANPSQVAIGFRSVLPITAPNNRCIWRKDRRMAIYRNLERQETAATDSSNGVLRS

FLKDIVSNSTLLKAPESAELLAQAIGKTLFGFLMQEDEEVDLNAPLATIGIDSLISIELRNWIRANIGVELTVLEIVRAD

DIAALGAQAQAKLIEKLVT

>Stael1|356106

MANAASLLSPAVSLHAEQSTLESSSPKLTSFTGSFADDDRSFQPPAEDVAIVGVACRTAGGNTSAEQLWQFLLEKKDAAG

EVPAWRWEPWLRRDARNAKEIEKTISKGYFIEDLENFDASFFGISPKEAEQMDPHQRLGLELSWEALENAGIDPKSLAGS

DTAVYMGMDTDDYSRLLLEDLPNIEAWMGIGTAPHGVPNRISYHLDLMGPSSAVDAACASSLVAVHMGRQAILNRESEVA

IVGGVHVLLAPALTRMLGKAGALTPEGICRSFDDAANGYARGEGGAVLVLKRLSSAIRDGDNILATLKGSAIAQDGKTNG

IMAPNAKAQELVGHWALSRSGIDPLSVGYVEAHATSTPLGDPTEISAISAVYGSGAGRNPDTPVYVGSIKPNVGHLEAAA

GAIGLIKAVLSVKKGELAPQARLNKLNTRVDWPNTGLHVVRETTKWTNPNGPRRAAVCSYGYGGTVSHAIVEEFARSHPE

TARSDTAEPVILTLSASQEKRLPLQARALAGWLEGAGKDADLRSVANTLAQRRAHHDYRVAIAADGRCDAIEALRSLASG

SPVDSPAVSQGRALGAMASSVWVFSGHGAQWAGMGKELMGNPIFLETVSELDDLINKETGFSVADAFRNGTFEASEHVQI

VTYVIQIGLSNVLQARGIVPQAIIGHSVGEIAASVVVDEYVTQLKSRGVKTFRVKTDVAFHSPMLDQLAAPLRNALFNSI

SPRPARIPIYSSSQVDPRSTSLRDIEYWVNNMIKPVLLKSAVTAAAEDGHRIFVEISSHPIVLHSVNETLVELGIDDEDF

ATISTMKRDAPAMGSLQYAIGKLYTRGAQIDMEALFGHKRLWCPSVPGTPWVHRPYYRQVETGPLGDGATHDVDKHTLLG

QRIPVGGTGTVLYTTKLDDKTKPFPGTHPLDGTEIIPAAVYINTFHHATGGRVLSDIQLRVPVSMSDETRKVQIILQGDG

LTVASAADTAAGEEEHGSWVSHSSCRWGKLEEKDTQISDKSLNVDVIKARIGTLLPNSFSVDYLTKIGVAGIAFPWQVVE

HYGNEKEMIAKVDMDPSVEKLSWDDRSWAPMLDAATSVGSTIFFNDPKLRIVSQIDRVSLYSDEPLPKIGYLYVEEAADA

KSPAAHVTVLSEQGKPLAKFQSMRFSEVEGASGVSGSVESLVHRIDWIPPKLSEKPRALDNVVLMSADETLLQIHKRQLA

SKANKVICARSHTELERPELQEVISKKGSTVVYLPGTVVSLKDVSASTEEFIWEVASLVKVIETKGLAGTCKLFVVTDGV

YLGDSATGLAQGALYGLGRIIAAEHPDVWGGLIDTETPGIFPFQAITSVPGLDVMRVVNGEPRRPIMRPLSLDQRHKPGS

NKTLLPKPEGTYLMTGGLGDLGLETLSFLVEKGARRIIVVSRRGLPPRKQWPALAQSDAKFGRAVKRIRELEGLGASIYG

IALDISAEGAVQRLLDAIDALGVPPVLGVIHGAGVLEDSLLLETTRKSFAYVLAPKVSGALALHEAFPPCSIDFMVLYSS

IGQLVGTAGQASYGSGNAFLDGLAVHRRACGDNTVALQFTAWRGLGMATSTDFLTVELQSKGITDISSEEAFRAWMHLDK

YDVEGAVVTRCLPVEEGSAVAVPLLEGVAVTKLRAGSAGAEPSSEDSDAGARPTNPQELEKWLNVKIRECIAAVLLIADI

DDIDVRMPVSDLGVDSVMTVALRQKLQAVLKVKVPPTLTWNHPTVNHLVPWFKAKFAEA

>Stasp1|367236

MAPHALEASNGESHHLNGHVKTLNGDFDKLNGTTASRNGYARIIDGDLNGNHNSTAAMNEDINGTDNPAFPEPIAICGMA

MRLPGGVRDAEGYWDLLYNKRSGQCKVPADRYNVEAFYGPGKIGHVNSMDGYFLEGYDLSHIDTAFWTMTKQEIEAMDPQ

QRMNLEIVYECLQNAGQKPTELRGRKIGVYVGTFDGDWLELDGRDTLFYNQYRLTGYADYMSANRVHYEYNFMGPSMTVR

TACSSSMSALYDACRSLHVGDCEAAVVATSNLMLSPRTTSTMADQGVMSPTGSCKTFSADADGYARGEAVSAVYIKKLSD

AIRDGDTVRSVIRSSVINAGGRASTLTAPNTTAHEALIRRGHEAAGITDFSQTAMIECHGTGTAVGDPIETKAVANIFGE

HGVYIGSVKPNIGHGESASGLSSIIKMTLALEKKIIPPNILFNKPNPKIPFDELNSFGVGGSNAFMLLESAEHHGVGRPK

QTFEPTSLEPVPKLLVFSAKHPESLKRSAADHESYINSRPESINDLSYTLGVKREVLAHRAFCVTDGQGTFELSRIQKST

HKSPPSIVFAFTGQGAQSAQMGKELIQNVPSFKKSIQYLDQILANLPNPPKWKLLDEIMASKKASRLGQAELSQPCCTAL

QIALVDLLASWDIHPAAVVGHSSGEIGAAYACGSLSAEDAIISAYHRGQVTLGLSADLVGGMAAIGLGAQDVAKYLQHGV

TIGCENSPESSTLTGDKAVLQKVMEVIKENHPDTLVRALAVDKAYHSAHMYTVAYEYTALLGKLRAGAPKVPFFSSVTET

IITDAHMLNASYWVKNLISPVKFTGAIGNIVDNLKEPKIFLEIGPHTALAGPIRQILRAKSSQDPYIGTLVRGQDAHEQL

LKTAGELWLNDQAVAFESINGKGNFLTDLPLYPWNYESIWGESRLSREWRFRKHPHHDLIGSRVLESTNQNPSWRNILRS

DEIRWVKEHEVTGDVVLPGVGYVCMAGEAVRQLTGTSDYTVRKVNIKAACVIQSGQDVELITHLSRARVTDSLESPWYDF

TVSSLNKDTWIQHAFGQIRPGSEIERENFKIEPLPRQLSKRTWYRKMQQMGLNYGTRFRGINNPTSHPTIKSAVANVKSE

LVEGESYYAVHPSAMDSLLQLLMLAVSNGLERRFTVLAVPTYIEELYVRPPVGEMTVQATADFPAKGGAMSGDVVAIDTE

GNTVVQLTTLQTSPLGDGKTDDNKGFNDLHAATELEWKPDLTFMKASDLFSISMERGEIHNNLDKLGALAMLEAEERLEG

QTTEQGHLNIFFNWLKKVNASIDPALARLDSPQRNQVMVELHDWLQGTTAKAGATAIMRILNKCRGIFEADVDPLDLLMD

DGILTQLYDFMQMQNSKYTSFIDLLSHKTPTLRILEIGAGTGGTTNTLLPFLKSQYGERTYFSYTYTDISSGFFIQARER

FKEYPGMEYAVLDISKDPLEQGFEANSFDMIIATNVLHATPSIHETLSNCHKLLHPRGRLFLQELDPVSKWINFVMGVLS

GWWLGAADGRPDEPYIPAERWEKELRGAGFDGIDSVHHDGYLDNNIIAMPTRPALSSRRITVLCVDSNAPKVQTIARGLQ

GKGFELDFCKVGQIPAKEQDAISLLDLDHPFLYSANEKEFLWFVTVVDRVKGTSEKHNGILWVTGACQVNCTEPKYALIT

GVSRVCRTERQMDFGTFELDSFDESTLATVAEVYAEFAQRSHEESMTPDVEWAYDNGKVMVSRYHFIDVAEGMKNTTVTP

EVSTKKLAIIKPGVLSTLAWEELGLPSLGPNDVRCEVKAVGLNFKDVLIAMGIITDLPAIGDGLGLESCSVVTHVGSSVS

SLKVGDRVIGCKTGSFTTSMVLNEKLCVKIPETASLSDEEAATMPVVVCTALYALQDLARLDSSKSILIQSAAGGVGIAA

IQIAKMIGAEIYCTVSTQEKIDFLNQTYGIKEDHIFNSRDANFVSGILAKTEGRGVDVVLNSLSGELLHASWKCVAEFGI

MVEIGRRDFIGHGQLGMELFDGNRTFVGFDLSTIAAKKQAVMMSLMERALRYNAEGHIKPITPIHEFPAAQIGEAMRYLQ

RGTHIGKVVVKMPENTETLPLEKVRGELRLSPEKAYLFVGGLGGIGRAVASWLVERGARRIVFLSRSAGKDPKTQIFLHE

LAAQGCNATTFAADVCNYDDVLKAVNSIDKPIGGVLQAAMALADVSLADMTFEQWQYAMLPKVQGTLNLHRALESHKDSV

DIFFTFSSAGSTMGNWGQANYNAGNCFLDAFIQYRHSVGLPASSLDVGVIEDVGYVAENPSMLDTLRATGQYLIQERELL

ESIELCMSRSQPKPLKRKRDGDTDELPRYANPSQIAIGYRSVLPITAPNNRCIWRKDRRMTIYRNLERQETATADSGNGA

LTTFLKDIVSNSTLLKAPESAELLAQAIGKTLFGFLMQDEEEVDLHAPLATIGIDSLISIELRNWIRANIGVELTVLEIV

RADDIAALGAQAQAKLVEKLVT

>Usnflo1|900218

MGMRLPGGVHNAPAYWDLLVNGKNGRCRVPRDRYNIDTWYGPGRVTHVGTHFGHFFEELNLANIDPSFWSLTKQEAELLD

PQQRLFLEVVYEALENSGATKWRGRNIGMYVGALGDDWGEMEMQDGQDLNPTRTTVYGDYVIANRASYEFDLKGPSLVVR

TACSSSMVALHLACQDVLTGECTGAIVGGINLIITPRTTVALTEQGVLSPSGQCKTFDADADGYGRGEGVSAIYIKKLSD

AIREGDPVSAVIRSTCVAADGKSFGFTMPNPESHEKLMRRGHQLAGIHDYSKTPMVECHGTGTPIGDPREVGAVANVWGD

CGIYIGSVKPNIGHGEGASGLSSIMKMVLALENRTIPPNINFKTPNPNIPWAKARLKVPTKATPWPTDRLERVAVNSFGI

GGSNAHVILESAASFGIGQKLKSTSGANDTLRPHLLTFTAKHPQALQRTAQNYEDYLTTHPDSLSDVAYSLNIKREVHSN

RAFCVTNGLDAFEISRLSKPTTSLPSLVFCFTGQGAQWARMGRTLLEKDPIFIETFNALDEALSRLSNPPPWSLKDEILA

SPTTSRLSEAEFSQPCCTAIQVALVDLLRSWESGLIAAAYASGAITAQDAISIAFYRGQATLPLKLSHKGGMAAIGLSRE

SAEQYLLPGVIIGCENSPASVTLTGDTTIMDRTMEAIRSVNPSVLVRVLHVECAYHSDHMKAVASDYLYQLGAINAKEPK

VPFYSSVSGKITTDLSASYWVQNLVSPVLFKTAVQALLAESPTSSNLAFVEVGPHSALAGPTRQILQSEGRPAEYIATLI

RNTEAYDSVLRTAGNLWLAGVDVNLAAVNPHGNLLTDLPTYPWHYDGEYWVENRMSRDWRFRKFDHHELLGARILESADA

GPAWRCKLRIEDVPWLRDHNILGDIIFPGAGYICMAGEAVKQLHAGTTDYTVRRVTFVSALVLHGEPVELVTILVPARLT

IKLNSEWYDFSVSSFNGDTWIKHVSGQVRPGPGLVQEAPEIEPLPRKVANATMYKVWKEFGLNYGGRFRGLSDISSHTTE

QEAVGTIYDKCSPEERALYTIHPASIDAAFHLSNVCVCRGLSRNFRMPSVPKYLDEFADAIRKARGGSFSNLVGVSNGKV

VLSWKNLELAPLSDGSEVIDEDPHAAAVVDWRTDIDFIDSRRLLHSLNKDFGDEEHKLIDRMGLACIIESRVQLAGLKTS

QPHLIKFRDWMDIPYAEAVQQRYPNVPDCAAIANMGSRERTKVIKDSLEASVGTVAYSVAIALYRIFDNCVKFYTGDADP

LQVLLADDILMRMYDFANNADHLQFLTLLSHKKPTLKVLETGAGTGGTTATVLPALKSEQGERMYSKYVYSDISAGFFIA

AKERFKDYDALEFSVLDITQDPLGQGFEGGFFDLIVASNVLHATPNLVQTLTNVRKLLSPQGRLFLLELSPESSKSVNYV

MGPLVGWWLSDDGREHEPYVSHEIWHEKLLQTGFMGVDAYAFDGNMSNSIIARPVSVSTEKLTDVSVVSSDMSSPHVIEA

SRFLKEKGLDLKFFAAGQPLPSGQPVVFMLDLEAPFLVDITADQFRAFKQSLFSVNDTGLLWVTGACQVECKNPDYALVN

GMSRSIRQETGIDFVTLELETFDESGWTALYDLLTTFPSRSRGGDEDTELDSEYVFNAGTLRIGRMHWIKVSKELQDQVR

KDCTKRLVIDKPGILQSLHWKQVTPTPAAGNGVQVETRAVGLNFKDILIAMGIVETGIGSPDSGDFGFEGAGIVSSIGPD

VEHLVVGDRVAFSSVGCFATSLSMREDDCTKIPDSLSFEDAATMPCVFGTAMYGLMELARLEKGQTLLIHSACGGVGQAA

IQLAKMIGAKIFCTVGNQEKVDYLISVYSIPRNHIFNSRDSTFLPAILKETGGRGVDVVLNSLSGELLHASWKCVASFGI

MIEIGKRDFVGKAELGMDRFENNRTFVGLDHTELCSQKSQVAKRLLNQIMEFYQQKKLRPITPVKVFEAANVEEAFRHMQ

KGQHIGKIVVTVSQNQESLKAEPARRETVLRGDRTYLFAGGLGGLGQSIATYLVEKGARYLIFFSRSAGTFADSNPYFFK

ELESLGFNVQAISGSIYNIQDVQEAVFSAANPIAGVLHASMVLRDANFMDIESEVWRAAVLPKVQGTWNLHNALSKQEEP

LDFFFLFSSVSGTAGQIGQANYAAGNTFMDAFVQYRHSLGLPASTLDIGIMEDVGFLAREKHLLDALKATSLHMLHEKDL

LDSLGLMIARSHPPHDNDNTATAAKSARLTSGYVNPGHVIIGMRSKLPLLSPLNRTGWKKSPRLLVYRNIEFQDQSDFGA

STDGSLKEFLQACSSTPEILETEQAEIFLAHETGKTLFTFMMRAEEEIDTTVPLASFGVDSLVSIELRNWFRQKIGVQLT

VVEIGGAASLREIGKMTAEKLIEKHKH

>BGC0001264.1|BAQ25466.1|MIBiG

MSSSASFNEPIAIVGSGCRFAGGASSPSKLWDLLCKPKDIRSDITGRRFNAEGFYHPDGSHHGHMNVLQSYLLEEDTRLF

DAEFFGTNPVEAKAMDPQQRLLLEVVYESIESAGLCIERLRGSNTAVFAGLMCGDYEAMMLRDLDQAPTHFATGTSRAVM

SNRVSYFFDWRGPSVTIDTACSSSLVAVHYAIQALRSGDSHTAVACGSNLIFGPEMYVIESKLKMLSPDGLGRMWDKDAN

GYARGEGVTAIILKTLSQALADNDRIEAVIRETGVNSDGTTPGITMPSASAQRDLIQSVYRKAGLDPEAMEDRPQYIEAH

GTGTPAGDPIEAEALSTAFFGNTEKASTPIYTGSIKTVLGHTEGSAGIAALMKVTQAIRNAILPPNLWFQQLNPKLKQFY

GNLQIPTQALPWPTVSDRRPKRASINNFGFGGTNAHAIVESYEPEPRQTVESPDAATVSTPFVFSAASTESLRSNLAAYA

TYLDANPKTSAGDLAYTLRERRSVLPFRIAFPDTTVESLKLSITTRLVEPGNESLGVRTWTAGNRGRSRLLGVFTGQGAQ

YARMGAELVNQAVLAGQLLEKLEGYLSELPEGDRPSWSLRDEMLADGPLSHVGEAAISQPLCTAVQIILVDLLKSAKVKF

DTVVGHSSGEIGAAYAAGYLSARDALLIAYFRGLHCKHATSPNGDIKGAMLAAGTSMEDAIEICEAEEFLGRVTVAASNS

SSSVTFSGDEDAIDEIAAVLQDENKFNRRLKVDTAYHSSHMLPCFDLYVASLRRAGVKALLGNGECTWISSVYEGRSIDP

STDELSGVYWAHNMTKAVLFSQAVRAAVKIATDNDPYTAVLEVGPHAALAGPAKQNIFEALQKELPYHGTLLRGGNAMTA

FSTCLGFLWTHLDTASIDLGSCEAAHSGNKQQFTVLGDLPSYQWKHESAYWAESRKSRQMRLRNQPFHQLLGDVSPDSAP

HILRWKNILKPREMTWLEGHQVQSQVVLPAASYVSTAIEAAQSLASGKKIQLIELSNFHIHNAITFDQNDIGIEVHIEVS

NIYIKENQVHANFTYSAALGDELNDLVLAANGELKVVLVDETPNISLFPQRQAPPPHMIPVQPSRLYGFMKGLEYDFSGA

FQSLIKLERNLGHATCLAQKAKVLVPDADELLVHPIDLDAAFQSVMLAYSYPGDDQLRLLHLPTSIAKLRVNPSVLASQR

YAENDMTLIDSTCSTGDRAEPGDGFSGSVNMYAPGFDHAAIQVDRVKFKPVGSDASNDRDVFYKMHWVPSAADGMLAAAS

VLVGEQDRELMFVLSRIAAYYLRIFDEQLPENDPARSTSPLCHYMNYARHMTNLLKNGQHQWAHQDWLNDTEEDVLDDIV

AKGFMENSDVKIMLLVGNTMPRVFKGETTMLEHFRTSGLLDEYYSNGFGTKQSTLWVASILKQLTDRNPHLNMLEIGAGT

GGATKTILQSIGHDFGSYTFTDISSSFFENAAETFSDWQDSMVFKVCNAEIDPVQQGFQHGSYDVVIAFMVVHACARLDE

AVANLRKLLKPGGLLVLGEGASDGAMQAGAGFIFGTLPGWWRGADEGRTLSPLVNASEWDVILKGSGFSGIDTMSPPTLF

NAFGITLFVSTAIDERIEFARNPLAITKSTVYNKVVIVGGRTPPIVQLSREIQEALIPLAKQVLSYASLEDLDENTLEDE

TVVVSLVDLEAPVFKGITSERWYKFRKLFETKRDILWLTSGRLEDEPYCNMTVGFGRSAMHEEETLRIQYVDVTNVGNFD

AQKIAQYLLRFTSARLDDKDILYTKEPEIIIDDEGRELVPRLFTIKASNDRLNSTTRSIFDPVDINKHVVELQYGKDGPN

FRQLSRYELSEEPTTPQSDHAELRLTSSTVSAIRCPTGYQFLVVGTDQTGAQRLALTSSLTSLLRIPLESTVLCEHPGLS

EANYLGLVAAELSVIAFCDSLFTGQKLAVHNAPASIVRAVLSHVSPKGLSVTFTTDTLGTAVSPDVASQIHIPMFSARSD

IEAILPSDIVCFVDFSASIQAENVAMITSCLPSYCRKENVNTIFSPHGIDTSASTAVLGQLLNRAVNIVKERNVSTTPTL

LGLKALAHGESGTDPLTIIEWTGCTTVPARVTRFESNQLFKSHKTYWLVGLSGALGISLCDWMIERGVRYLVLTSRNPKI

DPRWIRNHERNGVTIKIMLCDVTDEKAINEVHAEIVKTLPPIVGLLNGAMVLRDVSVRNMEFDQVTDVIRPKVLGSIHLD

RIFYNIDLDFFVLLSSINCVIGNVGQANYAAANMGMIGVAGNRRKRGLRSSVVNVGAIIGVGYITQSDRQLDVTVAKTAM

MHLSEQDFHQIFAECMEASHLDSPNGPEISTGLLSITPETIDIPPWYSDPKFARFRVHKAADTGDKSDATNSASTQDLLQ

ACRSQIEVANVIKQAYCTQLRKMLQVSTVDGDLMMMRGVDLGFDSLLSVDVRSWFLKNFRVSIPVLKIMANDVRMSSLVE

LAAESIPAELVPGVPQANANPNGPSSPDSDATESSNQNSDVDVTSTRATSPSTPAATSPDSNVKIKTNSSFAVDWKFETI

PPEPFALPGLSDAPKPRENPEVVVLTGCSGLLGHHLLNTLIAQPSICKIICLAVRRLSSRLESGDLPAPSERICYYEGDL

TSTYFGLDTTTWTSIFHETDAVIHNGSDTSHLKYYSALKQANVESTKQLVSTCLQRMIPLHYISSAGVALFAGLAAFPPI

SCTQTGKTPPADGSHGYMCGKWVCEKMLERTHEKHRLRIVIQRPSTIIRDGKDATVERAGFDWVNSLLHFAHKTQTVPRV

EFNAGAFDLVSVETCCEDVVRELPNRGREGITYVNNVGDVVIPMAQMADVGLSKVEKRYSVLPMEEWTKIVVNAGMHPAV

AALIETFDEPGVEKYPALLRSEDA

>BGC0001243.1|CBX87032.1|MIBiG

MSFPEPVAIIGMGCRFPGDSDTPDEFWKMLAEERSGLSRPPLSRWNIDGFHANKARPGSLTPEGGYFINEDIWKFDPAFF

GIVQEEAKAMDPQQRKLLECVYESFESGGITLSQLSGSNTGCYIGNFTSDYYLQGHRDHNNPKPYSLLGSGYTIISNRVS

YLFDLCGPRALQTKEIDAAVVGGTNLMLAVETQMSTDKVGVLSATSTCHTFDESADGYGRAEGVGAIFLKRLSDAIRDND

PIRGVIRGTATNANGKTSGITQPSAKGHETVMRTAYEFAGLDPRDTSYFETHGTGTQVGDPIEIKGVGNFFFNGTDRQKL

LVGSVKTNVGHSEAASALASIIKVCLAMEKRTIPATIGIKKLNPKIDFKGGRIEVVQKMTPWPKGFSVCRASINSFGYGG

ANATAIVEAADSVLPGKLTYTRRGQDERDLEASSEESESVEMVGTKSASQVRAVSRSEFLLLFSAHDISTLKSNIERCRD

VAEDYNILDLAYTLGCRRSNFFNCAYTVAREDDVEEDLMEHEITFGKRGNGGNIGFIFTGQGAQNAQMGRELMLTFPSYI

DTIRKLDRSLQSLGDDSPDWTIEDVLMEPAVTSKINDVEISQPVCTAVQIALVELLRLWNVTPVACIGHSSGEIASSYAA

NLIPAEEAIISAFYRGRGVGTLKVKGTMLAVGAGPEEIQPYLTDGLRIACYNSPNSVTLSGDIEPATIVQKKLESDRVFV

RELKTGGRAYHSHHMLNIGNDYESRLSEALSRFGASQTTNQAQVQNPVFFSSVTAQQMPSNFKPGPSYWRQNLESPVRFT

EAVEAALAADLGISQFVEIGPHSALAGPLRQIRDNLGITPKDLDYAATLVRGQSSVTRLLDLAGTLTMRGFSVNVERVNA

IEKREGGAIITKTGLPIVDLPRFSWNYSAGEIRNKNRPDEEHRLRKFKYHDLLGAILPGSSVEQRQWRNMLDSKNFPWLE

EHKLGPQPVLPGTGYLAIATEAARQFFHDKLTVSGAFRYFFPNISITSALNIPPSGSQVEIVTTMKFATITASITSKTIA

EFTISSIQAGNWTNHCVGTVTKKKAVTMAPRFDESKLQEPKAARTWYRGFQKVSLNYGPAFNGLSNIRTNPALEEAVADT

ELCPDGVSEHDSAYIVHPAAMDTCIQVALIGAHKGSLAGLKRSFVPTSMANVSLWSWADDDHITQLTPGKGKVLAHAEFF

SLRAMNGWCQLFSPEGKPLFEIEELSCTQYSEALDDLGTIDRHPYLRTVWKPDVDKMVSNLTDNSLLDLIVHKRPGFNIC

EILNTESMISDNLHRVLESGSSLRRYKTYTVMALGDVDIEPIKVKYEAFPGVVVQKLVLDDVPETFFDLLIVPQFPSDEI

DLAKLKGLLTPGGNMLLYSSEFKNGNTIKTDLQLAGLYTLLEDKESLLISPHQRTSSDLPAGEIVLVTRTSPTVFDSHIF

SGLSKSGRLITSISLQDFKFHAHTKATYVFIVESESSIFHGSLTSQELATIQSVASGATNMLWVTHGNLLEGDDPNAGIV

IGLGRCLQTEHPTLTFKTLDLDHRDPIQTISNITTILNAADSGDEDKEFMVKNGIFYVSRLSQDPLLDQQFVSGVESEPK

MIPYEPEKRIRLGIERVGIFDTIHFKDDELEASLKPGEVEVDVKAVGLNMKDFATLQGTYNSEILGLEGAGIVRSIGTGV

TNVAIGDRKLKPEETLDQMSSIMMPFLTAIYGLIYLAKLQPGESVLINSATGGVGLAAIQIAKMIGAEIFATVGTPEKKK

FLMQEYGLQDDHVLSSKDASFAAEIMQTTGGRGVDVSLNSLVREQLRATWNCIGHHGRHIELGQTDILDQGILDMSPFKR

GASFIAMDLVLVFEHKPDLISQILGEIMHYYRDCKIQPLPNLSVFPVSAIGKAFEEFGKNSRIGRVVVSFDTETINTRLS

LRRRRSQLLSSQMVHTYLLGVLEALVDVWLVTWLKKGARHLIFLGRSGEDRPEAASMIKDFRNDGITVDVVKGDVTRISD

VQKAVDLAAGPLFGVVQGVMALDDRLFTSHDLNSWEYAVRPKVTGTWNLHNAVASHSLDFFVMLGSSSALSGFPTQSNYC

AGNSFLEFFARYRQSKGLPATTISLTVVTEVGFVSQNERIEDGLARTGVHTINEAGVIDLIDTAMMKAPSSSWNLDPLAN

SFIVTGVEPLQLSANLDIDSIPFWRQPRIGPVFNAVLAKKSGNETGPGQNKRRLLLPDILEMIIEKFSQTFNVAVEDIDP

GTEIVRFGMDSMIGTSLRTWCYKTLGADIAASDFMSLNLTADSLAKKIYDIRKG

>BGC0000003.1|BAD97694.1|MIBiG

MDPQQRLLLETTYEALENAGIPQANTNGSNTSVHVAMFTRDYDRNVYKDTVGIPKYQVTGTGEAIMSNRISHIFNLHGPS

MTIDTGCSGAMTAVSQACMSLRSGDCDIALAGAVNLIMSPDHHISMSNLHMLNAEGKSYAFDSRGAGYGRGEGVATIVMK

RLDDAVRCHDPIRAVILDAVINQDGYTAGITLPSSEAQAQLERKALNRVGLKPQEVAYIEAHGTGTAAGDAAELDALSSV

FCVDRDLPLYVGSVKSNIGHLEAASGMAALIKATLMLENEAIPPSINFSRPKENLRIDERNIKIPTALQPWPKGASARIC

VNSFGYGGTNAHAILERAPERPTVMGPKNTPYLFLLSAKSRASLSRTVKNIKEWISSQHDTLSLRDLSYTLNQRRSMMSW

RFGGVATTHQELLDVLTQELKSSSAVRTPTRANINFVFTGQGAQWPGMGRELLVVRAFKDSLNQSRNVLHQLGASWDLFD

ELVRDKESSRLKEPQLSQPVTTAIQIALVETFRSFGISPGAVVGHSSGEIAAAYTAGYLSHDTAIKIAYYRGFSAEIAKA

KGMENGAMLATDLGEATAREYVAKLVKGKATVACQNSPNSSTLSGDTTAVSELEEMLSKDSVFNRRLQVDAAYHSHHMEA

AAEEYEKSLGDVCVEQPLTKVRFFSSVVGREVWEGFDSTYWTTNLTSTVRYCDALQALCRTQFAQPQGEQSHQLFVEIGP

HNALAGPTRQSISDLDKQSTYSYMSALVRGSGGVGTILGVLSELIKHGHHVDLAALRTLDPTCQEANVLHDLPSYAWDHS

KRFWNESRLSREYRLRKHPYHDLLGLKMTDHTPLRPSWRYLVGVEGLPWLKDHIVDGTIIFPGSGYLCMVMEAAETSFTK

ALIIPESPSRVELQLNFCPVGPTNGNAFHFVITAVSAAGIWAEHCKGSVEVKYAAANRPRKALDIPVTFDQISEGLDVES

EAIEKISSQELYDELSAVGNTYGPMFRGINKAIIQADRSASFISIPDVTRMMPAQYMRPHFIHPTTLDILLHSSLPLVNR

QVGQASIMPVRIDELALSTLIQNESGSSLAAITTLTSADLRGGDADILVFSDSGDATDRPVMSVSGLELRRLAPTGQPAT

SGTARDICYEMKWDADVEFISAEFLRPQKLPPSVKQKWDVIDRATDIYIQRCLQHLGKRALDASGDHHKLLVKWMNSTVA

KTQTCEDPTEAKILEMSSSQGVEGEFLARLGPALPEIITGKVNPLQLMLEDGLLYRVYADDSSKRCYDLMAGYLNSKSFK

QSGFAVLEIGAGTGGATLPFLQSLDHNGNRPVVFDFTDISAGLFESAKERLQDWSDVVNFRTLDIEKNPKDQGFTEGFYD

IILACNVLHATSSVDSTLSKVRQLLKPEGVLLLLEVTKPRHYHNVTFGTLPGWWKGVNDSRAAGPLLSPEGWSTRMRKAS

LNMQLAVYDDNETPISSLIVAKPIQEVTKKKQVQIVLDSSVPIWLRKFADQVLSRLAAEEFGVSLTSWDEMTVNPHDSSI

WLVIDNGEHPVLSHVTPIQLQSVTEMLKAPSHVLWISVVHDPQFSENPFKHLITGISRTAHAENDRLKMITVDVQQSICQ

EEGKEEGNRFMSFLMGVVISLSKADLLTIEREYVYKNGQVNIPRVLPSPDIQGWMPGNVTGLPEMKPFHDSQKAWILDIE

RSEFMKMPVFTENDAFRESLDENEIEIDVEAIGVPELLIRHSINGFAGRVIAIRSKVDGIKVKDNIVAFAASSYPNRLRV

HQSQARVVPQGVSSRIAAALLIPLMAVSHALVNIASTNSPIVLIHGATGTIAQSSVAIAKALGSVIIQTVSGDVESPALD

DVVSTFADHVVPDQGYSSKHQLQKVLRQRKVDVILSFSKNRVSKEVAGTLKPFGHCIHIENGPKPSLQIEQSQYLSNATI

SRFRMDAVVRAQPEAVAFAFSTVIDALGSSKMDSKAVNVVSRPVGELDRLFKQEYQHHRNESTVLHVDDCLVRVWSSEKR

SLSLDSDATYVVSGGRGDLGKRFIRLMCAAGARHFVTLSRGVSSSHTQLTSLQTELQENVRNDCVLQDIQCNIADLNEVQ

NALAIIKTQGLPPVRGIIQAAVALEDSTMNSITSDSFNRVLGAKAHGTMNLRNTFAPEGLAFFISLSSAVTVIGTSGQSS

YNAENSVQDALAQFSNRDGCHYMSLNVGTIEGADATADNQTRVQALRRQGLISITPDELLGFFRYSVTSEARKGHRCRQA

IIGFTPESLSLTTAANGTVHSPMFTHVRERGDRKTSEKRSGAKKTFKATIQETRDFEKISQLMALWIGEKVANLVAADAS

EVDLGSSIADFYVDSLIIIELRNWINRELQASIFIPETMESQNLLSLGAKVASRSALVPSSISSKVSNSNDEALSIDSTA

SLSLAPSSQPPEMLETPYAQLQHLPAADLHTALDMLIESRKGFCTQAELEETLRASAEWRGVEKADRDAIVSKFTGSNLR

LESYEKALHLERREPLQDHAVFYLGHITDQVPDHTQAERAAIIVHSTLSFKHQLEMGVLEQNSLNGSPLCMSTLQWLFHA

TQEPRHELDVMKKYRASGNVAIMRRGHIFVATVHDDDGLAALVALFEDVIQHSEDAIPALSILTSHRRDDWAQLKGSLES

ITGNAAKLEAIQSAAFVICLDEGAPTNPGERATSQLLNDRHLSNRWLDKTLQFSVAANGVSSLIGLNSTLDGLSVKQLHE

AITEQILASTRGHMDILHQDHERRPAKRLSVFRELGFEIPPPITTAIEEKRLRNLAHYPSVAAFSQHYADLNRTFLGTRR

LRSKGTVLMAIVFAIRLFYGRFEPVWETVTLAKYARGRTDWLQIVTPDVMEWIESAIQRNSGGKSTICGRDMLVQLQAST

TKHTQNVRQVADSRGFVEPLYAFQAFIESEGRKLPRLFKSEAWKHSDRNATPKLVKTDCLGSGGWLRMQEAGFLMPHPNS

LFIHYEVHHTDPLVLVQGRDRDVAKFSGCLNEAVKAMRTIIEQSS

>BGC0000146.1|BAJ09789.1|MIBiG

MTSQYGTNGASADPEPIAIVGMGCRWPGGVRDASSLWELLKNKRSGYREFGDHRFSRKGFHHPNSEHPGTVATEGGFLLA

EDPRLFDHAFFGIGSLEVETMDPSQRKLLEVVYEAFENSGEPWDSFSGSTTGVFVGNFSSDHLIIQGRDTDHPRPYASVG

TGTSILSNRVNYIFNLRGPSVTIDTACSSSMYALHLAISAIRNGDCDSAIVAASNTIIDPSTXLMMTKLGVLSPTSTSHT

FDSSADGYARGEGFSALYLKRMSTAVDGDYPIRALVRGSALNANGRTGGITHPGREGQEAVIRKAYENAGNLPMKDTTFF

ECHGTGTPVGDPIEISAIGNVFGSATTPEKPLLVGSIKTNIGHTEPASAIAGIMKVVLALENGFIPPSIGIKKLNPKLDL

KGGRINILTENTPWPDGRVRRASVNSFGYGGANGHCIIDDVRTVLPDYKKRTANTSIGHINGHTNGHTNGHTNGHTNGHT

NGHTNGHTNGAHASDGHNGHHQNGMNGNSASHMSEKADKVHYPFSYKPTLVKDFNAKPRRRVLIPFSAHNEASLDLNITA

ISEAIKRENLADVAYTLAAKRSRFMQRTFRIVDSESPANGFAVKEKVLASGTQTARLGFVFTGQGAQWHAMGADLFEYAV

FRTSIEYLDSILASLPTPSAWKIEDILAGNCDPNDIHKPEVSQTVCTAVQIGLVDLLYTWNVRPSAAVGHSSGEIAATYA

AGRITAAQAIAAAYFRGQAVSKNKSKGLMLAVGLGLDKAEAYISGLDSSVRIAAINSPDSVTLSGDESTIKDVAAKLNED

KVFNRELKTGGNAYHSHHMLALGEFYNSTLSEGLDYVKSLGQAEPSQLYATRPWMSSVYPSKSTENPPVSPSYWRANLES

PVRFSEALANMLNLPDPIDVLVEIGPHPALKGPVGQISRSVDKSLPYFPTLNRGTNGGISLLQLAGSLFSLNAEVDLTAV

NAVDVISANQLKLVHGTTATNLPPYQYAYGPVIYHESRFSKEFRGRDIVRHDLLGSKLPGNAKLRPQWRNILRLKDLPWL

NDHKLLPYPVFPAAGYIATVIEAASRIYNEQSEPLDITGYKLRNVTFSSAMRLPDDDFGLEIITSLELADAANPKAPTWA

TFSISSVAREAGTWTEHCSGRVRVIAGTSVANEKMSTEMDARTLDTKAWYKKFAEIGLGYGPTFQPLSNIRADPSKGLAV

AQLALHTTRDTVEGGESNYPLHPASLDAVFQLSLVASHGGQIDRVCNAFVPVHIDQLYVRNGVSQDSAVAIALGSMKGLR

SAHAKLQVLDKSEQVVLDVGNLRCVTYTEVLPSTGADKEAFSIPFLRLSWKPDIRAMDNEQVQRRFPPPTENVEKAYLFD

KLERLGTLYVAEIHERYAGQGQFSSAPAHIDNFLSWVRRRMKDDNKWVAEANSLTSSQRGILIKELFAEVGHISDVKIAN

KVFNNMEDILNERKTGLEVVIPDNLLHGMYEDGLIMTGAYPQLVRFFDLFGYANPNMRILEIGAGTGGATRKILKTLIGP

HGIKRYQDYTFTDISSGFLAQAREAFADFQDMKYSVLDIQENPLEHGYEAVYDVVVACECLHATPSIVKTLTNCRKLVKP

GGRLVVVENTRAVIGHGLVLGHLSGYWDGIPDGRVESPFLHLEGWNASLNQTGFAGAELVLDDYPAPYTTARTIVSSAVE

EPAKVGQSPNGTVHLVHGDNRPELLSRIEHELTERGTEFKVISIGDVETHLPDNSRTVAFADSKSLLVNASENDLKSFKA

LIRKSANLVWVTFGGIVHGHDPDASITTGLLRTLGTENPASQFLSIDVSPDSDFQEIRLTRTILDQELALSDRIAGESRD

YEFVWQEDCLWVSRLVPDVALQDKLELSESRPSRAEMLPLDSQGSVQAAFETPGLLTSLYFKPYEETWKSLPDDWIQVKV

AAVGLNWKDLLTSAGRFDMNTFSSEYSGVVAQVGLNVTNVAVGDRVYGYGRGHFGNYVRAPANFAYRMLPGEDFVKMATI

PLVGMTAIYSFECVTQLKDQERLLIQSATGGLGLSAIQLAKAKGAEIFATAGTQEKRRYLIDVVGIPASHVFSSRDPADF

AKLMEATDGKGFNVILSTSSGELLYDSIKMLAPMGRIIDVGRIDVQNSTSLALELFKRNATFTSFDLAVADDADRALGPA

LMKAVNKRVRAGQMGPLSSITTYDVSQLDQALMAFSKGTHVGKLVVTFQNPDALVKMVPAAPHAQFARNANYLITGGLGG

LGRSIVNFMAERGARHFTVLSRSRKINSEGQMLIDKLATSGTVVECVSCDVSDSKDVARAVQDAAVVRPIKGIVHAAVSY

QDLSFDKLAIEQWTSALAAKVQGTKNLHEATKTHALDFFLMTTTIESFVALATQSAYTAANNFQDYFARWRRQQGLPAST

VSFGLIRDVGHLSTNSTTLALMARNKVMDISEYNFLRLLEPAFLNNESALDPAASKEPYTGAVDDPLSVTNVVTCFDPAT

MATRKREEAAENNGNTGNSPRWYTDARVSLIMRAFDDAERYQASAGGGGDGGNERGNNAGVASLRSEFGEAVKAGPAERS

RTVALVTDAIVKTVAQMLFVDASGVDASRTVADYGVDSLIAAELRNWFNVAFGADVSMLEMLDTATSMKILANKIVDGAL

A

>BGC0001373.1|BAV32159.1|MIBiG

MASPIPLAVVGIACRFPGDATNPEKLWDLLAEGKSAWSRVPSDRWNEEAFLHPSPDDMNGSHNHLGGHFLRQDVGEFDAG

FFNVLPGEAAAMDPQQRLLLETTYEAIESAGIPKESLAGSKTAVYMAMFTRDYDRNVYKDMMSIPKYHVTGTGDAILANR

ISYLFDLRGPSMTIDTGCSGGMAAVAHACQALRSGVSDVALAGAANLILSPDHMVGMSNLHMLNAEGKSYAFDDRGAGYG

RGEGIATLVIKRLDDAIKANDPIRAIIRDAAVNQDGHTAGITLPSGQAQEALERQVWSNIGLDPREVGYVEAHGTGTQAG

DSAELEGISRVFCRGRTDSESLTVGSIKSNIGHTECVSGLAALIKSILVLEKGAIPPNVNYQTAKPGLDLDKRKLRVPTT

LQKWSQPGVPRVSVNSFGYGGTNAHAVLEKAPETQRDSASDSQEDVPRLFTLSAASQSSLQDMAASIAGWVSQERDSQPL

PTSRLQDIAYTLSERRSLMAWRFWSVASNEHELVDSLYEASRSTENISKISSSEPPPKISFIFTGQGAQWPGMGRELLQS

NAVFAESISRSNKILAGLGAAWGLVDEILRDKGPSRLREAELAQPATTAIQIALVDLARHWGIVPDSVVGHSSGEIAAAY

AAGYLSPQQAITAAYYRGFSSAVARSKGLGKGGMLAVGLGEDEVAPYLARISPENGEAVVACQNSPKSVTISGDDAAIAE

LSELLTKDDVFNRRLLVDTAYHSHHMEAAADEYRSSLGDMEPRNSTGTINMFSSVTGSLKTDDKFDANYWVSNLVGKVRF

RDALQALCQHDQTSSSPQTHRVFIEIGPHAALAGPLRQSVADMPTPLPHSYTSALVRGTSASQSALSMAGSLFSRGYPLN

ISALNSASSLSSSSSPSVIPNLPTYAWDHTKRHWHESRLSRDYRMRKHAYHDLLGLRMTDTTPLRPAWRHMVGVEGLPWL

RDHVVDGLIVFPGAGYMCMALQAAEQLALDLPSHSKVKRMRLQNVAFLKGLVIPDSTRERVEVQLVLTPLTGDNGPDNKL

GYSFLVTAYTADDDKWTEHCRGSVLIDLVSSSAASSQSTVGFESQHQITYAEAVSSLNLQPGEDIPPSELYQTLRKGGNA

YGPTFSGIQVFRLLPDNASEDTSSANVALSTIAIPDIQSIMPAKHMQPHIIHPSTLDVLLHTTLPLVSRRLGVVGSVMPV

RIDDLVIDLGEQQLETKTDAQLRAITTLTGSGFRSAEADMLVFPAPSSEVDQGQLMPVISVMGMELRSLAALDGAAAGDP

ATENLSVEESRDICYEMKWVVDESFLSAEHLVNAVQQRDSSFDTPLERCLGALDKYLGIKALKEFMADLKVLEIAAAGDS

NGECTLAFLDALQTRGALPAEYDLTAQPHGDALWQDKYPGVVTVRPLLDSMNHGLDGHYDIVFAAGSLNGSDGVTVQTSL

SNIRSLMKPGAVLVAIHDTNSSLSADVFSQTGFNTQLSIPFDELSLTIARAVGSASTTTPVKLQFIAEPGFSTSTSIRTI

IDNLPSTLTAKGVQIITAPEPCILNWTKGNLYDQDSDSEPVTLNIVLDNGASPILPTVTNGTPTFSNIVSLLQKQHSKVL

WVSLSDDEQHKLNPQKHLITGVARTAHAENELLELVTVDVQQPISESTTSGLVNFLGDIAASFPGLDGAAGETGTKKERE

YIYIGENHILVPRVMSSPSLNRQIKKSKETVTSTENFVMTPLKLDIAGKDGPGTAHAATFVEDESHRQPLGEDCVEIQAK

AFGVGSSSWASKGRPSSASSIAEYAGVITAIGSGVLISSGLKIGDRVVAWAETSLSFASRPRIPASQVRVLPDHVSLSTG

AGLPVSLMTACHALREISNVQPGQVVFIDGAASDIGQAALLVARYLGAKVIVAVSTSDEASFLQDKFGLPLANILPRASP

FLRHQLRKLLASGGAIDAILSCAGSAVPGEIIKTQKPFGTLVQVGGTTGAMTASAVNSTVVSLDLGSFLTQTHPSKATRL

FDMAMETVHRGLDLEPIRIAYLPMTNLNEALKSARRHENMTKYVVEVGQEAMVKVARPSYILPKLDEHATYVVAGGLGDL

GQRFLRLMAKAGARYLVTLSRSGAREGQQSALERELNSLSPLSSLNLLCLKCDVSKEAKIQNSLAEIKAAGFPSVRGVIQ

ASLVLGDSTLDNMTAQDFDRVLQAKAFGTLHLQRVFVPEGLAFFISLSSAVNMIGSAGQANYNAANSLQDALAQFDKSSD

CFYMALNIGLIEDATVNSDVIIQSVQRQGLTTIYHDELDAYFEYSLSAEARQAGCHQAVIGFTPESIAKTSIVNGTAKTL

MFTHVRRQISKQGQTEDDDAGGASGAVKTFAEFVAQGTHEQDDIEAFAARAIANKLADLMLIEPEDVELDESLNDFGLDS

LIAIELRNWIMRELGSPIQTSEVLGSENIWALARKVTLRSVHVTGGAGGDASSTGNSESMARTPSDSSTVPTSIPATPSR

SPSREPPAKETLTKSQQHLPIPDLTETLNMLVESRTAIGSLEEKAEIERVIQDFLTTDGPELVEILRSNNDSSSDARLDF

YNNHLHLERREPLQDHALFFIGHLAEGEAGAAPPPKHTQAERAAIITGAAMHFKQRLESGSLEQHKLNDIVLCMDTLQWL

FHTIQEPGVATDLAQKYPSNNKVVAMRKGHIFEIDVHPEDDYAALHQIFSDIIASSDSSSDSIPKVSVLTTKPRHEWAVL

RSQIQSLSPTNAETIDAIESCAFIVSLDHSSPETTSERSTSILLNDLHLSNRWLDKMLTFTVASNGVSSLLGENTMLDGL

SARQLSEYMTNEIFTNPKLSPPTSPPASTIRPLLFTLTPHVVETISQQIQHNLSTYHPISSSRHFYSQLNRAFLGSRGMR

SKGTVLVAIAMATRLFFGHYEPLWETVTLAKYKQGRIDWLQTLTPDMVAFVDSLIAIHSHSLTSSSEVDWKGMNKLLKEV

SISHVQNLQRVADGRGYVEALYSLMGTAISQGHDLPELFKSEAWKQTDRHLSPKRAKTDCLGSGGYLRMQEGGFLMPNPG

SLFIHYEVHHRDPLVNVSGREEDVARFEGILGACLGVVRRVVEG

>BGC0001190.1|EWG54266.1|MIBiG

MTLSNGSNGANGTSNGHGAHPSANGFHNAANGGANNGTPNGGAEYNASLPQVDGDISSAIAVIGVSGRFPGDATSPRHLW

DLLKEGRNALSDVPESRFNIDGFYHPDGGRAGTLNTKQGYFLKSDVDKFDAGFFSITPEEARGMDPTQRILLELAYEGLE

NAGLKIDEVANQHMSCYIGACQHDYWDLQAYDMDSAPKYTATGTGPALLSNRISWFFNLKGPSVTIDTACSSTLTALHLA

GQSIRNGESDSALVGGLGLHLLPNFGVFMSSMSFLSADNKCHSFDASANGYARAEGGGFVVLKRLDKALADGDTIRAVLR

STGSNQDGRTLGITQPSASRQEELIRATYASAGLTFDKTNFFEAHGTGTKVGDPIECSVIGNVFGKTRERPVYVGSVKSN

IGHLEGASGLAGLVKTIYSLESGVISPTYGLENVNPKIKLDEWKINLPTEKIKWPAGLRRASINSFGYGGANAHAVLDDA

YHFLKTHNLEGHHNTKAEDVPATGLIGNGSQDIIEKTDKKPRLFLISSHEESGIARLSQTLQAYLADPAARDLPEDQFLH

RLAYTLSEKRSSLPWKTYAAASTIEELQQALDGAPTKAARVPRSQALTFIFTGQGAQWFAMGRELQKYPIFRQSLHACSQ

YLKDFGSTWDLVEELNRDAKESIIDLPYVSQPSCTALQLSIIDLLASWGIHPQVTVGHSSGEIAAAYAKGAFDKEAAMRI

AYFRGHLTGNITKTGSMAAVGLGPERVSEYLSRVTAGKIVVACINSPASVTLSGDVEGIDEVLTFLQADDIFARKLRVTT

AYHSHHMQQISEEYLNSLSGKWELKPGNPKVRMFSSVSAKPIDGTELGPAYWVANLVSPVNFSGAVTAAANAGALGKRKA

SGKKGSADAMVEIGPHAALQGPLKQILDSIGDKGASPKYFSAIKRKQDAIQTTLEVVGELLVLGHQVNVPLVNAYTETTS

ALVDLPPYAWNTTNSYWHESAAVTAYKQRKHPRLELLGVRDPRSTKAEPAWHNYLRISEQPWIEHHQFQNTNIYPMAGMI

VMAIEGLRQVETRTDVEGYTIRDVNIGSALVVPLDQTIETRLQLTPWRSGPNVSWSHWTEFTVSSRNESGSWTTNCTGLV

STSYKRETNSTFLDEEAAANALLSQEYKAISNSDLPSVDPTVFYTKLDESGFSLGPAFRGVKELNLFDHKAHFSMEVIDT

KDFYPKKWEPAHLIHPAVLDVFVHLLISSTGDAAEIKARVPVSTASLYISADFDSTSGTKYHGFSTSKKHGATNMLSNVI

AFAEGGSKPLIALEGCKTVPLRGASDPSSGDGQSLGHVPVVPKKVVDVDISDAVTLEKLLQGTDFASKLGSYLSLLGQKR

PGLSVLEYSSSTSSILLRALTAQAEELQGSITSVALTTPLDGPADEETSVPEAWKNKVQQEKLDLTQDPSTQGFEDVALD

VIFIDVEEQGDISLVLKNAKKILKPSGILLITNHASAISTDLLTSTDLTSTTVSELIIARHKPDTDPSDHQVLIVTPPSP

SSGLSKLIAQAENDLTSQGYEVNKADFANIPEQTTPFLTLSALDVDTPFLESFHHETFTKLRSLFLASRGTLWLTLDTAS

RGLVNGLGRTIRAEHPDISFTVLSLDALTSLDSALNTKTISSIIENMSRKTFGETSDSEYVIRNNQVLVERLIPNPDLKA

LLDSSKTGNNLSAVKVPLKQVNKPLQLSIRDPGLLDTLEYLSVPDLFEPLGDNQIEIEVGSVGLNFRDVMVAMGQMEDNT

LGIECAGVVAKVGAGVQKFKVGDRVFGMHAGCFQTRVRVDPRTFQRTPEHLGDEEAASLMCTSATVVHSLIDVARLQRGE

SVLIHSAAGGVGQAAIRLAKYLGAEIFATVSSEKKKRLLIEDYGVKESHIFNSRDYSFADGILRLTNQRGVDVVINSLAG

EALRRTWLCVAPFGRFIELGKRDIYDNSGLDMRPFLDNITFSGLDILTQVISYPDRFEAIGNQVVELLSKNAISPLNNLA

RYSFGEVSKAFRLMQSGGHVGKIVLYPRPDDIVPIVPEGLESFCLPHDATYVLIGGLGGIGRSVTRLLVERGARHLVFLS

RSAAARPEAQALLDELHAQGVQAKAFAVDVAEKSQLEPVINDVKQSFPAIKGLIHCAMDLRDAVYSNMTADDWNASLRPK

LLATRNLHDLLPTDLDFFICLSSIAGIIGSRGQANYNAGNTYQDALAHHRAASGLAATSINLSLVVGIGVSTERSEVFQL

LKDGGLLGMDENDVLNVIKAAISGCAPTQVALGASTGGQLDKLAANDPYWFADSRFAVLNQLDRQGTGAVAGGQDWKKLL

AAAASPDEVYEIVLQQLLEGVSKIIKADVEDMDSRKSLPALGIDSLVAIEIRTWLLKEFQADLSVFDIVSNDPLTGFAKK

VMAKSVLIA

>BGC0001400.1|EAU29808.1|MIBiG

MAHMEPIAIVGTACRFAGSSSSPSKLWELLQNPRDVASEPPADRFNIDAFYDPEGSNPMATNARQGVSCAAPQYGSVGVA

RNNLANRISYFFDWQGPSMSIDTACSASMVALYEAVSALTRHDCNLAAALGANLMLSPQMFIAASNLQMLSPTSRSRMWD

AQADGYARGEGVASVLLKRLSDAVADGDPIECVIRAVGVNHDGRSMGFTMPSSDAQVQLIRSTYAKAGLDPRSAEDRPQY

VEAHGTGTLAGDPQEASALHQAFFSSSDEDTVLHVGSIKTVVGHAEGTAGLAGLIKASQCIQHGIIPPNLLFNRLNPALE

PYARQLRVPVDVVPWPPLSPGVPRRVSVNSFGFGGTNAHVILESYEPAEGLIKVDCNQNAVLPFVFSAESDFSLGSVLEQ

YSRYLSRNPDVEVHDLAWTLLERRSALMHRVGFWAPDIAHLKRSIQDELAVRKGGAPSTLICRPHGKTRKHILGVFTGQG

AQWAQMGLELITTSNTARGWLDELQQSLDALPEPYRPGFSLFQELAADSATSRLSEALLSQTLCTAMQVIWVKMLWALNI

HFDAVVGHSSGEIAAGFAAGFLTAEDAIRIAYLRGVFCSAPGSSGEGAMLAAGLSMDEATALCEDVSSSEGRINVAASNS

PESVTVSGDRDAILRAEQLLKDRGIFVRLLRVSTAYHSHHMQACSQPYQDALRGCNIQIQTPMSTTTWYSSVYAGRPMEE

GSVTETLGTGEYWAENLVSPVLFSQALSAAMSATNPSLIVEVGPHPALKGPALQTLSGITPAEIPYIGVSVRNNSAIESM

ATAIGAFWAHLGPQAINPRGYLALFQPNTKPSVVRGLPLYPFDHRQEHGYQTRKANGWLYRRNTPHPLLGSLSEDLGEGE

LRWNHYLSPRRIRWLDGHRVQGQIVVPATAYIVMALEAALALAVVKEKSLHLIRIDDLIIGQAISFQDERDEVETLFHLP

PMLENRDDNTAVGRFRCQMAASGGHIKTCAEGILTVTWGSPQDDVLPCPVFPSPAGLADVTDMEEYYASLRTLGYEYTGV

FQGIHSLSRKMGIATGQVYNPALTGFLIHPAVLDTGLQGLLAAAGEGQLTTLHVPTRIDTVSVNPAVCSIDSLSFEAAVT

RTGADGIVGDVELYTAANGPGAVFFEGVHVSSLVPPSAADDPSVFWVQHWTPLVLDVNRSESRLSPEWMTVLEGYERRAF

LALKDILQEVTPELRATFDWHRESVVSWIEHIMEETRMGQRASCKPEWLGQNLENLGHIWGRPDASIEDRMMYRVYQNLL

PFLRGEAKMLDALRQDELLTQFYRDEHELRDVNRRLGQLVGDLAVRFPRMKLLEVGAGTGSATREVLKHVSRAYHSYTFT

DISVGFFEDMLETLPEHADRLIFQKLDVGQDPLEQGFTENAYDVIIAANVLHATPALHETLRNVRRLLKPGGYLIALEIT

NIDAIRIGYLMCAFDGWWLGREDGRPWGPVVSASQWDSLLRETGFGGIDSITDRAADELTMYSVFAAQAVDDQITRIREP

LTPLPPQPPFCRGVIIGGSPNLVTGVRAIIHPFFSDVEHVSAIENLTEGAPAVVLMVADLSDTPCFQSLTESRLAGLKAL

VKMAEKTLWVTMGSEAENPFLCLSKGFLTSMNYEHPNVFQYLNIIDPADVQPVVLSEHLLRLAHTTQNNDFTLTSCVHST

ELELRLYPGGILKFPRINASNVLNRRYAAARRPVTSPVTDMQESVVVLGQGPDGKLQLLLGEERLLGDRTGVTINVRYST

NRAVRINGAGYLVLVLGQDKVTKTRLVALAGQSASVISTSCYWEIPADISEEQEAAYLYATATALLAASLIQSNGTTILV

HGADAILRHAIAIEAASRVVQPIFTTTSPSAASSTGFGKSILVHQNESRRQLAHLLPRYFTAAVNFDSNDHRLFDRMMAI

GHQSGVTQEHLLTTLTAVLPRPSASSLPAHPQAVIDALRKAALTAYQLTVQSTAPGHIATSIADIQSCSQELAVADWTPP

CGSVPVHLQPASQLVRLSAQKTYLLVGMTGALGQSITQWLIARGARNIVLTSRNPSVDPAWILEMQSTTGARVLVTSMDV

TSRASILAVAHALKAGWPPLGGVVNGAMVLWDQLFVDAPLSVLTGQLAPKVQGSLLLDEIFGQEPGLDFFILFGSAIATI

GNLGQSAYTAASNFMVALAARRRARGLVASVLQPAQVAGTMGYLRDKDDSFWARMFDMIGRHLVSEPDLHELFAHAILSG

RGPPSDVGFGPGEGECVIGGLSVQDPAVYPNILWFRTPKVWPFIHYHHEGTGPSSAATGSVPLVEQLKCATSLAQVGEVV

EAGVAAKLHHRLHLPGEVGSGNVTGDTRLTELGVDSLIAVDLRRWFAQELEVDIPVLQMLSGCSVKELAAAATALLQPKF

YPGVVGDSDVGSEKDGSSDSRGDTSSSSYQVITPEESD

>BGC0000129.1|EAL89230.2|MIBiG

MKENEPVAIIGTGCRFPGGASSPAKLWELLRNPREIARKIPANRFNIDAFYHPDGDHHGTTNVQESYFLDEDVRAFDAAF

FNISPTEAAAMDPQQRLLLETVYESLDAAGLRMDALQGSMTGVFCGALRNDYSQIQTMDPQALPAYMVTGNSPSIMANRI

SYYFDWRGPSMTVDTGCSSSLLAVHLGVEALQNDDCSLAVAVGSNLILSPNAYIADSKTRMLSPTGRSRMWDSQADGYAR

GEGVASVVLKRLRDAIVDGDPIECVIRASGANSDGRTMGITMPNAQAQQALILQTYARAGLSPQERPTDRCQYFEAHGTG

TQAGDPQEAAAIHASFFGPKSVADPLDRLFVGSIKTVVGHTEATAGLAGLIKASLSLQHGMIVPNLLMQQLNPKIEAFAA

HLCVPTECVPWPAVPEGCPRRASVNSFGFGGANVHVVLESYTARQITPSHNLPSSLPFVFSAASERTLTSVLESYATFLR

EHAAVSLPSLAVSMWTRRSAHRHRLTLIARSVEELQDHIDKELSRRATGTPSSIVSRPSSRPRRVLGVFTGQGVQWPQMG

LDLIEASPQIQQWMADLQEALDQLPLEYRPEFSLLRELSRPASSSRVHEGLLSLPLRTALQIVQVNILRAVGIEFSMVVG

HSSGEIVAAYAAGVLTAADAIRIAYLRGRAIHESRDSAGRMMAVNLTWQQAQAVCAAEPFSGRISVAAANSPSSVTLSGD

AEGLRDLEWLLKSLGFTPRMLQVDTAYHSPYMKPCADPYRRAMQACPVAVAPSAARWYSSVYPGEVMTGQDQAQRTGEYW

VENMLRPVQFAQALEAAVRETGAPDVIVEVGPHPTLRGPVLQTLSKVHSAHSTIPYLALAERGKPGLDCWAMALGLAWAH

LGPSVVRLDGYVSLLDPGQSHVPLTSLPPYPFDRSQTYWAQSRISLNYNHCITPPNALLGVLSPETGPGEFRWRNYLRPE

ELPWLADRRVGSRSVFPETGYISMALEAARMMVETQGLRLLSVKDFTVHTPLPIQNDAIGTEILVTVNDIFSHDGVISAL

FRCEAAVSDEFVKCATAKMIMHPGDPDRALLPTQGQRALAVGPVDINGFYNSLRCVDYHCTGPFAGLTGLHRGCDLATGT

VHVPSRDPDGPIILHPATLELAIQAMIAALGAPDEGLLTGALLSKTVDNIWVNPALCVSEKEMTVVGYLTDVDGDHIRGD

VDIFTRNGQKAVQMEGVCLVYQPSGIAPTDRQVLSQTEWGPLEPSLKTASRSLPANVLELYSLRDELALLYIKQASDGLT

DSGRNELDCDGTRLLAQMNQCIANAREGRQLAGSPERLDKSIEAFAARVGPSIDDSGLRAIAAVGQQRPHVLRESGHQRV

VTWPHFDEGNEYLKQDVQVSHLVDNLISVVSQTCFRFPQMDILQIGTLGGSVHSVLREMGRSFRSFTYAAPSQPTDGPGL

ERPGEVQHRTFDVDRNPLEQGYQDHAYDMVLFTTARFPLEVAVAHIRRLLKPRGFLVLMVRTNPNITHLNLLFGHPARCT

ETCSGEQVITKEHWIELLSTNGFRVESLDAPPEMAGHQGFSLLLCRASGEYKEPSPRGDLLLVGGGDKNADCVISELAEL

VQGRFDQVLRSPSLDLMEITVRSELTALILVDDQDLTEASLSALRRLITTSKRALWVTCAKSDQPNGGLTRGLLRSIMAS

EQLSCQLQLLHITDPVGVSVEILATALERLVQASAAQECPDSCGLDNIEPEVEYDGSMFLIPRQYHNNSTGLRHLGRRQT

VTGYVDLSQGVVQVLPPTTDATCERFRLLSVAQPPLTSDDGSTIHLRVRYSSLAAVRVAGAMFLRLVIGREVSSNNRMIA

LSSHIASQVIVPESWACSLPDTVSEAQEQAFLHVTAAALLAGYLFDQLPPFGTFVVHGADRVFQSIVHQIPTWRNVKVIF

STSTNNLDKDGAMLYLHEHSTARQLSQVLPSDVSAVAVLDRRGQGIYDRMLSLLPDNATRIQIDDLYRASASTMTPNGRV

SLLVVRAFITACLVAYTSCEAVSLTSMDLVPTATVAEYPPTHCHQGIIDWNPSIPVRAEIPTASSQVQLSPKKTYILVGI

ASELARMACLWLAAHGARWILLTSSKPEPDAWWVEELSSRGTRIAFSTMNLIDGVSVTSLHHSIAHPFPPAVGGVLIQPG

PLPDCSLSQLTAEVLQSRLHPVLKELQLLDELHETQPLDFWVLIGSISGTLGHADQALTAAMSERMATLVRQRRSRGRPA

SLVHLGEISGIDIPADGARPWWGPTAVTQRDIDEVLSEAVLCGGPNSFARSAEFIVGLRHQSLQTGRMAGPVPKLWPFYS

YTATASQGQKPPSLPERRQSAKDLVAAATSREEKAEAIAACLMEKIRARLKLNADAPLSSDTLISELGVDSLVAVELRRW

FAKQLAVDMSIVLILSGASVGELANAAASKLCNGNSELCTSSNLV

>BGC0001358.1|OAQ83760.1|MIBiG

MAEPIAVVGMAMRLPGNVRNGEEFWQLLVEKRNGLCDVPQDRYNVNGFHDPSGKPSTFRMNKGYFLQDVDIAQFDTSFFS

LSKAELERLDPQQRQLLEVAYECMEDAGATSWRGSNTGCYVGVFGDDWQDLNAKETLHKGGYRVTGYDDFVLGNRISYEF

DLHGPSMTVKTGWLIFSPTMTLALSDQGVLSPSGICKTFDATADGYGRGEAVNAIYIKRLSQAIEDGDSIRAIIRGTSVN

CDGRTQAMLTPSPTAQEALIRRAYEQAGIQDMSRTAMVECHGTGTSVGDPLEATAVANCFGDKGIYITSVKPNVGHSEGA

AGLTSLIKAILAIEHRQIPPNIFFESPNPAIPFSKCKLRVPVKTEEWPDARAERVSVNSFGIGGVNAHVIVESLREYQNH

DRGLSNGSTTSSSPAGDMTPTDSDGFEDVGSSESSTVDEFANSDGVHSNGHQDVDGSAESKASDAKLESNDTPQSRSTPS

NGDQTSHTVGRRNGYSGDDVEFPERPHLLLFSATSEPALKDTVKTYQEFLPTSHISLKDVAYTLALRRDHKPHRAFAIAG

NKSSIELSQLETVKTPARIAWVFTGQGAQWPEMGAELIDTNPVFQATIRGLDAFLAGLPSPPPWTIESELRKTAGDSRVQ

KAEFGHPLSIAVQIGLIDVLKSWGIKPDLVLGHSSGEMAAAYASGSITAKAAMAAATFRGTTSTSGTAEKRGSMAAIGLG

AHEMAPYMEPGVVVACENSQCSVTISGDSEQVEKVVQNVKTQREGVLARFLRVEKAFHSHHMLEYGPLYEEHLQPFVSST

SPLIPFYSSVTGKRLSGDGCLGPAYWRRNMESPVLFNTALRSAMTAYEGRLVLIEIGPHPALKGPIGQILRDMGRSADVH

VGTLQRDKGCDESLLQLAGKLFQQDVNVDFSHVLLPSGRHVANLPRYPWKRDNSHWAESRMTREWRFREHAPHELLGSRV

TEISNEPCWRTKLALEDVSWLSGHEVGGQVVFPGAGYISMVGEAIRQLHEELAYSLKNVSIKAGLVLEHGKTVEIVTSLS

PVATDSSDEASWYTFSISSYDGTKWVKHCVGEARASVDKAAQLSVQSPKGYARTVDANEWYNILNRVGFNYTGLFRGLGS

ITAAPGDNRAAASVPSLSQAGKFAMHPAVMDQCFQLFTVSAYGGLGRNCKNIAVPTFIEEIIVRPTAHDLRVGATIHTLE

RGSFVGDLVAEQAGELQLSLKGFKASALTRSDDEDESLPLITRFEWRPHAHFVSLADYLHPRTHIPREWPLFEEMMLLCA

IDHLETIKLTGETQPHLRKFFSWMQGQVDKYRSGRNLFVANDRGLLELTKAQRLGRIAEIAADGEKSQYPAFCIAIHRLF

QTAESIFSGETHPLHVLMKDDVLTEFYAVGDELNYATALRVLGHTNPRLRILEVGAGTGGTTVKVLKALTSSTGERLYST

YTYTDISAGFMASAKERFSEVEGLQYATLDISQDPSEQGYLEGSYDLIIGSNVIHATPNLNVSLSHLRRLLSPGGKLFLQ

ELCPDAKYVNYVMGFLPGWWLGDGDNRPDEPYISADRWAKEMVAAGFAEPEAMVIDGITPYQQSAGIIASPACETSKPLA

VSLLSHSMDGAYVAEAKRVLEDLGVAVDVVTFGQPLPSHDVVSLLDLQASTVHDLTEPSFKTLVAQLQALDLDAKVIWAT

RSAQVACTDPRTAMSLGLTRTARSELSVKLFTVEIDDKTNHLAASKCLVDILMRRHSPQLDAESMDPDWEYAVVDGQILV

PRMHWQTMAAAFERTNGDDSRPTEKHLSVKTPGLLHTMGWSQSERAPLEHGQVTVQTRAIGLNFRDVLIALGVLDNSTRE

IGLEGSGVVTEVGPGVEKLQVGDRVMYMSSGCFTTHITLSQTLCVKLDDGLTFEQGAALPCVYATAAMALVDKANLQPGQ

TILIHSACGGVGLAAIQIAQMLGGEVYCTVGNEDKVRYLMDNHNIPRHRIFNSRDTSFLRDVMAVTDNRGVDVVLNSLSG

ELLHASWRCVAEFGTMIEIGKRDFRRRAKLSMEAFEANRTFVGLDLWQVSQVRPEQVARLLERCIKWMQAGSIKPGVIAR

VWDAEQVQDAFRFMQGGRHIGKIIVKMPQDSSSLESTKERPSPSLRHDRSYLLVGGLGGLGRAIATWMAENGARHLIFLS

RSARQGPQLASFVEELAAQGCEVQLVAGSVSCPDDVKRAVDGASKPIAGVMNLSMVLRDISLSDMTFADWTTAVAPKVQG

TWNLHEAITSELDFFILCSSYSGIVGQWGQANYAAANTFLDAFVQYRHHKGLAASVIDIGVMGEVGFVSKNKDILGLFQK

SGMRILKEQDLLDATNLAIQRSKPSRAQVSDGCFDSPGQILLGLVTSVPIASPNNRVVWKNDIRMSIYHNINGGKDSASS

ATAELDDITTLLKSAASDPSVLQDEESTVIIATAIASALANFLIKEEGSIKVEDSPEHAGLDSLVAMELRNWIRQRFGVD

TTVMTIVQSTSIMSLGDYIRTALVKRS

>BGC0001358.1|OAQ83765.1|MIBiG

MSPTAIRDVSSRSSDNTPSDAQSTGSPVSTTLSTDQFLSDAESQGSFPPIAIVGIGLRLPGGVNTTEAFWSSILNKQSFR

SEVPRSRYNVDAFHSTSGRPGTVKSRHGYFLEDDISSMDAAFFSMSKAEVERLDPQQRLLLEVVWECMESAGQRDWRGRN

IGCYVGVFGEDWLDLNAKDPQHTGMYRITGTADFALANRVSYEFDMKGPSMTIETGCSSSLVGLHEACAAIHAGACESAV

VAGTNLLLSPTMTLALSEQGVLSPSGMCQSFDAKADGYVRGEAVNAVFVKKLEDAVRDGDPIRAIIRGTATNFDGKTAGI

SNPSSDSHEALIRQAYAAAGIDDFTSTAFVECHGTGTPTGDPIETTAVGRVFGEKGVYIGSVKPNVGHSEGASGLTSLIK

TVLALEHSTIPPQANFADPNPKSGDDLDIQFSPITKARPGSSTVNFLFTGQGAQWAGMALELLHDFPDFLASIRAMDKAL

QSLPHPPNWTMEEELQRPKELNRINEPEFSQPICTALQVGLVNLLYTVGIRPAAVVGHSSGEIAAAYAAGALSQDAAITV

AYYRGQVTKKQTRRGGMAAVGLGSEEVAQFLKATEHTAGIVGVACVNSPNSVTLSGDDEALDAAIGRIKSAMPDCFVRRL

KVDKAYHSHHMEEIGDIYEMLIAPYVSTKELRIPLFSSVTSKRIISSEHLGAPYWRSNLERPVLFSSARKFPPHELLGVR

TMDADDLEPAWRNMLRLDDAAWIRDHKIHDDVVFPAAGYVSMIGEAIRQINSDDIPIADFSLKQVDIRTALVLREDETKE

LVTRMRKVRLTTSLESSAWFEFCISSFNGETWVKHCTGQARASSDAVIQGEPELGEDHPRPVSSPAAWYRTMKAVGLNYG

PQFQGLTNISAASATMTKDEDRKAAATIRDRSHDEQDRMAETKYQIHPTTIDYCLQTFMVAVASGLSRELNNLRMPTYID

QLYIRRGATSMRVGVSVGTSQGGLVRGTATAVSDGQVVLWMRGVELSSLGDTNTPTEGGSSPDDGVAAAQLVWKPDLDFA

DPSTLIESHGRVRDACVKVERLSLLCSLETLRRVRDLPVGTSLSHLEKFRTWLVAQRQRAVDGTYDHVPDAPTLASLGPE

HLGAEMLAAQVAVDGTSGSEVGKVMVRSVEFAEAIFRGEIDSIEVLIQQSGLENVYIYMQSLCEYERYFELLGHSNPCLR

ILEIGAGTGGTTEGVLKGLTRPDSNGQPLRRYSQYDYTDVSVGFFGNAQQRFKDYENIQYRVLDITREPEAQGFEEGSYD

LVLASNVLHATPSLQETLSNVRRLLKPGGKLFLQELSPVYRAINYIMGFLPGWWLGDMDGRPTEPYVVPSRWDSELRKTG

FSGVDSAIFDDEAPYHLNANIIATAVLESPSQGTAPNGHVSILCTTRDSSIATQLHNTLADQGHDVTFTSLNDGPPENGC

IISLLDLEEPFFYRMTSEKLSKLQSYLGGIAPSSALLWVTGLGQVGCKDPVYASTLGAARTIRSELAIDFATLELDLSRL

PLRLYVDTIIGVCGKIERTCHLKGSGELDPDWEFASVDGEVLIPRYNWIGIDQDVQYIPGGSEAPADTSGDEEALQLSVG

RPGQLQSLTWVAGEALPPLGPDQIEIEPRAVGLNFKDVLVAMGIVQGLKPGLGLECAGIIRRVGSEVQDLKVGDRVVAFD

HGFFASRTITSSKLAAKIPDALTFEEAATMPCVYSTAMHALVQVGGLQSGQSVLIHSACGGVGIAAINICRMKGAEIFAT

VGNPEKAYFLMKEFGIRQDHIFHSRDGSFYTDLMEVTQGRGVDLVLNSLSGELLHTSWKCVAEFGKMLEIGKRDFVGRGQ

LAMDTFEANRMFVGVDMSQMAIGRPDMFKRVLGDCMRCFSQGLIDPIRPIKSFPASQVVDAFRYMQKGQHIGKIVVTMPG

EGHASSASNLEIVRRAKSAHFRPDASYLLVGGLGGLGRAVSTWMIENGARSLVYLSRSGGESLQDKAFVRELSAQGCTAQ

VFKGDAACQQDVEKAIRDADRPIAGVLLMSMVLQDRAFLKLTLNDWHAAVAPKVNAAWAVHNALESTQTNLDFMVLFSSL

SAVMGQIGQANYASGNTFLAAFAQYRHALSLPASVLDIGVMEDVGYVSENQGILEQFRSLGYYTLKEQGLLDALTFSIKN

QEPRTVDTSQLLNSAEIVIGLRSLQPVSDPTTRVLWKRDRRMALAHLKRASSSAGDTATAASGSQDLAVFVSKVADQPHL

IEQEETQEFLTKQIGARLYAFMFQPEEDLDVNLSLTALGIDSLVAIEIRNWWRQTFGLELSVLEIMSASSIKALGKLAID

GLRKEHRRDTGS

>BGC0001339.1|AMY15057.1|MIBiG

MVPHYQQASSCESNTMTAMDEYQHHEDATIPIAIIGMSCRFPGNATSPEKLWELCAEGRSAWSKIPKSRFRQEGFYNPNA

ERVGTSHVVGGHFLEEDPSLFDASFFNLSAEAAKTMDPQFRLQLESVYEAMESAGITLEHIAGSDTSVYAGACFRDYHDS

LVRDPDLVPRFLLTGNGAAMSSNRISYFYDLHGASMTVDTGCSTTLTALHLACQGLRNRESKTSIVTGANVILNPDMFVT

MSSLGLLGPEGKSHTFDARANGYGRGEGIATVIIKRLDEALAAQDPIRCIIRGTALNQDGKTATLTSPSQTAQSDLIRAC

YRAAALDPNDTAFLAAHGTGTRTGDAVEIAAAAEVFGEKRLPDRPLWIGSLKTNIGHSEATSGLASVIQAALALEKGLIP

PNINFKEPNEKLSQVSSAVKVPSTLEKWPLGSRVRRASVNNFGYGGANAHVILESGLTGSTQLANGNGHYETNGTTNGHK

GANGTTNGHKGANGTTNGHNGTNGITNGHDITRGTIDYEPLESFVISLSAKEEAGTRSMMTNLGEYLRKNHVDDETKHFK

SIAYTLGSHRSTFKWTAAKPITSLEELLAAAGGGQFQASRALERTRLGFVFTGQGAQWFAMGRELINTYPVFRKSLDRAN

GYLKEFGCEWSILDELSRDAETSNVNDMTLSPPLCTAVQISLVRLLESWGIVPTAVTGHSSGEIAAAYAAGALDFRSAMA

VTYFRGEVGLACQDKIVGKGGMIAVGLGPEEAEDRIARVQSGKIVIACINSQSSVTVSGDLAGIVELEEGLKAEGVFARR

VKVQAAYHSHHMQVIANGYLTSLKDILKPGKKFGEIIYSSPTTGKRETSAKLMASAQHWVNNMLSPVRFAESFQNMCFPT

QKVSRSGELEQDVDIILEVGPHGMLQGPIQQMMSLPRFESARMPYLSCLLRGQSAVYTMQSLAAGLMGWGYRVDMAAVNF

PQGTHGARILHDLPSYPWNHDNSHWWEPRLNKAHRQRVHPPHDLLGSLIPGRDLREPTWRHFIRVQDIPWIRDHVVQSQL

VYPGAGFICMAIEAMVQLHDLKDSQSKKIAGYRLADVDILRAMLIPDTSEGLEAHISLRPCSTKLLLTNEWYDFCVSSVG

EDDKFVDHCRGRIAVEFNTSSLSDAPKTTSRERSRGAGLTRSVDPSNLYSFLRAQGIYHGSIFQNLKTISSRKNYSESSF

VVADTASVMPDGFQSAHVVHPTTLDSIFQGAYTALPSAGLDQKTAMIPRSIQEIYLSSALTSEVGQCLVSDTSLIRYDGQ

SFTVNVGISSKADSECTPVLEIKGLRNQSVGQMAPQQGDSGNNDLCFKLEWALDISSVKQERLKEKFGFPLDPAEADIIM

GLRQACLYYIRQALTSLTPSARDQLDWHQKRFYDWMMLQMHLAEEDRLAPNSSAWLQCTSSDEQKLLENVRAASVNGQMV

VHVGESILAILRHEIAPLELMLQDKLLYRYYTDAIKWDRSYQQIDQLVKLHAHKCPSAKIIEIGAGTGGCTRAVLDALST

HGAARCAQYDFTDVSSGFFEAAQQKFTAFADVIRFQKLDIEKDIETQGFECGSYDLVIASQVLHATGKIEDTMANVRRLL

KPGGKLLLVETTRDEMDLQLVFGLLPGWWLSSEEERKMSPSLSTSSWEKVLKKTGFNGLDVELRDCDSDQFYSFSVIMAT

ASPTVPMNPVDFIILHGKSSIPDQWMNDLRTATSPFTKSDPVVGHINNADPTGKFCIFLEDPEEDILFHPDEKSYASIKR

VITQCKGLLWISRGGSMHGTLPTSSLKTGLLRTLRLEYAEKRFISLDLNPARAPWAHESISTIREVLRGALAQTAEIPIR

DSEFAENDGQLYVPRISSDIARNEALSSNSHSPAQTEPFHQPGKLLQMGIKTPGLIDTLQFSKTDAPDHLPADYIEIEPK

AFGLNFRDVMVAMGQLEESIMGFECAGIVRRVGPSSAGHNIKVGDRVCALLGGQWTNTVRVHWHAVAPIPQAMGWETAAS

IPIVFVTAYISLVKIAKLQAKETVLIHAASGGVGQAAIILAKYAGAEIFATVGTEEKRELLIKEYKIPDDHIFSSRNALF

AKSIRQRTNGKGVDVVLNCLAGGLLQESFDCLADFGRFIEIGKRDIELNHCLNMGMFARSATFTAVDLIAIGRDRSYMVA

EALPKVMALLQQKAVRPVTPISIYKIGDIETAFRLMQAGKHMGKIVITAPEDAMVPVVTQPPKLQLRSDASYLIVGGLGG

IGRSLCKNFVENGARSLVLLSRNANVSRQSGEFLDELRSTGCVVSVVDCDISNKTQVESTMLRLKEEKLPIRGIVHAGMV

LQDSVFEHMTLEDYNTATRPKVRGSWNLHSALSDCDLDFFIMLSSLAGVSGSASQANYTAGGAYQDALATYRRSRGLAAV

SIDLGMVQSVGYVAETKGVAERLVRMGYSPISEMEVLKIVEHAITNPPPETSSGQIITGISTKPGRHWTESSWLQDARFA

TLRERARDVKEQSNAQGGGQDKQIGAGQELSMATSLVEAIDVVGRAITAKLATMFLIAAESIIASKSLSEYGVDSLVAVE

LRNWLAAQLSSDVSVFDVTQSQSLTALATTVATKSSRIDKSLLVA

>BGC0001339.1|AMY15068.1|MIBiG

MDVSKEEGQRANGFTNGNINETTNGHTNGYTNGHTNGHTNGTTNATTNGTTNGTMNGTTNGTTNRTTNGTTNITPEFEGK

LPQVPVAICGIGVRLPGGVRSDSDLFQMLVDKRDARGIVPADRYNVKAYYDPRGRPGSILTEYGYYIDEDLAQMDASMFS

MSNVELSMMDPAQRLLLEVTREAFEGAGEGDFRGKNIGTFVGDFTTDWQELQYADLIHTAPYQVIGGSDFVLSNRLAYEY

NLTGPSASIKTACSATAEALHEALLAIRAGSCPSAIVAGANLILTPRGGIGMTAMGVLSPDGSCKTFDSSANGFARGDSV

CAIYIKRLDLALRDGNPIRAVIRACDSNADGGGSGRTFGTPNPITHEALIRKTYADAGLDLHSTSVIECHGTGTPIGDPL

EAEAVANCFADGVRPVYIGSVKPNLGHGEGGSAMASIIKAVVALENRTIIPNVKFNNPNPKIAWDKNLKVPTEPLPWPQD

CQERMSINSFGLGGSNTHIIIDSAASFGIPSPENSLRETANSPNEAQTSILLMSANSPSSITAMSQRYSEYIQAHPDRVE

DMAFTLATRRERLKQASYCIVDHGISSNPPPPMASSGVLQTAFIFTGQGAQWMGMGKELMQQQPAFAHSIREMDTVIKSL

EYAPQWSLEGILLSDNDADKSALAQTDRAQPISTALQVALVDLLATWHVYPAAVVGHSSGEVAAAYAAGILTRREAIITA

FYRGHACARCEIPGGMAAVGLGRTKVEPSLKTGVVIACENSNASVTISGDRSALEEVMADLREKYPTALVRKLQVPMGYH

SHHMATVADLYKELVSPHLDPKAPQVPYFSTVYGRQVQEGKAFGPSYWQLNMESPVLFRTAVSEMLKEMGPNTAHLEVGP

HSALAGPLRQIYEETGNSAPYASTMVRGQNSCTAFLEAIGKLFCFGLSPQIPSTKTRTVLPDIPTYPWDYKDKFWSETRV

MSNWRFKKHRTHELLGERSLESSDIEPCWRNLLRTGTVPWLADHCVGSDIVFPAAGFIAMAGAAASQLAGSDGHYTVREV

NIFSALVLHETTATELITTLRKQPLTSSLQSKWFEFSISSESNGVWTKYCSGLVTASVVISAGLPEMPDTKTFPRKVDTS

RWYTTMSRIGLNYGRRFVGLEEISCSPVHQVASVQITDVQDDYEPYPLHPSTLDKFLQSWTLAFTKGEYRLLTQLYLPTF

IEELSVSPAPRKKISGRTLASGIPGTTVGTSLGMVDDELVFSLRGFKCKRTDESFIQNVSKPRSMTLEWHLDTDFTDLHQ

LIRPTRDTAPENEILERLYLLYALENWDQLKDSTSSHPHLNIYLSWLAEEVKSFTEPGHPLISDSKELVSMDVPHRRREI

AFLRQRSKHYPMAAAVEVYARTCARMVEIMEGKDNLLNVLLEDDLLAKFYNYYNDASDLSSFFQAAGLNKPHMRVLEIGA

GTGGWTSHALRGLTSELGDRLYEEYTITDVSHGFLNQCKERFAAHSNIKYALLDISSDPLEQGFEEGYYDIVIASNVLHA

TPKLVETLSRCRKVLNPAGRLLIQEACAPGSRHGYIMGLFEGWWAGREDGRVRSPLMPVEEWDARLKLAGFEGAGTVVLD

GQVPFYNYANIIAQPAPTTNVQPESRLTLMTSRPELDDFSATTKTMLEEAGYQLDVCSWGAELPSDQDVVFLVDTEASVP

SLADENPENLATFLRYMKDISTSTVLWVTKPAQTACPDPRNGLITGMARTLRAELDMYIATLELDKLDRSAASAVLQVLR

KLQDAARLEETQEKDEKSSDIKVDFEYALSDGELLIPRFHPFVVDQALLKDVPRADSRHLEIGQRGMLNTLHWVGDTLSA

LGSNEVELNMTAVGMNFLDLAVAMNIVDMSQSLGKGYNALGSEGSGIVTRVGSNVTNLKIGDRVATMGVDTSVFATKLQR

PAGSCVRLPSGLSDEDAAGILVPYATVLWSFIEKARLKKGQTVLIHSAAGGVGIAAIHVARWIGAEIYTTVGAQAKVDFL

VNELGVARDHIFHSRDDSFVKDVLSATKGKGIDVVLNSLSGELLHATWQCVAPGGCMLEIGKRDFLGRAQLAMHLFEENR

AYFGIDLSRLALSEPEALQDLLQKTMDLLEKQQLQPLWPTNTFDAVAVEDAFRYMQRGVHMGRIVVRMPEDDSILPIAPM

LPKPQFKADSTYLLTGGMGGLGRSIIRWMVSYGAKDITVVSRSAGNRDVDRALITEIGELGCTLRCFAADISDMDSLQNV

LSSLTKPVAGVLHMAMVLRDVGTLNMDFDSWTAALRPKVQGTWNLHDKLSGSLDFFVLFSSISGTLGSYGQANYAAGNTF

LDSFVRFRHGLGQPASVIDIAAIGDVGYVAETKDVAERIGRAFGSLGTEQEFLDTLQLAIARSTEVPEQQKLSSKTTKYS

EPSQIVMHNKMIPPLSDPRNTTPWKSDARMAIYRNTEEAPQSANSQSKERLGLFLVSLSTDPDQLDEPETPVLFAQEIAK

RVAAFLMKGDKDDDALDTSLTLSQMGADSLVAIEIRNWWKQTFGMEISTLELNSPGQTFDSLGRLATKRLKEAYLLKNSG

S

>BGC0001606.1|CCT75967.1|MIBiG

MPSQIPQWREPIAIVSMACRLPGGIDKPLDLWDHVRAGCSSATAIPKDRFNAENFLSMDPNQKGAQAFRGAHFVKRDIKQ

FDHKFFGISKDTATAMDPQQKQLLEVVYECLESANISMETISKSKIGCYCAMFVSDYHDMLMQDPEYLPTFIAIGTTRTM

LANRVSHALDLGGPSVTIDTACSGALVALHLACQALQAGECDGAVIGASNLFLSPDYALSLTRLGAIAADGQCKTFDASA

NGYGRGEGTNAVYVKRLSDAIRDGDSIRAVIRGTSSNSSGATPAITEPSGRAQADTILQAYAQAGINDFSETGYFECHGT

GTPVGDCIELGAVGSVFSESHKTQDALWVGSTKPNVGHSEAASGLSSLIKVVLALEKGEIPPNTNYKTPNPKIDFDGWRV

RVPTVPQPWPSKSIRRASVNSLGIGGSTAHAVVEFYEPPQLTNGSTNGANAVNGTNGINGTNGINGINGVNGHHEDEEKT

NDPYFLLFTSGASKSSRETNEQNLLEFLKSHEECKSLTSPLVKALNARSQINIRPWKSFAVAQSVDGLVQQLETNALKVG

AGPTIGGSPRVLFTFTGQGAMWSQMGKRLLDAFPVARNSLYNLEEVVRELQSSKTPTWSLIDKLTTELSQEEIDSPAIAH

PLCMAVQIALTDVLSSWGVLPDGVVGHSGGETAAAYACGALTAKEAITVAYYRGIACQNAPSGAMLVIRSAPNAKELQDA

LERNDVQIACFNGPQNLTLAGSAEGVKNVAAELSTHGIVSRAVAVTRAYHTRAMKTVVDEYVGQLKGVIQPKIGRVPMYS

SVTGLELKGTEVDADYWGANLVSPVLYTDAVTLALTSSNLKFDLCIELGPHSLLSRPTSEIVKSLPDSPQLPFFATMLRN

ADSSQQLMNLAGDLVLNGKQLDLDQVNKIAGKVGRLPNHIQDNLPAYAWDYSSTPWTEPRNSQEWRFRKSPRHEILGSRC

RGVNPSAPTWRNKVSIEDAPWLVDHQVNGIVTFSFTTGIAMVIEAMMQVQEENKEIDWANHSFELQDFVFSNSIILPDES

HIDLFLTLIPDNDNAKSEETWYDFTISSLRGDVDIRHCHGRAAVLETSKDNVALRRRTSWHHMPLKVPLKSYYKTLERVG

YGYGPKFQLLTEVRVRPSLSACSAKIDMTSTAQSPVPGQRYLLHPAMMDAALQTPALANRSGFFQEIDTLLLPSKMKRIS

IRMPAKNTDVASCTTNTSPVGFSRIQGSVECYDSLSRPFFVVEGLQMDRATSDDNTTLPWLRLTWKPDIGDISSSDPMLS

PIKIQSLPAEKKLVNLENLVKELIPLIVENGIEKGKDLAPHLLSYHSWFLDQAELHKERLAARHKQQNGFATVQDAIMNV

VANSGISQTVDASIVSQLAINMSRIFQGDVEALAVWLENDLLYRFYEESIFTTSMNQKLLSVAELLAHKNPNMKILEIGA

GTGGATTELLHGFSKAGGKNAYQSFTFTDISAGFFDKAKKKFAQWDRIEFKTLDVEKDIAEQGFTEKYDLVVAANVLHAT

ADLPFAMKNIRSLLRDDGYLLVGELSEDLTSANFLWGPLTGWWLRPRSPGRSGPGLTLDEWRNELAADFDSVSEIEAKHD

KTDTEQLSSTIVMMARAKPMEYTPTKPLSEEKVHIAGVGSDLSMQDHLQKYLGTRGISASSSSLEDLASREWAGEWLILV

DETEGSFLASLQPEQLTALKSWLTKPIKCIWVTRKVYLDPQNTTGGLVTGFARTLRGENSQCQLYTLDLSSDGDITANVI

YHVLERAHYSHDDPISRLDYEIAEKDGQLWTCRLVNDTPLENAYGPARKMDASSTQVVKAPHHLVMGEVGILESLTMAQD

DAYTAIPDGHVLVDVKAAGLDDRDGFIAQGSLPATSFGRECSGVVTRCGANVSSFSPGDRVAVIGQGTFATQYLAPSDCC

SKIPDWLSFEDAAAIPTNFITALYALTTPARVSTGQKILIVNASSTQGIALIKTATALKLDVYAAISDATTKPILTRVGL

HSAKIFVNPTNTGRSSVSRSTTFQAYKLVLNTKSGQYADFAHLVANRGTYIEVTSGESSGDVGHVVPNKNVMFASVDLAD

AYQESKQDLGELLGQVIDMVEKREVDVDSSVSVNGLDSLQSSFAALIEGTSNKQVVSLANVDDQKLIKTRPKTSRFNPHK

TYIITGGLGGLGRAISVWMASYGARHIILATSSITRASESGDLLQQLSSYGCNARVEVCDVGDSEAVERLVASIDTPVGG

VIHSALKLSDCFFEDITLEDFDAVFGPKVNGSLNLHNSLLNQDLDFFVMLSSGCGVLGNEGQSNYAASSTFLDTFARYRQ

SLGLPASSVDLGFVEDVGNISERPEIQASLLSRGLRPITVRDVLRVVEGAIATGSPKNLITDSTYDSFVQSQIVLSFGMI

DKATAEYQSWAQDAKFGLLRSRAADNAALDSDSDSGESAVQTAFKALRNTLGRLGDAPEGKEAALQPFVCTALVAKLAQV

LSIKVGDIQPSRSAIQYGMDSLIAIEVRSWARYAFQIDLPINDLTNPYSIQDLSARVSRMIAG

>BGC0001275.1|AAX35547.1|MIBiG

MSPFLEAEPTEHESSDMSVLSKWTASSSPSVSGKSEIDEIVVVGMACRVAGGNDTPEKFWESIMNKTVSSGEIPAMRWEP

YYRRDSRNAKILDDTTSKGYFLKNLEDFDSTFFGISPKEALLMDPQQRISLEVTWEALEDAGIPPQSLAGTNAAVYMGVN

SDDYSKLLLEDLPGVEAWMGIGTAYCGVPNRISYLLDLRGPSTAVDAACASSLVAIHHGRQSLLTKETDVAIVGGVNALC

GPGLTRVLDKAGAISKDGTCRSFDNDANGYGRGEGASVIILKRMSDALRNHDRILAVVKGSAVGQDGRTNGIMAPNGLAQ

EAVARSALVNVDPKTIQYVEAHATSTSVGDPVEITAMSHIYGANREGNAPCYIGSVKPNVGHLEAGAGAVGFMKAVLALN

KGVIPPQANLQKLNEKIDWATSGIRVPFEATSWPEVSQPRRAAICSYGYGGSVSHAVIEAYQAGARDVEEADIDDDGPKI

LLISAPQEKRLSGYAKALGEWMASSQGTKFSVSSIASTLAVRRGHHDCRAGFLVSSHEETLDLAGRLEKGLTSPDIATGR

VLGKDENNGAVWLFSGHGAQWLEMGVELLAKESLFRETIQDLESVIQDEAGFSAIHALQTSDFETSDKIQVLTYVMQIGL

ATLLRSKGARPSAVIGHSVGEIAASVITGALTPYEGALVVCRRSVLYREVMGKGAMVLVNIPFAEMLTTLEARSDITASI

NSSPSSCVVSGSIEAINRWSAIWEERGIKIFKVKSDVPFHSPLLNCLATSLYTSLVTALAPRTPFIPLYSTATENPREPV

PRDASYWVRNMVRPVLLTSAVEAAAKDGYRVYLEVSTHPVITHSVNETLMDMDIEDAVVIPTLLRNKPTRKALLKSLMMM

WLKGVTINWKLHFGGVAWAHEVPKTEWKHQSFWKPIGTGSTDTSTMVHEVTSHTLLGQRTSVAGEKITVFTSKLDNDTKP

FPGNHPLHGTEIIPAAVLFNTFLHATGTKSLTNVNLRVPVAISAPRDIQVLVQGTEVRLMSRLVQEGEEKEGDRLASWVT

HTTASTVAEPASDIPAYGADINIPSIRARIGTELKTSFSIDYLSGVGVPAMGFPWAVTRHVGNTKEMLVLVDVSPETAND

ATLPWSATSWAPIFDAATSIGSTLFYETPRLRMPAQVGSTSVRDGAVPPKKAYIYVQEASSTSTLAVDVTITNEAGMALA

KFTSMRFSEIEGTPGARKDNIDGLVHQLAWPVARLSEVPSLLKHVVLVGDEDHAFMEPYLRQLSKRRVKTTCVGSAQSLS

VESNSEGTVILYLPSRVASIDDVPEAANRFCKDLLDIVKYAAANETNTRVFAVTDEALHGTSCSSLAHAPLLGLSRIIAA

EQPSLWGALIDVDCETFPFHVVKNMSGADVVKVEDSIPRVGRLRAMPQNKSYPSNQSAKLQPRPDGTYIISGGLGALGLE

VASFLVERGARRIVLLSRRALPPRKDWNVAIEPEKGIIGNIQALEMVGATVYCLALDLSAANASVILSEKLDLLSLPPVV

GVVHAAGVVGDELVLSTTPTAFNRVLAPKVAGAITLHKAFPPTSVDFFMLFSSCGQLFGFPGQASYASGNAFLDGLAEHR

RARGDNAIALQWTSWRGLGMAATTQASADFIEAELEGKGITSISRDEAFLAWDHVAKFDIAHAVILRTRTLDATEVLPAD

ILTEIAVRRAPSTPSPVESVPAPAPALPPPGPDRTVYLTAQIAGCVATVLQLPDVSDVDPRVALPELGMDSVMTVALRKQ

LQMTLGVRVPPTLVWGHPTVGHLVRWFAGKV

>BGC0001035|EPE34340.1|MIBiG

MGDISGSVDPSNMAYEPLAIVGMGMRLPGGIHTAEDFWNLLVEKRSSRCKVPSDRFNIEAFYSPSGRVGTVKMEHGHFLG

PTDDLQHFDASFFSMSKKEVEILDPQQRMLLEVVYECMQNAGQSNWRGGNIGCYVGVWGEDWVDIHAKDSQDAGMYRISG

GQDFAISNRVSYEYDLKGPSFTIKSGCSSSMIALHEAARAIQAGDCDGAIIAGTNLIISPTMSIAMTEQGVLSPDGACKS

FDESADGYARGEAINAIYIKRLSDAIRDGDNIRSVIRATASNCDGKTPGITLPSSESHEAMMRRAYKEACLDPTQTAFVE

AHGTGTKIGDPLEATAIARVFSSEKGVYIGSVKPNVGHSEGASGVTSIMKAVLALENRTIPPNINFSTPNPQIPFEASNM

KVAVEPIPWPKVQAERASVNSFGIGGANAHVILDSPASMGISSTKRNTTNGLSVNGHSINGNSVNGHSVNGHSTNGHSIN

GNSVNGHSVNGNSVNGHSTNGHSINGHSANGNSINGHSVNGHSKPRPALLVLSATNTESLRANVVKHQQYIETNPEKLVN

IEYNLCNRREHLSNRAFCVTDGLSTLQFSPLTKPKKTPTLVMVFTGQGAQWAEMGKELMADFPSFSQDIDLMNNTLSKLD

HPPSWNIKEELLKHESVSCLSKAEFAQPLVTAIQVALVNLLRQFGVKPAAVVGHSSGEIAAAYAANAITANEAIIIAYYR

GQVTKGFSRRGGMAAVGLGREDVMSFLNPGVSIACENSSSSVTLSGDEDALNATCEIIKTALPDVFLRPLKVEMAYHSHH

MKELGEAYEALLRPHLKSTTPVVPFFSSVSGKVVSKSGTLDAAYWRSNLENPVLFNSAIKLILDTLAQDHLFLEIGPHSA

LAGPIRQILKANSRKNDTYVTALERGKDCGESILKMIGELYLQHISINFKQVAPNGTVLTDLPLYQWCHDQEYWKESRVS

KQWRLRKYPNHEILGSRTVEGNELQPEWRNVLHLDNVPWLRDHQIINDVVFPCAGYLSMACEAIRQISASEDFTFRNIVI

QTALVMSDSKSVEMITSLRPVRLTNTLNSVWWDFSISSYNGSLWIKHCGGQIRSGTDNPKFKLPRRIEDHPRSVPSPYPA

MKKVGLNYGPTFQGLERLSALPKKMTASATLSKSELSESHYAIHPATIDHCLQLFIVSTCEGTLRNINKLCVPTSIDQLY

ICGGKSTSGIKAEACGAQTSTGTISGDVVAISGDAIILSLIGGQFSPIEEDSSDEDLDTVAGARLDWKPDLDFVQIDNLI

RPRQQSTAATKTLEKFTLLSMIDIGRRISGLVTKSEYLEKFRSWINAQVERASEDRYNLVEDAQALTVLNAGERQMLIAE

LSKEISNSEVATSGELISRVVKNCEDIMVGKIDGIEVLLPENGLTHFYDSLEHRTDCIDFFTAAGHSKPNLRVLEIGSGT

GGTSAVVLKGLTSTAQRMYSTYTYTDISSGFFVEAQERFKDYHGLEYKVLDISKDPTEQGFQEGSYDLIIAANVLHATPS

LNTTLGHARKLLAEDGRLFLQELSPQVQFANLIMGVLPGWWLGEADGRANEPYISPERWAVELRKAGFSGCDATVYDAEQ

PYQFNANIISRPAKVDRIARRITLLYEPNLNIQQIKFSLENKGYSVDLCTIQEEPPAGQDIVSLLELETPMFEKISGTDL

ALFQRLVKNLGTNHLLWVTRSAQIESYDPRFGMVLGLARTLRSELSLSIATLEIDTVDEVAYNAITNVFDKLKNSSSVSD

MNPDYEFVLSKGVVNIGRYHPVSVEQELAVSASQSQAVKLEIGRFGLLQTLRWVPDLQNKVGHDQVIVEPRCAGLNFKDV

LVSMGIVSGDGLGLEGSGTVVGVGSEVTDFQVGDRVLYIDQNCFSTRTAIPALRCAKIPSTLSWEEAATMPCVYATVIHS

LLNLGRIQKGQSVLIHSACGGIGLAAIQICQNIVGAQIYVTVGNEEKVHYLMDTFGISRDHIFNSRDTSFLPAIKAATNG

RGVDVVLNSLSGELLHASWECVAEYGSMVEIGKRDFIGKAQLNMDLFESNRSFFGVDLAKFDAARCQLLLVQMMEFYEKG

LIKPIAPMKVFEGAKVEDSFRYMQKGSHIGKIVVTIPEQNTDLPLASIVPKLKLNPDAGYLLVGGLGGLGRAVSTWMVER

GARHLIFLSRSAGKSDQDQSFFRELESQDCTVQAFTGSVATFQDVQNAVQRASKPIKGVFQMSMVLNDKPFLEMSCSDWE

TSVLPKVEGTWHLHHALPKDLDFFVATSSLSGSFGNAGQANYAAANTFLDAFVQYRHSLGLPASVVDIGVMGDIGYVSRN

AAIQESLRGAGTYFLQEQDFLDSLNWAVAKSAVKPSLPGQNQLLIGVRSSKSLSDPSNRVSFKRDARMGAYLNTGSSTSA

NTTNATDQLKSFMSSVETDSSILNVPASLDLVTNEIGVRIYTFMLQPIEDLDVSQTLAALGVDSLVTIEIRNWMKRSFGG

LEFSTLEILNAGTIEALGLLTIEGLKRKYEMKDGEAKFSEREDTYLLMKAP

>BGC0000680.1|CBF80428.1|MIBiG

MGSLDDNTLQQVSVLFGPKYPEVELPAGHIRRYLSNQRNANWLHDAIRDLPSVWHDILRLWPAAEKLHGDARLRQLSAFL

GGGTLRPDMAEPMNFLLVPATVLRHLVDFLELKEDKNYDVCDIQGFCVGFLAAIAAACWSDNEDEFGKVVSTVLRLAVYI

GAAVDLDELCEQPARSIAVRWRTAQEHKLLTEVLTRYQGAYISCVTDENAVTVTVWDSQSVSFAKELEKHGLSVKTTTLR

GRFHHSNHTQAVEDILQSCERNSRLCLPSKCHKRSLPRSNINGRVCEADSLFTVAVESILTTQANWKITVTATLDNMGQS

DARSIIPIGAGQFVPRHARCRMLNIVEFNKGEHINGRRKMQSATALDVGVNVTAPETTAVPIAVTGMACRYPQADSVEEL

WRILDLGQCTVSPMPNSRLKSGSLQREPKGPFFGNYLARPDAFDHRFFGISAREAESMDPQQRVLLQVAYEAMESAGYCG

LRRSKLPDDIGCYVGVGCDDYSENVGSRNATAFSATGTLQAFNSGRISHYFGWSGPSVTVDTACSSAAVAIHLACQAIRT

NDCAIAVAGGVNIMTDPRWSQNLAGASFLSPTGASKAFDADANGYCRGEGAGLLVLRPLEAALRDGDPIHAVITGTSVNQ

GANCSPITVPDSNSQRSLYLKALSLSGLTPDVVGYVEAHGTGTQVGDPIEFESIRKTFSGPNRATKLYVGSIKDNIGHTE

TSSGVAGMLKTILMIQKRRIPKQANFRRLNPRITLNERNHIEIPTQSIDWEAEKRVAMVTNYGAAGSNAAIVLREPASTP

ATSNSAHRETLPSHVPFYVSARTEESLRSYCEALQSTIREVAQSGTNTVQHIAYNLARKQNRDMEHFVTFPAAAGEPSEL

MTRLGSIASAHTQVERRSQSFHPVIICFGGQTGDTASISRNLFESCELLRFHVDECENACNALDLPSLFPAIVSPFPNKD

IVNLHCVLFSIQYATAKAWLDSGLQVTRMIGHSFGQLTALCVAGGLSLIDGMRLVATRAQLIQKHWGPHTGVMLSLRASK

EKVQALLDAASGHADLACLNGPDNFVVAGDEESIRRIEIIATEKGMHVELKRLKNTHAFHSRLVDAILPGLSEVANTLTF

RQLDIPVEACAEQEDDWLWVTGDKIVQHSRKPVFFHDAVERTLSRVDGPCVWLEAGTASPVINMVRRVVEASRPLKSHVY

LPTDLSGAQAQANLAKVTCTLWSKAVPVQFWPFHPSETGYRWINLPPYQFAKTSHWIEYNPDAFRSPPQVPDQENVQEAS

LVRLLRQDGKEALFTINNKDNVFRMCTAGHAVANQNLCPASLYFELVVQAALLVSSTATKPTMYHIESLNICSPLVLGMP

GAVLLQLTQQDESHGQWSFVLSTRDGLQDAVTHATGRVSLQAAGSNTGICARLSSLQRLLNLASWNSIATSPSSSGLKRS

TVYQAFARAVNYADYYRGVEEVYAVGHEATGRVILPSSPTKCNPCDPILIDNFIQVAGIHVNCLSETHDDEVFVCSSVGD

VLIGESFVRRDTAATVPWAVYSNYEPESKKKIVCDVFVLDHTTGALAVCMLSATFTGVSIQSLKRTLNRLSNHTARPTEA

EQVSINVAAEATALSSTPVAHVSSSDGDLLAVQTMLGELLGISADELSAAAALGDIGVDSLMSTEVLTEINKRFGVAISN

AELTQIPDVGGLVQRIFPGHSVVRIKTHSQGAVETEITITDREPKSISVDLAPVCDTSPTAFVDKASKLFATTRTSAEFS

RKTRFAGFCDTVFPQQMELVTSYVVEAFHALGADLASLTPGQVVPPVKILPQHGKVMNQLVAVLEYSDLIERRESEIIRS

QQPVGTVPSLILYKKILNKHAQHASEHKLLHTTGSRLAECLSGKADPLSLLFQNAEARALMTDVYSNAPMFKSATIQLAQ

YLKDLLFNLGTQREIKVLEIGAGTGGTTNYLVQELAAVPGLRFQYTFTDISSSLVTLARKRFKAYDFMRYTTLDIENDPS

PELQGQYDIIISTNCIHATRNLITSCTNIRRLLRPEGILCLIELTRNLFWFDLVFGLLEGWWLFNDGRSHALAHERLWDH

NLRQAGFNWVDWTDNDSAESDILRLIVASSTQPFYALEGDDECEADCNTVQEQTVLYNTRDGLELFADIYYPEKTDRSGA

KRPIALLIHGGGHIMLSRKEIHHEQVRMLFDMGFLPVSIDYRLCPEVSLLDGPMQDACDALAWARNKLPQLQLQRRDILP

DGNNVVAVGWSTGGHLAMTLAWTAPARGVSAPEAILSFYSPTDYTDPFWSKPNFPYRVDVSTSDIQTGNPLDALQDAPIS

GYNPPPSKRALGGWMAPSDPRSRIALYMNWTGQTLPVLFYGCNYRARAAESGQDYEVVLPEPILSEVQKVCPFSQISAGS

YRAPTFLIHGTLDDLIPVQQAQRTHDKMQACGVDSDLRIVRDGLHLFDLEANFAGNQHAFQAVVDGYEFLRRHVGL

>BGC0000046.1|ACZ57548.1|MIBiG

MPIFYESSSDASDAAPEFDLRNACQRIVTDDEYVVSSSTEPPLSQQLEPIAVVGMGCRLPGDVSSPSDFWRLMMEKRSGQ

TPKVPSSRFNIDAHFHPDNDRPGSFHVYGGYFINETLQEFDPAFFGITPVEATWMDPQQRKLLEVVYEAFESAGLTLDQL

SGSDTACFMATFTADFQQMSFKEPSFRHSLAATGVDPGLLSNRVSHVFNLRGPSIVVNTACSSSVYALHNACNALRNHEC

SAAVVGGSNLILTVDQHMNTAKLGVLSPTSTCHTFNSYANGYGRAEGVGAIYLKRLSDAVKDGDPIRGVIRSSATNNNGR

APAVGITYPGFDGQRNVMMHAYQRSGLDPMLTGYFECHGTGTAIGDPLEVHAVSDVMNANRTEADGPLQMGAVKTNIGHS

EAASGLSAVIKAILIAERNIIPPTRGLTDPNPKIDWKGWQINVPTESMTIPKHLPITRISVNSFGYGGTNAHTIIESPNS

LLAFPQSYQYSMPGTTTKSKLARGAVKRNRPYLLVFSAHEIGALKRNATAYGRVAANYSLLDLSYTLANHRTRFHSKGMV

VTTPASLHEDIVNGSPNLVLAHKKETATTLGFVFTGQGAQWARMGAQLMAYYPTFLTSIRRMDLALEDLNDAPSWTLEEV

ILQDSATSCVGEAEFSQPLCTAIQVALVQLFRLWGIQPSVTIGHSSGEIGAAFAAGYISEAEAIWIAYYRGQVVKNIDSV

GAMMAVGLGAEAVAPYVESYEPEVVIACHNSPSGVTLSGSVEILKSIEGTLQAEGIFARLVKTNGKAYHSRHMLPAVERY

ESLIGKARQATTQKHVSSKTKMVSSVTNSVLADDAVLDEKYWSANLVSPVLFNQAVQTALKCKEVPEVDILIEIGPHSAL

SGPLRQIKTYLHADKLQYLPTLVRGFPCANQVLKLAGELFLRNYPLDLARVTAIEEVYPSGKIIPRMGNLIVDLPPYQWD

KTKRYWAESRESKEQRSPRFPRHDVLGQLTTGASLAEPTWRNILRIKDLPWLRDHSLGGEAVFPAAGYLSMAMEAVTQIN

EMTEKPCKITSYVFRDIVIQQALVTPDDDNGIEVLLNMHPSRINTDDSGKQWWDFNVSSVSIEGHRKNHMAGSIAIRTSA

RSGLARKVPNLPQRASGRLWNHALKKVGFNYGPTFQDMDNITFDGSTYCAHASTNVKTAVMNDESRHVLHPAIVDSCLQL

MIVAIWAGRASAMQFGAVPVRAEEIVIWKPKATHLTEGARATMFSWIDPRGQRLFNAHSQLVAEDRTVLMEIKNMRCIAY

EAAIPQKLEAPIQPRPYSQLVYKPDVLWVHNTQTHLDVATFVELAEFKTPGLRVLVTDLMVAQSLIAKFPGIPMTLANRD

VKEAEAEADPSGVKKFSLMSLDLTASLATQSYEKLKNSFDVVIAPNILSNSLECIAELLVEGGQAVLGVNGSTIVHDLEK

AGLSGPVFSMKDTMFVTSKVRESDKPVSILVQLIYRHNPTEDITRLRLHLEETGFRSRISKLGDPCPPGSNVIMLADLED

PLVATMSEPEFQHLQTLLSDSANVLWVSCGDYLGAGIDPEAAMTLGLLRTLRSERASLKATFVDFVRTDLASEEFLSRTT

SLAIALFDDEKKLETEYIARDGQLLLSRLIPAEEVNKTHGKIGRETKPQPFDPKAELVGRIQAGKVVFETALLDKPPLQQ

DEIEFRQLATGFNLEDQAAITGASFETDFSHETTGIVTKIGSAITKVSVGDKIVAFSASRFSTYQRVAECLVQVLGPEEP

YTTIAGLPMYYGAALYGLETLARLQYQESVFILPGSGLLGAAAIWIARALHCLPYVVVRDSAEAEHVATTFSLPSAQILT

EYRPEQLVDLDIDVVFSGSSVEPAVAREAWRHTPAFSRFVNCTAAASTSPLDSIPASRGASYLSVNFPRLFQKPRVLGTL

LERIMVLYRQGSIPAPSITVRNITELNESIHSFVDSICDNKIVIAHQTSEGLVDIVESRPRLSLPPDATYLLVGCLGGLG

RSLTSWMMKHGARNFAFLSRSGMDSEQAAILVNNLETRGANVQVFRGDATVKEDVEEAVRSIPADRPLRGVVHAAMVLRD

GLFQNMAYENWTTSIRPKVLGSKHLTEVVADLNLDFFLMMSSVSGILGTPGQANYAAGNSYMDALARLRRSQGKPACAVV

LPMILGVGVVAQNDGLEDSLKRKGMYGIDEEALLDSFEAAIIEQRPQTCQQNEALDHLIVGLDPAGLHKARQEAEGDVDA

FWSADPRFSSLVHSMNVYGGGNQGGDGEAGSILTRLRAAGTESPAKAVDLVRDHFIAKLARILLVDEAEFGGDDNAERSI

ASYGVDSMIGAELRNWIFKDLGLDIAFQQLLSPSLTISKFSELVCGAQGIVVEQKV

>BGC0001280.1|BAQ25466.1|MIBiG

MSSSASFNEPIAIVGSGCRFAGGASSPSKLWDLLCKPKDIRSDITGRRFNAEGFYHPDGSHHGHMNVLQSYLLEEDTRLF

DAEFFGTNPVEAKAMDPQQRLLLEVVYESIESAGLCIERLRGSNTAVFAGLMCGDYEAMMLRDLDQAPTHFATGTSRAVM

SNRVSYFFDWRGPSVTIDTACSSSLVAVHYAIQALRSGDSHTAVACGSNLIFGPEMYVIESKLKMLSPDGLGRMWDKDAN

GYARGEGVTAIILKTLSQALADNDRIEAVIRETGVNSDGTTPGITMPSASAQRDLIQSVYRKAGLDPEAMEDRPQYIEAH

GTGTPAGDPIEAEALSTAFFGNTEKASTPIYTGSIKTVLGHTEGSAGIAALMKVTQAIRNAILPPNLWFQQLNPKLKQFY

GNLQIPTQALPWPTVSDRRPKRASINNFGFGGTNAHAIVESYEPEPRQTVESPDAATVSTPFVFSAASTESLRSNLAAYA

TYLDANPKTSAGDLAYTLRERRSVLPFRIAFPDTTVESLKLSITTRLVEPGNESLGVRTWTAGNRGRSRLLGVFTGQGAQ

YARMGAELVNQAVLAGQLLEKLEGYLSELPEGDRPSWSLRDEMLADGPLSHVGEAAISQPLCTAVQIILVDLLKSAKVKF

DTVVGHSSGEIGAAYAAGYLSARDALLIAYFRGLHCKHATSPNGDIKGAMLAAGTSMEDAIEICEAEEFLGRVTVAASNS

SSSVTFSGDEDAIDEIAAVLQDENKFNRRLKVDTAYHSSHMLPCFDLYVASLRRAGVKALLGNGECTWISSVYEGRSIDP

STDELSGVYWAHNMTKAVLFSQAVRAAVKIATDNDPYTAVLEVGPHAALAGPAKQNIFEALQKELPYHGTLLRGGNAMTA

FSTCLGFLWTHLDTASIDLGSCEAAHSGNKQQFTVLGDLPSYQWKHESAYWAESRKSRQMRLRNQPFHQLLGDVSPDSAP

HILRWKNILKPREMTWLEGHQVQSQVVLPAASYVSTAIEAAQSLASGKKIQLIELSNFHIHNAITFDQNDIGIEVHIEVS

NIYIKENQVHANFTYSAALGDELNDLVLAANGELKVVLVDETPNISLFPQRQAPPPHMIPVQPSRLYGFMKGLEYDFSGA

FQSLIKLERNLGHATCLAQKAKVLVPDADELLVHPIDLDAAFQSVMLAYSYPGDDQLRLLHLPTSIAKLRVNPSVLASQR

YAENDMTLIDSTCSTGDRAEPGDGFSGSVNMYAPGFDHAAIQVDRVKFKPVGSDASNDRDVFYKMHWVPSAADGMLAAAS

VLVGEQDRELMFVLSRIAAYYLRIFDEQLPENDPARSTSPLCHYMNYARHMTNLLKNGQHQWAHQDWLNDTEEDVLDDIV

AKGFMENSDVKIMLLVGNTMPRVFKGETTMLEHFRTSGLLDEYYSNGFGTKQSTLWVASILKQLTDRNPHLNMLEIGAGT

GGATKTILQSIGHDFGSYTFTDISSSFFENAAETFSDWQDSMVFKVCNAEIDPVQQGFQHGSYDVVIAFMVVHACARLDE

AVANLRKLLKPGGLLVLGEGASDGAMQAGAGFIFGTLPGWWRGADEGRTLSPLVNASEWDVILKGSGFSGIDTMSPPTLF

NAFGITLFVSTAIDERIEFARNPLAITKSTVYNKVVIVGGRTPPIVQLSREIQEALIPLAKQVLSYASLEDLDENTLEDE

TVVVSLVDLEAPVFKGITSERWYKFRKLFETKRDILWLTSGRLEDEPYCNMTVGFGRSAMHEEETLRIQYVDVTNVGNFD

AQKIAQYLLRFTSARLDDKDILYTKEPEIIIDDEGRELVPRLFTIKASNDRLNSTTRSIFDPVDINKHVVELQYGKDGPN

FRQLSRYELSEEPTTPQSDHAELRLTSSTVSAIRCPTGYQFLVVGTDQTGAQRLALTSSLTSLLRIPLESTVLCEHPGLS

EANYLGLVAAELSVIAFCDSLFTGQKLAVHNAPASIVRAVLSHVSPKGLSVTFTTDTLGTAVSPDVASQIHIPMFSARSD

IEAILPSDIVCFVDFSASIQAENVAMITSCLPSYCRKENVNTIFSPHGIDTSASTAVLGQLLNRAVNIVKERNVSTTPTL

LGLKALAHGESGTDPLTIIEWTGCTTVPARVTRFESNQLFKSHKTYWLVGLSGALGISLCDWMIERGVRYLVLTSRNPKI

DPRWIRNHERNGVTIKIMLCDVTDEKAINEVHAEIVKTLPPIVGLLNGAMVLRDVSVRNMEFDQVTDVIRPKVLGSIHLD

RIFYNIDLDFFVLLSSINCVIGNVGQANYAAANMGMIGVAGNRRKRGLRSSVVNVGAIIGVGYITQSDRQLDVTVAKTAM

MHLSEQDFHQIFAECMEASHLDSPNGPEISTGLLSITPETIDIPPWYSDPKFARFRVHKAADTGDKSDATNSASTQDLLQ

ACRSQIEVANVIKQAYCTQLRKMLQVSTVDGDLMMMRGVDLGFDSLLSVDVRSWFLKNFRVSIPVLKIMANDVRMSSLVE

LAAESIPAELVPGVPQANANPNGPSSPDSDATESSNQNSDVDVTSTRATSPSTPAATSPDSNVKIKTNSSFAVDWKFETI

PPEPFALPGLSDAPKPRENPEVVVLTGCSGLLGHHLLNTLIAQPSICKIICLAVRRLSSRLESGDLPAPSERICYYEGDL

TSTYFGLDTTTWTSIFHETDAVIHNGSDTSHLKYYSALKQANVESTKQLVSTCLQRMIPLHYISSAGVALFAGLAAFPPI

SCTQTGKTPPADGSHGYMCGKWVCEKMLERTHEKHRLRIVIQRPSTIIRDGKDATVERAGFDWVNSLLHFAHKTQTVPRV

EFNAGAFDLVSVETCCEDVVRELPNRGREGITYVNNVGDVVIPMAQMADVGLSKVEKRYSVLPMEEWTKIVVNAGMHPAV

AALIETFDEPGVEKYPALLRSEDA

>BGC0000170.1|EHA22196.1|MIBiG

MSASRSSTKFSTPAEGSDNGKEFTTPATSTEGHEVPDRPGDALADVAIIGMACRTPGDVRSPDSLWQYLLKKGDASGSLP

DWRWEPYRQRHPRNAALLAQTTAKGYFLDDIDHFDAAFFSISPREAEQMDPQQRLALEVAWEALENAGISPPQLAGSNTS

VYMGVNSDDYAKLLLEDLPNVDAHMGVGTAYCGIPSRISYILDLMGPSVALDAACASSLVAVHHARQAIRAGETDLAIAG

GVNALLGPGLTRVLDEAGAISTDGKCRSFDETASGYGRGEGAGVVILKRLDKALADGDHVLAVLKGSAVASDGKTLGIMA

PNARAQLLVAQKALAEAKVSADSINYVEAHATSTSLGDPTETNALAEVYGAGSGRSPSDPCYIGSIKPNIGHLEAGAGVM

GLIKAVLVLRHGQVPPQANLKTLNSKIAWNENLLCPPRELVTLPCPGPIHPLRAAVASYGYSGTVSHAVLEAFAGHSEFA

ERLSQIPTGDDPSPVLLLISAPQARRVSAAAGALKQWLSENEASISLKTVSSTLAQRRAHHRYRHAIVADSVPDAIAALD

DVSKEAPNRWVIKDKIDSKAAKGPVWIFSGHGAQWADMGRELFESSPAFEEVVRNLEPIIQDEVGFSAIETLQKGCPDRS

DVVQVMTFLMHLGIAAVLEIESGPPSAVVGHSLGEAAAAVVSGALTWREGALVVCRRARLYRELMGQGAMALVRVSAEEA

RTRIGRQTGVWVAIETSPSACVLSGEVDAIKQLSDRWREEGIEVRMVASDVPFHTPMLERLAKPLYESLRGELHPRVPNR

ALFSTSQPDPRSEVLRDAQYWVTNMIQPVRLQSAIAAIAQDGFRALVEVSSHPIVTHSVVETMGECTEDPVLVTPTMVRR

QPALKSILAATGRLHCFGCAIKFIELDPNAPWNSSVPSTVWHHQPFYRAVSQTSASSQLETTHDPAANNLLGKRIALWGT

EEVLYQTRLEEENRPFPGHHPLHGSEIVPAAVLLRTFLQALTPRCVEQVSLQVPVVVSPARKVQIRHNTRNITITSCLEE

SSSQEDGSWLVNTTAAVGAANVVPSQSRMDLSELRKRLPQKLADSFSIDYLASVGVSAMGFPWQVTHHVASDDEMLARVD

ANPDNMGGMNDFLTSLMDAATSISSTLWHRQPLLRMPTSVRRVVAVHEIPIPRVVYIHCTKVASTSECTADVTLTGEDGT

VLMEIQGMSFAGLEGESFSRKSTAGLVHQIQWPPAALVEDPSEFSHIAFVTPDITDPRLEQYQSQLDALAITSSVHQAAS

DLPLTSHTSLAVVYLPQTMTDVFDTATRSCNDLVSIIQTITAAASSTTRVFVLTAGTELGHSALLGLSRIIQAEHPDIWG

SLIEVEDTFSLPLMAMRYVRDADVIRIKDGVPRIARLRPLPSASSSLTPLTFSPASTYLITGGLGALGLSVAHWMVTQGA

RRLLLLSRRALPPRSTWSSTHMNNPTIQSILALERLGATVHCLPIDISLPMAASGLRSTLETLNLPSVAGVIHAAGIVSD

QLVEQVTPDVLESVLAPKIKGALNLHDVFPPASLDFFVLFSSCGQLLGFPGQASYASGNAFLDGLARSRRAQGDNAISLL

WTTWRGMGMGQSANGAMEAELYARGITDITPDEAFRAWSAVASTGGGGTDHAVIVRARVLEGGEPLPHPILTDIATRKAE

VVNAGEHPAGSQEVKLSGRELEQHLRDVINGCVSKTLSVKEDEIDDAVALAEMGMDSVMTVNFRMTLQQTLKVPVGPTLI

WKCPTVQHLVKHFTKELDA

>BGC0001729.1|BAV69313.1|MIBiG

MGSLGDLPLNRISVLFGSKYSEIDRSALHIRRYLSTHRAATWLEGAVEDLPSVWQDVTKVWPAGEGIHGEARLQQLSAFL

RGEGLPSNMEDPMNYLLMPITVLRHLVDFHEFKEAGVNCDIKSMQGFCAGYLAAVAACWEKDQSEFSKVVATMVRTAIFI

GAAVDLDELATQRATSIAVRWKTAEAYKPFAATLGRYPGAYMACITDESSVTVTVWEDQAAALVQELERNGLLVKDTRLR

GRFHHADHLSAAQDILKLCQQDSRFQLPDTCPAEELPRSNADGDLPTLKSLLSAAIQSILITQADWNLTVSNTLNSLDSS

DAKCILSIGAGQFLPRQARSQILNITDSSRGDNLVNGDHDSMTITNGASFVADSINGTAPVPTSIPIAVTGLACRYPQAD

CVEELWKILEQGLCTVSRMPESRLKPDRLQRKPDGPFWGNFISRPDAFDHRFFKISAREAESMDPQQRLLLQVAYEAMES

AGYCGLRATNLPEDVGCYVGVGTEDYSENVGSRNATAFSATGTLQAFNSGRVSHHFGWTGPSVTVDTACSSAAVAIHLAC

QALQTSDCSVAVAGGVNVMTDPRWSQNLAAASFLSPTGASKAFDANANGYCRGEGAGLVILRPLEAALRDGDPIHAVITG

TSVNQGANCSPITVPDSNSQRSLYMKALSLSGLKPEVVSYVEAHGTGTQVGDPIEFESIRKTFAVPSRTERLYVGSIKDN

IGHTETSSGVAGLLKTILMLQKGKIPKQANFTQLNPKITVNQEDKMSIPTSSILWKTQKRVAMVTNYGAAGSNAAIVLKE

PISTPRALCSDEKERLPSVVPFFVAAQTDESLRAYCQTLKASLLNGAHLESIAVQDLAFNLARKQNRSMEFSVSFTNSSS

LTELHDRLDDVISGRMNIEKKTHTSNPVVLCFGGQTGNKASISESLVASSALLRLHLDECESACKALGLPSLFPAIFDSS

PNNDIVNLHCVLFSIQYATAKAWIDSGLKVDRMIGHSFGQLTAVCVAGGLSLIDTMQLISTRAHLIRSEWTSEIGVMLSL

KGEKNAVRELLDSVPESADLACVNGADSFVAAGSEVAIHEIQKNAAERGIKSQRLDNTHAFHSRLVDPILPGLAKVASTL

NYKPLRIPVEACSESEDDWLLPTWEKIVQHSRKPVYFHQAVHRTISRIQGPAIWLEAGTMSPIIGMVRRAVDTPSSVQGH

VFCPMDLSGPQAESNLAKITSSLWSNGVPVQFWPFHSSQRGYQWINLPPYQFAKTSHWIEYDPTAFSYQISKHEEPLTEG

LKLVQLLKNEGKVSLFRINDNDPMFRMCTAGHAVVEQNLCPASLYFELVARAATTTLPKGTDPTMYHLADLNISAPLVLD

MPGSVLLELTQRDSTPGQWAFVLFTREDTLQSVTHATGTISLSPGANNTGISSRFSSLKRLLNPAHWDSIATSPSSSGLK

RSTVYQAFRRAVTYAEYYRGVESVYALGHEATGRVNLPSSPTKNSPCDPILIDNFIQVAGIHVNCLSETHDDEVFVCSSV

GDVIIGESFVKRDPSVATPWVVYSNYEQESRKKALCDVFVVDEATGSLALCVLAATFTSVSIQSLRRTLTRLTNKGVSPV

PVDIAVAAEVAPAVPAASLITATRASSNGDDLRTVQAMLSELLGIPASEIPASASLADVGVDSLMNTEVLSEIKNRFQVV

ITKSELTAIEDVGALVQRIFPGRSTVHIETHAQPAVGITAINGGSKPSSRGSVPASRVGDDLSGFADKAGELFTASRKSN

EHSKATQFLGFCDTVFPQQMELVTAYVVEAFKALGVDLQSLNAGQPIPSVDILPQHSQVMNQLYAVLEYSGLIERSGTSF

CRGHCEVNQNATPVLHQRILNDHPHHTSEHKLLHTTGPRLADCLTGAADPLSLLFQDAQARALMQDVYSNAPMFKSATMH

LAQYLKNLLSQVNSPRPIKILEIGAGTGGTTDYLLKQLSSVAGLCFEYTFTDISPSLVTLARKRFKTFNSIHYQTLDIEK

GPTSEMLGQYDIIVSSNCIHATRSLSTSCSNIQKLLRPQGILCLIELTRNLFWFDLVFGLLEGWWLFNDGRSHALAHESF

WDRTLRSSGFNWVDWTDNQSEESNILRLIVASPTRPALSLEATMESSDIHEETVVYGRKDDLDLLADIYYPQILDSDGKS

RPVALLIHGGGHIMLSRKDVRHTQVQLLIDMGFLPVSIDYRLCPEVSLLEGPMADACEALAWAQSTLPQLNLQRPDIRPD

GNNVVAVGWSSGGHLAMTLAWTAPARGLRAPSAVLSFYCATDYTDPFWTKPNFPYQGDVSIEDVPTQSPFLGLNDRAITS

YNPAPSKRALGGWMSPSDPRSMIALHMNWTGQTLSVLFNGHKYKSLVAIAGGDDNVILPKPTLSEIQKACPLSHVCAGRY

KSPTFIIHGTLDDLIPVEQSQRTHDQMLANGVESELRVVADAPHLFDMSPNLKNNKDAFRAVADGYEFLRSHVRL

>BGC0000076.1|ACD39758.1|MIBiG

MPSTSNPSHVPVAIIGLACRFPGEATSPSKFWDLLKNGRDAYSPNTDRYNADAFYHPKASNRQNVLATKGGHFLKQDPYV

FDAAFFNITAAEAISFDPKQRIAMEVVYEALENAGKTLPKVAGTQTACYIGSSMSDYRDAVVRDFGNSPKYHILGTCEEM

ISNRVSHFLDIHGPSATIHTACSSSLVATHLACQSLQSGESEMAIAGGVGMIITPDGNMHLNNLGFLNPEGHSRSFDENA

GGYGRGEGCGILILKRLDRALEDGDSIRAVIRASGVNSDGWTQGVTMPSSQAQSALIKYVYESHGLDYGATQYVEAHGTG

TKAGDPAEIGALHRTIGQGASKSRRLWIGSVKPNIGHLEAAAGVAGIIKGVLSMEHGMIPPNIYFSKPNPAIPLDEWNMA

VPTKLTPWPASQTGRRMSVSGFGMGGTNGHVVLEAYKPQGKLTNGHTNGITNGIHKTRHSGKRLFVLSAQDQAGFKRLGN

ALVEHLDALGPAAATPEFLANLSHTLAVGRSGLAWRSSIIAESAPDLREKLATDPGEGAARSSGSEPRIGFVFTGQGAQW

ARMGVELLERPVFKASVIKSAETLKELGCEWDPIVELSKPQAESRLGVPEISQPICTVLQVALVDELKHWGVSPSKVVGH

SSGEIGAAYSIGALSHRDAVAAAYFRGKSSNGAKKLGGGMMAVGCSREDADKLLSETKLKGGVATVACVNSPSSVTISGD

ATALEELRVILEEKSVFARRLKVDVAYHSAHMNAVFAEYSAAIAHIEPAQAVEGGPIMVSSVTGSEVDSELLGPYYWTRN

LISPVLFADAVKELVTPADGDGQNTVDLLIEIGPHSALGGPVEQILSHNGIKNVAYRSALTRGENAVDCSLKLAGELFLL

GVPFELQKANGDSGSRMLTNLPPYPWNHSKSFRADSRLHREHLEQKFPTRSLIGAPVPMMAESEYTWRNFIRLADEPWLR

GHTVGTTVLFPGAGIVSIILEAAQQLVDTGKTVRGFRMRDVNLFAAMALPEDLATEVIIHIRPHLISTVGSTAPGGWWEW

TVSSCVGTDQLRDNARGLVAIDYEESRSEQINAEDKALVASQVADYHKILSECPEHYAHDKFYQHMTKASWSYGELFQGV

ENVRPGYGKTIFDIRVIDIGETFSKGQLERPFLINAATLDAVFQSWLGSTYNNGAFEFDKPFVPTSIGELEISVNIPGDG

DYLMPGHCRSERYGFNELSADIAIFDKDLKNVFLSVKDFRTSELDMDSGKGDGDAAHVDPADINSEVKWNYALGLLKSEE

ITELVTKVASNDKLAELLRLTLHNNPAATVIELVSDESKISGASSAKLSKGLILPSQIRYVVVNPEAADADSFFKFFSLG

EDGAPVAAERGPAELLIASSEVTDAAVLERLITLAKPDASILVAVNNKTTAAALSAKAFRVVTSIQDSKSIALYTSKKAP

AADTSKLEAIILKPTTAQPAAQNFASILQKALELQGYSVVSQPWGTDIDVNDAKGKTYISLLELEQPLLDNLSKSDFENL

RAVVLNCERLLWVTAGDNPSFGMVDGFARCIMSEIASTKFQVLHLSAATGLKYGSSLATRILQSDSTDNEYREVDGALQV

ARIFKSYNENESLRHHLEDTTSVVTLADQEDALRLTIGKPGLLDTLKFVPDERMLPPLQDHEVEIQVKATGLNFRDIMAC

MGLIPVRSLGQEASGIVLRTGAKATNFKPGDRVCTMNVGTHATKIRADYRVMTKIPDSMTFEEAASVAVVHTTAYYAFIT

IAKLRKGQSVLIHAAAGGVGQAAIQLAKHLGLITYVTVGTEDKRQLIREQYGIPDEHIFNSRDASFVKGVQRVTNGRGVD

CVLNSLSGELLRASWGCLATFGHFIEIGLRDITNNMRLDMRPFRKSTSFTFINTHTLFEEDPAALGDILNESFKLMFAGA

LTAPSPLNAYPIGQVEEAFRTMQQGKHRGKMVLSFSDDAKAPVLRKAKDSLKLDPDATYLFVGGLGGLGRSLAKEFVASG

ARNIAFLSRSGDTTAQAKAIVDELAGQGIQVKAYRGDIASEASFLQAMEQCSQDLPPVKGVIQMAMVLRDIVFEKMSYDE

WTVPVGPKVQGSWNLHKYFSHERPLDFMVICSSSSGIYGYPSQAQYAAGNTYQDALAHYRRSQGLNAISVNLGIMRDVGV

LAETGTTGNIKLWEEVLGIREPAFHALMKSLINHQQRGSGDYPAQVCTGLGTADIMATHGLARPEYFNDPRFGPLAVTTV

ATDASADGQGSAVSLASRLSKVSTKDEAAEIITDALVNKTADILQMPPSEVDPGRPLYRYGVDSLVALEVRNWITREMKA

NMALLEILAAVPIESFAVKIAEKSKLVTV

>BGC0001340.1|ANF07288.1|MIBiG

MTCKDKKMEDCLENSGRMTHGVADAMGDKTMPIAIVGMSFRGPGDATNVENLWKMISEARESRSPIPQERWNNDAFYHPD

HKHHGTHNVKAGHFFKEDLSRFDAPFFSMTSTEAAALDPSQRLLLECTYEALENGGIPMEKIVGSKTSVFVGSFATDYTD

LLLRDPETVPMYQCTNASQSRAMISNRLSYFFDLHGPCVTVDTACSGSLVALHLGCRSLQTGDAKCSIVAGVNVILNHEF

MITMSMMKFLSPDGRCYTFDDRGNGYARGEGVGCVILKPLVDALKDGDIIRAVIRGTGSNQDGKTSGITLPNPVAQEALI

RDVYEAAGLDPKETTYVEAHGTGTAAGDPIETRALSNVFCRDRPSDDPLLIGSIKSNVGHLEGASGIAAVIKTVLMLENE

IILPNRNFESPNPRIPFRDWKLKVPLSVQPWPARSPHRASINSFGYGGSNAHAIIEGAWSYLHDRGVTGPVRGSKFLRSL

SETIWHRLDGIISYPPTEGEPAIKPNTHHANENFSSLVTSSGNCQVQLTSNETQRTKLFILSAFDEAAGNRQVESMIRYL

SDRSTLEDGRFLDDLAYTLNERRTKFIWKAAIQAKSIVELKEALGKSPRYVKSFKPPALGFIFTGQGAQWCGMGRELLAE

YPVFQSTIKRISAYLESLGAPFNVEAEINMDPSISQINSALYSQPMCTAIQIGLVDLLASWGIMPASVTGHSSGEIAAAY

TIGALSLEDAMAIAYYRGVAAQSMQEKSTVKGTMMAVGMSSEDAKPVLASLTTGRATVACVNSPNSITVSGDETAIEELQ

AIMDERKIFYRRLAVDVAYHSHHMALVADTYADAISEIQTREVGDIEFFSSVTGKQATAADLGPSYWVSNLLGQVKFANS

VRLLCLQTGAERKTRRQGQSKKVNMLVEIGPHSALAGPIKQILQAEPKLKDSEITYISALIRKQNSVTTVLDLAGKLAAH

NYPVNLSTLNRPVGAEITSIVVDLPSYPWNHSNTYWAEPRISKTFRTRQFPRTDLLGALDRSSNPWEPRWHNHIRLSEKP

WVLDHKIQSNTVYPAAGFIAMAIEAVYQQRKDNSSKPISGFKLKDVNIGSALLIPEEEETIETLVTLKRHFDHARSPDTY

WNEFHVYSVTAANTWTEHCRGLVTVQREEEVHPDDTRTMSTEQGYLRDVYTNIERNCVREFDMEEFYTHLASIGLEYGET

FSRVLRAKSGHDLATGTITIPDTAAVMPLGYEHPFIVHPATLDGAFHLVFSAMSGKEGFLEDPAIPVFADEIFVSCEIPA

EPGRELNVCTLLNERNQRSLRSSIWITDPYNPEGGPVVTFTDLHWKVLEKEFKEKHLGIDERVAYSLQWKADVDMMAPAN

LSRMHTGLVPRDVPSRLKVLDQGAVDLFERASELISDSEISQMTNYHKSLWKHMQHLADLIVPSSEHTVKDNTLCDDACL

ADKEQLIEKDHSNCSEGILLRRIGDSLPDILRQRLDPSTLLERETLHRYIRESWSANGIHDAAADYLSLLCHKHPDLSVL

EIDGGIGSVASKAVPAMMGPEGSDMPNFVQYMLTSMQASRLEVLKAENAPLINLISIKELNMAEDPIRQGFKEASFDVII

APAQIAANASSVAVLRNVKKLLKNKGRLIILGTACIQPSLLLMLGVFSEWWEAQGGYSYTASDEELRKRLGQAGFSDTKP

LTIDSDGAPGHKTSIIVARPALPTLVKLTDIVVITGEDDERLRIDVTHLLKLFAGMGVRANVATLEDVEPAGETCIAICD

LEGNVMSNPSESVFEKIKKIFLQSEGTLWVTRGGGSLSSQNPRANIIAGFARTARAENDGSKIVTLDLDAESQLSPITTA

EVICSLFEHVFASASSPENVDAEYVERRGRIMIPRIVENSPLTNKILSIRNPIPELQPFQQPGRPLVLDASRQSLTDMQF

VEDKRLSGELDKGSIEIEVRAAGLTAKDVMIASAQAMPAPLGTECSGIVTKVGKEVHWFAPGDRVAGFGNGTVCNLYRGR

ATSFQKLPENMPFEIATSLPVAFVTAYHAVHNIAHMTSKDRVLVHSADTTIGHAVLEICLSVSSRVFASVSTWEGKRLLV

SKYGLPERYILHKEDPNLKTTAMQVTSSEGFDIIFSTTHGYSSQLAWDCIAHYGRFVSLDSRDADLSGRLDMSSFKKSAS

FSGFNILHFLNHKPRVVDEIWASVMHLFRRKILVAFHEITTFSIGSIEDALRAVKESDIRKVVLVASPESTVKALPPDIT

SNFLREDASYLLVGGLGGIGRALAIWMINHGARNIIFANRSGMSRPESRMTVELLREKGATVAVYSCDVSKSEQLAELVE

ESSQIMPPIRGVVQGAMVLRDVLLEKMTLADYKAVTCPKIQGTWNLHNHLPKDLDFFIMLSSVSGIIGNASQAAYAAGNA

FMDAFAAYRNSLGLPAVTLDLGVITGVGYLASSENRDLLEAMERQGFESMDEEKLMALIQSTLVESRRDGAQFQTITGLG

KWKKGNSLPAFDSPIFSRFRRLSLDAETSSSEGDRANAFRDSLALARTIEEASELVCAALVTKLAARSGISPENVDPSKT

VSEYGVDSLVAVELRNWITHEMDSTVPILELLANNPMNSLSVKIASRSKLVHLDAEKE

>BGC0000120.1|AIG62146.1|MIBiG

MHSVSPSTYPSGGTSPAPADTPGTEYSEYEFSNDVAVVGMACRVAGGNHNPELLWQSLLSQKSAVGEIPEMRWEPYYRRD

PRNAKELKKTTSRGYFLDRLEDFDCQFFGISPKEAEQMDPQQRVSLEVASEALEDAGIPAKSLSGSDTAVFWGVNSDDYS

KLVLEDLPNVEAWMGIGTAYCGVPNRISYHLNLMGPSTAVDAACASSLVAVHHGVQAIRLGESQVAIVGGVNALCGPGLT

RVLDKAGAISSDGSCKSFDDDAHGYARGEGAGALVLKSLHRALLDHDNVLAVIKGSAVAQDGKTNGIMAPNAKAQQLAAR

TALNVAGVDPSTVRYVEAHATSTPLGDPTEISAIAGVYGTNRPADDPCYIGSIKPNIGHLEAGAGVMGFIKAILTIQKGV

LPPQANLTNLNSRIDWKTAGVKVVQEATPWPSSDPIRRAGVCSYGYGGTVSHAVIEEFNPILRPDPLDDGAATGPGLLLL

SGPQEKRLALQAKTLREWMTADGKDNNLSEILTTLATRRDHHDYRAALVVDDHLDATQVLQALANGTDHSFTTQSRVLGA

DVSKDVVWVFSGHGAQWPDMGKQLIHNPVFFAAIQPLDELIQAEIGLSPIELLRTGDFESSDRVQILTYLMQIGLSAILQ

SNGITPQAVIGHSVGEIAASVVAGALTSAEGALIVTRRALLYRQVMGKGGMILVNLPSAETEEILGRRQDLVVAIDSSPS

SCVVAGDKDIVAETAEAFKARGVKTFTVKSDIAFHSPTLNVLMDPLRDALGQALAPTVHIKLYSTALVDPRGQDVRDLEY

WTGNMVNRVRLTSAIQAAVEDGYRLFLEVSTHPVVSHSINETLMDAGLEDFAVIPTLLRKKPTEKHILHSIAQLHCRGAE

VNWAAQMPGRWATGLPTTTWMHKPIWRKIETAPLHTGLTHDVEKHTLLGQRIPVPGTDTFVYTSRLDNETKPFPGSHPLH

GTEIVPAAGLINTFLKGTGGQMLQNVVLRVPVAINAPRSVQVVVQQDQVKVVSRLISSDPSLSDDDASWVTHTTAYWDRK

VLGSADRIDLAAVKARLTTKLADNFSIDYLDKVGVSAMGFPWAVTEHYRDTKQMLARVDVNPAVLGDDPLPWDSSSWAPV

LDAATSVGSTVFQTAALRMPAQIERVEIFTSEDPPKISYLFVEEASDSVPTSHVSVLSETGEVLAKFTAMRFSEIEGTPG

VSGSMESLVHQIAWPPATPAEEPLLITKVILVSPDATARAQYAATLPTQVQSFQFSTTEDFFSNASSLPLEKGTVVAYIP

GEVASLAEVPAASESFTWNLLELIKFIVNGSLPIKVFTLTSSVGDGQTPTALAQSPLIGLARIIASEHPDLGSLIDIEEP

KIPLSTMRYIQGADVIRISDGIARVSRFRSLPRTKLRPASEGPRLLPRPDGTYLITGGLGILGLEVADFLVEKGARRLLL

ISRRALPPRRTWDQVSEDLQPTIAKIRLLESRGASVHVLPLDITKPDAVEQLSTALDRLSLPAVQGVVHAAGVLDNEMVL

QTTRDAFNRVLAPKIAGALALHEVFPPKSVDFFVMFSSCGNLVGFTGQASYGSGNAFLDTLATHRARLGDSGAVAFQWTA

WRGLGMGSSTDFINAELEAKGITDVTRDEAFAAWQHLAKYDIDHGVVLRSLAIDDGEPVPVPILNDIVVRRVSELSGSAQ

AAAGSSGNDAVPSSGPELKAYLDEKIRGCVAKVLQMTAEDVDSKAALADLGVDSVMTVTLRRQLQQTLKIPVPPTLTWSH

PTVSHLVVWFAEKIGK

>BGC0001450.1|AVK70100.1|MIBiG

MITSTSSTEVLTPANGSDDSKGTTTPATSSGDPEMHDDLLRNDTHDDVAIIGMACRVPGDVNSPSALWQFLLEKGDASGD

MPTWRWDPYRQRHPRNAAVLAETTAKGYFLNDIDQFDAAFFAISPREAEQMDPQQRIALEVAWEALENAGISPSRLAGSN

TSVYMGVNSDDYAKLVLEDLPDVGAHMGVGTAYCGIPSRISYLLDLMGPSVAMDAACASSLVAVHHARQAIRAGETDLAI

AGGVNALLGPGLTRVLDEAGAISADGKCRSFDDSASGYGRGEGAGVVILKRLDKALTDGDQVLAVLKGSAVASDGKTLGI

MAPNAQAQLLVAQKALKEAKVTSDSINYIEAHATSTSLGDPTETNALAEVYGVGSGRHPSDPCYIGSIKPNIGHLEAGAG

VMGLIKAVLVLRYGEVPPQANLQTLNSKIAWKENLLRPARELVTLPRGALSRPLRAAIASYGYSGTVSHAIIEAFAGQSL

FAERLAQIPDSDAAPALLLLSVPQANRISTTAAGLSRWLRDMDGAVSLATVASTLSQRRMHHRFRHAIVADSVANAIATL

DDLAKDVPSRWAISDRIGSEAGKGAVWVFSGHGAQWPDMGRELFHSSPAFGEVVRNLEPIIQAELGFSAIETLHAGCPDR

TDLIQAMTFLMHLGIAAVLEAESGPPTAVVGHSLGEAAAAVVSGALTWHEAALVVCRRARLYREFMGQGAMALVRLSASE

ARARIATHPGASVAIETSPTACVVSGTVDALQRLSEQWLEEGVEVRAVATDVPFHTPLLEKLAGPLRGALKNELHPQVPH

RALYSTSLLDPRSDLLRDAEYWVMNMIQPVLLQSTVAALVDDGFRAFVEVASHPIITHSIVETISEQTTDRFIATPTMVR

KQPALKSILAAVGRLHCFGCAVKPTDLDPTVPWSSSVPGTIWHHQSFYRAVSGMTAAQLASTHKPAANDLLGTRTALWGT

EEVLYQTRLEEDNRPFPGRHPLHGSEIVPAAVLLRTFLRALSPRSVEEVSLQVPVVVSPAREIQIRHNTRNITITSRLEE

STSNEHNSWLVNTTATVGADATPSVSHVDIAEVAKRLPQKLSDSFSIDYLASVGVSAMGFPWRVAHHVASDNEMLARVHA

NPDSLPGMDDLLTSVMDAATSIASTLWHSKPRLRMPTAVRRVVAVGVATPQVVYIHCTKAQSSVDEADVIISSEEGMVLM

EIQGMAFAGVEGESLSRKSTSGLVHQISWPPAALAEEPLEFAHIAFLTADATKAQVGTYQRQIEGRGISTSIHERASDLP

LTTHSSMAVVYLPQFTDRIFDTATRSCNDLVTAAQVILSSSDKPTIRLFAVTSESNLGHSALTGLGRILHTEHPEIWGSL

IDLEDPSVFPLMAMRYVRNADVIKIEDGVPRTARLRPLRPAPPHSTAGPATLTFSPASTYLITGGLGSLGISVAQWMVTQ

GARRILLLSRRSLPPRSIWTASHKPGTQFIIDSILSLERLGATIHPVAIDISHPSAVTNLRSALTTLSLPPVAGVVHAAG

ILRDQLIEQITPDAFEAVLAPKIAGALALHTVFPPSSPDLDFFVLFSSCGQLLGFPGQASYASGNSFLDALARSRRKEGD

NAISLLWTSWRGMGMGASSNGALEAELYARGITDVTPDEAFLAWSSISGTEGADHGVVLRARPLESGEPLPHAILRDIAP

RKEKAVGEGNGENEEQRKKLSGKELAEYVLVVVKKCVSTTLSIPEDEVDETVALPEMGMDSVMTVNFRMSLQQTLTVSVG

PTLVWKYPTVHHLVEYFCQVLDE

>BGC0000098.1|ABA02240.1|MIBiG

MKATAASGTPTPIAVVGMGCRFAGGATDPQALWKLLEQGGSTWSKTPSSRFNVSGVYHPNGQRVGSMHVRGGHFLDQDPA

LFDASFFNMTSEVASCMDPQQRLILEVVYEALEAAGIPLESVAGSNTAVFSGAMYHDYQDSLHRNPETLPRYFITGNAGT

MMSSRVSHFYDLRGPSVTVDTACSTTLTALHLAIQSIRAGEADMAIVAGSNLLLNSDVFVTMSNLGFLSPDGISYSFDPR

ANGYGRGEGVAAIILKALPRALRDGDPIRLVVRETALNQDGRTPAITGPSPEAQACLIRECYQKAGLDPRQTSYVEAHGT

GTPTGDPLELAAISAAFQGQPLQIGSVKANLGHTEAASGLASVMKVALALEKGIVPPSARFLQPSKKLLEERKFQIPLSS

QLWLPIDGICRASINNFGFGGANAHAIVERYDPAARISTSKPNGHIRPHDSHVEADRGKIYVLSAKDEHSCQEMISRLRD

YLNRANPTDERQFLANMAYTLASRRSNLRWKAACRAHSLASLLSVLVSDGTRPRRSAEKARLGWVFTGQGAQWFAMGREL

IEAYPVFKEALIECDGYIKGMGANWSIIDELRRGEAESRVNEAEFSLPLSTAIQVALVRLLWSWGIRPAAITSHSSGEVA

AAYAVGAFSARSAIGISYIRGALIAKTQPAPTTKGGMLAVGLSRSEVGEYITRVQQQGEEYLVVGCINSPSNVTVSGDLS

AVVRLEELLHADQIFARRLKVTQAFHSHHMQPLSGEFREALVEVFNADITDTTNACQDVVYASPKTGKRLDDCNHLRDPM

HWVESMLFPVEFESSFREMCFDRKDQAQEVDKIIEIGPHGVLSGAIKQILQLPELAAFDISYLSCLSRGKSAVDTIQLLA

MDLLQGGYPVDLNAVNFPYGCEAAEVQVLSDLPTYPWNHKTRYWKEPRISRAARQRKIPVHDLIGVQEPLCPPLLHLWQN

VLRISDVPWIRDHVVGSRILFPGAGFISMVIDGLSQICNHDPETCGLSYILRDVDLAQALILPTDGDEGVDLRLTIRAAD

QKSLGMRDWQRFSVYSIAGDKDDWTEHCTGLIRAQVDHPVSSSSIQQKTNPPQWSRKMAPQDLWASLHATGICHGPLFQN

IERIESDGQASWCTLTVADTVATMPHAYESQHIVHPTTLDSAIQAAYTVLPFMGTLMKTAMVPSRIGGMKIPASFASLEP

GDMLCAQAKIKNQGLSAFTTDVAVFNESDMDEEAGIELEGLTFQSLGAVISDSRRDLTENESTYSSWHWAPDITLTNSTW

LERILSTGTQSQEIGVMLELRRCTVHFIQEAIENLTTEDVERLSGHLVKFYCWMQAQLACATNGELGQDSADWLRDSEQE

RQSLRSRVVAATNNGEMICRLGPKLSAILRGELDPLELMMDGQLLSRYYIRAIKWSRSNTQASELVRLCCHKNPRARILE

IGGGTGGCTQLIVNALGPTKPVGRYDFTDVSAGFFEAARKRFSGWQDVMDFRKLDIEGDPEVQGFDCGSYDVVLACQVLH

ATSNMQRTLNNVRKLLKPGGKLILVETTRDQLDLFFTFGLLPGWWLSEEPERQLTPSLSPELWRSVLSATGFSGVDLEVR

DCDSDEFYMISTMMSTATPGTPATTLNGPAEVLLVHAGSPPPMDWLQNLQVALGGKNSSITSLKALQGVSDLKGKMCVFL

GEMDRTLLESVVSDDFTSLTSMLQYSQGTLWVTRGAAMASDDPRKALHLGLLRTLRNENHGRRFVSLDLDPLRDPWTAQS

CDAIVNVLNAVGASHEKEFEYAERDGTIHVPRTFSDSSSSEKEDLVVLEPFQNETRLVRLDVQTPGLLDSLHFKLCSADE

AWSSELPEDWVEIEPRAFGLNFRDIMVAMGQLESNRVMGFECAGVVTRLSKAATTGAGGLAIGDRVCALMKGHWASRVRT

ARTNVICIPGTLSFEQAASIPLAFTTAYTSLYTVARLQRGEKVLIHGGAGGVGQAAIILAQLVGAEVFTTAGTHSKRNFL

IDKFKLAPDHVFSSRDSGFIEGIRACTNGKGVDVVLNSLAGPLLQYSFDCLVNFGRFVEIGKKDLEQNSRLNMATFARNV

SFSSIDILYWEEAKSAEIFRALTEIMRLLEQKTIDLIGPISEYPMSAIEKAFRTMQSGQHVGKLVVATAETDMIPVRRGT

MPVALKLDASYLIVGGLGGIGRRICEWMVDHGARHLLILSRSGRTDPFVTGLQKRGCVVRIHSCDVADESQLHAVLQQCH

EDNMPPIRGIIQAAMVLKDALVSQMTADDFHVALRPKVQGSWNLHKIASEVDFFIMLSSLVGVMGGAGQANYAAAGAFQD

ALAQHRVAQGKPAVTIDLGMVKSIGYVAETDPAVAERLARIGYQPMHEEEVLAVLERAMSPSSSSAPPSSNPTIPASPAV

IVTGINTGPGPHFTNADWMQEARFAGIKYRDPLKDDRGGALSSSQPADEDSVRARLSRASTEEEATALVVQVMGHRLVTM

FGLTESEMSATQTLSSVGVDSLVAIELRNWITAQLNVDISVFELMEGRTIAEVAEVVVKKYGVGSKV

>BGC0001816.1|AQM58285.1|MIBiG

MSLPAQSPIAVVGVAYRAPGIGRKGLYEFLAEGKSAFSPVPKERFEQGAYHHRNSEKAGVFSPEGAHFLPDDIYAFDAPF

FNLNADEVRSMDPQHRMLLECAFEAAESAGITLAKLHGTKTGVFASLERCGYGEELLNDLPTSTKYTVFSTAGCMAANRL

SYFFGLEGPSISLDSACSSSVYALHLACQSLRMAECSAAFVGAATLIINAKPIIALDTMGALSPDGKSYAYDSRRNGFGM

GEGGGCLILKRLEDALEAGDPIQAVIRHTVCNHSGRTRGITMPSQLAQEDLLLRVHTEVGLKTGDTSVVEGHGTGTQVGD

PIETRAIANIIAKDRADPVYIGSVKSNLGHLLSSSGMLAIVKAICMLQHATIFPNSGFKEMSPEIDALKLRVAKTCLPWQ

ARGPRRVCTTNFGFGGSNAAVLLEEFQDKSLITGPGNGNKISHGPSRPPLQTKDGIDANGQSSKSYQRLFLLSAKSEKSL

TRLLSSFALHLTKVPTSVAGDRYLADLSFTLNQRRTHFSHRIALVADSVEDLTQQLSTRSENRTGKAYPGQEVVPLFVFT

GQGAQHSRMAAELDQYKPFAMAILRAERYLQEFGATWSLTKELFQCDGEKSRINEAEISQPACTAIQLGLVLLLRSWGIS

PAIAVGHSSGEIAAGYAAGALSFKTAMAIAFFRGKSTMECRRKNRSGLGGMVALGTDVETATRLVESTARVGRASIAAIN

SPSSVTISGDIAAVNRIAQIADVQGIFNRKLKLDVAYHSHHMEPATASYQAAIEPYCAAERTRSKSNGIITTDRASFFSS

VTGCTESADVIQSASYWTENLVRPVKFSQAMQNILSQLGSDKRTVAAIIELGPHAALKGPINQILQSNIEIQSAYLPTLL

RDYKNTETLLGLAGRMFTMGSSLNLAAVNNTTSKDARVLTDLPSYEWSKETRYIHEYPRSVQEKHPGHHYNPLLGWKIPS

EGNDHIFRQVFTLDEMPWIRDHEVAGDAVFPFVGFVRQAVEAFKAIPKTTSEVSSVSLRELHIKRSLRVDNAGRVDMITK

LRPAETGMGAFSSTIWVFEIMTWTESAGWTVHAHGRIGSEAVDFAAGGGPERTMAEEVLSMAQPTATDAEREYKILQESG

ISFGPRFRNMIALWAAPGIAVHETQPPRTDEDSFQGSHTFLELVTLDSMLHSAGIAIGQDDNNGGIRPVYVPVCSSRLQL

STIPVTAEHRFITVTRRLERDRKSGTSRINFVVFVITQSGRVPYLELDMTLQRITQLNNIVNLSREELPEGFYDTLVPHI

GFADGKMLARHFADNNLVASGRHMRHQLANVSLHFLACALKTTTDVNRAMIPFHHQKFLHWAKELVADAVIPQVTPQLIE

EVSKCSAVGELLRAVGEQIPQILRGQVQPLELMMKDGLLTRSYEDSITLHTCNKALAAYVSGLGEINPELRILEVGAGTG

SATLPILEALSGGEATDNDSVPNFSSYHYTDISTGFFENARRKLSRWPQLTYQKLDIRHHPAEQGFKVGSYDLVIAVNVL

HATPDLKATVQHVRALLKPGTGKLGIVEHTDNNDPTVLPFALLPDWWSVSDEFRQSRGGPLMSREHWNRLLVSAGFSGVE

GAVESGEDSLFWTSMVEEHTDFLSDALDEVTIYGSLTDPSEIKLAESVARAMSGIPHLPIPRVKPLAELDDVQGSYSILL

DNPQRSVLSTISSEDEFNKLKSVLLSSKGLLWVSPENRCPEYARIKGILRTLRLEESARKFLHVDGIPLDSIRGASAVAH

LALALVRDKAPAAFREQEFVWHNDMLHVPRLRKLQEARETFALESGISICKEQPIWQSHGPENVLRLSIETPGDLDSIYF

ERHSLSRTALCDDEILIQVSAVGINFRDVLALLGRIPWSPPGREGTGTVMQVGANVSHLQAGDRVFFMVLEGALGTYVRT

PGQFARKVPQSISTADAAGLVAAYVTALVCLDHVARIRQGESVLIHAASGAVGQACILLAQTRGADVFVTAGSAEKRQFL

HETFEIPESHISSSRTLEFRHRVLKQTGGKGVDVVVNCLSGQLLQETWSMVTDFGRFVEIGKKDILENSHLSMGKFSCNV

TFAAVDLTQYFHQRPEVLHSCLDEIVDMLEAKAIWPIQPVTPIPISDIQSGLRRLQSGHNIGKIVAILGPDERVKAERQS

PLQKEKPLQADATYLITGGTGGIGRSLVPMLLDNGAANIVLLGRSGDSSRDVKRLIQQYSRPQCGIQVRAIACDVVSRVS

LSTALHAVKDLPPVRGVIHGSLYLRDSLFMNATFEDWRKISGPKIDAAWHIHELLPGLDFFVALSSGIGIVGNVGQSIYG

GSSTFLDAFAQYRARQHLHSVSISLPVIDDIGYVKEREGLRARLMEENVSFKLSIAQVLAAVKGAIIGRSSGLNKDSRAF

LFVREDSVASQGWENRWHYLYPARRRNAAKVAGSGQDVDRSTPSGEEGRLEALCNKVSSITMIDREDVTPSRSLSEYGLD

SLVAVELRHWIRREFGADLALTHIVGAESLQALSSRIVAQG

>BGC0001057|ABB90283.1|MIBiG

MSVDNKQVPGPVAIVGLACRFPGDATSPSKFWDLLKSGKDAYSETTDRYNAQAFYHPNSKRQNVLPVTGGHFLKQDPHVF

DAAFFNITAAEAISLDPKQRIALEVAYEAFENAGKPLKQVAGTTTACFVGSSMSDYRDAVVRDFAHNPKYHVLGTCEEMI

ANRISHFFDIHGPSATVHTACSSSLVAIHLACQSLLSGDAEMALAGGVGMILTPDGTMQLNNLGFLNPEGHSRSFDKDAG

GYGRGEGCGILVLKKLDKAIQDGDNIRAVIRASGVNSDGWTQGVTMPSSEAQAALIKHVYETRGLDYGATQYVEAHGTGT

KAGDPVETGAIHRTIGQGASKNRKLWIGHLEAAAGVASVIKGVLAMENSLIPPNIHFASPNPEIPLDEWNMAVPTKLTPW

PAARTKRMSVSGFGMGGTNGHVVLEAFNSTPQSILYGDAQYQPAHNGKRLFTFSSHDQAGLDRVSKSLVDHLDSLGPAGA

RPEYLADLGYSLSVGKSGLSWKTAHLAESLTELREKLSSPQSEHAVREPRSQPKIGFVFTGQGAQWARMGVEMLHRPVFK

ESVQRSTDYLQQLGCDWTPIVELSRAQKESRLTLPEISQPICSVLQIALVDELRSWGVAPVSVVGHSSGEIAAAYCIEAL

SHKDAIAVAYFRGKVSAGLNHLNGGMMAVGCSRAEAETLIDESDLQGGHVTVACVNSPSNVTLSGDVAPLDQLKGILEKR

GIFARRLRVEVAYHSTHMNSVFADYTASIADIEPQSCPSHQPIMVSSVTNNQVDPALLGSYYWGRNLISPVLFSDTIKEM

VSPADGNGQKAVDLLVEIGPHGALGGPIEQILSHFDIENVGYQSMLTRGQNAVETSLELATSLFLQGVAIDIQKVNGDSG

CRLLTNLPPYPWNHSKKFRAESRLQRELIAQSTPTRSIIGAPVPKMNESQRVWRGFIRLDDEPWIRGHTVGTTVLFPGAG

MVSIVLEAAQQMVDPGKVARAFRLRDVSFSAAMALPEDQATEVIIQMKPQLVATSGSTPATWWEFTVSSCAGTDQLRDNC

RGLITIDYEGNTSQQMAHEDSQVVSGRISDYHQILEECPATYAKDRFYKHMMKAAWRYGETFQGVENCHPGDGKTVFDVK

LIDIGETFSKGQLDRPFLIHGATLDAVFQGWLGSTYKNGTFEFDKPFVPTKIGEMEISFNVPSEAGYMMPGLCRSHRSGF

NELSADTIMFDKDLSRVILSVIDFRTSELEMDGAATEETTVEVDPADITSKVLWDYSLSLMEPCDLKQVMGSIVAQNSLT

DFVRMLLHDNPAANIVEFISRSDGLPNTYASKLPPGTILPTQIRYAVVDETEDVGDENAASSMLTIDALVDSVSASGATA

DIVVIPQGFQFQDNYAKILEPLAKVSKPNTTIVVAVDTPDTTVPLKAKGFQLLHSIQGTPSLEVFAGLTGEQEKPTNGIH

KEEVVLLLPSMLSTVTKEFAEEVQLDLEGQGFSVSTESLAESIDDSTFDGKTCVSLLEVERPLLDSLSESDFQLIRKVVL

TSQRILWVTHGESPSLALVDGFSRCIMSEIEGVKFQVLHLSEPTGLQHGPRLAAKVIASKASDNEFRDKDGLLQVARIFK

GLTENENIRHHLHDDVRVTRLSNQEHPLRLTIGKPGLLDTLYFVDDERVLAPLADHEVEIQVKATGLNFRDVMASMALVP

VKGLGQEASGIVLRTGRDATHLKPGDRVSTLDMGTHATVMRADHRVTVKIPDAMSFEEAAAVPVVHTTAYYALVRLAKLQ

RGQSVLIHAAAGGVGQAALQLANHLGLVVYATVGSDDKRKLLTDTYQVSEDHIFNSRDASFAKGIMRVTGGRGVDCVLNS

LSGELLRVSWSCLATFGTFVEIGLRDITNNMLLDMRPFSKSTTFSFINMYTLFEEDPSALGDILEEVFKLLGGGILQTPS

PMTVYPINQVEDAFRIMQQGKHRGKIVLSFPDDAQAPVLHVAKNSMKLDSQATYLFVGGLGGLGRSLAKEFVSCGAKNIA

FISRSGDSTSEAKATIKEITSRGANVKAYAADISNETAFLNAMKECSREFPPIKGVVQMAMVLRDVVFEKMTYEEWKLPL

KPKVQGSWNLHKYFDHERPLDFMVICSSSSGIYGYPSQAQYAAGNTYQDALAHYRRAQGLRAVSVNLGIMRDVGVLAEQG

TSGNIKLWEEVLGIREPAFHALMKSLIKGQTDNNSEFPAQICTGLGTADIMATHGLAKPTYFQDPRFGPLAVTSLSSDAS

GDKQSTAMSISSQLSEASSKAKATEIITNALIGKVADILQMPQSEVDPGQPLYRYGVDSLVALEVRNWITREMKVNVALL

EILAAVPMESFAGKLASTSKLVTVS

>BGC0001205.1|KGO40478.1|MIBiG

MSTETQPEMASTPPEPIAIIGMSCRLSGEASSVDGFWDMLRNGRTGHGRVPSSRYEASAWYHPNQDRKGGINHDSGFFLE

EDPSRFDAPFFSITAKEAAGMDPTQRLLLEVAYETFENSGVPMESLPGSRTGVFTGCMTNDYELLSTGDLYNMPHNAATG

NARAMLANRLSWFFDLRGPSIMLDTACSSSLTALHLASKSLRDGECEMALVSGASLILHPNFTQRLSSMHMLSPDGISHS

FDASANGYGRGEGFAAVLLKPLRTALADNDAIRAIIRATGINQDGRTPGITMPSRQAQAGLIRALYGPGLPSLQETAFFE

AHGTGTKVGDPTELSAIGECLMGAETSTNDCLYVGSVKGNIGHTEGAAGVASLIKVVLCLENDMLVPNAGFSKLNSNIHL

DKWLLRLSDKTIRWPSHLPRRASINSFGFGGSNAHAIVESASTYLERPAALLSGLDKGEPQIVVFSTHDKTGIDRVAAKW

GPFLQAQIDAEQNISFRDIAYTMYARRSQLSFRSFAVAGSLGQLRDALQQGLPHFLRANGTAHANLAFVFTGQGAQWAQM

GVELLQVTSFRESITRSEQILSSLGCPWNLFEEIQVEAATSRMNQPDRSQSICCALQIALVNLLASWGVHPKATVGHSSG

EIGAAYAAGFITQEDAIRIAYFRGLCSLQVACHGRAGAMLAANLSLPDAQTYLQGVPPRSVVVACVNGPKSVTLSGDADR

IDQLEKQLQADGLFARKLRVETAYHSPHMNMVAEGYRHDLQDIQPAKCGESSIAMFSSVTKERVYATDMTADYWVRNLVS

PVEFLSAVTSLANMTEASQYRHRAVAVKWSAFLEIGPHEALKGPFLQVLKSINAGLSTVPYHALVRRHADALQTTLNVAG

LLWCIGIPIDIEAVNSSINTAVPQLMHNLPSYPWNHQGSFWHEPVASARLRKRREPHHDLLGSPMDFQNDTEPRWRNFLR

VSDIPWLADHVVADSILFPAAGMIVMVAEAGRILANTSLRLEGIEFNDLAFLQGLVIPDDDRGVETVLHVAPYHELAEWY

EFTLFSLPEDGPWVRHATGTFTLHYDARGVPLNVEEWGLSVERFRKIQTAECETNRDAVYEWLSQTGGVTMGPAFQSVSR

AAFCTEENRLWIEGEVTDTRTMMPSEYASPCFIHPTSLDTLFQAAVLSCSDALGNQNAKIPVGVDRLYLSTTWDLQQGDY

FSVHTETCLNDGDSRLDSIASDVSWSQPRVVLKGVRLGPVPMSKVPSTSTTAGVDSGTSRFSSIVWAQHLESPTSPALAG

HDRDGQLTDWVRDICYTYGNACALVVTQPSWKSPAMTSIQTVRPQLGSRPCLQGLTIVIVGLDKAADEFATAVTRLMPGA

QVKQIAALQDFSPSTFNESFFDVVLVDQPCIGNAADADVLLTSLSSTTKQDGVLAVRTYDSQLDPMDYIQRSSEWKVSGR

IRDGDFLLAHRQRIPAPLDSTIFVLMPDTEQIPPTFRVALERALSAVGVKLCPVDVEDINGLAGKMVISLLEFRHPWTSK

WTSVAMAQFKMLLEARYILWVSPIPILSKDASAASFGASTGLLRTLRNEQPGVTLPQVQYDPDDPNSETSLAQGILQVIQ

LTLVPVPHRNHDMEYRLQHGRLLVPRVVSEAVVDDKMQTLLHGPRPILARLADDPRALRFHAGSPDGHGGQWVEDRQLVS

DVPDDHVEVQLSLRSVVARGSRNFNAHESRLSVVEAVGVIRKLGFAGSTDLSVGDIVVLLVPGAGTVDGMSNRIQVSSKA

VAKLPAQLTLAQAVTVPLAYILAYTSLFDIARLGPNCRVLLVGPVGPILRALLSCALEIRGMQVYVATEERAVVEELVAQ

YAIAPEYVLSIHGGLDGRIADLTEGKGVTAVLSCLGGSSGRLAARCLGSGGHYVDLTGEMNLAALPKAVVSQGCTFTSVN

LNSMLQNQSEKVYSSFRRAVATIGLHHQIQPTSIFPISKWAEAESLARQTGISVAIDFTDPGQVPVVPALQEPVNLPPQQ

TYLLAGGLGMIGLGFAKTLVDSGARHLVILSRSGVLQPSQRIAVASLADQGCHVEIIRCDISQEADLQQVLSQVRSQNWQ

LKGIIQCATVLKDAAFHTMTFEDWASSTNPKILGTLNLHKVFVDVDLDFFITLSSVSGLIGNIGQANYAAGNVFMDELMI

WRRAHGLPGHSIDIGLVPDASGMSDMAETAEVRRSRYSHLEGTEITLRELQMLLRVIILGDIPVPVQIIAGITDDLPREG

ASSWQYDRKLDHRVRLGHSEPDNMPAQISELLKSSPTIEDASYVVNQALREYLASAMATTADTIDSDLPLSSLGVDSLKV

TEVQNWVSRKMGAQLSSFDFLGMQPLRVLSEKIAAQSAFVTVS

>BGC0000063.1|ACB12550.1|MIBiG

MGVIESPPSTGLGSAEEITQANHSHEDSVLPVAIVGMGMKLPGGIHTPDELWEMLVGKRSTRCEIPSTRFSVDGFHSPTS

NPGSIAMRHGHFLDDKDDLHRLDTSFFSMGMTEVSDIDPQQRMLLEVAYECMQSSGQTKWRGSNIGCYVGVWGEDWLDLH

SKDLYDSGTYRVSGGHDFAISNRISYEYDLRGPSFTIKAGCSSSLIALHEAVRAIRAGDCDGAIVAGTNLIFSPTMSMAM

TEQGVLSPDASCKTFDANADGYARGEAINAIFLKPLDNALRDGDPIRALVRATSSNSDGKTPGMSMPSSESHEALIRRAY

EEVCLDPKDTCFVEAHGTGTSVGDPLEATAIARVFGGSSDNKLYIGSVKPNLGHSEGASGVSSVMKAILALENRTIPPNI

NFSTPNPKIPFSEMNMAVPVDAIPWPQDRPLRVSVNSFGIGGANAHCVIETLEEYLGGSLPNRRQVAPTQNGNGSVDADS

SSAVTSITAMKMDVRRKKRQSAVETARQVSTLRFITDNAKIRPSKALYVLSAANSTSLRQSVMDYQEYLGSHKTDPVDVS

YTLCNRREHLSHRTYGVVTTESTNDTYIVPDFSPVSKTNNNTLPEINMIFTGQGAQWAGMGKELMDEYETFYNTIAYLGL

VLSGLEHPPTWDLIQELSRPGESSNVGRAEFSQPLVCAVQVALVDLLRSWGVTPAAVVGHSSGEMAAAYAAGAISSEEAI

TIAYYRGYVNQQYTRDGGMAAIGMGAQEVTPYLIEDVTVACENSPQSVTLSGDKSALEEVCQKIREQVPDCFIRQLKVDI

AYHSHHMKDLGGLFESLLKDKVYSQAPTIPFFSSVAVQQVTEPRSLDAAYWRENLESPVRFTGAVKLLLEARASTASKQV

FVEIGPHSALSGPLRQIFKAHGRGKEAYVSAMIRGQDCTESVLKLAGELFCHGSSLQLSNITADGDVVVDLPPYPWNHDK

EYWSESRVSKDWRFRKFPNHELLGSRTLESSSLQPEWRNVIRLDRISWLRDHQVLNDVIFPCAGYLAMAVEAVRQVAGSP

DIEGFTLKSVVVQSALVLTESKPVEMITSLRPFKLTNTLDSAWWDFCIVAHNGTSWIKHCEGQVRPGQDAHQKTAIFPQT

EPVREHYTRLVDNLYPELLRIGLRYGPSFRGLENVSCVPNGKSAAATLHETTVSESSYAIHPTTIDHCLQLFFPASCDGA

FYRAEKLCVPTAIGRLYLADGKLCEVESARAEASAATNSGGSISGAATLVSKHNSALLSLEDGKFSPLEMDLAGDGNADL

VGAARLEWKPNLDFADLHSLVRPSHASMNDGPELDLVERLTLLAILEIHERIHGVATPGDHAHQHIPNFRGWIEDQVTAA

TEGKYLGVVVDARETASLEREARLSLMTNLRQQVLQTGAASAAVLIGRVVDHCEEIVKGELEGIELLQAEDGLTNYYNYV

ESRTDSIDFFATAGHTRPTLRVLEIGAGTGGGAQVILEGLTNGKERLFSTYAYTDISAGFFVAAQERFKAYKGLDFRVLD

ITKDPAEQGFEPGSFDLIIAGNVIHATPLLNETLANVRKLLAPEGYFFLQELSPKMRMVNLIMGILPGWWLGAAEGRVKE

PYLAPSQWDTVLKEAGFSGVDAAIYDAPCPYHLNANIISRPAKESASQPRATKGRLTLLHHSEDMNSSSITQLRKVLGAR

GLETNMLALQEHEELKVGEQDVIISLLELKKPFFSSVSAAQLESFQRVVAKLGSTEMIWITRPAQHGLSASDDPDFGLSL

GLARTLRSEQSLAITTIEIDQVNDETFKAVTDLVINVLDHREDGTESTGGATTMDPDREYVVENGVVKVARYHPVSLSQE

LASRATKPEAVTLEIGRMGLLQTLGWVPFPTFDPGYGEVTIEPRCAGLNFRDVLLCMGVVEATGVGIGLEGSGIITKVGT

GVSKLQPGDPVFYLADNCFSTQITISAQRCAKIPSQLAFEDAATMPCVYATVIHSLLDIGGLRSGQSILIHSACGGIGIA

ALNLCRNIEGLDIYTTVGNEEKAQYLVDNFGLQRSRIFNSRDASFLYDVRAATQGRGVDLVLNSLSGELLHASWQCVAPY

GKMLEIGKRDFIGKARLEMDLFEANRSFIGIDLARFDSARCQKLLERTAAMIETGSIQPIKPVKVFDASDAEGAFRYMQK

GVHLGKIVVSIPPHSSTLLPVTPKPLQVKLNAEASYLLVGGLGGLGRAAATWMVESGARYLIFFSRSAGLSARDQAFFQE

LASQGCTAQAVQGDVMNLAHVELAMASAPPGKPIRGVLQMSMVLRDKPFADMSLEDWDTAVKPKVGGTWNLHLAAPKDLD

FFFATGSISGSFGTPGQANYAAGNTYLTALFEHRRALGLPASVLQIGLIEDIGYLAQNPERAEALRAAGGFFLRTRQLLE

ALNWALLSSDPHHPEYQLTIGLRSDKALSDPANRVIWKKDSRAALYHNQEISTDAGSGDDQSINAIRLLVASCDEDPAIL

EDSVTVELVTNEIGKRVCMFMLRPVEEMDPTASLTSLGVDSLVTIEIRNWIKRTFGGVEVSTLEILNSGTIEGLAKLTLD

GLKARFAASEKNDGDAYLEMKAP

>BGC0001141.1|AIA58899.1|MIBiG

MAPHNSLDDTPLSSRTFIQEPIAVVGIACRLPGHSSTPKKLWDFLERGGIAANDTPSTRFNLAAHHDGSKKPKTMRTPGG

MFIEDADPRDFDAGFFGISGADAAAMDPQQRQLMEVVYECLENSGVPFEKLYGAQVACHVGSYAVDYDAIQARDPEDRAP

GAVVGIGRAMLSNRISHFFNFKGPSMTIDTACSGSLVGLDVACRYLHTGEVDGAIIGGANMYFSPEHNLNTGAMSVANSL

SGRCHTFDVKADGYCKAEAINCVYLKRLSDAVRDGDPIRAVIRGSATNSDGNTPGIASPNSAAQAAAIRSAYANAGITNL

NDTSYLEFHGTGTQAGDPLEAGGVASVFSASRKPEAPLYIGSVKSNIGHSEPAAGISGLIKAILSIEKDLIPGNPTFITP

TPKIDFEGLKLQPSRANRRWPAAPFKRASVNSFGYGGSNAHVIVEEPKVLLPDMESTYVSSYQTEADLFADDEEVAGGRL

QLLVLSANDEASLRANATTLKNYLTNPNVKISLGDLSHTLSERRSHHFHRGYLITDKASIDENALVIGKKSTNEPRVGFI

FTGQGAQWPQMGKAIIDTFPEARAVVLELDEFLQSSSLPPSWSLLGELTEPREAEHLRKPEFSQPLVTALQIALFDILQR

WGISPRAVAGHSSGEIAAAYAAGLLSKKAAIRAAYYRGQAAALAEKGTADQNQQAFGMMATGIGAEGITPYLQGLGQSVQ

IACYNSPSSLTLSGTVDALAKVQKQLSEDSIFARMLQVNLAYHSTFMREISQGYTDLLNKDFEHLPFKQDSVRMFSSVTG

EQLAGPTDSEYWKSNMVCPVRFDAALSNMLTASDAPDFLIELGPAGALKGPISQVLKSLEGTKAQYTSAMARGAADMQSI

FAVAGSLYVAGGKVDLAQVNKVDGIKPKVVIDLPNYSWNHSTKYWYESESSKDWRNRLFPPHDLLGSKVLGSPWRSPAFM

RSLNVQDLPWIADHKMGPDTVFPATGYISMAMEAIYQRSEALHMLEGEKKVENPRYRLRDVQFKKALVLPDNQSTRMSLT

LSAYTGVGDWFEFKVSSLAGTTWIEHVRGLIRIDEDVPQVASAEEIKPLSHQVDASLWHKCMLDAGYSFGPKFLKQLQIE

ARPGSRRSRSILGLEVPESKYPQSKYPMHPAAMDGCFQTCAPSLWKGNRHAVNAVLVPAMIDSLTITSSKADRGLSLTSA

AYVGLGRPTDNKNYMSNASVYDPETGNLLLRLSGLRYTRIDTGPSVYDAHTFSALISKPDVSLLSSQSLENLAEREQGLN

DRSFGVATELVRLAAHKKPAQRVLELNFVPGLSQSIWASAIEGQDNIGKTYRQFVYRLTDPKALVEAGQQYTSEKMEISL

LDPEGMTLAEDEFDLVVVRLSPAADNVEHVATQLKKVVKEGGQVLFIRQRSVQNSEVIVNGEAEQFDNGSYADLLKSAGL

TFAGHVAFEEGNEFASLSLCRVQPEPDCTGKDVAIYHFVEPSTSALKVITALKARGWNVTTYRAEEASTSPKRFLVLDEL

DTALLPTLSPAHWDSLKILLSLDKRVLWVTNGSQTVISEPNKAMIHGLGRTVRAEDPLVQLTTLDVSASSTDATVDSVEV

ILERLALPEVFHHVESEFIERNGLLHINRIQPDDQVNAVASDSYEGSEPVEQSLHDSPNMIRLRCERVGTTDSLIYSEVS

PYELPLDDNKVEVEVYAAGLNYKDVVITMGIVPENEHILGLEGAGIVRRLGKNVHKVRKLDIGQRVLVFKKGAFANRVHA

EAERVYPIQDSMTFEETCTLASSYLTGIHSLFNLADTKAGSKVLIHSASGGLGLACIQLCQYVGAEVFATCGNKEKRDFL

VKHAGIPADHIFNSRDTSFGAAIMAATNGYGVDTILNSLTGDLLDESWRCIAAEGTMVELGKRDMLDRKGLSMEPFGRNA

SYRCFDMGHDIVSDAMINNLLKRLFALLEAGHVKPVHVATTFGWDNVSGAMRYMRSANHIGKIVISSGDKPIIVPVRPSR

APLQLRGEAGYLLIGGLKGLCGSVAVNLASLGAKHIVVMARSGYDDEVSQRVITDLAALGCTITLGQGDVSKADDVRRVI

KQSPVPIGGVIQGAMVLRDRVFTDMSIEEYHAAVDCKVAGTWNIHNALIEENMKVEFFTMLSSVSGVVGQKGQANYAAAN

AFLDAFAIYRHNLGLAGNSVDLGAIQDVGYMSHHVDLLENLSSDAWTPINEALMLKIVEFSLKQQLTPISKASAGQLITS

IAVPQRENSSLLRDARFSTLSFSDGEDVGAGSDGKDAGIQALQLLVKNKAAVSAIHDAVIDVTVRQFTTMLSLSEPMEPA

KAPSSYGLDSLAAVEFRNWVRLELKAEVTTLDIISATSLEQLAQKIVARLTAV

>BGC0001273.1|AAS98200.1|MIBiG

MPFLDPSSSSSERDKSTSGTEYSTRDESRGVAVVGMACRAPGGINDPDQLWKSLLDKLDASGEVPSSRWESYRNRNPRFE

SALDGTTNRGYFVENIEEFDCQFFGISPKEAEQMDPQQRISLEVAWQAIENAGISAQKLNGTDAGVFWGINTDDYSRLIL

EDLADVEAWMGLGTGFTGVPNRISYHLNINGPSVSVDAACASSLVAVHNGVQAILEGESTVVIAGGVHAMCGPALTRVMD

KAGVLAPDGRCRSFDDSACGYGRGEGAGAVVLKSLPEAINDGDHILAVIKGSAVAHNGRSNGIMAPDSKSQQRVALKALR

AAGVDPMTVQFVEAHATSTSLGDPTEISAIAEVYGKLRPTNDPCYVGSIKPNIGHLEAGAGVMGFIKAVLALQHGVIPPQ

TNLQTLNSKIDWKNAGLRVVKEKTKWPATEGRRRAAVCSYGYGGAVTHAILEEAPPLPELVEPEIPVVNETGPRVLLVSA

LQEQRLPAAAESLRDWLSQHPQPPSLDSISKTLAARRTHHDIRAAAIVDDTASAVGALDNLAKKSNAVWTVQGRVLKSAP

TRDVVWVFSGHGAQWQSMGKEVLKNEVFLNALKPLDAIVRNEIGSSPIEWLQLGDFASSDRVQILTFLMQIGISAILQSQ

GIVPNAVIGHSVGEIAASVVAGALSPEEGTLLVTRRSVLYRQVIGQGAMALVRRPFEQVSRELEGNDKVTVAIDSSPSSC

VIAGTKDAVKEIQKSYNKQEIENFTVKTDIAFHSPMLDQLANPLSVALRDAIWPKQPSIKLYSTTLSDARSQNPRGVKYW

IDNMISPVRLTSAVQAAKEDGFRVFLEVSSHPLVAHSIEETLVDSDAGEHVVIPTLRRDQPAEKSVLRAIGRLHCSGVDI

DWENQISGPWVHGVPTAPWVHRPFWRKPSAAALALHNIRGHSLLGQKVTVAGSETVLYTTTLSKEAKPFPGSHPVMGTEI

IPAAVLLNSFLQATRGFALQNVNLIAPLETESQRLVQVVIQGDNVKILSQIVQKEGLLDNPWTTHTTAEIVPRSEATEAA

VDDTIKSHRPGKELSKSFTMDYLTGTGVDGMGFPWAVLEHWGDTQEMMVRVEVDPDNERLQWETSSWAPFLDAATSIAAT

IFHDRAKLRMTAGLGAIEILKVQDPPRVGWIRVQRNDGSDCTCNVDILDEEGEHFVRINTMRFAELESSSDQNDGVSKLV

HRVAWPPARLTEEPLDIQEIVLVTSDEDAKTVYTKEVPRHVGLTHISTPDELVRRGADIAYRKELVVVYVPGQVSSSQDI

PNLVERFLAELLAVAKHLIQSGVPSKLFVHTTNAFQGKTPTALAHGPLLGTSRLIFSEHPDIWGAHIDDEYRAVPLSTMK

YIQGSDVIRVLDGVARTAKLRDLPLDRRTQPVSQMLPRPEGTYLITGGLGDLGLATAEFFADHGARRIVLLSRRQLPPRR

LWGSLTGADQAVAARINELEKRGLCVFVLSIDIGSSNAAETLIQTLDQLGLPTVQGVVHAAGVLEDQLILNTTEEAFHRV

LSPKVTGTLALHQAFPPSTLDFFVLFGSCGHLVGFPGQGAYAPANAFLDSIATHRRNLGDNAVSFLWSSWTDMGLGNGND

LLVSEIENKGITEISRQEAFEAWLHLARYDIDHGVVVRALPIEEGQGIVNPLLTDIAVYRRSTNSGPKSGEPAAATRTSS

IPSSGPELKAYLDENIRATVGEVLHLPPSDVDPTVALADMGMDSVMTTVLRRQLQKAFSINVPPTLIWNHPTVGHMVGWF

AEKLAA

>BGC0001143.1|EHA28244.1|MIBiG

MAGEYSTAPMAIIGMACRFSGGATSPEKLWDMIVQRRSGWSEIPTSRFNANGLYHPNGERVGTTHVKGGHFLEDDIACFD

AAFFGMASETASAMDPQYRMELEVVYEALESAGIPMESIKGTNTSVYGGVMFRDYHDTHSRDLDTLPRYFMTGNAATMAS

NRISHFYDLRGPSMTVDTGCSTSLTALHLACQNLRSGESNMSIVTGASLMINPDVFLSMSNIGFLSPDGISYAFDSRANG

YGRGEGVGALLVKRLDDALRDGDSIRAIIRETGVNQNGKTPSITAPQQAAQEALIRQCYERVNLDPAQTTYVEAHGTGTP

AGDPLEVGALAAALGGSRSAEHPLYLGSIKANIGHTEAASGVASIIKVALALEKGQIPPNTQLNTPNSELRLNDRNMEVP

VSTQRWPVGKGPRRASVNNFGFGGSNAHAILESPPVENGANRTNGQLKSNGPVVNGNKTNITKRETPWVFRLSAKDAQTC

QQMAADLSTYIESHPPVDEEAFLGRLAYTLGSRRSVFSWTAAVSARSLAELTRALDDDERLVPSRAAPSLRLGWVFTGQG

AQWYAMGRELIATYPVFRSTILECDRYMTEMGSTWTLMEELHREESTSQVNNIVYSLGLATAIQIALVELLWSWGIHPTA

VTGHSSGEIAAAYASKALDMKSAIGIAYLRGVLAEKFDDKILGKGGMMAVGLGRKPVEHYLSRVTAGYCVVACVNSQYSV

TISGDIPAIDQLEQLLQEDQVFARRLRVNGAFHCEQMRPMADLFDWSLRYLLTPHPDFGSVLFSSPKTGSRIQDGTILAT

SSHWVGNMLQAVEFESSFRHMCFGDPSPKGAKGTQDVDLVLEIGPHGALGGPIQQLMTLPEFEGSGISYLPTLVRKQDAV

FAMQRLAIDLTHRGYPVDLNAVNFPHGTLSLSILHDLPSYPWNHSTRYWLEPRRNRADRQRQAPPSDLVGYSQPSITPLA

RTWRHIIRLSDLPWLGDHRVQSSIVFPGAGLVSMAIEGMRQVAAGRQQTVSAYELRDVDIAKALTVPEADEGVEVQLNIR

PCDEQMLGTKDWLAFQIFSVSGDSRWTEHCSGRISVITTSDSTPLPSAIPSQSEDLYNRRIDPRYMWAAMRSVGIYHGPL

FQNIHQVLAKPSASRTIFAIADTAAVMPKKYQTPHVLHPTTLDSVFQAAYTLLPESGARLPSAMVPRHIRSVRVSAQISN

SPAHELEAYATLNRDYDAQSFETSLTVVDAKDGNSPVLEVDGLTCQSLGRALDREADPHENEICSRWEWAPDIGTLDAAA

CKDRIRCAPEAAEIETMRDLRRATILYILDIVSSLTVADVQQLRGHLKKFYVWMVEQLKKASRNQFAPDSAQWRDISAAD

KAALYEKVGRTSVNGEMLCRLGPLGASFLRQEMAPLEVMLENRLLFRYYLEALKWDRSTRQVSELVRLCTHKNPRAKILE

IGAGTGGGTQVILEALGKENGSSTGARFGRYDFTDISAGFFEAAKERFQDWADLMNFQKLDIEHDPVAQGFEEGSYDVVI

ACQVLHATKSMDRTLTHVRKLLKPGGKLILMETTRDELDVFFAFGLLPGWWLSEEEERRTTPSLTLPFWNQVLSRNGFAG

LDLEVHDCDSEEFYAFSTILSTAQAPALSITSPVTIVTGTSPPPTSWMSELQTAVAAHIGCQPVIATLETVTPQGNICIF

LGEADEPLLDHVSNPVEFDRIIHLATRCKGLLWITRGGSLDVDKPAMSLSQGLLRTLKSEYQGKSFVSLDVDPRRSPWTA

EVVQAISQIFPASFSETTDPATCEFEYAERDGVLHIPRTVKDIPMNRNIFPESDTTEKTIHCRFRDAARPLRMKIGTPGL

IDTLVFHDDLDAKSDPLPADWIEFDPTAFGLNFRDVMVAMGQLEANAIMGFECAGTIVRLGATAAAKGFAVGDRVCTLLR

GHWATRPRAPWTSVMRIPQHLSDQEAASFPTVFATAYIALHETARLQRGESILIHAATGGVGQAAIQLAQLIGAEIYATA

STPAKRQLLHETYGIPENNIFSSRDPSFATDVHLRTDGRGVDVVLNSLAGRLLQESFNCLAEFGRMVEIGKRDLEQHSGL

DMYPFTRNVSFSSVDLLTWQSRRGADISCVLQSLSKLLGEKKIMPVYPLTLYPITQIEKAFRTMQTGQHMGKIIISVGEQ

DTVPVVERPPPFSLRSDASYVIVGGLGGIGRVLCEWMMARGARHLIIISRNARPGPFVTELEQQGCEVRTLACDIAAEDQ

LAAALAQCADMPPIKGVIQGAMVLKDTVLEQMTVGDFEAAVRPKAQGSWNLHQQLGDVDFFIMLSSLMGVMGAASQANYA

AGGAFQDALATYRRNRGLPAVSLDLGIVRSVGFVAQTDGVQERLVQMGVTSLSEETVLRILEQAITHPTGPPQIITGINT

APGKHWDEASWIQDPRFAALRYRDSTQAGSSRATTGTAKQGKIRDQLAEIASPVDAAALICQELMQKLASMFGLVVEEMS

ATQDLSSYGVDSLVAVELRNWLVAQVGAEVSIFDLMQSPSLEDLSLRVATKRT

>BGC0000160.1|EAU32819.1|MIBiG

MEVHGDEVLSVDSGVSTPPSTGSGFRRPLETPGTEIGNLNLNPQNEVAVVGMACRLAGGNNSPEELWQSILNRKDASGEI

PSMRWEPYYRRDIRNPKILDQTTKRGYFLDHVENFDAAFFGVSPKEAEQMDPQQRLSLEVTWEALEDAGIPPQSLSGSET

AVFMGVNSDDYSKLLLEDIPNVEAWMGIGTAYCGVPNRISYHLNLMGPSTAVDAACASSLVAIHHGRQAILQGESEVAIV

GGVNALCGPGLTRVLDKAGATSTEGRCLSFDEDAKGYGRGEGAAVVILKRLSTAIRDGDHIRAIIKGSAVAQDGKTNGIM

APNAKAQELVAWNALRTAGVDPLTVGYVEAHATSTPLGDPTEVSAVSAVYGKGRPEGNPCFIGSVKPNVGHLEAGAGAVG

FIKAVMAVEKAIFPPQTNLKRLNSRIDWGQAGVKVVQETLEWPGNEDDVRRAGVCSYGYGGTVSHAIIEEFAQQLQRPTT

NTTDEDPLPRILLLSAPQERRLALQARTQASWIAAEGRNRTLESIATTLSTRRGHHDYRAAIIAENHDDAVQKLSDIVNG

KAAEWTTSSRVLDASCSKDVVWVFSGHGAQWTAMATDLLKDIVFYQTISRLDPIVEREMGFSALHSLASGDFESSIKVQV

LTYLVQVGLAAILRSKGLEPQAVIGHSVGEIAASVAAGCLTAEEGALIVTRRANLYRRVMGAGAMVLVNIPFVDMEKELQ

GRTDLVAAIDSSPSSCVVSGATEAVLALVEDLKSRGVNAFRVKTDIPFHHPMLDQLSEPLREAMAGSLSPRKPRVRLYST

SAEDPRSMVARDIYYWTSNMVNPVRLTAAVQAAVDDGLRLFLEVSSHPIVSHSVRETMLDLGVEDFTVTNTMARNKPADK

TILSSIAQLHCRGAVVNWKKQLPGPWALDVPLTTWDHKPYWRHIHTGPISASTLHDVDKHTLLGQRVPVAGETTMVFTTQ

MDDQTKPFPGSHPLHGSEIVPAAALVNTFLHATGATTLSNITLRVPVAISQPRDIQVVVSQNQIKICSRLTQKAGSGADE

GSWLTHTTGQWEAGGSKNAPAQLDIAAIKARLANNKLADNFSIDYLDKVGVSAMGFPWAVTEHYGTLQEMIARVDVAPDV

PATSPLPWDAASWAPILDAATSVGSTLFFDQPRLRMPAHIHGVQVYTTQPPLKVGYLYVEKAGDRDLAVHVSVCDELGTV

LARFESMRFSEIEGTPGSNGSEESLVHQLAWPPAIYSEKPLTINNVVLVSRDKNVADLYCGSLKDRVSSITVLDAAADLL

SLSQDSSSVLQAKDTAVVYVPGPLHSADSIPTAAHSFLMELLLLVKIIVNGSLPTKVFVLTDRVCESESATALAQSPIHG

VSRIIAAEHPDQWGGLIDVETPGQFPLETMKYVQEADNIRISDGIPRIARLRPLPRDKLLPPSKQTSLLPRPESTYLITG

GLGALGLEVAQFLVEKGARRLILVSRRALPPRREWADILADASSSLAPALETIQALEAQGATVHTLAVDISSPDAAPQLA

VAIDSLSLPPVRGVVHAAGVLDSQLVLSATSDSVERVLAPKITGALVLGTVFPPKALDFFMLFSSCGQLLGFPGQASYAS

GNAFLDAFATSRRHQGDNAVAVQWTSWRSLGMAASTDFINAELASKGITDITRDEGFRAWMHISKYDIDQAAVLRSLAFE

ADEPLPTPILTDIAVRKAGSASSADAPSAAPKETNEMPESIPERRTWLDERIRDCVARVLQLGSSDEVDSKAALSDLGVD

SVMTVSLRGQLQKTLGVKVPPTLTWSCPTVSHLVGWFLEKMGN

>BGC0001305.1|CCT72377.1|MIBiG

MGSQISEKYYAREPIAIVGTSCRFPGGASSPSKLWELLDNPRDVVQKIPPRRFSTEAFYNADSQHHGVSPSDLIKGLRLT

LQWSTNVKHAYLLDDDPRGFDRDFFAINPKEAEAMDPQQRILLETVYESVESAGYSIEQLRGSSTAVFVGCMSFDYQFAA

IRGIDSTLPQYHATGAAMSILANRVSYFYDWKGPSVAIDTACSSSLVALHQAVSALRSGDAKLAVAAGSNLILGPEPFIS

ESKLNMLSPNGRSYMWDASADGYTRGEGFSVLLLKTLTKALADSDHIECVIRETGVNSDGKTPGITMPSSNAQAQLIRDT

YAKCGLDPNRESDRPQFFEAHGTGTQAGDPIEARAIQEVFFPDHTQPTDSKLLVGSVKTVIGHTEGTAGLAGVLKATLAI

QHSQIPANLHFNELNPKIRPYYDNLRIPTETTPWPTLSPGAPRRVSVNSFGFGGTNAHAIVESWDGPYVRERSPAPNAIG

AGPFVLSANSSQALAANAGALAEYLKEHPTTNLGQLGYTLFKRTNLPFRAAFSGSSVECLVEKLQAGKESLKSNSRITTL

PESLPPRVLGVFTGQGAQWAKMGEGLYQTSMVFRNSVEQMQHSLDSLPESDRPNWTLAEQLHAPKETSRVGQATVSQPLC

TAIQVALVDVLHVVGVELSAVVGHSSGEIGAAYAAGYLSAGDAIRIAYYRGFHSHLAQGPGGKRGSMMAVGLSYDQALSF

CNELSGGITVAASNSYTSCTLAGDAEVIEEAKSRLEENGTFARVLAVDTAYHSHHMKPCATPYLESLKQCDIQVQKPKKG

CAWYSSVWGSDGSSRSFNTEGELLQGQYWIENLTHPVLFSQALARALNEDQCFDCALEVGPHPALKGPSLEIIKTLTGVS

LPYSGVLKRGEGAAEAFADALGLLWKSFPSSRPTITFNGICRAFSAVKPSSMTILKNLPNYSWDHATLFWKESRASRNFR

TPKHAPHELLGRSVSHGEHSRREVHWRQVMRLRELPWLRGHKIQGEILFPASGYLSMAYEAAIRLVDEQQSVRIVEMQDM

DIVRAMRLEDDTSGLEVLFTIHVTSQSQSCINANVACYSGAVDAPQPLDAHQTELTAHFNGRIRLWLEQPSAYTLPLRSE

PLLPMSELNMAQVYSSLSKEGFDYSGDFQAKSMLRRLGHTVVTLSSPSPVSWTHTLTHPAPIDTAVQGLLTAFSFPGDGR

IGTTYLPTRIESVRISTAPSESDAPILKADSVVTLTDVTTITGDVDIFDAASCHTQVQMRGVQMTAIGKPPDRWLYAGKI

WARDIAYGLEPGSGTQFSDDDRLLYEQLVRTAYFYLRQLRSKIRPPELMLMGKFRRHMMRWVTEHLFPQIESGQHPDIRT

EWKDDTLDTVQRWRNSRPADNNDMNLLHAMGQSLIPIVRGTVPPLKVLVQDGMLDRLYFEGIGFRDGNVDLGAIVKQLGH

EHPRMRIVEVGAGTGGTTRTVLDALETRYAAYTYTDISTGFFENARSVFSQHASKLTFKTLNIENDPVDQGFTEGSFDML

IASNCLHATHSLENTLRQCRKLLRPGGRLVLLEITRDFLPIQMIMATLPGWFLGMDEGRLWAPTVTLDRWDEILKTTGFS

GVDISSTPSYCSVIVSQAVDETVSVLRNPLASPDGVPSLSEIVIIGGVGSGLAPQIQSLLTSTIPVETRIASSLEDVEVN

RGSTVLCLGDLDSPSFCNMDQTRFEGLQKVFQAANAVLWVTSGATSGTNPEANITVGMGSTLMAERGDLKLQFLDVDDPT

TIDPSMLARMLLRLAILDQSKSDETLWTIEPELTLKNGALYIPRVQPLDVINRQSTARYRQVIEDIDLNSASPVVDLVKY

QDSWKLQSSPIDSTRVNGIEVRVTASSLHTLSCEGGKPEYIFMGRELLSGDNIIGLSASNSSIANITDDQVLHRWQDSQT

GAINVAQLSDLLAKALAENLLRNTTGPVWIHGAPSNLSEAIGEVTKEEGLDVFQTTSDLAQVAAFNFIHPYATRQDIQDK

QPSGLRNFVDLEHSELRNEALHTSVAASLPVSAISHRCMMDLTHGINRSYLAKLAARCLTEDRKSSEDHSQVILIDRIAS

ESSKDLGPTTVIDWHAANTVTALVRPLEHRDLFFPSKSYLLFGMTGDLGISVCKWMVDNGARNVVLASRNPNVPSSVLNF

MSRKSATVRALSVDITDMESLCTAREDIESTMPPIGGVMNAAMVLRDRLFHDLTWEDFAAVLGPKVQGTQNINNAFGQKE

SALDFFVCFSSATSIIGTVGQSAYAAANHFMVSLVQQRKRRGLAGSIVHISVLTGLGYIFRRGSEHTAVIDKALLPRLER

QAETDLHEMLAEAIVCGRPGSSQPPELITGIKAVFQGEWRDDPRLLGYLGQQQLGNDSAKDQAAGMVSVETQLDAADDPA

ECLPILEKRFAQALGNMLEIDPEKVDGSMPVASLGIDSLVAIRIREWFMKELGVEVPVLKIMSVGHSLSRVCDDVLVDWR

RVKKEGELEDKK

>BGC0000062.1|AAD43562.2|MIBiG

MGVIESPSSTTSGSAEEMAQAITGHEDSVLPVAIVGMGMRLPGGIHTPDELWGMLVEKRSTRCEIPPTRFSVDGFHSPSS

KPGSIAMRHGHFLDDKDDLHRLDTSFFSMGMTEVSDIDPQQRMLLEVAYECMQSSGQTNWRGSNIGCYVGVWGEDWLDLH

SKDLYDSGTYRVSGGHDFAISNRISYEYDLKGPSFTIKAGCSSSLIALHEAVRAIRAGDCDGAIVAGTNLVFSPTMSVAM

TEQGVLSPDASCKTFDANANGYARGEAINAIFLKPLNNALREGDPIRALVRATSSNSDGKTPGMSMPSSESHEALIRRAY

GEVFLDPKDTCFVEAHGTGTSVGDPLEATAIARVFGGSSDNKLYIGSVKPNLGHSEGASGVSSVMKAVLALENRTIPPNI

NFSTPNPKIPFSEMNMAVPVDAIPWPRDRPLRVSVNSFGIGGANAHCIIETLEEYLGRSLPNESQVAPIRNGNGSVQADS

SSAVTSITAMKMEVRRKKRQSAVEAAGLVSNLRLVADSTKIRPSKALYVLSAANPTSLRQSVMDYQKYLASHKTDPVDVS

YTLANRREHLSHRTYGVVTTESTNDTPIVPDFSPLSKTNNNSLPEINMIFTGQGAQWVGMGKELMDEYETFYNTIAYLGL

VLSGLEHPPTWDLIRELSRPAESSNVGRAEFSQPLVCAVQVALVNLLRSWGITPAAVVGHSSGEMAAAYAAGAISSEEAI

TIAYYRGYVNQQYTRDGGMAVIGMGAQEVAPYIVEGVGVACENSPQSVTLSGDKGVLEEVCQKIKEQVPDCFVRQLKVNV

AYHSHHMQDLGGLFENLLEGKVYSQSPTIPFFSSVTVQKITEPRSLDAAYWRQNLESPVRFTGAVKLLLEARASTGSKQV

FVEIGPHSALSGPLRQIFKAHGRGKEAYVSAMIRGENCTESLLKLAGELFCHGTSLQLSNVTADGDVVFDLPPYPWNHDR

EYWSESRVSKDWRFRKFPNHELLGSRTLESSSLQPEWRNLIRLDGIPWLRDHQVLNDVVFPCAGYLAMAVEAVRQVAGTS

EIGSFTLKSVVVQSALVLTESKPVEVLTSLRPVRLTNTLDSAWWEFSIVAHNGTSWIKHCEGQVRPGQDAHQKTAVLPQS

EPISQHYPRLVDNLYPELLRIGLRYGPSFRGLDNVSCVPNGKKAAAMLRETTVSESSYAIHPTTIDHCLQLFFPASCDGA

FYRAEKLCVPTAIGRLYLADGKSCEVESARAEASAATNSGGSISGAATVVSKQNSTLLSLEDGKFSPLEMDLAEDGNADL

VGTARLEWKPNLDFADMCSLVRPSHASMNDGPELDLVEQLTLLAILEIHERIDGAVTPGGHDHAHQHIPNFRGWIADQVT

AAAEGRYRGVVADAREIASLERGARLSLMTNLRQQVLRTGAASAAVLIGRVVDHCEEIVKGELEGIELLQAEDGLTNYYN

YVESRTDSIDFFATAGHTRPTLRVLEIGAGTGGGAQVILEGLTNGKERLFSTYAYTDISAGFFVAAQERFKAYKGLDFKV

LDITKDPSEQGFESGSFDLIIAGNVIHATPTLNETLANVRKLLAPEGYLFLQELSPKMRMVNLIMGILPGWWLGAAEGRV

EEPYLDPSQWDTVLKETGFSGVDSAIYDAPYPYHLNANIISRPAKESAPQPRAIRGRLTLLHHADDTNSSSITQLREVLD

ARGLETDMVVFHEHEELKAGEQDVIISLLELKKPFFSSISAAQLESFQRIVAKLGSIEMIWVTRPAQHGLSASDNPGFGL

SLGLTRTLRSEQSLAITTLEIDQVNDESFKAVTNLAIKVLDHREGGSTESTRGATTMDPDREYVVENGVVKVARYHPVSL

SQELASRASKPEAVTLEIGRMGLLQTLGWVPFPTSDPGYGEVTIEPRCAGLNFRDVLLCMGVVEATGVGIGLEGSGIITK

VGAGVGKFQPGDPVFYLADNCFSTQITISAQRCAKIPSQLAFEDAATMPCVYATVIHSLLDVGGLRPGQSILIHSACGGI

GIAALNLCRNFQGLEIYTTVGNEEKVQYLVDNFGLPRSRIFNSRDASFLYDVRAATQGRGVDLVLNSLSGDLLHASWQCV

APYGKMLEIGKRDFIGKARLEMDLFEANRSFIGIDLARFDSARCQKLLERTAAMIQTGIVQPIKPVKVFDASDAEGAFRY

MQKGVHLGKIVVSIPPHSSTALPITPKPLQVKLNPEASYLLVGGLGGLGRAAATWMVESGARYLIFFSRSAGLSVRDQAF

FQELASQGCTAQAVRGDVLNLADVELAMASAPPGKPIRGVLQMSMVLRDKPFADMSLEDWDTAVKPKVHGTWNLHLAAPK

DLDFFFATGSISGSFGTPGQANYAAGNTYLTALFEHRRALGLPASVLQIGLIEDIGYLAKNPERAEALRAAGGFFLRTRQ

LLQGLNWALLSSDPHHPEYQLTIGLRSDKALSDPANRVIWKKDSRAALYHNQEISTDAGAGDDQGINAIRLLVASCEEDP

GILEDPATVELVTNEIGKRVCMFMLRPVEEMDPTASLTSLGVDSLVTIEIRNWIKRTFGGVEVSTLEILNSGTIQGLARL

TVDGLKARFAASEQTDGDAYLEMKAP

>BGC0001385.1|ANF07288.1|MIBiG

MTCKDKKMEDCLENSGRMTHGVADAMGDKTMPIAIVGMSFRGPGDATNVENLWKMISEARESRSPIPQERWNNDAFYHPD

HKHHGTHNVKAGHFFKEDLSRFDAPFFSMTSTEAAALDPSQRLLLECTYEALENGGIPMEKIVGSKTSVFVGSFATDYTD

LLLRDPETVPMYQCTNASQSRAMISNRLSYFFDLHGPCVTVDTACSGSLVALHLGCRSLQTGDAKCSIVAGVNVILNHEF

MITMSMMKFLSPDGRCYTFDDRGNGYARGEGVGCVILKPLVDALKDGDIIRAVIRGTGSNQDGKTSGITLPNPVAQEALI

RDVYEAAGLDPKETTYVEAHGTGTAAGDPIETRALSNVFCRDRPSDDPLLIGSIKSNVGHLEGASGIAAVIKTVLMLENE

IILPNRNFESPNPRIPFRDWKLKVPLSVQPWPARSPHRASINSFGYGGSNAHAIIEGAWSYLHDRGVTGPVRGSKFLRSL

SETIWHRLDGIISYPPTEGEPAIKPNTHHANENFSSLVTSSGNCQVQLTSNETQRTKLFILSAFDEAAGNRQVESMIRYL

SDRSTLEDGRFLDDLAYTLNERRTKFIWKAAIQAKSIVELKEALGKSPRYVKSFKPPALGFIFTGQGAQWCGMGRELLAE

YPVFQSTIKRISAYLESLGAPFNVEAEINMDPSISQINSALYSQPMCTAIQIGLVDLLASWGIMPASVTGHSSGEIAAAY

TIGALSLEDAMAIAYYRGVAAQSMQEKSTVKGTMMAVGMSSEDAKPVLASLTTGRATVACVNSPNSITVSGDETAIEELQ

AIMDERKIFYRRLAVDVAYHSHHMALVADTYADAISEIQTREVGDIEFFSSVTGKQATAADLGPSYWVSNLLGQVKFANS

VRLLCLQTGAERKTRRQGQSKKVNMLVEIGPHSALAGPIKQILQAEPKLKDSEITYISALIRKQNSVTTVLDLAGKLAAH

NYPVNLSTLNRPVGAEITSIVVDLPSYPWNHSNTYWAEPRISKTFRTRQFPRTDLLGALDRSSNPWEPRWHNHIRLSEKP

WVLDHKIQSNTVYPAAGFIAMAIEAVYQQRKDNSSKPISGFKLKDVNIGSALLIPEEEETIETLVTLKRHFDHARSPDTY

WNEFHVYSVTAANTWTEHCRGLVTVQREEEVHPDDTRTMSTEQGYLRDVYTNIERNCVREFDMEEFYTHLASIGLEYGET

FSRVLRAKSGHDLATGTITIPDTAAVMPLGYEHPFIVHPATLDGAFHLVFSAMSGKEGFLEDPAIPVFADEIFVSCEIPA

EPGRELNVCTLLNERNQRSLRSSIWITDPYNPEGGPVVTFTDLHWKVLEKEFKEKHLGIDERVAYSLQWKADVDMMAPAN

LSRMHTGLVPRDVPSRLKVLDQGAVDLFERASELISDSEISQMTNYHKSLWKHMQHLADLIVPSSEHTVKDNTLCDDACL

ADKEQLIEKDHSNCSEGILLRRIGDSLPDILRQRLDPSTLLERETLHRYIRESWSANGIHDAAADYLSLLCHKHPDLSVL

EIDGGIGSVASKAVPAMMGPEGSDMPNFVQYMLTSMQASRLEVLKAENAPLINLISIKELNMAEDPIRQGFKEASFDVII

APAQIAANASSVAVLRNVKKLLKNKGRLIILGTACIQPSLLLMLGVFSEWWEAQGGYSYTASDEELRKRLGQAGFSDTKP

LTIDSDGAPGHKTSIIVARPALPTLVKLTDIVVITGEDDERLRIDVTHLLKLFAGMGVRANVATLEDVEPAGETCIAICD

LEGNVMSNPSESVFEKIKKIFLQSEGTLWVTRGGGSLSSQNPRANIIAGFARTARAENDGSKIVTLDLDAESQLSPITTA

EVICSLFEHVFASASSPENVDAEYVERRGRIMIPRIVENSPLTNKILSIRNPIPELQPFQQPGRPLVLDASRQSLTDMQF

VEDKRLSGELDKGSIEIEVRAAGLTAKDVMIASAQAMPAPLGTECSGIVTKVGKEVHWFAPGDRVAGFGNGTVCNLYRGR

ATSFQKLPENMPFEIATSLPVAFVTAYHAVHNIAHMTSKDRVLVHSADTTIGHAVLEICLSVSSRVFASVSTWEGKRLLV

SKYGLPERYILHKEDPNLKTTAMQVTSSEGFDIIFSTTHGYSSQLAWDCIAHYGRFVSLDSRDADLSGRLDMSSFKKSAS

FSGFNILHFLNHKPRVVDEIWASVMHLFRRKILVAFHEITTFSIGSIEDALRAVKESDIRKVVLVASPESTVKALPPDIT

SNFLREDASYLLVGGLGGIGRALAIWMINHGARNIIFANRSGMSRPESRMTVELLREKGATVAVYSCDVSKSEQLAELVE

ESSQIMPPIRGVVQGAMVLRDVLLEKMTLADYKAVTCPKIQGTWNLHNHLPKDLDFFIMLSSVSGIIGNASQAAYAAGNA

FMDAFAAYRNSLGLPAVTLDLGVITGVGYLASSENRDLLEAMERQGFESMDEEKLMALIQSTLVESRRDGAQFQTITGLG

KWKKGNSLPAFDSPIFSRFRRLSLDAETSSSEGDRANAFRDSLALARTIEEASELVCAALVTKLAARSGISPENVDPSKT

VSEYGVDSLVAVELRNWITHEMDSTVPILELLANNPMNSLSVKIASRSKLVHLDAEKE

>BGC0001565.1|ATQ39432.1|MIBiG

MAPQRTSPPNGTAFPDADAPRYMYRPASPTTESSWEESVDGVDLAKPMAIIGMAMRLPGSVRSADDLWDLLSARKSGLCD

IPKNRFNADGFYDPARGPGTIPVKKGYFLHDVQIEEFDTNVFPIPRMELELLDPAQRQLLQVAYECIENAGGTSWRGSRT

GCYIGEFGEDFADSSARESQQRGNLRYTGLADFAIANRISYELDLQGPSMVVKTACSSSLVCLDLACKAIQSGECDSALV

GGVSMMFSPATYISVTDLGVISPNGQCRSFDAGADGYARGEAVNMVMVKRLDHALRDKDPIRAIIRGTGVNTDGRTNGML

TPSSTVQANLIRHTYKTAGIDDLSQTAVVECHGTGTPVGDPIETEAVGRCFGDEGITITSVKPNLGHAEAAAGLSSLIKC

VLSLEHRQVLPNINFKTPNPDIPFEKYKLRVPTEVESWPQGRAERISINAFGIGGVNAHAIIESPAQFGIKKPANGVASR

GGHTNGDAVPNGSHKQIEHDRHHQDQIAHLNGDYTNGTAINGANGNGTTINGANRNATAINGANGNGTAVNGKHADHGPD

LLVFSAYSSDSLDRHISAYRDFAATHQGTSLKDLAYTLSSRRDHRPYRAYAIADDVSGVQDASATVNAGGDGASPPVGWI

FTGQGAQWPEMGARLIDTNTAFANRIRKLDKYLQTLELEPPVSIEDELRKTKEDSRVHRPEMGHLVCVALQVALVDVLRS

WNIVPDFVLGHSSGETAAAYACGAITAEAAVYAATRRGIGNASSQRKGSMAAVGLGRDEIQPFLLEGVNIACENSQSNVT

LSGDSEQVESIVATLKAERPGVFARLLRVEKAYHSLHSADPEIPFYSSVTGDRLTGEGKLGASYWRANMENTVLFNPALR

SALRDRPAKLVLVELGPHPALEGPVGQILRDLGRADDIYLGTFIRGSECDRSLLHLAGKLYQQSIPMNLAAICPPGAVLT

NLPSYSWSQDTTHWEESRVTRGWRLREHPPHELLGSRVLETEGEPSWRKVMALEDALWLDGHEVNGQVVFPAAGYISMVG

EALRQLTGDTTFTVRNVRIMSGLVLGSDKPVELVTLLRSTTLGTSDDSEWYEFTITSFDGTAWARNCHGEAMASSDKSFQ

LDSISPAASSFPRKLDRRDSYDILRRVGFNYTGEFEGMAHISVSPSSLQAHGITSPGRQVTDKSSRCATYSVHPAVMDQC

FQLFTVALCRGLRRSNRRLAVPTFIEELVVSPSASPLSVTARINRLDEVGSWTGDFVALAAEEPVVRLKGMKSVVLATPA

QSEPPLSTELEWKPHSDFVGLEVGMHPRVPRKREWALLEELVLLCNLDHLDQIKPDDSTAPHLVKLLDWMRLVTDRYKSG

KNMFVSAGAGLEHLDHDERVARIDFLMAQLSDTQDIVFARAIHRLFIQTPPIFAGEGHPLHILMPDNVLSDFYDATSFDM

ADAVRLVANTNPHMRVLEVGAGTGSMTARLLRALTSSHGERLYSHYGYTDVSAGFMAVGKQRFADVENIDYDVLDISKDP

EEQGFKPGSYDLVIASNVLHATPTLDETLRNVYSLLKPSGRLFLEELTPDAMFVNYVMGFFYGWWLGAEDNRVEQPWIAP

ECWAEKLVAAGFQKPEAIVLDSEKPYQISAGIIASRGLRSTLPSKVAVLCHTANEPLAVEMIYRLSDMGIDVETCLWGQP

LPSCDIISVLELEKPLLHEMSEETFKTIIAYFQSHKARVIWVTEACQIDCGSPELAMMLGFARTTRNEYSLQMYTVEVDK

KTTTSRTTEAIIDIWGRVSTPDLDSESMDHDYEYALVDGRILVPRFHWQTMSGAFSGATRKETNDAEVALKHLNMGTPGL

LRTMKWIDGDVPAAPAKGKVLVEVKAVGLNFRDVILALGVVEGNPSGMGHEGSGVIRAVGPDVQDLSVGDRVMFIHDGCF

TTQLTLPKDICVKLDDSTSFIQGAALPAVYATALAALVDVSRLQRGQSVLIHAACGGVGLAAIQIAQNIGAQIYCTVGSE

PKRRYLEDTYNIPADHIFKSRDVTFLPDVMRATNGRGVDVVLNSLSGDLLHASWRCVAEFGLMVEIGKRDFQRRSKLSME

VFEANRSFVGLDIRGLSISRPGRAADLMRRCLDMIRSRAIQGPVACTTFPAAEIQDAYRYMQSAKHIGKIVIEMPDDPRE

DLGAGAPPEGGSTELEASPRPRPSFRSDRSYLLVGGLGGLGRAVATWMVEHGARVLVFLSRSARENDGNRDFLNDLRSEG

CTVLLVPGSVSSLEDVQTVVAIATAYQPLGGLINMSMVLRDVTLNKMTYSDWTTAVEPKVRGTWNLHHATAHCTSLDFFI

LFSSQNAQIGQWGQANYAAANTFLDAFAQYRHGHNLVASVIDVGLMGDVGFAAENRAILKKLGRIGMYILQETDLLDAIN

LALLKSQPVHETEDRKTRYVTPGYVGIGLNTTTPMSAASTRVSWKRDPRMSIYHNMGKSGGDASGENSSKGGSLKAVLAA

EPSEEKKTEIIAKALAGTLGNFLIKDGSNFPLDKPLKMLGMDSLIAMEVRNWIRQNIGAETSTFTVLQSSSFMHLAGEIR

AAMNAASEE

>BGC0001404.1|CAP95405.1|MIBiG

MGSIDNTARGSSASEPIAIIGMSAKFAGDATNTDNLWRMLIEGRSGWSPFPDSRFRSEGVYHPNNERLNSTHVKGAHFLA

EDVGLFDAAFFGYSGETAASMDPQYRLQLESVYEALENAGLPLTKIAGSNTSVFTGVFVHDYRDGFLRDADNLPRLMATG

TGVPMMANRVSHFFDLRGASMTIETACSSGMVAVHQAVQSLRTGEADMSIVGGANLTLNPDMFKALGSAGFLSADGKSYA

FDSRASGYGRGEGVGTLVVKRLSDALAAGDPIRAVIRESMLNQDGKTETITSPSLEAQEALVRGCYQKAGLDPRETQYFE

AHGTGTQAGDTIEAQGIATVFASRQEPLLIGSIKTNVGHTEAASGLASIIKTALAMENGVIPPSINFEKPNPKISLDDWN

LKLVREVETWPAGPIRRASINNFGYGGSNAHIILEDSASWVKAIGGQNGRTNGFADGHSNGPNANGHHSTLDPHVQESQV

ISKVLVLSGKDKQACEKMTANLADYLRQTQSTNSNPRELLDSLIYTLGQRRSRFPWVVAHPIPVTEGYETVVQTLQSPKF

KPTRTSRRPRIGMVFTGQGAQWNAMGRELIEAYPVFKASLQEAAGYLEQFGAEWSLMDELMRDAEKSRINEVGLSTPICV

AVQISLVRLLRAWGIVPVAVTSHSSGEIAAAYSAGAVSYKTAMAFSYYRAVLAADKSLRGPVKGGMIAVGLGLEETESYL

RRLSSEGQAAIACINSPSSITVSGDLSAVVELEDLANADGVFARRLKVDTAWHSHHMTPIANVYCEALENTRAEKIDRDA

LTTVAFSSPVTGGRITDAQQIARPEHWVESLVQPVQFVAAFTDMVLGGSGSVGSNVDVVVEVGPHTALGGPIQEILGLPE

FKDLNIPYYGTLVRKLDARDSMHALASSLLREGYPVNMGAVNFAHGRGQYVKVLTNLPSYPWNHQAKHWAEPRLNRAIRE

RSQPPHDLLGSIVEGSNPNAPSWRHILRMSESPWTRDHAIQSNVIYPAAGYICLAIEASRQLHVLNQTAGEIGGYRLRDV

DFLQALMIPDSSDGIEIQTTIRPVSEKDIASQGWRHFEVWSVTTDNRWTQHAKGLVSVELGESSVRMSRPARKNITGYTR

RILPADLFANLRNLGITHGPVFQNMDSIIQSGSEMRSVVSMTLPDVSVPNDLPRNHILHPVTLDSVITAPYSAVPGAAAR

EITAKVPRSVERFWVSSKISHDAGHSLEADTTLIRDDDQGMAADVLVSDHDTGNIMLEMNGFSYQSLGRSTSLQKSESWP

NELCNKVVWSLDISTPLPATLAAVRNELACTVQSAECDTTKATLRACIYFMQLALVALDSHDIAEMEQHNASYYTWMKDT

VELASSGKLFEGSAEWLYHSENERQLHIEQVQTRLDGEIVCRLGTQLVDILRGHTGALDLVMQDNLLSRFYSYAPRWKRA

GTQIAGLLRHLSHKNPRARILEVGAATGAIALHALGALGTSDSGGPNASMYHFTDTSTALFETARESLQPWADLLSFDEL

DIEHDPASQGYTPGTYDIVIASNIRSISESTSQALSNISSLLKPGGTLLLVEPLKYEVDVHFVRRLLPGRWWDDSTELKA

NLCLDMPSWENQLLSAGFTGVELELLDREDPQEAALVTFMSTVQLPQPPKSNVDADQVVIVTSRNGCPPAAWVKGLKDAI

AAYTVSEGKLGPIVQDLESLAATAASYADKICIFLGEVDEGILYNLNSTLLEGIRSMSTNSKGLIWVTRGGAVDCERPEM

SLATGFIRSLRNEYVGRKLLTLDLDPKGTPWSDVSMAAIAKILGTVIGNSAGGSMVEKGAVELEYAERDGVILIPRIYHD

VTRNRMLSPDASDAAMEKISIENFYQPTRPLCLKPDLLVFGDDDFSADYLEHLPPASLEVQPKAYGATLNSVGDHIAGFE

CAGIITQVGEEAAAQGYAVGDRVLSVLRHSSFPSRAVVDWKLTTRMPTDMTFQEGASLPLSFLSAYFALVEIARLQRSRS

VLIHAGAGDVGQAAIMVAQHLGAEVYVTVGSPAERGLLILKYGLPADHIFSCTDLSLANAVVAATQGRGVDVVLNSLTGP

LFQESLNLVAPLGHFVEIGRRNTQTNGYMHMRPFDRGISFATLDIPSLLEYRAMDVHRCLAELTRLIELKAVTPVHPITF

HAIGEIAEASRLLKAGDQIGKVVLSVDEHSTVTAVPSKPAAKLSSEVSYLIVGGSGGLAQSVAHWMVNRGARNLVLLSRS

AGTSEKTAAFAEDLRQAGCRRVLPISCDVANEESLGDAINQCAQEGLPPIRGIIHAAFVLRDAFVEKMTLDDWTYTIQSK

VAGTWNLHNQFNLPGDLDFFVLFSSINGILGYASQSAYSAAGAYEDALAHWRVKHCGLPAVSIDLSVVNAVGYVAEANAS

ETLRRSLLRAGRRVIDEDHVLGSLESAILSPFDPQFVVGGINSGPGPHWDLDGDLGRDMRVLPLKYRPPAVTGQSQDDDS

SSDSLAAKMIACESQGDAVRVVGTAIAEMLAEMFLVPIEDVDLGQSPSQQGVDSLVAVEVRNMLFSQAGAEVSIFNIMQS

PSLTQLAIDVVDRSAHVKLAG

>BGC0001254.1|BAJ14522.1|MIBiG

MTPLREPIAVIGSACRFPGGANSPHKLWELLRDPRDILREFPDDRLVLSKFYNGNANHHGSTNVRNRSYLLSEDIRAFDA

PFFHINPREADGMDPAQRILLEAVYEALEAAGYTMEQMQGTHTSVFVGVMNSDWWDLQMRDTETIATHAATGTARSIVSN

RISYVFDLKGVSMTIDTACSSSLVALHQAVQSLRSGESTAAIVGGANILLDPAMYIAESTLQMLSPESRSRMWDKSANGY

ARGEGCAAVFLKPLTRAIADGDHIECVIRETGVSSDGRTQGITMPSAAAQAALIKSTYRSAGLDPLADRCQYFECHGTGT

PAGDPIEAQAIAEAFFSHSGEDAEIYVGSIKTVIGHLEGCAGLAGLLKASLAIQNRTIPANMLFNDLNPLIGPYYRNLKI

LQAAKPWPQDIHGPRRASVNSFGFGGTNAHVILESYEPEMQGTHVLQERSFHGPLTFSACSKSSLLATISNFTSYIKTNP

AVDLQNLAWVLQRKRTEFPVKQHFSGSTHARLIESMEAYLQNAGSSGLHNTTIDTKLLYPSEIPRVLGIFTGQGAQWATM

GKEFIQNSYLFRESIDRSEAALVALPDPPSWSLISELFATVETSRLNEAELSQPLCTAIQIAIVDLMFAAGVKLDAVVGH

SSGEIAAAYASGIISAADAMAIAYYRGFHAKRSHGTGGKRGGMVAAELSYEAALQFCEKVEWAGRLVLAASNSPSSITLS

GDLDAVQEAHAYFEKENIFSRLLRVDTAYHSHHMIPCAEPYLTSLKACNIQVSQARSDCIWISSVSGDVQSSSEEQGALT

GEYWVDNMVKPVLFSQAVQCSIWNSGPFESIVEIGPHFALKGPTTQVMEAVLESSPPYLSFMRRGHGIETFSDGLGRLWS

KLGPSSVDLTGYWKACSSSHIKFQMLKGLPAYAWDHDKVYWKEGRISRNHRLRKDVPHELLGRRTADDSDYELRWRNVLR

LTEIPWIRGHKFQGQVLFPAAGYVTMALEASKALVGDRHVRLFELRDICIRKALVLEEDQSSLETVFSVKRLNADFGIHD

ENMDLLEAEFSCYVCADETVGTLEKTVSGRIIIHLGSGVDVKLPPAAHFCTDLSPVDLDRFYSTVEELGLSYQGLFKGLD

HAERMLNHSHALAVWENHSMGAGSMVHPALLDVMFQAIFVASISPAAPSTLWTPYLPVSIDRIIVDPDHIPVYSHPEVRA

HIQAYVTKSSASSIVGDIHLLDSNGIHNGIQVEGLSLKSVAEPTEENDRSIFSQTVWDTDIASGTDFLRDRKEDAQESKL

IHAIERVALFHFRSLVEAITVEKAKTLAWHHQLFLKAVKANIETIRTGGNPVVRQEWLCDTRETIEDLRAQHPGQIDLNL

MHAVSEKLISIVCGETQILEVMLQDDMLNDFYMRGRGFETMNNCIARAVQQIAHKHPRAKYLEIGAGTGGTTHRILDTIE

SAYTSYEYTDISSAFFEKASHKFDKHASKMVFKVLDIEKDVVDQGFENGAYDVVIAANVLHATRTLSGTMGHIRSLLKPG

GYLIFMEVTGDQLRLLFLFGALPGWWLGAGEGRSLGPGVSTIAWDNILRNTGFSGVDDVFYDFPDRSRHTCSVMISQAVD

DQLRLLRDPLAATGMPISEQVLIIGGDTSSVSQLAYDTKRLISPWASCVAINNIDGLDSRRLPSRFSVICLTELDKPLFS

EIMSEQRLSNLQNLFATANVVFWITSGCNEGTPVANMMVGIGRALATELPHLTLQFLDVKTVERLKPSIVAQSFFRLVLA

KPLVMAEKSMLWTTEPELVFDGDDILIPRVLPDKEMNNRFNAARRPISENLWKESTCIELSNADNSSAPALFEIKNTIRP

GETTIDVKYSVCLGKRCTFVLGVVSGTSDTVLAISDTNASSVRISKEHVFFLPHDFSGNSATLLLDTANHVLAAKLLRCI

SPNSIALIYEPGVRLAAAIRHHAHENTVDVFPATSNREKCGEGWAFIHPHATERDIRTIIPRNTGCFINLSFKPPGALSR

ALLQQTIIHGPDCLSQIVSSADGFLLEAAFNWATTGLLSLDSVETVSVQSYVGTTRPSRDFPLVFDWTAPRLPVTVKPLE

PKGLFLPDKTYLMIGMTGDLGRSLCRWMAEHGARYVVLTSRNAEVDSAWIESMAAIGATVKVYKMDVSNRKSVLGVYTTI

KNSLPTIAGVCNAAMVLEDRLFANMTVGALSKVFEPKVEGSKVLDEIFHELNLDFFILFSSLTSILGNGGQSNYHAANLF

MTSMCAQRRARGLAASVMHIGMVADIGYVARSDRHIENHLRKLQYHPMSETDMHYLFAEAVMSSRADHPGNWNIVSGIET

FVDAPGVKLRPPHYHNPRFAHYVREENARKEDLRTDKTEKSVKELLEDAISEEDVTTVFQQAFLIKLERLTQLESHRIDA

NKSLLNLGVDSLSAVEIRNWFLQTVGVDIPVLKLLRGDTVSEISIDATKKYLAQRTS

>BGC0001290.1|CBF87072.1|MIBiG

MSPASRSRVEIADSESDSERLSSSPWSILSDNDSNTSDERSTRAGPGSLEPIAVIGIGCRLSGSATDVSGLWDMLKSGRS

GWTPGPGTRFNMKAFQDPTGTRSGTTNATGGHFIREDISKFDATFFGINPVEAQAMDPQQRLMLEVAYEAFENAGITMDA

LWGSNTGVYVGQWASDYHEIATRDIERPPLYLVTGTGPAITSNRVSYVFNLRGPSFTVDTGCSSSLVALHQAVLSLRNRE

TTQCFVGGVNLLLDPQRFHYQSRLKMFSKDGRSFPFDARANGYGRGEGVTGVVLKPLSVALRDGDPVRAVIRNSVLNQDG

RTPGISVPSAVAQKEAIIRAYRQAKLDLYADYVEAHGTGTKVGDPIETSAIAAALTQRRSPSRPLPIGSIKGNIGHTESA

AGLAGLIKSVLMLENGMIPPQVNYETTNPDIHLEEWNLRIPTKLERQTLRRISLNSFGYGGTNAHVIIDAAHEAISAFGR

LSLSRHLQLSYHSEKPRVFMVSGASEKACQRVCARLARYLVVNHRNSINPDALLARLAHTLAKQSIHAYRVIFVASELDE

LIKQLITASHSTITRREKFGQHRIALIFSGQGAQYAEMGRDLLKSYPSFVRSLERARQQLSRLGCTWDLLSELCRPKADS

RVNEPAFSQPMCTAIQLALVDLLNEFGVSPSAVLGHSSGEIGAAYAAGALSFRDAISVSYYRGKLASELLAENQSPGAMI

AVGAPPDIAEQHINKLGTDVGRMRIACFNSPSSVTVSGDVAAIDRIKEVLDTEGLFNRKLITHGAAYHSHQMKLIEDKYI

AALKGLKAKPVSSSIRMFSSVTSKELDESTVLDGGYWAQNLVSPVLFSQALRTMCEQDYNGLPIDTLIEVGPHSQLSGPV

NQILKTIPGPHGQASYTNTLKRGDDAETALLRCLGFLAIKNGSVRLCDLNKDSKDSDIQPLADLPPYSFDHDRSFWHETR

LSRDYRHREHLPHELLGTLSADVNKLEPRWRRFVSLKETPWLRNHIIQGLITFPAAGYITMAIQAIRQHMHTANPASTIQ

FIRLRDVSFGKGLVLPDENAEVEISLSLRPQARTARESSGIWNEFRIFTVTPDQKWTEHCRGLVQAEVDSVEGFRSIFTP

ADISRIDSECTHGTIPQKFYAVGKRNGLDWQHPFNNLHQIRSSKHSCVATARVPEYEMPSGGMEDLLHPAVLDSALFHGL

STVIYLEDGRSSAYVPTFIKQLWVANRHVAPGSYLTCSTIRRNEPLVFDLHTKDEINQMAVVAQGIRVTSLGGDVAAGVS

KREACHTQTLVPYVDAWTTEHRDQVCRATIELGSLMETNRALDAITIHFAQNAIREISLNDIQETHLQRYFQWMGTLADE

TYDNILLENKPEDLGVIGEAIAILGPHLVDILKGKTSALSLLTKNNLLSRVYTEWCSSRLYPQMSAYCHELGRFNPQLKV

LEIGAGTGSATLPILKALNDCSGRFIQRYDFTDISPGFFEPAKERLGDLANVVEFRVLDAGRNAQEQGFEEGAYDLIVAC

NVIHATPRIDETLRNIRPLLKPGGKFMLMEISRYTLYFNIVFGLFEGWWLGYDEGRTRSPLLTDSEWCQRLEKAGFAHIE

KAFVDYPHENGGSLSVFISTAPFPRRNESLPIHLLTDSNASNATEEQAQEIQQACQTSVALLPITHPCQHGGVAILLPEI

AKLLCAEPDVNVWNSFKNWILKSRAVLLVSNCTMADSSHAETGLWAGFARTMRLEYPNLRQVVLDIQTPNVPVMSKLKEV

LPIILNSSSFDLDCLSSEVENEFTEKDGQLFVSRYAYRPDISRDVDLTSRQAASEPVPFVSTGRILTAELGVPGLLETIR

WKDDIECPPLGPDDVRFELRGASINFKDVLIAAGQLEGITEMRNDCSGVVVEVGENMKHRFKPGDRVCALYSRSYTNYPL

VHGDCCQVIPDSLSFAEGASLPIVWATVYYGLVDKGSLSKGEKILIHSAAGAVGQAAIMLAQHLGAEVFATVGSEAKRDL

LHAKYGVPYDHIFSSRTTAFYGEIMKSTGGYGVDVVLNSLSGEMFRESCNLMASFGRFVEIGRKDLMDDALMPMEFLLRN

ITFSYVDLTAIIEQRKPLARRLLHDIADLAASGSIRPVTLTTMPISDIEIAFRQIQAGKHTGKIVLTVEENQEVPAVPSM

PKQARLHEDASYIVVGGLGGLGRWLTTWLADHGAKHIVALSRSGAKDADSRTFISNIRGRGVNLIAPPCDVVCADAVVAL

AQELKRSELPPVRGVINSAMVLRDTLFDNMTEDDWRTALASKVRGSQNLHTTFKSLDFFVMMSSIVAVRGNYGQSNYSAA

CSFQDTFVRHMVQQGEPAFSINIGPIRDVGYVSENPEVAEALRRNGLGSIGVSDVLIVLNHAILNARGANPSTCVASIGL

IASDDESENGRDFLMTDRRFSQLVKHNGSKQKSAGEALDAITLLSAATQLDEAVHIVTNAILNQLSKLIVTPVEMLSPAQ

SLDSYGVDSLVAVELRNWIGAYLHANVQLMVIRGTGSISQLAAIVAKESRVVKL

>BGC0001749.1|EHA52508.1|MIBiG

MSPIFLGDSEDAATCRCGPPSSPSPELSGTETALTSDSDGPELLNPGPQGPEPIAIIGMGCRLPGGASTPSKLWELLEAG

RSAQGRLPADRYNMDAFYHPNGDRPGSMNTSGGYFIQEDVRGFDNSMFGINHLEAMYMDPQQRKLLEVTFEAFEAAGLSL

DAVSGANVGCYVGNFVTDFITMQLKDAEYTHRYTATGSGTTILANRISHVFNLKGPSFVIDTACSSSLYSLHAACSALWQ

RECDAAVVAGANLIQSPEQQLATMKAGVLSGTSTCHTFDASADGYGRADGIGVLLVKRLSDAIRDNDPIRSVIRSTAVNS

NGKTNGITLPSADGQEAVIRKAYALAGLGYGDTDYVECHGTGTAVGDPIEVEALSRVFRRQPGSQPLLIGSVKTNLGHSE

AASGISSLLKVAMALECGRIPPTIGISSLNPKLKLDEWNMRIVTENTEWPQNRTPNGQQGGRALRRAGVNSFGYGGANAH

CILESPDSHVPRGYRERGAATRLTNTTTGAPRTALLLPVSSKSASSLEQKSADIASYVAAKTASSADLQASELAYTLGVR

RSHLSSRGFWIAAPDSLSEDVVVGSDAASSKLHTRIPGRAYGRHPLAFVFTGQGAQWAGMGRELMDEFPSFRRTVQMLDS

TLQLLPHPPTWTLRGALLEPPESSSINLASRSQPVCTAVQLALVRLLRDWGVAPGFAVGHSSGEIAAAYAAGRLTARQAI

AVAYYRGYAVERSTTVGAMMAAGLSQDEADGDIAALGLAGKIRVACVNSPESVTISGDTDGIDEYKAVLDGRGVFARLLK

TDGRAYHSHHMAAIGGLYEDLVVEALASPAVQNDLDDAGQQNSSPAQWISSVTGQVVGDDMPTAEPSYWRANLESPVLFA

QVVEKLLSPGTPVHLVEIGPHSALEMPIKQTRTKVGIDAAKTPYNSALLRGKNSTTTMLTLAGELFLHGHPIAFGAVNNT

TTAHTHSRYPPTKPGMLLSARQPQVLTDLPRHVWEYDGGAGFHEPRSSIEWRNRTHARHDLLGSRVPGGDGITRQWRNVL

RAADLAWLVGHKLDTTTVLPAAGYLAMAVEAACQSTGLSLERYGRGSPRCSFALRHVHVEKALMVPDDQQSGIEVFTTLQ

PTTAPRTATAGSGGWYKFIVSSFVAGESTRHAHGLVKLTQNEDRPPARRLPVEDEAMEQSAPRTWYRRFVQEGLNFSGPL

QSLSRIETHRRRGEMHLLAQTSLSPGLGGESAYALHPIAIDALFQSGPIACTRGVVRDFTAKVPVYIKDMELRMPSRSLL

SGSFVSPSGDESAEATAQQGSIRTICKSAGLGAISVDSQLFDGDDLVLCVSGCRMVPYSSGTAVGAAAGDGYERHPMLHV

AWKPDVERLADAGIEHGASALTAYLSQFQGTTVIGDDADGVAGVLGAAKLAGGVLDLVVHKRPTLHVLLASDSTQDDGAA

KHMRELLGVGTAFQRCLSLWKRSTDEDGAVHFQDLSAKEDTTANGTATAASSAPPSIFDVLVILDLDTSTKGSTSADLAS

YSSLVDEKKGTLIWSGPPSSASTASSGSIPAKLSPLGFSCMEAQQSHGTPVLEVVLAQRGLPDKKQELGQQEVLIVERNP

DHKLNSELALHVAELTNKPAKRVTLDQLTPDLATAHATVIATVELEDALLADVKDGDFAQIKTLTDHCTNLIWVTGGGLA

DGTRPEQAVVFGLSRALMMEQPSLRFFVVGVDGECVAAETTARQVLGVARQALLDDAEPDFEFVQDGRAGGALQVSRFVP

DDAMNSTFRQRQPNATETLEMRLGDAHPCRLSLAGPVAANMSDAFVFTRDLTHKNNDHGIGQDEVEVQVLTVGLHARDLR

AMTGETSDGDGDTQPHAVTSQYVGRVVRVGSAVEGLGVDDSVLVMAPGRCATVERIPASSCSVLRDGEDPAAMASIPLPA

CTALYALRDRARLQPGETVLVCYREADAHGRDRSGPAAVHIARALGANVFAVVVVDDGDDEAKQEQRSEIVGDLGLPETH

VSFVKVGDGAGFGSDMLSSHGRVQVVANFCTDRWPLSNVAALCADDARIVHVGRGTVLGELVTTDPTILRKNIALSTFDV

NLLLTPVPSSPSTTRSGLLLDDVLSLWRQGKLNGLLGTQPRLFDVANLAEAFRALSGSTTKAGHTTPRGAVSVSFEATSL

VRVAPPNYHTVFNPDKSYLLVGCLGGLGRSMSRWMLSRGARKFTFLGRSGTDREPAARLVQYLELCGASVTVVRGDVVDA

SDVERAVAASAAAGPIGGVVQAAMGLDEALFTAMPAAYWRKGLAPKVRGSLNLHAALAGRDADLDFFLMTSSVSGSVGTA

TESNYCAANYFLDVFARHRRGLGLPATSVGLGMISEVGYLHENPEIEAMLLRKGIQAISEDEMLNMIDISLSASSSSRTR

GSPAAAWRGTDHALAHTLTGLEPIGVRELRAQGFDVSSPVLGDPRASLLAAALAADENESAGAGAGGASTSSGGLPAGLA

QAVAGGSAGAVAAQALELVADKFSNLVLVPRDRLDLLRPLSDVGVDSMLAAEFRGWIYQQLKVNVPYLTMLASTTTLTML

SELIAGKLLEA

>BGC0000045|AGC95324.1|MIBiG

MPSAQHSVGDVPIAVVGLSCRFPGDASTPSKFWDMLKNGKDAYSPTSTRWNSDAFYHPGDGRLNSLPTKGGHFLKEDPYV

FDAAFFNITAAEAIALDPKQRIAMEVTYEAFENAGMSLQQISGSQTACYIGSGPSDYRGAVERDFLHNPKYHLLGTGDEM

ISNRISHFLDIHGPSATVQTACSSSLMATHLACQSLRSGESEMAITGGISLMLTPDFTTHLNNLTFLNPEGLSKAFDESA

GGYGRGEGCGIIILKRLADAIQDGDDIRAVIRGTGANSDGFTQGVTMPSFEAQAALIRQVYSSNGLDYSTQYVEAHGTGT

KAGDPIETRAIYSTIGKGSPKPRKLFVGSVKPNIGHLESAAGVSGIIKGILSMEHNLIPPNLHFTKANPAIPFDEWNMAV

PTKLTPWPVAATKRMSVSGFGMGGTNGHVVLESFDSTRSTNGSGYSGGFSTFEKTRTKKRLFVFSSHDQAGFKRNANALA

EHLDTVGSVASSSDFMANLAHTLSGARSSLSWRATCIAENKIELRDYLTTKPGDGASRDATNATRAPRIGFVFTGQGAQW

ARMGVEMLDRPVFRDSVAQSTHYLQAMGCVWDPVAELKKTQADSRLSQPEISQPICSVLQIALVDELRSWGVTPSKVVGH

SSGEIAAAYSIGALSHRDAIAAAYFRGVATVRLRADAPDLKGGMMAVGCSRDEAEELIEQSKLDGTAAVACVNSPSSVTL

SGDVDTLEQLRAICDEHKVFVRRLKVEMAYHSRHMNRVSGTYAEFIADLQPIPREYNENEDDDSIQTMLSSVTGQEVAPE

LLGPYYWVRNLVSPVLFSDAVKEMVAPDEAEGDNTVDLLIEIGPHGALGGPVEQILGHHGVKHITYKSMLTRGRNALETS

LELASELFLKGVPIDISQVNSDLNPRRLTDLPPYQWNHSKVFRHETRIQRELVMRQFPSKSIIGAQVPMMDESQHVWRNF

LRLSDEPWIRGHKVGSTVLFPAAGLIGMALEAAQQLVEPSKTARSLRLRDISFFAAMALSEDVPTEVIMHLRPHLLATSG

STPAAWWEFTISSCAGIDNLRDNCRGLITIDYAETTSEQMASEDASLEASRIAHYHRVREESSYTYSKEDFYSQFEKIAW

NYGEAFRGVEKVYLGDGQATYDVKLVDIGETASKGQLDRPFLIHAGALDSILQGCLGSTYRNGRFDMDKPVLPTFIGQME

ISLDIPGDAGYVLPAVCESKRHGFKELSSNIYAFDSAVSKVNLSVVDYRVSELENDSGEQDSQQLEVDPAEITSEVRWNY

ALEVLEPEEIKKVVLAVAAEDRVVELIRLYLHNNPAATVIELVPDYEALERATMSLLPPGTILPSHIKYAVAATGSKSEN

QVDIENVIGTPFDLGDLDDTLPTDIAAADLLVIPQSVNNHKDLGVLLTRLTSFGKPDASLVLAVNSSVNVSNSMLESKGF

RRVFDLENSVALYKSRQSGHTNGHTNGHTNGTSTRSELFIIEPLATSSRINSFSGALQVTLREHGYPVFVTNWTEISARP

AADLEGNTFISLLELEQPLLDALSEPDFYSVRKLLLNSDRLLWITAGDNPSMGVVDGIRRTMRSEVAGLKFQVLHLSSLD

TALQCGPALAGRIMTTDTKDDEFQERDGMLQVARIFNSPEGNEGVRRCLEDSVRVERLGEQERALRLTIMKPGLMDTLTF

IEDDRMTGPLGATEIEVDVKATGVNFKDIMAAMGLVEVSLIGQEASGIVTATGSTAASRFKPGDRVTLLWEGMHVTKLRI

DHRLAVHIPDSMSFEEAAALPMVHTTAYHALVNVAKLRPGQSVLIHAAAGGVGQAALQLATHLGLVAYVTVGSEDKRRLL

MEKYNVPEAHIFHSRDTSFAKAIKRVTGGRGVDCVLNSLSGELLRVSWTCLAPFGTFVEIGLRDITNNMRLDMRPFSRST

TFAFINIANFFDPEGLDALGQILSDAFALVHKGVLGTAYPLTVYPVSELETAFRTMQQGKHRGKLVLSFGDNAQAPVLCK

ARDSLRLSPKSTYLFIGGLGGLGRSLAREFVACGARHIAFISRSGDSSAEAKATVQALTTLGANVKAYRADVSEEAAFLS

AMQQCATDLPPIAGVVQMAMLLRDTLFEKISYTDWTQPMRPKIQGTLNLHNYFSATRPLDFFVICSSISGIFGYPGQTQY

AAANTFQDALARHRRNQGLKGVAVDLGIMRDVGILAEQGTTGKLADWEAILGIREKPFHALMKSVINSEWKGAVPPPAQL

CTGLGTADIMARFGLERPEHFSDPRFGPLNVLSIESSSSLSTDQDTASSPSTRLAAATTLDEAVVIITDALVHKMAEILQ

MPLSEVDPGRPMYRYGVDSLVALEVRNWITRELQANMALLEILAAEPMRVFAGKIAEKSKLVAGRKG

>BGC0001600.1|CEF75886.1|MIBiG

MQGPTNEPIAIIGTGCRFPGGSNTASKLWDLLKDPKDVSKEVPEDRFNLDRFYHKDSSHHGTANVRRSYLLDEDVRLFDT

QFFGISPGEAQAMDPQHRVLLEVVYEAIESAGKTIHGLHNSDTAVYVGLMCTDYYVIQAADLNSVPTYNATGVANSNASS

RVSYFFNWHGPSMTIDTACSSSLVAVHEAVQALRNGTSRMAVACGTNLILSPLPFISESNLSMLSPTGKSRMWDADADGY

ARGEGVAAVVLKPLSAAIEDNDVIECIIREVGVNQDGKTRGITMPSAQAQASLIRQTYAKAGLDPATPEGRCQFFEAHGT

GTPAGDPQEAEALKTAFFPNETDSVTNGTNGLLSEADNLLVGSIKTVIGHTEGTAGLAGLIKACMALKHGAVPPNLLFNR

LNPALEPFTKHLSIPTSLTPWPTLLTNVPRRASVNSFGFGGTNAHAILEAYSQAPQNSFATPSSSPLVVPAIPFVFSAAS

ETSLRGVLESFLEYLNTSKDKDESLDLTSLAYILSTKRTVLSQRVSIIASTFEQLLEKVEAVLDDSASSVVGSKAATLSH

PALLGVFTGQGAQWATMGTKLMRSNPLAQSVIQDLDAVLASLPECHRPRWSLGRELLADTTSRIKEAELSQPLCTAVQIM

LVDLLKANGVQFQGVVGHSSGEIAAAYAAGFVSSADAIKIAYYRGYFAKLASGSSSTGKDSSVKGSMMAVGTTYEDAIEL

CQLEDFRGRISLAAHNGPNSVTLSGDSDAINQAHFIFSEEEKKFARLLKVDTAYHSSHMQPCVSPYTEALQACGIVAREP

ASDAPKWFSSVRSGKPVLDVDGLDCQYWVDNLLSPVMFHEAVQGCLDSSDTYNAILEIGPHAALKGPLDESVLELMGNKL

PYTSALVRGKDDIESFSTALGFLWTQFGNQCVDLGTFQKRVSKDTGSKLCSTMDDLPTYAWTHDKPLWAESRSTKLFRTM

PGSFHDLLGIQTADGTAEEWRWQNILKTKELPWMVGHALQGQIVFPGTGYIALAMEASLQIAQGRPVSKIDLYDLEIRKA

IAVNESASGTELLVTMTNVSAIHPDVETITADFATYSTISRESGSMALNCCGKVCIFLQTETVTTTGSDGHAAEQFATRS

TPVPGMAGIDVERFYSAMQHDLGYMYSGPFRGLSRLSRKLGFSEGSIQRPPFGEDGSETTLIFHPGMLDNALQGLFAAYS

APGDGRLWSMRAPTACRRVSLVPSLCGPNMTEEVDFDCTLTDSRDDFITGDVEVYASGYSQRIIEIEGLSFSPFAAATDR

DDRQLFQEQIWCVNEADGPLVLGNMAPTFEERTKALDAERAAFFYLKKLHLSVPSDQRSQLPWYRQSLLDNAERLYDLVC

SGTHSYAPQSWIQDTKEDVYAMMESYGPQDADFNLTKAVGENLPLPDVIKGDTNILQYMTQNNYLDRYYTHAIGFGWLNV

LISGVVGQIADKHPKMRFLEIGAGTGGATGAVLDRIGQAYSSYTYTDISSGFFERAVDKFQDHAGKMLFKMLDIEKDPVS

QGFPEHSYDIILAANVLHATKNLTETLQNTRRLLKPGGFLVLMEILGNDVMRIGLVMGGLPGWWVGKDDGRRWGPTITLE

EWDTLLKGTGFAGVDTNTPMPDKVQMPGSVFVAQAVDDRIVKLRDPLQHDALPSAATDHVNGIQNGHTPSPTISKGTSHL

VVIGGSSTSGSKLASDIIRVLSPLFAEIIHIPQLDSKDAIAKIPSNVDLHILSLTECDTGGTFFHNISNTAWQNFQHLLA

TSPASLLWVVPNTRSGNPLGAIGTGLFRSLFYEIPETKFQVLDLDEKATGYLSGCAGLIAKLVQQLRLVTDTSSARGPST

LTPETSEDDLQSVNDGTATVEMLWTVEPELYLHDGRLYISRVRLQKAQNDRYNSWRRPILQLTESKSTVDPSLSTSSLGR

QTSLELQWKDDAYYNLKEINWFAKPLSTDSATIDVSCSLASCLKTPAGFFFVQVGTDVNTGEKKLCLSTENRSRVTVHSS

WTETLKQEHDVADGQYMSFIVADMIVQQIMYMLPPTGILLLHEPDPGLASLLTRQLANIGRKVVFTTTRSDKSTNLLSKA

NWIFMHPRLNKRLIESALPHGVTFFIDCGQAEDVIHEGSHGKDHGLGLRLHNSLSRTCVKRTLQDLTSRTASVAPHEATG

EVVKLLHRITTFAAAQLNSVPDGAPLKVKSLSEIVSRAKTRALATAEGCSTGPFCLVNWHAESQVPISVAPVWDRDDLFR

SDRTYWMLGLTGDLGRSLAEFMISRGARHVVLSSRTPQPDEMWVERQQKKYGATVVYIAVDLTSLDSVQKAHKQIVKSMP

PLAGVANGALVLKDSSVAKMTIEQLQAVLRPKVDGTLHLQSVVDANSGSEEQPLDWFIAFSSIVGTTGNLGQAAYSAANG

FLKAWVSQQRSMFGHNAAVIDISRVLGVGYVERETQSNSGRLTREQTDRLMNRTGTLAMSETDLHQLFAEAVVAADHCSA

SNTGLSVGARDAEIITGIAPISSAQAEDVFWARNPRFGLLVIDSNAAVGGDDQDGKGSERRQVPVKTQLAAANTPQEVTS

VLTSCIVTKLRASLFLSASDSFSETVALVDQGVDSLVGVDIRTWCIKELDVDVPVLKILGGASVVDLADYILESLPVKEK

SK

>BGC0001252.1|BAN19720.1|MIBiG

MEKDTPVAIIGVSYRAPGIGGKGLWDYLAEAKSAWTKVPPERFEHFAWYKAGEKKTGVFANEGAHFVDNVFDFDAAFFNM

RADEARCADPSHRFMLEVALEAAENAGQSLLDLSGKKIGVFVGAGQHEYSHRVSDDEYAIQTFTATGVAPCMAANRLSYF

FDIDGPSVVLDAACASSAYAVDMAVKAIRNGDCDGAFVGAAALNLSPSGWLVLDQSGTLSDIGRSFSYDAKASGFGRGEG

AACLLIKRLEDAIRDGDPIQALIRGTACSHSGRSEGITMPSRRAQEKLIWDVHNSAGLDPSNTAVVEVFSRGHGTGTAVG

DPIEAGAFTSVLARNRTAANPIYIGSLKSNFGHLEGASGVLAMIKAVLMVRNGVVLPTAGFERINEAIDNYEKIKVPTTP

LPWPENEPRRCLVTNFGFGGSSSAVIIDRSPYLHALDGYEDLADIKIPRLNGSSGRSESGSGQSQRLFVFSAKTRDSLTA

YLASFHEYLLKAQESHEFLKDLSYTLGQRRTHHAYRASVVANSISDLRKEIPNLKPSKIRQRSVIFVFTGQGAQYARMAY

NLRQFTVFAETLEKAETQLNKMGASWSLTEELNKLTDTRINDAEISQPACTAVQLAMVALLQSWGVVPNMVTGHSSGEIA

AAFTAGLLTFQEAIAISYFRGQSAVQLSAAQHEYKKGAMLALGVGSEDALKIIDEHAQGYATVAAINSPRSVTISGDKTA

IENVRKAADMQGLFARMLKVEVAYHSRHMEQVAASYLKDIEPYFQGKAIPAENSGACRPVFVSSVTGQIIDAVDSSYWIK

NLVQPVLFADAIKEVLTHEDQGKSQSIHGSSKTLVEIGPHAALKNPVKQTAELLSSERAWNLASLNYLPSLLRGTNDVHA

ILELARALFDLGASVELSGVNGTNKHNARVLTELPSYAWDRSYYELRPRVTHDKQFPGEEYHALIGRKAPSNAAQENTYR

QVFTLDEMPWIRDHVVSGVTVFPMTGYMSCAIEAARRVDSAAPAAFLITDFHVVQSLEIHEEETVDLTTKLKPAATGEGT

FSSKVWSFEVVSWSEANGWTRHCWGKIEPEIADLTLATPTFEASLPLVTSMAGVIEHDMDNEYHNIELRGTKYGPSFRNN

VKFYEGKNYTVLEHRIRDLGDALKIPVYRGSPVSVDPPTLDSFLQGAGPFQYDGSGRRLTQMPDYISRFRISNNITSEPN

HRLDVVMRRLDYDDKGGRMHVSVAVFGRGSDDQFTPIAEWESFTFRTVSSADDQSASVPDNWSWELLPRYDLISKDTLRD

RLLESVGDLGEEEDVRMSKLDAVGCYYIEKALKDTVTLDYSKLPTHLARFVHWGRNVLKEYEVNFESEPTSLLEDVRNLD

AQGELLCLMGENLVDILAGRIEPLEVMLTDGRLMRHYEADVANAHLSKIIGYLTENMADLEPWQRILEIGGGTAGTTLPV

LEGLSRNRDEPGCLDYTFTDISSGFFEMASKKLSRWSQQITYKRLDITQDPAMQGFTQESYDVVVAANVLHATADMVTTM

THVRSLLKPGGKLILLEATRHPPWLLPFTLLPDWWAAKDKYRDHKQGPMMPAVVWNDLLLDSGFSGVDVVIPTNYRTDNP

LMNVVCSTRIGKQDDSETITICGPLVDDTEVNFAQSVARSISKELGCPTEIKRFADIKPDDESYYILLDSKHESVFQNFN

PGKFECLKSLLLRNKGLLWVTAKGCSPDAKMIQGMVRTVRLEVEPKNLMLLDNVPSTPEGLSGILKLAARLRDPEVSRDQ

DMDFAWHDGAIHLPRMRQLKDLKEQFSVEEGVAFRRTQNLRDNSDRGLEMTIQAAGSPDTIYFRRTDPYEVSEDEVLVRV

EAAGVGHRDFEVLMGSIPWAPPGYEGAGKVLKIGSQVSHLREGDDVFFLTPEASAFATEVKLASWLVARIPKNMTVTDAA

ACPLGYCLATLAFRTARLTKNETVLIHSAASSVGQACILLAQDIGARIYVTAGTEDKRDYLHQALGIPRDHIFSSRTAEF

RDSLLCKTNNRGVDVVVNSLSGELLTETWAVIAAFGRFVEIGKKDAFLNNSLPMRPFNNNVTLSAIDLRDLYHHRPDDVR

SVWNEVVNLLQRKQVRPVDSASVVSISHFSAALRILRSRDHIGRLVVTLGDDNSVMAETALRPSQVSLKDDATYLVAGGT

RGIGLDLAYWMIEHGARNIVLLGRSGASGPEAQKILNKFRNTKVCVRAVACNVGDRDELQNALESIKDLPAIRGVVHSAL

LLSDKLFVNASYEDWVINTTPRVAGAWNLDDLLPTDLDFFVALSSFNGDTGHTGQAIYAGTAGFYNAFSQYRNNRGQYTV

SIGLPVVLDVGYVADHDLRGGLLNDSLSAGVTMADIRATFNCILLGPSSPFVRNGRASTFKVYINGQPVQDVTWNYFHPA

HSKVRLTNANRNKVKATSGGAEISSASWTTAEDPLTGLIEALIAKVSAMTMMEREDVLPDAPLASYSLDSLVSVELRNWI

RRETTAEMTLVSITKAENLRALAVNILAQRKAG

>BGC0000039.1|BAC20566.1|MIBiG

MNNTPAVTATATATATATAMAGSACSNTSTPIAIVGMGCRFAGDATSPQKLWEMVERGGSAWSKVPSSRFNVRGVYHPNG

ERVGSTHVKGGHFIDEDPALFDAAFFNMTTEVASCMDPQYRLMLEVVYESLESAGITIDGMAGSNTSVFGGVMYHDYQDS

LNRDPETVPRYFITGNSGTMLSNRISHFYDLRGPSVTVDTACSTTLTALHLACQSLRTGESDTAIVIGANLLLNPDVFVT

MSNLGFLSPDGISYSFDPRANGYGRGEGIAALVIKALPNALRDQDPIRAVIRETALNQDGKTPAITAPSDVAQKSLIQEC

YDKAGLDMSLTSYVEAHGTGTPTGDPLEISAISAAFKGHPLHLGSVKANIGHTEAASGLASIIKVALALEKGLIPPNARF

LQKNSKLMLDQKNIKIPMSAQDWPVKDGTRRASVNNFGFGGSNAHVILESYDRASLALPEDQVHVNGNSEHGRVEDGSKQ

SRIYVVRAKDEQACRRTIASLRDYIKSVADIDGEPFLASLAYTLGSRRSILPWTSVYVADSLGGLVSALSDESNQPKRAN

EKVRLGFVFTGQGAQWHAMGRELVNTFPVFKQAILECDGYIKQLGASWNFMEELHRDELTTRVNDAEYSLPLSTAIQIAL

VRLLWSWGIRPTGITSHSSGEAAAAYAAGALSARSAIGITYIRGVLTTKPKPALAAKGGMMAVGLGRSETNVYISRLNQE

DGCVVVGCINSQCSVTVSGDLGAIEKLEKLLHADGIFTRKLKVTEAFHSSHMRPMADAFGASLRDLFNSDNNNDNPNADT

SKGVLYSSPKTGSRMTDLKLLLDPTHWMDSMLQPVEFESSLREMCFDPNTKEKAVDVIIEIGPHGALGGPINQVMQDLGL

KGTDINYLSCLSRGRSSLETMYRAATELISKGYGLKMDAINFPHGRKEPRVKVLSDLPAYPWNHQTRYWREPRGSRESKQ

RTHPPHTLIGSRESLSPQFAPKWKHVLRLSDIPWIRDHVVGSSIIFPGAGFISMAIEGFSQVCPPVAGASINYNLRDVEL

AQALIIPADAEAEVDLRLTIRSCEERSLGTKNWHQFSVHSISGENNTWTEHCTGLIRSESERSHLDCSTVEASRRLNLGS

DNRSIDPNDLWESLHANGICHGPIFQNIQRIQNNGQGSFCRFSIADTASAMPHSYENRHIVHPTTLDSVIQAAYTVLPYA

GTRMKTAMVPRRLRNVKISSSLADLEAGDALDAQASIKDRNSQSFSTDLAVFDDYDSGSSPSDGIPVIEIEGLVFQSVGS

SFSDQKSDSNDTENACSSWVWAPDISLGDSTWLKEKLSTEAETKETELMMDLRRCTINFIQEAVTDLTNSDIQHLDGHLQ

KYFDWMNVQLDLARQNKLSPASCDWLSDDAEQKKCLQARVAGESVNGEMISRLGPQLIAMLRRETEPLELMMQDQLLSRY

YVNAIKWSRSNAQASELIRLCAHKNPRSRILEIGGGTGGCTKLIVNALGNTKPIDRYDFTDVSAGFFESAREQFADWQDV

MTFKKLDIESDPEQQGFECATYDVVVACQVLHATRCMKRTLSNVRKLLKPGGNLILVETTRDQLDLFFTFGLLPGWWLSE

EPERKSTPSLTTDLWNTMLDTSGFNGVELEVRDCEDDEFYMISTMLSTARKENTTPDTVAESEVLLLHGALRPPSSWLES

LQAAICEKTSSSPSINALGEVDTTGRTCIFLGEMESSLLGEVGSETFKSITAMLNNCNALLWVSRGAAMSSEDPWKALHI

GLLRTIRNENNGKEYVSLDLDPSRNAYTHESLYAICNIFNGRLGDLSEDKEFEFAERNGVIHVPRLFNDPHWKDQEAVEV

TLQPFEQPGRRLRMEVETPGLLDSLQFRDDEGREGKDLPDDWVEIEPKAFGLNFRDVMVAMGQLEANRVMGFECAGVITK

LGGAAAASQGLRLGDRVCALLKGHWATRTQTPYTNVVRIPDEMGFPEAASVPLAFTTAYIALYTTAKLRRGERVLIHSGA

GGVGQAAIILSQLAGAEVFVTAGTQAKRDFVGDKFGINPDHIFSSRNDLFVDGIKAYTGGLGVHVVLNSLAGQLLQASFD

CMAEFGRFVEIGKKDLEQNSRLDMLPFTRDVSFTSIDLLSWQRAKSEEVSEALNHVTKLLETKAIGLIGPIQQHSLSNIE

KAFRTMQSGQHVGKVVVNVSGDELVPVGDGGFSLKLKPDSSYLVAGGLGGIGKQICQWLVDHGAKHLIILSRSAKASPFI

TSLQNQQCAVYLHACDISDQDQVTKVLRLCEEAHAPPIRGIIQGAMVLKDALLSRMTLDEFNAATRPKVQGSWYLHKIAQ

DVDFFVMLSSLVGVMGGAGQANYAAAGAFQDALAHHRRAHGMPAVTIDLGMVKSVGYVAETGRGVADRLARIGYKPMHEK

DVMDVLEKAILCSSPQFPSPPAAVVTGINTSPGAHWTEANWIQEQRFVGLKYRQVLHADQSFVSSHKKGPDGVRAQLSRV

TSHDEAISIVLKAMTEKLMRMFGLAEDDMSSSKNLAGVGVDSLVAIELRNWITSEIHVDVSIFELMNGNTIAGLVELVVA

KCS

>BGC0000089.1|AAD34559.1|MIBiG

MTPLDAPGAPAPIAMVGMGCRFGGGATDPQKLWKLLEEGGSAWSKIPPSRFNVGGVYHPNGQRVGSMHVRGGHFLDEDPA

LFDASFFNMSTEVASCMDPQYRLILEVVYEALEAAGIPLEQVSGSKTGVFAGTMYHDYQGSFQRQPEALPRYFITGNAGT

MLANRVSHFYDLRGPSVSIDTACSTTLTALHLAIQSLRAGESDMAIVAGANLLLNPDVFTTMSNLGFLSSDGISYSFDSR

ADGYGRGEGVAAIVLKTLPDAVRDGDPIRLIVRETAINQDGRTPAISTPSGEAQECLIQDCYQKAQLDPKQTSYVEAHGT

GTRAGDPLELAVISAAFPGQQIQVGSVKANIGHTEAVSGLASLIKVALAVEKGVIPPNARFLQPSKKLLKDTHIQIPLCS

QSWIPTDGVRRASINNFGFGGANAHAIVEQYGPFAETSICPPNGYSGNYDGNLGTDQAHIYVLSAKDENSCMRMVSRLCD

YATHARPADDLQLLANIAYTLGSRRSNFRWKAVCTAHSLTGLAQNLAGEGMRPSKSADQVRLGWVFTGQGAQWFAMGREL

IEMYPVFKEALLECDGYIKEMGSTWSIIEELSRPETESRVDQAEFSLPLSTALQIALVRLLWSWNIQPVAVTSHSSGEAA

AAYAIGALTARSAIGISYIRGALTARDRLASVHKGGMLAVGLSRSEVGIYIRQVPLQSEECLVVGCVNSPSSVTVSGDLS

AIAKLEELLHADRIFARRLKVTQAFHSSHMNSMTDAFRAGLTELFGADPSDAANASKDVIYASPRTGARLHDMNRLRDPI

HWVECMLHPVEFESAFRRMCLDENDHMPKVDRVIEIGPHGALGGPIKQIMQLPELATCDIPYLSCLSRGKSSLSTLRLLA

SELIRAGFPVDLNAINFPRGCEAARVQVLSDLPPYPWNHETRYWKEPRISQSARQRKGPVHDLIGLQEPLNLPLARSWHN

VLRVSDLPWLRDHVVGSHIVFPGAGFVCMAVMGISTLCSSDHESDDISYILRDVNFAQALILPADGEEGIDLRLTICAPD

QSLGSQDWQRFLVHSITADKNDWTEHCTGLVRAEMDQPPSSLSNQQRIDPRPWSRKTAPQELWDSLHRVGIRHGPFFRNI

TCIESDGRGSWCTFAIADTASAMPHAYESQHIVHPTTLDSAVQAAYTTLPFAGSRIKSAMVPARVGCMKISSRLADLEAR

DMLRAQAKMHSQSPSALVTDVAVFDEADPVGGPVMELEGLVFQSLGASLGTSDRDSTDPGNTCSSWHWAPDISLVNPGWL

EKTLGTGIQEHEISLILELRRCSVHFIQEAMESLSVGDVERLSGHLAKFYAWMQKQLACAQNGELGPESSSWTRDSEQAR

CSLRSRVVAGSTNGEMICRLGSVLPAILRREVDPLEVMMDGHLLSRYYVDALKWSRSNAQASELVRLCCHKNPRARILEI

GGGTGGCTQLVVDSLGPNPPVGRYDFTDVSAGFFEAARKRFAGWQNVMDFRKLDIEDDPEAQGFVCGSYDVVLACQVLHA

TSNMQRTLTNVRKLLKPGGKLILVETTRDELDLFFTFGLLPGWWLSEEPERQSTPSLSPTMWRSMLHTTGFNGVEVEARD

CDSHEFYMISTMMSTAVQATPMSCSVKLPEVLLVYVDSSTPMSWISDLQGEIRGRNCSVTSLQALRQVPPTEGQICVFLG

EVEHSMLGSVTNDDFTLLTSMLQLAGGTLWVTQGATMKSDDPLKALHLGLLRTMRNESHGKRFVSLDLDPSRNPWTGDSR

DAIVSVLDLISMSDEKEFDYAERDGVIHVPRAFSDSINGGEEDGYALEPFQDSQHLLRLDIQTPGLLDSLHFTKRNVDTY

EPDKLPDDWVEIEPRAFGLNFRDIMVAMGQLESNVMGFECAGVVTSLSETARTIAPGLAVGDRVCALMNGHWASRVTTSR

TNVVRIPETLSFPHAASIPLAFTTAYISLYTVARILPGETVLIHAGAGGVGQAAIILAQLTGAEVFTTAGSETKRNLLID

KFHLDPDHVFSSRDSSFVDGIKTRTRGKGVDVVLNSLAGPLLQKSFDCLARFGRFVEIGKKDLEQNSRLDMSTFVRNVSF

SSVDILYWQQAKPAEIFQAMSEVILLWERTAIGLIHPISEYPMSALEKAFRTMQSGQHVGKIVVTVAPDDAVLVRQERMP

LFLKPNVSYLVAGGLGGIGRRICEWLVDRGARYLIILSRTARVDPVVTSLQERGCTVSVQACDVADESQLEAALQQCRAE

EMPPIRGVIQGAMVLKDALVSQMTADGFHAALRPKVQGSWNLHRIASDVDFFVMLSSLVGVMGGAGQANYAAAGAFQDAL

AEHRMAHNQPAVTIDLGMVQSIGYVAETDSAVAERLQRIGYQPLHEEEVLDVLEQAISPVCSPAAPTRPAVIVTGINTRP

GPHWAHADWMQEARFAGIKYRDPLRDNHGALSLTPAEDDNLHARLNRAISQQESIAVIMEAMSCKLISMFGLTDSEMSAT

QTLAGIGVDSLVAIELRNWITAKFNVDISVFELMEGRTIAKVAEVVLQRYKA

>BGC0001124.1|EHA19289.1|MIBiG

MDTPLSSSEISPRFSNTVPSSVSSMTPNADPSVIVGLACRVPGATNPSQLWENIVAQKDLQRKMPADRFNVDAFYHPDGT

NKGTTNAKFGYFLDQDIGMFDAGFFRISGKEAEAMDPQQRLLLEVVYEALEDAGITLDEVNGSNTAVYCGSFTNDYNAMV

TKDLEYYPKYTVTGTGNAILSNRISYFYNLHGPSVTIDTACSSSLVCFHLGNQSLSQNESDIAIVVGSALHFDPNVFITM

TDLGMLSSDGRCRTFDSMGSGYVRGEGICAAVLKRRRDAVYSGDNIRAVVRASGVNHDGIKQGITLPNTDAQEKLIRRTY

DLAGLDPNDTQYFEAHGTGTARGDPIEARAIGAVFGSTRSEPLYVGSVKSNIGHLEGASGLAGIIKATLALEKSQIPPNM

HFKRPNPEIKFDEWKIQVPQDIINWPASANGIRRASINSFGYGGTNAHVILDAYKPEDSEAELQAIPAISSSIPVDRPYL

IPLSAHSTKAGALWEDKLTKYLSNEPIGRPAVSDLAVSLSTRRTMHGNRSFIIGKDMPTVLQGLEQPPSPAAAWTRPLKE

TPRLGFVFTGQGAQWFAMGRKLIQQSYLYRQTLERCDAVLQSLPDGPDWTVLEELLRTEEASRLKETRLSQPICTAMQLA

TVCLLKQWGIEPSAVVGHSSGEVAAAYAAGILTFENAMIAAYYRGLYMSSGVDGSMTTDGAMMAVGLTEAEAKKELETYT

GQICVAAVNSASSLTLSGDKDAIVRLRDSLVERKIFARLLQVAQAFHSHHMLPLAPKYEEALKNCVGFGTSPARVRMFSS

VTARLARPGEMGAGYWTANMTGTVRFSDALTGILLNEEDEQNVDILVEIGPHPALKGPSRQVMNALKLNLPYLASLTRGV

DDYESMLTLAGQLFQYGFPVDLIAVNSDHFLRRETGIIQSELHGKRLRDLPTYAWDHKRYWSETRPIREHRLRKQRHSIL

GARMPGMPERTPHWRNYLRLKEIPWLADHVIDGNAVFPAAGYFSMAIEAAVSMCAEDSVIKEIALRDLNVQSALLLSDSE

EGTEVIMELRPATQSAKSKSALWYEFTIYSYGETKILNEHCSGLVSVETNALTLPMRWESSKTFDDLAKESQESIPAETL

YDHLTALGLQYGPSFQLLTGDVQTGPGFALAGLDFQPSQFSVQAADLTIAHPTLLDASFHAIFPAIESALGRSLDEPLVP

TFVRSLKVSGDFLACCRESREQKFQVTCFTRLPGPRVALSDLTVCSKESNMPLLQFKGLEVTALGSDKTDNSAGRSLFFR

TRWQPAFTFLGPDHPAVAQKNVSEILDFFAHQFPDTRILHISDTVDGTRDVLKYLGGRSNERRRFHSITPVFQTQIALEE

IDALSQEWPGLVEISEPEPNAYGLVVLSSDAAGLDSRQFVKEGGFVLALGPHPQPEGLHDVFFTKDLAVWQKSTDNAQKP

KQLSLILPSCPSQRTLDIADGMETQHGSSVFVTRTSLAALSNEALQAEDIVVLANLDEDVLFEHSSSDQSTFLAIKRLLT

TGGKNIVWVLEGGSMDAPKPEHAMIIGLARVARSENDQLRFVTLDLPRASTQETVVRHVWRLLDRSITEDEVTVRDNCIF

IPRIEADDQLNSKLRNGTNSQPREEPLGAGRPLALKIGRVGLLETLVFEDDEQILDTQLADDEIEIEVKASAINFRDIAA

SMGIIDDYKLGDECAGIVTRIGAQVNPRDFQVGDRVAAWRPGQGAHKTIVRNPASLSYKLGDMSFVDAASLPCILTTAYY

SLVHVAHLQPGETVLIHSAAGGVGQMAIQVAQYVGARVIATVGSQAKRSLLKSRYGLADDMIFNSRDDSFVRDVLDTTGG

RGVDVILNSLAGKLLHATWSCVAPFGRFIEIGKRDIHENSKIDMDPFRRNVAFASVDLITIFEKNKPLGARLLKECGTLV

HEGHITPPETVTELPYSDAVKAFRLLQMGKHTGKVVLVPHAGDRVLVRPSTYRNQPLFKHEKTYLLVGGLGGLGRTLAEW

MVRKNARRLAFLSRSGADKEEAKRTVEWLRERGVSVTVFKGDVSRYEDVECAVKAIDNLGGIFQAAMVLQDAPLENMSYQ

QWQICVEPKVKGTYNLHQATLGTQLDFFICFSSASGSIGSKGQANYSSANCYLDALMRHRREMGLAGTTMNCGMIVGIGA

VAANQALLKVMMRSGYDGVNKEELLYQIEEAVLSDNSKKVSRRGVDLHQTITGINMTKADFYWCQKPLYRNLYNNHEFLG

QTAIKQGTKSLASQLQGTKSIEERTTLVLSAFIEKVADVLSVSVDSIEPANPLSAYGLDSIIAVEFRKWFSRSVGVEIAL

FDVLGAPSILALVTKASGLITITTSND

>BGC0001253.1|BAJ14522.1|MIBiG

MTPLREPIAVIGSACRFPGGANSPHKLWELLRDPRDILREFPDDRLVLSKFYNGNANHHGSTNVRNRSYLLSEDIRAFDA

PFFHINPREADGMDPAQRILLEAVYEALEAAGYTMEQMQGTHTSVFVGVMNSDWWDLQMRDTETIATHAATGTARSIVSN

RISYVFDLKGVSMTIDTACSSSLVALHQAVQSLRSGESTAAIVGGANILLDPAMYIAESTLQMLSPESRSRMWDKSANGY

ARGEGCAAVFLKPLTRAIADGDHIECVIRETGVSSDGRTQGITMPSAAAQAALIKSTYRSAGLDPLADRCQYFECHGTGT

PAGDPIEAQAIAEAFFSHSGEDAEIYVGSIKTVIGHLEGCAGLAGLLKASLAIQNRTIPANMLFNDLNPLIGPYYRNLKI

LQAAKPWPQDIHGPRRASVNSFGFGGTNAHVILESYEPEMQGTHVLQERSFHGPLTFSACSKSSLLATISNFTSYIKTNP

AVDLQNLAWVLQRKRTEFPVKQHFSGSTHARLIESMEAYLQNAGSSGLHNTTIDTKLLYPSEIPRVLGIFTGQGAQWATM

GKEFIQNSYLFRESIDRSEAALVALPDPPSWSLISELFATVETSRLNEAELSQPLCTAIQIAIVDLMFAAGVKLDAVVGH

SSGEIAAAYASGIISAADAMAIAYYRGFHAKRSHGTGGKRGGMVAAELSYEAALQFCEKVEWAGRLVLAASNSPSSITLS

GDLDAVQEAHAYFEKENIFSRLLRVDTAYHSHHMIPCAEPYLTSLKACNIQVSQARSDCIWISSVSGDVQSSSEEQGALT

GEYWVDNMVKPVLFSQAVQCSIWNSGPFESIVEIGPHFALKGPTTQVMEAVLESSPPYLSFMRRGHGIETFSDGLGRLWS

KLGPSSVDLTGYWKACSSSHIKFQMLKGLPAYAWDHDKVYWKEGRISRNHRLRKDVPHELLGRRTADDSDYELRWRNVLR

LTEIPWIRGHKFQGQVLFPAAGYVTMALEASKALVGDRHVRLFELRDICIRKALVLEEDQSSLETVFSVKRLNADFGIHD

ENMDLLEAEFSCYVCADETVGTLEKTVSGRIIIHLGSGVDVKLPPAAHFCTDLSPVDLDRFYSTVEELGLSYQGLFKGLD

HAERMLNHSHALAVWENHSMGAGSMVHPALLDVMFQAIFVASISPAAPSTLWTPYLPVSIDRIIVDPDHIPVYSHPEVRA

HIQAYVTKSSASSIVGDIHLLDSNGIHNGIQVEGLSLKSVAEPTEENDRSIFSQTVWDTDIASGTDFLRDRKEDAQESKL

IHAIERVALFHFRSLVEAITVEKAKTLAWHHQLFLKAVKANIETIRTGGNPVVRQEWLCDTRETIEDLRAQHPGQIDLNL

MHAVSEKLISIVCGETQILEVMLQDDMLNDFYMRGRGFETMNNCIARAVQQIAHKHPRAKYLEIGAGTGGTTHRILDTIE

SAYTSYEYTDISSAFFEKASHKFDKHASKMVFKVLDIEKDVVDQGFENGAYDVVIAANVLHATRTLSGTMGHIRSLLKPG

GYLIFMEVTGDQLRLLFLFGALPGWWLGAGEGRSLGPGVSTIAWDNILRNTGFSGVDDVFYDFPDRSRHTCSVMISQAVD

DQLRLLRDPLAATGMPISEQVLIIGGDTSSVSQLAYDTKRLISPWASCVAINNIDGLDSRRLPSRFSVICLTELDKPLFS

EIMSEQRLSNLQNLFATANVVFWITSGCNEGTPVANMMVGIGRALATELPHLTLQFLDVKTVERLKPSIVAQSFFRLVLA

KPLVMAEKSMLWTTEPELVFDGDDILIPRVLPDKEMNNRFNAARRPISENLWKESTCIELSNADNSSAPALFEIKNTIRP

GETTIDVKYSVCLGKRCTFVLGVVSGTSDTVLAISDTNASSVRISKEHVFFLPHDFSGNSATLLLDTANHVLAAKLLRCI

SPNSIALIYEPGVRLAAAIRHHAHENTVDVFPATSNREKCGEGWAFIHPHATERDIRTIIPRNTGCFINLSFKPPGALSR

ALLQQTIIHGPDCLSQIVSSADGFLLEAAFNWATTGLLSLDSVETVSVQSYVGTTRPSRDFPLVFDWTAPRLPVTVKPLE

PKGLFLPDKTYLMIGMTGDLGRSLCRWMAEHGARYVVLTSRNAEVDSAWIESMAAIGATVKVYKMDVSNRKSVLGVYTTI

KNSLPTIAGVCNAAMVLEDRLFANMTVGALSKVFEPKVEGSKVLDEIFHELNLDFFILFSSLTSILGNGGQSNYHAANLF

MTSMCAQRRARGLAASVMHIGMVADIGYVARSDRHIENHLRKLQYHPMSETDMHYLFAEAVMSSRADHPGNWNIVSGIET

FVDAPGVKLRPPHYHNPRFAHYVREENARKEDLRTDKTEKSVKELLEDAISEEDVTTVFQQAFLIKLERLTQLESHRIDA

NKSLLNLGVDSLSAVEIRNWFLQTVGVDIPVLKLLRGDTVSEISIDATKKYLAQRTS

>BGC0001276.1|BAA20102.2|MIBiG

MEVHGDEVLSVDSGISTPPSTGGGFRRPLETPGTEIGNLNLNPQNEVAVVGMACRLAGGNHSPEELWQSILNRKDASGEI

PSMRWEPYYRRDIRNPKILDQTTKRGYFLDHIENFDAAFFGVSPKEAEQMDPQQRLSLEVTWEALEDAGIPPQSLSGSET

AVFMGVNSDDYSKLLLEDIPNVEAWMGIGTAYCGVPNRISYHLNLMGPSTAVDAACASSLVAIHHGRQAILQGESEVAIV

GGVNALCGPGLTRVLDKAGATSTEGRCLSFDEDAKGYGRGEGAAVVILKRLSTAIRDGDHIRAIIKGSAVAQDGKTNGIM

APNAKAQELVAWNALRTAGVDPLTVGYVEAHATSTPLGDPTEVSAVSAVYGKGRPEGNPCFIGSVKPNVGHLEAGAGAVG

FIKAVMAVEKATFPPQTNLKRLNSRIDWDQAGVKVVQETLEWPGNEDDVRRAGVCSYGYGGTVSHAIIEEFAQQLQRPTT

NTTDEEPLPRILLLSAPQERRLALQARTQASWIAAEGRNRTLESIATTLSTRRGHHDYRAAIIAENHDDAVQKLSDIVNG

KAAEWTTSSRVLDASCSKDVVWVFSGHGAQWTAMATDLLKDIVFYQTISRLDPIVEREMGFSALHSLASGDFESSIKVQV

LTYLVQVGLAAILRSKGLEPQAVIGHSVGEIAASVAAGCLTAEEGALIVTRRANLYRRVMGAGAMVLVNIPFADMEKELQ

GRTDLVAAIDSSPSSCVVSGATEAVLALVEDLKSRGVNAFRVKTDIPFHHPMLDQLSEPLREAMEGSLSPRKPRVRLYST

SAEDPRSMVARDIHYWTSNMVNPVRLTAAVQAAVDDGLRLFLEVSSHPIVSHSVRETMLDLGVEDFTVTNTMARNKPADK

TILSSIAQLHCRGAVVNWKKQLPGPWALDVPLTTWDHKPFWRHIHTGPISASTLHDVDKHTLLGQRVPVAGETTMVFTTQ

MDDQTKPFPGSHPLHGSEIVPAAALVNTFLHATRATTLSNITLRVPVAISQPRDIQVVVSQNQIKICSRLTQKADSGADE

GSWLTHTTGQWEAGGSKNPPAQLDIAAIKARLANNKLADNFSIDYLDKVGVSAMGFPWAVTEHYGTLQEMIARVDVAPDV

PATSPLPWDAASWAPILDAATSVGSTLFFDQPRLRMPAHIHGVQVYTTQPPPKVGYLYVEKAGDRDLAVHVSVCDELGTV

LARFESMRFSEIEGTPGSNGSEESLVHQLAWPPATYSEKPLTINNVVLISRDRNVADLYCGSLKDRVSSITVLDAAADLL

SLSQDPSSVLQAKDTAVVYVPGPLHSADSIPTAAHSFLMELLLLVKIIVNGSLPTKVFVLTDRVCESESATALAQSPIHG

VSRIIASEHPDQWGGLIDVETPGQFPLETMKYVQEADNIRISDGIPRIARLRPLPRDKLLPPSKQTSLLPRPEGTYLITG

GLGALGLEVAQFLVEKGARRLILVSRRALPPRREWADILADPSSSLAPALETIQALETQGATVHTLAVDISSPDAAPQLA

VAIDALSLPPVRGVVHAAGVLDSQLVLSATSDSVERVLAPKITGALVLGTVFPPKALDFFMLFSSCGQILGFPGQASYAS

GNAFLDAFATSRRHQGDNAVAVQWTSWRSLGMAASTDFINAELASKGITDITRDEGFRAWMHISKYDIDQAAVLRSLAFE

ADEPLPTPILTDIAVRKAGSASSADAPSAAPKETNEMPESIPERRTWLDERIRDCVARVLQLGSSDEVDSKAALSDLGVD

SVMTVSLRGQLQKTLGVKVPPTLTWSCPTVSHLVGWFLEKMGN

>BGC0000155.1|ABB76806.1|MIBiG

MTSRQNTNTPMPLAIIGMSCRFPGKVASLEDFWDMLSNSKHGYRQFPRERFNWEAFYHPNQSRKDCIDVNCGYFLDGDIA

EFDAQFFKMNGTDAASFDPQGRMILECVYEALENAGVPKESIVGSKVGVFSTSNTSDYTLSLKDDIYSMPALVGVLGHAC

MLSNIVSNTFDLKGPSVSIDTACSSAFYALQLASQSLRSGETEMCIVSGCALNISPWRWTMLSNLTMLNPDGLSKSFDPQ

ADAGYVRGEGAASIIVKPLDAAIRDNDRVHCVLSDIGVNHNGRTNGYTLPDARMQASLMRELQVRLDIKPDEFGFVEAHA

PGTRVGDPIEISALQEVFSTSARTLEDPLLIGSVKANVGHLESSSGFPSLIKAAMMLKKGLVVPNANFENESMNSHLKEK

NMRVPISTQPWPKGKTYIAINNYGFGGSNSHCIVRAPPIPQGLVSQKETRNVESDYLFVLSANDEVALRRTREQLVEFLE

SVDASSTTMQNTAYTLGQRRSLLSWRATVVASNIDDLIIQAASPQVIPRRVTRQPTLVFAFTGQGAQYFGVGRELLQYPV

FSTTLKMASACAESFGANFSLQDELYGNEATSRINDADVSQPASTAIQIALVDLLRSWGIQPSAVVGHSSGEVAAAYAAG

LLSLPGAMRIAYARGQMAIRIKKVQPDFKGGMLAVAAGPADVLPLLDIVTSGKVVIACENSPKSVTVSGDEAGLVELESL

LEEDGLPHRRLAVDFPYHSTFLDPFIDDYEEAICTDDTFSNLQPTAEYFSAMAGRKVEPVTVQKPSYWASSAKFRVRFTS

AAKALLRSKPSPNVVVEIGPNPTLVGSLKSILSEIKKEIPHPIEVVPSLHRGQNARTAMLKLGASLVSFGQRIDMEQVNF

ASGHISGQPPTLVDGIKPYPWTRSHHWIKSRVRDDDLHRPFPHHDLLGSINSSWGSKELVWKNNLDVENVPWLRDYQVAS

SITYPLAGYVCAAIEASKQFAMTRNLFLDRAFKGFTVRDMIIDESLVMKEGIPVELVTKLRSLPGTNFEEFEVLSWDEGQ

RAWKRCCRALVKCEATTDGVEQVEEMKWAESRAACHSCVGSPLLYQRLSKVGPRRTGKFRNVVDLRYGAGKTTAEVVVSD

TKASMPQHYESDMTVHPTTIDGLFQCGSCIPFLDESSSVVGGSSNIWVPRSIKEFTIQTRPGEALKPEMVFRTVARVDKN

ERHDRSYSIDGTTDNAPICQIRIRGLKLAVEATLAPQWPAPHYGCYKIAWQNATELRSQAAQWHVLQGPGDVKNLAGSVS

KKIGGTVRPLCEGVPSEASFCVVVDVGEGLLASVERESFNHIKQALTTCEGVLWVTCGAFGVSYDSTHPNAGMVTGLLWT

IRSEMRASVASLDLDANASSDIEAQAALVKRVADHLAAAAQNADVQAEMEFTEKQGQLMVSRVVHDTQLDNVVHAVTGVI

APRTEPFDPEVRGFFTLQRPGMPDSLYLQRTDVPDPLDESEVEVRIAAIALDADDIHGLQGRALSGTVVRCGSTVTRVQP

GDRVFGLANIDGAVRTFARAPETCLARTPANIPIDAAAALPATLGAAYHALVDLGRLVAGESVLIVAVGSALGQAAIQVA

LAKGALVFALAHSQEERDAAIVAGASIDRVVTTLVGLPPIQILFNPVSDANANLSMLGALAPLGRIVQVGEPSHQYPALA

VGHSFSIAHLDAVADALPAQMAAILDAVVGLVDSKFVHSPPVRTVGLEYLSEALSNISETDSKKLLLVPGKNEMVKATPS

CPAPPTFDPAAVYLLVGGSGGLGRVIAKWMLNNGARKIGLLSRSTSMSPDVRTLVDDAAGIGAEVFLLPCDVTSQHHLQR

VIDQCVIEKGQIKGVINAAMVFKGGVFTSVSFDDFTSVVQPKVCGTWNLHHALREATLDFFILISSVAGIMGTPGHSAYA

SANTFLDSFAMYRMQQGLPATSLALTAVVDAGYMAENASKLQKLKYVSEFEGEILLTADVLALLGAAVTGSIASSCKGFS

IIGAGFGTALKLPSYAQDPRFSTLTSNHSQDRKSKPRTTTAANTDTLVYAVDQADTKEEATQLLLAAIRDKIAQLQLIPV

SDIVDDQTITELGLDSLTVMELYSWVGRLFRLRFGIQEYARLDTLEKIVDSVIVKREAAKVEAP

>BGC0000155.1|AAB08104.3|MIBiG

MTVRDSKTGGITPIAVVGMSFRGPGDATNVEKLLNMISEGRESRAEVQAKKWDPEGFYHPDSSRHGTHNVEYGHWFQQDV

YNFDAPFFNVSPAEAAALDPQQRMLLECSYEAFENSGTPMSKIVGTDTSVFVSSFATDYTDMLWRDPESVPMYQCTNSGF

SRSNLANRISYSFDLKGPSVLVDTACSGGLTALHLACQSLLVGDVRQALAAGSSLILGPEMMVTMSMMKFLSPDGRCYAF

DERANGYARGEGVAVLLLKRLEDALADNDTIRAVIRGTGCNQDGKTPGITMPNSVSQEALIRSVYKKAALDPLDTTYVEC

HGTGTQAGDTTEASALSKVFSPGRRLPLLIGSVKTNIGHLEGASGLAGVVKSILMLEQGVILPNRNFERPNTKIPLEKWN

LRVPTTLECWNNVKTRRVSINSFGYGGANVHAILESATDFLRDNSMGTDSTRFASRRSVVVGNVGQTKPAVSLVQDMSSN

DRSHEDPTPLLFALSAFDSSAGDAWARSLSIYLSQRQGSDEKTILSSLAYTLSDRRTWHPWKAALSATTIQELITKLEKV

RFVNMAPRHNIGFVFTGQGAQWCGMGRELISIFPRFRQSLIACDIALQSFGADFHVIDELEADVESSRINKALYSQPLCT

ALQIALVDLLVSWGIYAQSVTGHSSGEIAAAYAAGALSLSDAMLVAYARGCATANLAKKGAKGAMAAVSMETQELSHILS

ALENGKVGIACFNSPTSCTVSGDKSALDELQDVLRQKGVYNRRLIVDVAYHSHHMELIADSYRSAISSIQPLPGSDVKFF

SSVTGELLDKNKLGVDYWVSNLVGQVKFAQSLSSLVSSHHGTGTPQIQALIEIGPHAALGGPISQVIDSEPLANPTGYFS

ALVRKKNAVTTILSLAADLFLSGYPIQLSAVNQNCNSRHTPLVDLPSYSWNHSKAYTAESRISKTYRQRRYPRLDLIGVF

DVHSSVLEPRWRQVIRLSELPWLQDHKIQSSILYPVAGYIAMAIEAATQRNQMREMGNDILGYQFRDVAISSALTIPDMP

GQVEVFITLRSFSESVRSPSNLWDEFSISSVNDENRWTEHCRGLISVLKSSKLSNLVNGKMQDASTIACQHDLREVFATC

CKTEWDVKDMYEHFWETGMQYGPTFANLCDVRCTSNKCIGKVKVPDTAAVMPMKHEAPFIIHPGTLDSIIQTYLPALVQA

GHLKSATIPVAIESMFISRNVTRQAGDLLTSYASSTRKDYRYFSTSMSVFADGPSSENQLVITIDDMTLVALDRPNSSEE

SGEALPLAFNLHWKPDVDMLTEEQLVEMINASTKVKDHIAAKKMKQTAAQLGKEILARVPFEQAQVVGESSRHLWKLLHA

SLESLSTPDHRGALDEISSLKNVDSTLAQAADRLSNVLTGRVAPSDVASMYDLMEAVRIPELYDNNLPTATYLHLLGHKK

PSLRVLTVGPQSGPTSLNLLMLLAELGGGEIPFAVLHHSDAELNIDQTVRSRFPSWADSVGFRDVFNESGASQQNPPIVN

ETYDIVVAFNVLGSSPGFSKTLSAAAPLLNARGKILLVDNSHKSPMAALVWGPLPSFLSTWVDEKSADSPDVDCAVQSMG

YDIYARLCPNVTVIQRAAQVQKAEKTIGLDVLVVTDGEPAGVDLQQLQTLCEDQYAEVHVASLEHARPRPGQACIVLSEL

SRPVLAAPTAAEWEAVKRITDTCSGIVWVTRGAADNVCSNPQVSLIQGFARTVRAEAGDKPITTLDLDNDKVLSAQAAAA

YIAAVFQRMMQGGEDIDVELQERRGILHVARLIEDGDAAKQLQGEATAMELRLDQAGPCRLFAGTPGLLDSLHFTVDDRV

QESLETGQIEVQVHATGINFKDVMMAMGQIAVEDLGCECSGVVSAVGDGVVGLRVGDRVACMGPGSFCTQLRVDARLAHR

IPHHMELETAAALPITYVTAYHSIHNIAHLRHGETILIHAAAGGLGQALVELSQLVGARVLVTVGSTEKKRLIMQRFRLS

EEDILFSRDTSFVHDVMRLTNGRGVDVIMNSLAGESLRQSWTCIAPNGRFVELGQRDITVNSRLDMAPFARNVSFTAYNL

AYMLRHDPQAAHEVLAEVLALYDQGKLRGPEPLEKCTFSQLGNAFRKIQTGRHMGKMVAVANPDDMVWYKPPPASRRTLF

RPDASYLLVGGVGGLGSATALWMSTRGARHLLLLNRSGADTEAARTTLATLRANGCTATVLACDVADKAQLSSVLAEARS

NWPPIRGVIQGAMVLRDSMLANMTLEDYMAVVRPKVQGTWNLQTHLPADLDFFILESSISGIIGNPGQAAYAAANTFLDA

FARWRRARCQPATVIDIGAVHGIGYLERNVDVKLSMERQGFAFTDEQLLMRLLEFAISHSSREPHRAQIVTGLGPWHPDT

SLPGLNAPLFSRYRMLSCQNSTGSTDVDTLRGILAQSSSFDSAVTIVLSALVDQVVSRTEIPIENVHTTKSLQDYGIDSL

VAVELRNWLIKDMDSVVPMLELLGAESLSALAVKIAARSQLISTNNRG

>BGC0000022.1|EAA65604.1|MIBiG

MGSTSSEPTYDSEPIAIIGLSCKFAGSADSPEKLWEMLAEGRNAWSEIPESRFNHKAVYHPDSEKLGTTLDPQFRFQLES

VYEALENESTAGLTIPSIAGTNTSVYAGVFTHDYHEGLIRDEDKLPRFLPIGTLSAMSSNRISHFFDLKGASVTVDTGCS

TALVALHQAVLGLRTREADMSIVSGCNIMLSPDMFKVFSSLGMLSPDGKSYAFDSRANGYGRGEGVATIIVKRLADALRD

GDPVRGVIRESYLNQDGKTETITSPSQEAQEALIKECYRRAGLSPSDTQYFEAHGTGTPTGDPIEARSIASVFGKNREQP

LRIGSVKTNIGHTEAASGLAGLIKVVLAMEKGFIPPSVNFEKPNPKLKLDEWRLKVADTLEKWPAPAERPWRASVNNFGY

GGTNSHVIVEGVPKRLYTPANGNETGQIKHETESKVLLFSGRDEQACQRMVASTKEYLKKRREQDPPMTPEQVKTLMQNL

AWTLTQHRTRFSWVSAHAVKYSTSLDTVIDALESPPPASRPVRIPDSPFRIGMVFTGQGAQWHAMGRELIAAYPVFKATL

DEAEQYLRQLGAGWSLIEELMKDAATTRVNDTGLSIPICVAVQIALVRLLKAWGITASAVTSHSSGEIAAAYTVGALSLR

QAMAAAYYRAAMAADKTLKSAEGPQGAMVAVGVDKAAAQAYLDRVEKSAGRAVVACINSPSSITIAGDEAAVVAVEKLAT

EEGVFARRLRVETGYHSHHMEPIASPYREALRAALAQEDAESGTKDQTDVPGFADATKPGSLDHTVFSSPVTGGRVTDAK

VLSDPEHWVRSLLQPVRFVEAFTDMVLGSTDSSNIDLILEVGPHTALGGPIKEILALPDFSSRNVSLPYMGCLVRKEDAR

DCMLTAALNLFSKGHSIDLLRLSFSSGIPELQVLTDLPSYPWNHSIRHWSESRRNAAYRKRSQEPHELLGVLEPGTNPDA

ASWRHIIKLSEAPWLRDHVVQGNILYPGAGFVCLAIEAIKMQSAMSGTNDVTGFRLRDVEIHQALVIADSADGVEVQTTL

RSVGGKVIGARGWKQFEIWSVSADSEWTEHARGLITVDTETKASTLVASTLDESGYTRRIDPQDMFASLRAKGLNHGPMF

QNTLRILQDGRAKEPQCVVDIKIADVSSSKDSGRMSLLHPTTLDSIVLSSYAAVPSSDPSNDDSARVPRSIRSLWVSSMI

SSAPGHTFTCNVKMPHHDAQSYEANVTVVDEAGARAESMVEMQGLVCQSLGRSAPAEDREPWTKELCANVEWAPDLSLSL

GLPGSSDAIDRRLNTLRDQNPDERSIEVQTVLRRVCVYFSHDALSSLTENDVANLAFHHVKFYKWMQDTVNLALARRWSA

DSDTWIHDSPAVREKYISLAGSQTVDGELICQLGPLLLPVLRGERAPLEVMMEGRLLYKYYANAYRLEPAFEQLKSLLGA

ILHKNPRARVLEIGAGTGAATRHALKTLGTDEDGGPRCESWHFTDISSGFFEAARAEFATWGGLLEFNKLDIEQDPEAQG

FKLGSYDVVVACQVLHATKSMHRTMTNVRSLMKPGGTLLLMETTQDQIDLQFIFGLLPGWWLSEEPERHASPSLSIDMWD

RVLKGAGFTGVEIDLRDVNVDAESDLYGISNIMSTAVGTAGSSPEKVDAAQVVIVTGNKTGFQDDWVRGLQAAIAQDSGS

DALPEIISLESPSLGAEAFQSRLVVFVGELDRPVLASLDSTELEGIKTMALACKGLLWVTRGGAVECTDPDSALASGFVR

VLRTEYLGRRFLTLDLDPAAHSPASDISVIVHLLSSRLQPAVETAAPADSEFALRDGLLLVPRLYKDVVWNALLEPEVPD

WASPEKPRAYGLNFRDVMVAMGQLKERVMGLECAGVITRVGAEAAAQGFAVGDRVMALLLGPFSSRARVSWHGVASMPAG

MGFADAASIPMIFTTAYVALVQAARLSQGQTVLIHAAAGGVGQAAVILAKEYLGAEVFATVGSQEKRDLLIKEYGIPDDH

IFNSRDSSFAPAALAATAGRGVDCLIEVLAPFGHFVEIGKRDLEQNSLLEMATFTRAVSFTSLDMMTLLRQRGDEAHRVL

SELARLAGQGIVKPVHPVSVYPMRQVDKAFRLLQTGKHLGKLVLSTEPDEEVRVLPRPATPKLRADASYLLVGGVGGLGR

SLASWMVEHGAKHLILLSRSAGKQDSSAFVNGLRDAGCRVAAISCDVADRADLDRAIAAASELGFPHVRGVIQGAMVLQD

SIIEQMSIADWNAAIKPKVAGTRNLHDRFSQRNSLDFFVMLSSLSAILGWASQASYAAGGTYQDALARWRCSKGLPAVSL

DMGVIKDVGYVAESRSVSDRLRKVGQSLRLSEESILQTLATAVLHPFGRPQLLLGLNSGPGSHWDPSSDSQMGRDARFAP

LRYRKPASTKSAQTSSSGDGEEPLSSKLKSADSPDAAANYVGGAIATKLADIFMVPVADIDLTKPPSAYGVDSLVAVELR

NMLVLQAACDVSIFSILQSVSLAALAGMVVEKSAHFEGSATGTVVVA

>BGC0001436.1|ASK38717.1|MIBiG

MVENVSSPSSPRTSSPSGSCTPTSATSVGSDDKSMPIAVVGMSFRGPRDAISVESLWRMISEGREGWSKIPKSRWNNDAF

YHPDHSRHGTINVEGGHFLEEDLARFDAPFFNMTNAEAAALDPQQRLLLESTFEAVENAGIPLDKMLGSKTSCFVGSFCG

DYTDMLVRDPEAIPMYQCTNAGQSRAITANRVSYFFDLRGPSVTVDTACSGSLVALHLACQSLRTGDAKMAIVSGVNTIL

SHEFMSTMSMMRFLSPDGRCYTFDERANGYARGEGVGCLLLKPLSDALRDNDTIRAVIRGTGSNQDGKTSGITLPNANAQ

QELIRDVYAAAGLDPLETEYVECHGTGTQAGDPLETGAVAKVFSPGRPDDRPLRIGSIKTNVGHLEGASGIAGVIKAVLT

LENQCFLPNRNFKSINPRIPLKEWKLKIQLENERWETVGPHRVSVNSFGYGGSNAHAVLEDTKGYLEQRSLTGSFRRVRA

LPHAATDLEPVSDPGSGPERTRLFVLSSFDQASGQQQIDQLREYLEQNSSRIDDQYLADLAYTLGERRSPFLWKTAMPAS

SVSSLVEGLKTRAKVSRAEKKKPTLGFIFTGQGAQWCGMGRELLAAYPVFASSVDAIATYLKSLGAPFDVREELVRDPKD

SKINQPLYSQPICTAVQIALVDLLTFWGIRPASVTGHSSGELAGAYTAGALSMEHSMAAAYYRGVASSDLPRDHTQRGAM

MAVGASKDAIQPRLSSLTTGTAVVACVNSPSSVTISGDASAVDELHGLLEKDQVFARKLAVDVAYHSHHMKAVADQYRTA

MAAAGVTAVQPESTEPEVEFFSSVTGEKASLTDLGIEYWVANLLSQVKFADSVHRLCMETSASGRARKTKTKAPKRSGAN

NKAKVDMLVEIGPHSTLAGPIRQILGADQTLEQASIRYASALLRKSSAVDTTLTLASTLLMAGYPIDMAAINRPSDHHRV

GVLVDLPPYPWNHSGSYWAEPRLSKAYRNRAHPRNDLLGVLDTHSSPREPRWRNYLRTSEIPWIKDHMIQSNVVYPAAGY

LTMAVEAIGQRIGDNFPGHRISGYRLRDVAIEAALVISDDSEPEVMLSLRPSGDSGLVPAERWHEFHVLSVTPDNRWTEH

CRGLICAEVAAMDGDEDDRGAEAGLTAETERWIEEAEQLCQKDVDIPRFYAELTGLGLEYGETFANMTRARSASHVCLAE

IEVADTAAVMPLGFQSPFVVHPSTLDSLFHPLFVALSSDESLQDPAVPVAIEEIWIRHGMAKEAGHKFQVCASTQETGRD

RIQAAISVVDAQRARSGPALTVRGLTCQFLDRASGDVEGDEQPTRLAYELHWEADVDLLSSSDLATLCAVGRPRDVGEKV

ARYVKLLGHKNPHLAILEVGAGQGELCIPVFRALAGEANSTPSFQSYTLADTEPGLSETIATIADQFDERADLIQYKELD

ISSDPLQQGFNAHSLDLILLPSRGVSATLRSKILKHAHQLLTPEGRLIVVDTRDLQEWWQALRESNFTDPEVIHDSPSET

EADISVLVSKPQPQPRDQTPSDPPDVLVIAENQDSGVSIEHLQRLLADAHVPATVTDFAHADPEGKTCIVLSELTTSLLS

HPDQHSFETLKRILVAGGRGVLWVVRGATGPAPTSSLATGLLRTIRSETDDDRPIVSLDLDASHPLSAESAAQSIFSAFR

HRFVSPGGSHEVEYAERDGILRIPRVVESSLVNHEIVSSLRPAVAEDQPFFQPGRPLELTVGTPGRLDSLYYVDRSCISE

LPSDYVEIEVKAIGLGNGDVKTALGHDDAATRLGAECSGVVTALGDAVSGFKIGDRVAGFGAGTVATLYRDQAARFQLIP

DDMSFARAAALPVAYITAFFAVHALGQVSRGDRVLIQDAGTAAGQALLELCALAGGDIIAVVDSPSQRAFLVGEYDLPAS

RILVGLRGRRLATSVMTLTRGCGVDAIFNFRGGEERRLCWSCVAPYGRFIDLGGGPSDLTDMPQLEMASFFSKNASFTAL

DFHYLVTQKPQAVHRIWSDVMALVRAKAIRGPPRLQLHSVSEVETALKQSQDGCDVEKVVIRAERDTIVQAIPPPKGDLL

RADASYVLVGGLGGIGRAMASWMIANGARHLIFVNRSGLARNEARETVESLEGHGAHVAVYSCDVSDRDQVAQMVAQSSK

EMPPIRGVIQAAMILRDMLFEKMSVDDFNTVLQPKWQGTWNLHGLLPRDMDFFIMLSSISGVIGNATQAAYAAGSTFLGA

FAQYRSSLGLPAVTLDLGVITGIGYLSEHEELLQGMQRQGFEGTNEQTLMALIRSAIVSPRRTGSQAEIVTGLGTWREGV

SLGNFDQPLFAHFRRQALGLRDATAEGPGTSVRESLRGCKTLDDAVALVCAALIDRLASRLNTPVDNIDSQRAMSEYGVD

SLVAVEMRNWIGKEMESTMPILELLANQSISQLSEKIAQRSKVVAVSGSEE

>BGC0001245|AHV78245.1|MIBiG

MPSTSNPVQTPIAIVGLACRFPGDATSPSRFWDLLKNGNDAYSPTTDRYNASTFYHPASKDRQNVLPTKGGHFLTQDPYA

FDAAFFSITAAEAAAMDPKQRIALEVVYEAFENAGQPLQRVAGTQTACYMGSSMSDYRDGVVRDFGHMPKYHILGTSEEM

VSNRVSHFFDLHGPSATVQTACSSSLVATHLGCQSLRSGESEMAVVGGVGMILSPDGNFQLANLGFLNPEGHSRAFDEDA

GGYARGEGCGILVMKRLDDALRDGDSIRAVIRGSGVNSDGWTQGVTMPSSEAQAALIKYVYESNGLDYWSTQYVEAHGTG

TKVGDPAEVGAIHRTIGQGATKSRKLWVGSVKPNIGHLEAAAGVAGIIKGVLAMEHGLIPPNIYLSKPNPAIPFDDWNMA

VPTKLTPWPITQGVRRMSVSGFGMGGTNGHVVLESFKPGHNYANGSLTNGTANGVKTNGVKTNGALTKRTGFHSGKRLFV

LSSQDQAGFKRVGNALAEHLDSLGPAASTPEYLANIAHTLAVSRSGLAWRASFLAESAAELREKLSTEPGENAVRAANSQ

ARIGFVFTGQGAQWARMGIELLERPVFRDSVAKSADILKSLGCDWDPVTELSRGKDESRLGIPAISQPICSVLQIALVDE

LSAWGVVPSKVVGHSSGEIAAAYTMGALSHHDAIAAAYFRGNASAGLKHLKGGMMAVGASPEEARQLILEAALKNGSVSI

ACVNSPSSVTISGDVAALEELRAILEERGVFARRLKVDVAYHSSHMNSAVGKYYASIADIEPAQPSDGHPIMVSSVTNSE

VDAELLGPSYWVRNLISPVLFADAVKELVRPSSSSGDDGEENTVDLLVEVGPHSALGGPVEQILSHHGIKNVAYSSALTR

GANALDSVLKLAGDLFLQGASFSLQTANGDSQSRLLTDLPPYPWNHTKTFRADSRLHRELTTQPFAPRSLLGAPLPTMAE

NEHQWRSFIRLADEPWLRGHTVGSTVLFPGAGIISVILEAARQALVDPGKTARAIRLRDVNLFAAMALPEDQPTEVIVHM

RPHLIATTGSTPASWMEWTVSSCVGTDAQLRDNARGLVAIDYEEHRSAQMAGEDALADAARVAEYHRVRDECAEVYGKER

FYEQFAKAAWNYGELFQGVELCRPGPGKTVYDVKLVDVGETYSRGQLDRPFLINAASLDAIFQGSLGATYKGNGVFEYDK

PHVPTTIGELEISADIPGDASYVLPSVCRGERYGFNELSSDIAVFDKDVSKVFLSVKDFRTSELDMEVGQGDGDGVEADP

ADITSEVKWNYALGLLRTDEISRVLSGFPTQARLAELIRLALHENPASTVIELISEAADYGNAAMSKLSESDIRPDRIRY

SVANPGQSDGLAFGQIVSLGENNSPVPADKAPSDLLIVSHEPSDPSSLEKLIDLARQQLAKPDATIVVTASSDAAASALV

GKGFQIVSGVEDGNFIALYSSREAQKGPIANGSPAHEVVIIQPSTATSAAEKFASVLQETLQNQGDAVSISVWSNSIRAE

DVKGKTYISLMELERPLLEDLSEPDFETVRTVGLNCERLLWITCGGNPAFGMVDGLARTMMSENASIKFQILHLSEPTGH

QYGPSLATRVLASSTGDNEFQEVGGILQIARFFKSHQQNESIRHHLEASVRNENLNDKGEALRLTIGNPGLLDTLRFVPD

ERMDPPLEDHEVEVEVKAAGINFKDVMACMGLIPVEALGLEASGVVVRMGSKVTDFKPGDRVSTVHIGAYATRIRVHHHG

LAKIPDSMSFEDAAASPIVHTTAYYAFICAAKLRKGQSVLIHAAAGGVGQAAIQLAKHLGLVIYVTVGTEDKRRLIMEEY

GIPEEHIFHSRDASFVKGIKRITGGRGVDCVLNSLSGELLRASWGCLASFGHFIEIGLRDITNNMRLDMRPFGKSTSFTF

VNNHTLFLEDPETFYHVFHDCFALIQQNVLRAPAPVVVYPVGQVGDAFRTMQQGKHRGKLVLSFAGSDDGKVEAPVLRKA

EDSLKLDPDATYLFVGGLGGLGRSLAKAFVASGARNIAFLSRSGDTTPQAAAVVAELCASHGGGVRVKAYRGDVSDQSSF

LAAMSQCSQDLPPVKGVIQMAMVLRDVVFEKMSHADWTVPLRPKVQGTWNLHTYFSSTSATRPPLDFLILCSSISGIHGN

PGQAQYAAGNTYQDALAHHSRRRSGAPVVAVNLGIMRDVGVLAETGTTGNFALWEHALGIREPAFHALMKSLINQQLRGT

CPPQVTTGLGTADIMAAHGLARPEYFADPRFGPLAVASVAAGAAAGEGQGGGESVAAKLGKVKGKDEAGEIITEALVQKT

ADILQMPASEVNPSRPLYRYGVDSLVALEVRNWISRELKANMALLEILAAVPIESLAGKIAEKSKLVAGG

>BGC0001405.1|EAQ86385.1|MIBiG

MVEGANQEWSKDQEPIAIIGLSCKFAGDAANPQGLWKMLAEGRDAWSEIPTSRFDPKGVYHPDAQKLSTTHVKGAHFIEE

DIGLFDAAFFNYSAETASALDPQYRIQLESAYEALENAGLSMSQVAGTNTSVYAGIYTHDYHDGLMRDEEKLPRFLLIGT

MAAMASNRISHFFDLRGPSMTIDTGCSTALVALHQAVLGLRAREADMSIVGGCNLMLAADMFKMFSSLGLVGPDGRSYSF

DSRANGYGRGEGVGTVVIKRLSDALAAGDPIRAVIRDTYLNQDGKTETITSPSQAAQEALIRECYRRAGLSPQGTQYFEA

HGTGTPAGDPIEARAIAAVLGGGRTEKLRIGSVKTNIGHTEAASGLAGLIKVVLALEKGQIPPSVNFKEPNPQLKLDEWG

LKVATELEPWPATNGEPRRASVNNFGYGGTNSHVILEDAGRWAPQPLIKAPPSTQAEAQSDVLVFYGRDEQACQRIVANT

QEYLRNRKLENPAMSAGEILSLIKSLAWTLSMRRSRFPWVSAHVATYSDDLDQVIRSLDAPQFKPVRIPSRAPRIGMVFT

GQGAQWYAMGRRLIHVFPIFRSTIYTAQHFLREFGADWSLIEELNRDAETSRVNTTALSIPVCVAVQIALVRLLRFLGIS

PSAVTSHSSGEIAAAYTVGALTLRQAMAAAYYRSAMAADKTLMNASGGGPKGAMVAVGVGSEAAQEFLDRVASTNGKAVV

ACINSPSSVTIAGDEAAVQQVEDLAKGDGVFARRLRVDTGYHSHHMMPIAEPYRQALRQALSSNNQTSHNETDPATAGIP

EGAPWAKQYLAALSAALEAEAQTLRDEGPIAFSSPVTGGRITELSQLADPEHWVGSLLQPVQFVDAFTDMVLGDMDESGT

SVDVVLEVGPHTALGGPIKEILAEAEFEGVDVAYMGCLVRNEDSHDNLRAAALNLLRRGAPVHLEQLAFPWGRWPFLGES

GYTRRVDPEDLFAELRMQGVHHGPMFQNTTSIVQDGRSKEPRCVTTIEVADVGTKHVVHPTTLDSVIISSYAALSGSGAS

NDSAMLPQSIPEALGLEPHGHDGPATPFMCNTKAPRQDAQGSEAHISVVDGAEAVIEVQGFVCQSLGQSAAVDAEEKQPL

DQGAVRQGRLGARRCSVPGSARRDIRREEKVLTEQDVANLAPHHVKFHTWMKDTLDAAAARRLGADSHTWTSDDPQKRQQ

YTTLAATKSVDGELMCRLGPHFVPVMRGEKAPLEIMMEDQLLYKYYANALRFRPGFVQFAALLRAVAHKNPHPDKEPGPL

YDTWHFTDVSSAFFEAARSEFGAWSNFMEFDQLDIEKSPESQGFNLASYDIVVACQVLHATKSMARTMDHVHKLMKPGAT

LLLMETTHDQLDLGFIFGLLPGWWLSEEPERHSSPSLSIPMWDRVLKGAGFGGIDVEIHDYENDDDMYAISSMLATASPR

QFKLASDSVIVVTSDKAPPPSNWLQSLRESIAKVTGGALPAVQPLEAPSATAETYNTKICVFVGEIEQSILHTLDSASLR

SVKTMATACKGLLWVTRGAAVECVDPNKALVTGFLRSLRSEYVGRYFPTLDLDPQTPVWSESGIAAIAHVLQTSLGTADD

VYSIMAEPAPVDNEFAVRDGLILVPRLRKDLARNKMLTPEAPDWTMPESIPEAPLFQEQRPLRLKAGVPGLLDTLVFADD

DEDISGDADTVEIEPRAYGLNFRDVMVAMGQLRERVMGLECAGIITRVGSEAAAQGFAVGDRVMALLIGPFANRARVSWH

GVAHMPEGMTFDDAASLPMIFSTAYVGLVDIANLRPGQSVLIHAAAGGVGQAAIMLAKDYLGAEVYATVGSQEKRDLLTR

EYGIPAERIFNSRDASFAPAVLAATGGRGVDVVLNSLAGPLLQAGFDVLAPFGHFVEIGKRDLEGNSLLEMATFSQVASF

TSLDMMSLLRERGTHAHRVLSEVARLAGRKVIEPIHPVTVFPMQQVSRAFRLLQTGKHTGKVVLSVAPDEQVRVLPRAPA

PKLRPDASYLLVGGVGGLGRSIAHWMMDHGARNLILLSRNASKQNAGTFITQLREAGCRVVAISCDVASEQSLTRALDRC

KNEEGLPPIRGVIQGAMVLKDSILEQMTLDDWQTAIRPKVAGSWNLHSNFTQRGSLDFFVMLSSLSCILGLASQANYAAG

GSYQDALARWRQASGLPAVSIDIGIVRGVGYVAESRAVSERVRKPGQMLVLPEDSVLRAIGAAVLHPLEQPQVLLGLNSG

PGPQWDPTSESQMGRDARFVPLKYRRPTGAQAAGQQLGDADVKPLSAQLQEAGSRDEATRLVGDAIASKLGDIFMIPIDD

IDLAKSPALHGVDSLVAVELRNMLMLQAAADISIFSIMQSASLGALASDVVAKSSHVEIAAGA

>BGC0001246|AHV78252.1|MIBiG

MSTHNSTPFGGDTSIAIIGLSCRFPGDASSPSKFWDMLKNGRSGFSPSTTRYNADAFQHPMGGGNRQNVIPTLGGYFLKE

DPYVFDAAFFNITAAEAAALDPRQRISLEVAYEALENAGLPLQKIAGSQTACYMGSSMSDYRDGVARDFAHAPKYHILGI

SDEMIANRISHFLDIHGPSATVQTACSSSLVATHLACQSLRSGESDMALAGGVGMIMSPDSTMHLNNLGFLSPNGQSRSF

DNSADGYGRGEGCGILVLKRLQDAVADGDCIRAVIRASGANSDGWTQGVTMPSQKAQADLIKRVYETHGLDPGATQYVEA

HGTGTKAGDPIEAGAIYSTIGHPGSSSSPSRKKLWMGSVKPNIGHLEAAAGVASIIKGVLALEHGLIPPTINFSEPNPSI

PLADWDMAIPTKLTPWPVVQRKRMSISGFGMGGTNAHLVLEAPERSHRSSSPTTKVTPTDDVNLKKLFVFSSQDKAGFER

LGRSLVDHIDSLGPVASRSDFLSNLLHTLGVARAGLRWKSTCLAQSVTELREQLLGGVLAENAARTPGAPPRIGYVFTGQ

GAQWARMGVELSQKPVFRESVARSAGYLKSMGCDWDPMTELLKDQETSRLSRPDISQPICTVLQIALVDELHAMGITPSK

VTGHSSGEIAAAYTIGALSHHDAVAVAYLRGQASAGLKHRTGGMMAVGCSREQTRKFLEELGVKQHVTVACVNSPSSVTL

SGDVKPLEELRVKLDERGIFARRLKVDVAYHSSHMHACSTSYFTALENIEARQLRSLGPENETPPPIMVSSVTGKEVDPE

LLGPYYWVQNLISPVLFTDAIKELVLPIDRQTKVVDVLVEIGPHSALAGPIEQTLAEHNIQNVTYASVLTRGQSAVTTRL

DLAAELFRLGVALNVDKVNGDSGARLLVDLPPYPWNHSEQFRADSRLQREFVAQAHPTRSLLGASLPSLDENERIWRGFI

NLNDEPWLRDHTVGTTVLFPGAGMVSLVLEAAQQLAEPNQTPHSFRMRDVAFMAAMSLPEDTATEVIVHMRPHHAATSGS

TPATWWEFSLSSCSGPSAKLRNNCRGLLAVVYRETLSPYMLKEEEASVSARVADYHTILREYPETCSSDAFYDRMSRCAL

PYGSAFRGVENCHPGYGKTAYDVKVLDIGETFTQGKLERPFFIHAGTLDSILQGWLGTTRSKDGNGDFGLPKTLLPTAIG

ELEIAASLPARPGYVMRSMCSSRQHSFSEFSANISTFDEDLSRVLLSITDFRTTEVDVDEGGVADVAEVPASDLHTADLI

SKIHWNYAMDLMTPVEICERVEAAGATTADEKLTELVRLALHQKPSSILIELVDDVSGLSSSLMSSILSGEVIRPAQVLY

GLLHGDLDGSPNGFVLGEQGDPLPSGIGLADVIVIPSGIETKLEGIIDRVLRLSKPEVRILIAGSQDTHPESTATIFKSR

SLACLAQVATGLEWAALYQSDTSGKQTNFTNGTHSEDFIILEPNDLSTNGREFCTNMQESLRDQGHTAVVSSLSHESQRD

RLEGSTYISLLELERPLLRDLSESEYVSLRALLVGCERLLWVTSGDDPSLSIMDGLARTVNSEVSGAKVLTLNLSGENNP

RTGPPLAARILNASAGDASGDREFRERCGMLQVARIQSCPETNSSLRAHLEDRVLPRPLDQAGSPGGENTSFHLTIGKPG

MLDSLRFVPSETMLLEPLGENEVELEIKATGINFRDIMASMGLVPVKGLGQEASGVVLRAGSRASQIFSQGDRVTAVSTG

GTHATRARCEYRVTTKIPDDMSFEEAASLPMVYSTAYHALVKLAKLRRGQSVLIHAAAGGVGQAAVQLAKHLGLVIYVTV

GTNDKRQFILDQYGIPQEHIFSSRDSSFVRGIRRITEGRGVDCVLNSLSGELLRVSWECVAMFGTFVEIGLRDITDNMRL

DMRPFVNSTTFSFFNIQTLIDFAPSTLGETLQEVFQLLHKGELRSPFPLSVYPVSQVEEVFRSMQQGKHRGKLVLSFGDV

KVLAPVLTKAKDSLRIPSDVTCLLVGGLGGLGRSLALDLIAAGCRHIAFLSRSGDAKPESKAVVKDLAAQGALVKVYRGD

VADETSFLSAMKACSEELPPIKGVVQMAMVLRDAVFEKMSYEEWTASLRPKVQGSWNLHKYFDQRRPLDFMIFCSSISGL

FGNPGQAQYAAGNTYQDALARFRREQGLRAVSVNLGIMLDVGVIAETGIHNFKPWEQFLGIREPAFRALMRSLILEQHQQ

NSPIGNAQICTGLGTGDMIAKHNAPTPPWLEDPRFSPLAVRSAQSMAASGNKEDGAGSMSIAAKLAEAGGSKDPAAAATA

IIADALVNKMADILRIPPSEVDPSQAMYHYGVDSLVALEVRNWITRELKASVALLEILAAVPIERFAAQIASKSKLLSVN

>BGC0001557.1|ASK38717.1|MIBiG

MVENVSSPSSPRTSSPSGSCTPTSATSVGSDDKSMPIAVVGMSFRGPRDAISVESLWRMISEGREGWSKIPKSRWNNDAF

YHPDHSRHGTINVEGGHFLEEDLARFDAPFFNMTNAEAAALDPQQRLLLESTFEAVENAGIPLDKMLGSKTSCFVGSFCG

DYTDMLVRDPEAIPMYQCTNAGQSRAITANRVSYFFDLRGPSVTVDTACSGSLVALHLACQSLRTGDAKMAIVSGVNTIL

SHEFMSTMSMMRFLSPDGRCYTFDERANGYARGEGVGCLLLKPLSDALRDNDTIRAVIRGTGSNQDGKTSGITLPNANAQ

QELIRDVYAAAGLDPLETEYVECHGTGTQAGDPLETGAVAKVFSPGRPDDRPLRIGSIKTNVGHLEGASGIAGVIKAVLT

LENQCFLPNRNFKSINPRIPLKEWKLKIQLENERWETVGPHRVSVNSFGYGGSNAHAVLEDTKGYLEQRSLTGSFRRVRA

LPHAATDLEPVSDPGSGPERTRLFVLSSFDQASGQQQIDQLREYLEQNSSRIDDQYLADLAYTLGERRSPFLWKTAMPAS

SVSSLVEGLKTRAKVSRAEKKKPTLGFIFTGQGAQWCGMGRELLAAYPVFASSVDAIATYLKSLGAPFDVREELVRDPKD

SKINQPLYSQPICTAVQIALVDLLTFWGIRPASVTGHSSGELAGAYTAGALSMEHSMAAAYYRGVASSDLPRDHTQRGAM

MAVGASKDAIQPRLSSLTTGTAVVACVNSPSSVTISGDASAVDELHGLLEKDQVFARKLAVDVAYHSHHMKAVADQYRTA

MAAAGVTAVQPESTEPEVEFFSSVTGEKASLTDLGIEYWVANLLSQVKFADSVHRLCMETSASGRARKTKTKAPKRSGAN

NKAKVDMLVEIGPHSTLAGPIRQILGADQTLEQASIRYASALLRKSSAVDTTLTLASTLLMAGYPIDMAAINRPSDHHRV

GVLVDLPPYPWNHSGSYWAEPRLSKAYRNRAHPRNDLLGVLDTHSSPREPRWRNYLRTSEIPWIKDHMIQSNVVYPAAGY

LTMAVEAIGQRIGDNFPGHRISGYRLRDVAIEAALVISDDSEPEVMLSLRPSGDSGLVPAERWHEFHVLSVTPDNRWTEH

CRGLICAEVAAMDGDEDDRGAEAGLTAETERWIEEAEQLCQKDVDIPRFYAELTGLGLEYGETFANMTRARSASHVCLAE

IEVADTAAVMPLGFQSPFVVHPSTLDSLFHPLFVALSSDESLQDPAVPVAIEEIWIRHGMAKEAGHKFQVCASTQETGRD

RIQAAISVVDAQRARSGPALTVRGLTCQFLDRASGDVEGDEQPTRLAYELHWEADVDLLSSSDLATLCAVGRPRDVGEKV

ARYVKLLGHKNPHLAILEVGAGQGELCIPVFRALAGEANSTPSFQSYTLADTEPGLSETIATIADQFDERADLIQYKELD

ISSDPLQQGFNAHSLDLILLPSRGVSATLRSKILKHAHQLLTPEGRLIVVDTRDLQEWWQALRESNFTDPEVIHDSPSET

EADISVLVSKPQPQPRDQTPSDPPDVLVIAENQDSGVSIEHLQRLLADAHVPATVTDFAHADPEGKTCIVLSELTTSLLS

HPDQHSFETLKRILVAGGRGVLWVVRGATGPAPTSSLATGLLRTIRSETDDDRPIVSLDLDASHPLSAESAAQSIFSAFR

HRFVSPGGSHEVEYAERDGILRIPRVVESSLVNHEIVSSLRPAVAEDQPFFQPGRPLELTVGTPGRLDSLYYVDRSCISE

LPSDYVEIEVKAIGLGNGDVKTALGHDDAATRLGAECSGVVTALGDAVSGFKIGDRVAGFGAGTVATLYRDQAARFQLIP

DDMSFARAAALPVAYITAFFAVHALGQVSRGDRVLIQDAGTAAGQALLELCALAGGDIIAVVDSPSQRAFLVGEYDLPAS

RILVGLRGRRLATSVMTLTRGCGVDAIFNFRGGEERRLCWSCVAPYGRFIDLGGGPSDLTDMPQLEMASFFSKNASFTAL

DFHYLVTQKPQAVHRIWSDVMALVRAKAIRGPPRLQLHSVSEVETALKQSQDGCDVEKVVIRAERDTIVQAIPPPKGDLL

RADASYVLVGGLGGIGRAMASWMIANGARHLIFVNRSGLARNEARETVESLEGHGAHVAVYSCDVSDRDQVAQMVAQSSK

EMPPIRGVIQAAMILRDMLFEKMSVDDFNTVLQPKWQGTWNLHGLLPRDMDFFIMLSSISGVIGNATQAAYAAGSTFLGA

FAQYRSSLGLPAVTLDLGVITGIGYLSEHEELLQGMQRQGFEGTNEQTLMALIRSAIVSPRRTGSQAEIVTGLGTWREGV

SLGNFDQPLFAHFRRQALGLRDATAEGPGTSVRESLRGCKTLDDAVALVCAALIDRLASRLNTPVDNIDSQRAMSEYGVD

SLVAVEMRNWIGKEMESTMPILELLANQSISQLSEKIAQRSKVVAVSGSEE

>BGC0000012.1|BAD83684.1|MIBiG

MDKPVAIIGIAFRGPGDARDPEAFYRMLVEGRNARTEIPKDRYNVDAFYHPDSERLGSIQQRHAHFLQQDFKVFDAPFFS

ITPKEAKAMDPTHRILLEATYEGFENAGLSLEKVSGTQTSCYIGTFTADFPNLQARDNEGPSIYHATGMSASLASNRLSW

FYNLRGPSLTVDTACSSSLTAFHLACQSIRTGEAEMSVVGGANLMFGPDMSILLGAAKILSPEGKSKMWDANANGFARGE

GFGVTILKPLDAALRDGDNIRAVVLATAANEDGHTPGISLPNSEAQQELIRRAYQIAGVDPAETAYVEAHGTGTQAGDPL

EARAILKTIGSVEGRKSDLYVGSVKTNIGHLEGAAGVAGIIKAALTVERGMIPPNLWFEKLNPEIDLPDNVKIASKLTPW

PHSGPRRASINSFGFGGANAHAILEDAASFLSRHGLSGRHTTAPLLPSLSSVRVSHPSDSPSSSASTVAEEATSGESSDN

ISDVSSEVLRDVYPIPKLFVLSSHDQEGVKRSAERLQEYLASKTSTTPSFLNDLAYTLSAKRTTLPWKSYAVSSSVSGLR

EKISSLPPVVRSSTSAIPRIAFVFTGQGAQWHAMGRGLSVYKTFAESLQRSEVMLKSFGCPWSLAEELSRSKAEYKLRET

DYSQPACTAIQVALVDLLSGFDVRPVAVLGHSSGEIAAAYAAGFIDQEAAIKIAWLRGQVNKTVSKNGGMLAVSASADSI

QSHLDGLKSGKAVVGCFNSTKACTVTGDTSAIDELQTMLKDAQVACTRLPMDVAYHSFHMEAAREKYEQALEGIAHGSTS

TIPMFSSVTGTLVDPAQMKPSYWVDNLVSPVNFVAATRSLLSHPQESKSRKAFANLFVELGPHSALRSYLLDIISSENRA

ADTTYTTLLRRNFDGAATALEAMGHLWAHGCKVDLSKVNDVSSDSTNMLIDLPPYAWNHKPYWDESHLSRQHRLRKSPRT

DLVGYRLLGTPEHTWRNFLRCNENPWIREHKVQGDILYPGAGMLVMAIEAAHQLAQETSADEIYGFELRDVSIDTALRVP

DTEKGIEVMTQLHNRRTGTRAAPSSTLYEFTVSSWSEEMSSWSTHARGLVSITFKTFSPSMEREVALGNERYMSSLAEVR

RVCQTPARSFLYDTVETIGMQYGPTFRNMTELYAGPNSSYGVINVPDTKAIMPKGFEFPHVVHPATLDSVLHLIFPSISG

EDQALNEAVVPRSFDRIFVSASIPKNAGAELRGCSSAKKLSYTTWNSNITMSDATMTEPVVIMEGVVLASVGATEDASKQ

LETRASCFAQNWHVDADLLTPSQIKEIIYKRTLKSKDDDSVLDLLEFVCLVYIYRILDWFQTEEGKPHVPRDGFWKSYVE

WMHDTVKQFPALPADVETEMEKARQRIVKSESGDITVQMVDRIGQNLSRIFTREVEALQCMTEGDLLYSFYRGAFGTSFN

TNVAEYVGLIADKQPGLRILEIGAGTGGTTYHVLERLRNPDGTSKAAQYYFTDISPGFLAKAADRFNQDASIMQFGTLNI

ENAPTEQGFSPELFDLIVCANVLHATKSIQETLTHCKLLLKPGGRLVLSEVTIKRIFSGFIMGPLPGWWLGEADGRMGGP

LLDADEWNVALKKAGFSGVDVDVRGDRETSKEPVSLLISTKPKTTAPSLPSCLVITTGTEASNMLADTIQRVFTSAGHDI

SIAQWNSVTKTQVEGKYCLCLAEWEDAILVNLTDDDWDRLREVVLSSEGALWITGGGALDTPFPMKSLMVGLGRAIRNED

AGVRLACLDLDPPTTIDFEEASRTTLKVAYAHIRGDGTEGEFAAHGDKVYVPRVERTLQVDGSLRKYEAKGDPEMVPFKS

CGRPLKLTIKTPGLLDTFQWEEDETYHTPLPEDWIEIEVKAVGLNFKDVLVALGNLAENKLGVDASGIVTRVGSAVTNVQ

VGDRVMTASCDTFATYVRFPAKGAIGVPTGMSFEEAASMPLIFLTAYYALVTAGGIVAGEKVLIHAAAGGVGQAAIMIAQ

AKGAEIFATVGADTKKQLLIEQYGIPEDHIFSSRDTSFVKGVLRATDGQGVDLVLNSLAGEALRLSWTDCLAKFGRFLEI

GKADLFANTGLDMKPLLDNKSYIGVNLLDFENNPTPRAVALWHDTAKMIHDGAIKPIAPLQVFTMAEVEKAFRHMQAGKH

MGKVVVRVDDADVVRAVPRIPRVSIHPDATYMIAGLGGITREIARWLAEKGARYLVFLSRSAASGTDNQAFALQLRNTYD

VTPLAYDCDVGNKAALQAVLDDLKAKGLPPINGCATGAMVLQDCLFDKMTADHVRTTVGPKVHGTWNLHELLPRDMDFFV

MLSSLAGVMGHRGQGNYGCGNNFQDEFASFRRGQGLPAMTIDIGYLLSVGFVAEHDEYVDHVKAMGLKVMHTSDLHGLVA

TAIEGPSQHPGQVMCGLPFNEHDDAWYWMADARFAALRNVAAGSAANAGQAISLREELTRCGTVNEEAVQLITAAIAQRL

ASLMMIPEADIDAGRPLSAYGVDSLVAVEVRNWVAREMAVEVSVFDVMQNVPMTQLAQNLAEKSKLLLGQA

>BGC0000134.1|ACD39774.1|MIBiG

MPSATAQDARAPIAIIGMSCRFPGDAEDPLKFWDLLKEGREAYSEKTHRYNEEAFYHPGGQFNNKRQNVLPVKGGYMLKQ

DPYVFDAAFFNITAAEAISFDPKQRIAMEVTYEAFENAGMTLQKAAGTRTACYIGTSMSDYRDSIVRDFGNYPKYHLLGT

SDEMISNRISHFFDLRGPSATIETACSSSHVATHIACQSIQSGESDMAVVGGIGMLLVPESTMQLNNLGFLSAFGQSRAF

DASGAGYGRGEGCGIFILKRLDKAMEDGDTIRAIIRGSGVNSDGWTQGVTMPSGDAQASLIEYVYKSNGLDYEGTQYVEA

HGTGTKVGDPTEAEALHRTIGQPTPKRKKLWMGSVKTNIGHLEAAAGAASMVKGVLAMEHGFIPPTLHFKNPNPAIKFDE

WQLGVPTKLMPWPACQTRRMSTSAFGMGGTNAHLVLERPNEPAIPILERGAIGVSRKNQKRLFVFSSHDQAGFKRICDRL

VEHVDTLGPKSSNPDYLANLAHTLAVGRSGLTWKSSCFAENIVELREHLTSSSLPEGAVRAAGGQTRIGFVFTGQGAQWA

RMGVELMDRKVFGKSVAKSTALLQEMGCEWDPVVELSKSQKESQLVKPEISQPICTILQIALIDELRSWGIRPAKVVGHS

SGEIAAAYCMGALTHRDALAAAYFRGKASANVKRRGGMMAVGTTPEDAKKLITETKAQATVACVNSPRSITLSGDVDALE

ALRETFEKQGVFARRLKVDVAYHSSHMRSCSAEYQSSIMDLEPSELDGANESKEPILMVSSVTGGLVDAEALGPYYWIRN

LISPVLFSDALKELVCPADSGGSSDVDMLIEIGPHSALRAPIEQILSHHDIKNVEYASMLTRGESGSETILGFAAELFRR

GVPFDIAKANDDAQCRLLTDLPPYPFNHSQQFRAESRLQRETLTQQNPTKSLIGAERPSLDEHERVWRGFINLDDEPWLR

DHTVGSTVLFPGAAVITIVLEAAQQMAEAGKTIRSLTLRDISFMAMMTLLEGTPTEVITHVRPHLVATTGTTPATWWEFT

VSSCTGVTSNVRNNCRGLFSINYEDSRSSHMEMELERFEGDRVATYHQIKKECVEVISKQAFYDTLARSALAYGPHFQGV

DNCRPGNGQTAFEVIVSDLGETFNKDKLTRPFLIHGGTLDSIFQAWVGSTKDSNGPGSFGFEKPLLPKSIGELEISLDFP

GEVGYSLNGLSTSKKHGFSEWSTDITMFDRNVSKLLLSVKDFHLAELEVEDADRPDRTEHVDVDPAEISSEPKWNYALDF

MSTQEIKQVVETASSSDDKLMQFISLAIHQRPNLEILELVESANQLRQTAVSKLPRGRLLPNQASCAILGGDYDNNNESA

AAFGRIFGLDSSEAVPSDVAPADLVIANFNISNLEDIAERLVVLAKPEARILLIADKKVDSSVTSLADKGFDLVFSTEAD

SESLSLYCFGKKEEPQPERLTNGSTGQEVVILEPSSLSAESDRFSKDLQHALDNIGYNVSTVTDIHGAHAAKARIYVSLL

EIEQPVLENLSQSEFEGLRDLLLNCDRLLWITRGDGPSLQLVDGFSRTIRSEFAGVEFQVLHLSGKNSRQGPSLAAQIVF

KQSTESEFREDDGHLQISRWYRSVEEDDHIRNHLLDSIRTVSLPVGGNIEDNSSYRLAVGKPGLLNTLHFVSDDNTEAPL

ADNEVEMQVKASGINFRDIMGSMGLLPVSGIGQEASGIVVRVGKLGASSLKPGDRISTLTVGGTHATRIRCDYRVAKKIP

EGMSFEEAAGIPVVHCTAYYALVKLAKLRPGQSVLIHAAAGGTGQAALQLAKHLGLTIFATVGTDTKRALIREKYGVPDE

NIFHSRDGSFVKGIERATNGRGVDCVLNSLSGELLRLSWGCLATFGTFVEIGLRDITDNMRLDMRPFAKSTTFSFINMVT

LLQENPDAMGEILESVFEMIHQNVLQPVFPVTVYPVGKVEEAFRLMQQGKHVGKMILSFAAGDARAPVLCRAKDSFKLDP

NATYLFIGGLGGLGRSMAVGFVACGARNIAFLSRSGDSKPEAKAVVDELRELGTRVQVYLGDVSDEASFRGAMEQCSREL

PPVKGVIQMAMVLRDVVFEKMKYDDWTTGLRPKVQGTWNLHTFFDKDRPLDFMIFFSSIAGVFGNPSQAQYAAGNTYQDS

LAKYRRDRGLKAVSVNLGIMRDVGVIAEGDSHFMQQWEEVLGIREPAFHALIKSIINGQLETSNIREAAKCPVQVTVGLG

TGDILARNKIREPDYFRDPRFGALAVCSSTSTAAASSGENGVSIASQLAGLSNEADPEEAAGPIITKALVSKLAKILQVP

PSEIDSSRPMYRYGVDSLVAIEVRNWITKEMSANMSLMDILGAMPMEQFAVQIAKKSKLVGGS

>BGC0001697.1|EAA36364.1|MIBiG

MAPHSTLDSDYSSGSSTPTSASAAGDGFVDGLNGLNNGRAVDPQEPIAIIGMGCRLPGGSHSSSKLWELLKAGRTAQSRF

PPSRFNIDGFYHPNSDRPGSLNMEGGYFIEDDIRGFENSFFGINNLEATYMDPQQRKLLEVVFETFENAGFTLDQVSDAN

IGCYVGNFVTDFITMQLKDSEYTHRYSATGLGTTILANRISHVFNMKGPSFVIDTACSSSLYCLHAAVAALIAGECDSAI

VAGANLIQSPEQQLATMKAGVLSKTSTCHTFDSSADGYGRADGIGAILVKRLSDAIRDGDPIRSVIRGTAINSNGKTNGI

TLPSADGQEAVIRKAYAQAGLGFNETDYIECHGTGTAVGDPIEVEAVSRVFKKPQGAPLLIGSVKSNLGHSEAASGLSSI

IKVAMALEKGEIPPTYGVKNINPKIKTDEWNVQIVTETTPWPKNLPHNAGRLFRRAGVNSFGYGGANAHAILEAPQMHVP

VGYNRGSLPASLTRSTLFLPFSGSNTAALERRVTDIAAAIDFENVNIADLAYTLGVKRTHLSTRGYILSGQDTLKDDLKP

ENLRVALQGKTYSKLPLAFVFTGQGAQWPEMGKELMKEFPSFRRTIQRLDAALQMLPHAPTWTLQGAILEPAKTSMINHA

SRSQPVCTAVQIALVQLLASWGIKPESVIGHSSGEIAAAYTAGYLTPEQSIIIAYYRGHCVTKSTMVGAMMAAGLGAEDA

NKKISELDLVGKIRVACVNSPESVTISGDTEGIETLRAQFDQAGTFARVLKTDGKAYHSHHMAVIGQEYEDLLTEALDGD

DFPTTSNGVRFISSVTDAVVNHAVGPAYWRANLESPVLFANVVERLIKDTASHLVELGPHSALELPIKQTRTKLNISETK

VHYGSALSRGKNSITTILNLVGDLFLHGHDISFKGVNYVDSAFNSPKARKNVKTQEKMLLDLPNYTWDYSGTVFNESRVS

VEWRNRKYPRHDLLGSQVHGGNGISTNWRNVVKAKDIPWMEGHKLDTTTVFPAAGYLAMAVEAMCQVADVTKEQEPALSL

RNVNITKALTLGSEETDAGVELFTTLYPAQLPGGATDAGWYQFNISSYTNGTATTHANGLVKIDSAPAPLEVNLPIVPST

MEPQAPRTWYGKFAKGGLNFQGQFQSLTEIQNPRKKENPHTLAKTELRQGGGSGPSTESEYLIHPITIDALFQAGIIAST

SGVVRELRAKVPVHIEEMHLRAPVGGQKELKVNATSEAVGFGTIRVDGELFDDEGRVFLQINRCRQVSYQSGIQQEAGDE

RHPMLRVVWKPDVTRLGAGDAKEFSQYIEQYAAKSESKVDDATVRLGAALDLLIHKHPRLRILNLDVNLTEFLVDTLRLE

TDFKKCKTLVSGSYSEDGTLTFEDLTNEGKTSTAAQVFDVVILGSKAQELEAAKELVDENGSIIVNGSPADADKLQTLGF

TTLQAPSDTILAQTPQEITAKQQKTLSKQVLIVERNADHVLNSAIAAQAKKITGLEAKRIPLESVTADIIAAHTRVISTI

ELENPVLSRVTEDEMKHIKTLTDNCTNLVWVTGGRLFQSASPEHAVVYGLSRALMLEQPSLRFFVVDVDHEGTPVERSAK

HVVEVLQQALIEADPDYEFVQNAGLLHVSRFVPEETLNRVFREKQGAEKLALPLKDARPFRLGTDMVGQIDSIFFRREEA

KDVQLADGHVEVSVKAVGLNTKDLQAINGDGDNTSGSFCTSQYTAVVANVGTGVENLAVGDRVVVMTPGYFATTESVPAW

ACQKLADNEDFTTLSSVPLQLSTAIYAVNNRAHVQAGESVLVITGSDIAADQAAIRVAQLAGAEVFAVGESTNLPSERVF

TKGDKALVAKLLKATEGRGVDVVLNFANDAAPISSIGNVFADCGRLVHVGKSSLAEAIATDSTLFRKSVTVTTFDIANIL

SLKTVAGQKIRSQLLADSIALYRQGQLNLASSPKVFDVSEVRDAFRALAAKGHSGSVVVSLENEASLVPTLPLKYDTVLS

PEKSYLLVGCLGGLGRSMSKWMLARGARKFVFMGRSGTDRAPARRLVEDLELAGAQVTVVRGDVINMEDVELAVNGIDGP

IGGVIQAAMGLDEALFTTMPRDYWLTGLKPKIVGSWNLHNAIRGRDSELDFFLMTSSISGSVGTATESNYCSANYFLDVF

ARHRHSLGLPATSIGLGMISEVGYLHENPEIEAMLLRKGIQAINEDEMLQIIDASLATPTAVPGSYDELARAHVLTGLEP

LGLKELRAKGFEGTSPVLGDPRASLLSAALDESTDAASSNAASGMPAEVAEAIATGASVEDAVLKMISKKFSNLVLIPED

KLNLTKPISEVGVDSMLAAEFRAWIFQAFKVDVPYLTLLSAAATLTLLSELITKKMMEAQDA

>BGC0001244.1|AIW00670.1|MIBiG

MATPDDPATPALSLSASNSSSPTAASSVPPPTGTSEIQYDDVAIIGMSCRTAGGNDSPEKLWRFIMDKKDASGESPSWRW

EPWVRRDTRNAKVIEKTISKGYFIEDLENFDASFFGISPKEAEQMDPHQRLGLEVTWEALEDAGINPQSLSGSDTAVYVG

VDSDDYSRLLLEDIPNIEAWMGIGTTAHGIPNRISYHLDLMGPSAAVDAACASSMVAVHTGRQAILAGESRIAIVGGVNV

CLSPALFHMLGAAGALSPDGVCLSFDEEARGYARGEGAAILILKKMSHAIMDGDHILATIKGSAIAQDGKTNGIMAPNAK

AQELVARKALKQAGINALTVGYIEAHATSTPLGDPTEVSAISAVYGVGRPTDTPALIGSIKPNVGHLEAAAGAISLVKAV

MAVQKGIVPPQTRLNKLNTRVDWAKSGLHVVRESTQWGTEDSPRRAAICSYGYGGTVSHAIIEQFAHAADPFTASTSDDN

HPTLLLLSAPQGKQRLPAQSAALAEWISPAGAHESLRSIAATLATRRAHHENRAAFIVSSHTEAAETLNLFSKGAEHDSI

VQSRTLDNNINKQIVWVFSGHGSHWSGMGKQLLQNAVFYRTVAPLDIVVVQELGYSAIEALKTGRFESSGQVQVLTYMTQ

IGLIQLLKAKGVHPHAVIGHSVGEIAASVAAGCLTPEEGMIIVTRRARLFAKVIGCGGMFLVSLPFAEVLAELGGRTDIV

AAIDSSPSSCVISGLNAPLEEYVEKLKNRGIRVFQVKTDIAFHSPMLEVLSKPLKESLEGSLNPQPPNIKLYSTSQADTR

HPARRDAEYWVDNMVKPVWLRPAVTAAIEDHYRIFMEVSTHPIVSHSLDETLAENGASDFTTIHTMKKEQSAEKCILHAV

AQLWTKGVKIDFKFLGRQWSREVPKIRWSHKRFWKEVSTGSASAQTVHDPDKNNMLGQRMVVAGTNMTIFTTALDESSKP

FPMPHQLHGTDIIPVSVYVNTFIKATGGKVLSKMELRVPLAVTNDVRNVQVIVDGQSVKVASRLSSSDDMSWVTHSTASW

ENEPSANVLPSLDVSSVIKRIGTRVSETFSVDYLKKTGVSGMAFPWAVNDHYNNTKEMLVTLDNDPEHETMSWDPCSWGA

TLDAATSVGATLFSREVKLRIVSHIDRLTIYSSDPPPKRYHLYVTEASSSQVHACSADISVLDLSGTLLARIESIRFTEV

EATPTKSTSIESGVHQIAWVPARLSEKPLSLEQIVLVSEDDAKLEQYANELQRQAPKIVKLTSAAKLRDNGALFMREKNA

TVIYCPGTVTSLEDVASASHRFIWEVATAIKFLVENSISAKFFVILDRTFLAGSPTALAQGALYGLARVVASEHSDIWGG

LIDNEGPLFPVMPLKYVQDQDITRYIDGVPRVARMRPFTKQQRYAPSTARTLLPKPEGTYVLTGGLGALGLETCDFLIEK

GARRIVVISRRDIPARSQWSKASENLAPILERVKAMEKTGASIYFVSLDIGAADAHQQLLFALERLSLPPVLGVIHASGV

LEDSLLVDTTSDSFARVLSPKISGALALHKAFPPGTLDFFVLYSSIGQLVGTSGQSSYAAGNSFLDVLAAHRRSQGDNAI

AFQWTAWRGLGMATSTDFLTLELQSKGITDVGRDEAFQAWEHMSKYDVDQAVVTRTLALEADDILPCALLEEVVVRKARA

QDQSAPASGNASDSSGRPTASADLKPWLDVKIRECVALVMGVEDIEEIDTRVPLSDYGVDSIMTIALRQKLQSKLKIKVP

QTLMWNYPTVSAMVGWFQKQFEEGQ

>BGC0000077|ACD39767.1|MIBiG

MPSTSNPSHVPVAIIGLACRFPGEATSPSKFWDLLKNGRDAYSPNTDRYNADAFYHPKASNRQNVLATKGGHFLKQDPYV

FDAAFFNITAAEAISFDPKQRIAMEVVYEALENAGKTLPKVAGTQTACYIGSSMSDYRDAVVRDFGNSPKYHILGTCEEM

ISNRVSHFLDIHGPSATIHTACSSSLVATHLACQSLQSGESEMAIAGGVGMIITPDGNMHLNNLGFLNPEGHSRSFDENA

GGYGRGEGCGILILKRLDRALEDGDSIRAVIRASGVNSDGWTQGVTMPSSQAQSALIKYVYESHGLDYGATQYVEAHGTG

TKAGDPAEIGALHRTIGQGASKSRRLWIGSVKPNIGHLEAAAGVAGIIKGVLSMEHGMIPPNIYFSKPNPAIPLDEWNMA

VPTKLTPWPASQTGRRMSVSGFGMGGTNGHVVLEAYKPQGKLTNGHTNGITNGIHKTRHSGKRLFVLSAQDQAGFKRLGN

ALVEHLDALGPAAATPEFLANLSHTLAVGRSGLAWRSSIIAESAPDLREKLATDPGEGAARSSGSEPRIGFVFTGQGAQW

ARMGVELLERPVFKASVIKSAETLKELGCEWDPIVELSKPQAESRLGVPEISQPICTVLQVALVDELKHWGVSPSKVVGH

SSGEIGAAYSIGALSHRDAVAAAYFRGKSSNGAKKLGGGMMAVGCSREDADKLLSETKLKGGVATVACVNSPSSVTISGD

AAALEELRVILEEKSVFARRLKVDVAYHSAHMNAVFAEYSAAIAHIEPAQAVEGGPIMVSSVTGSEVDSELLGPYYWTRN

LISPVLFADAVKELVTPADGDGQNTVDLLIEIGPHSALGGPVEQILSHNGIKNVAYRSALTRGENAVDCSLKLAGELFLL

GVPFELQKANGDSGSRMLTNLPPYPWNHSKSFRADSRLHREHLEQKFPTRSLIGAPVPMMAESEYTWRNFIRLADEPWLR

GHTVGTTVLFPGAGIVSIILEAAQQLVDTGKTVRGFRMRDVNLFAAMALPEDLATEVIIHIRPHLISTVGSTAPGGWWEW

TVSSCVGTDQLRDNARGLVAIDYEESRSEQINAEDKALVASQVADYHKILSECPEHYAHDKFYQHMTKASWSYGELFQGV

ENVRPGYGKTIFDIRVIDIGETFSKGQLERPFLINAATLDAVFQSWLGSTYNNGAFEFDKPFVPTSIGELEISVNIPGDG

DYLMPGHCRSERYGFNELSADIAIFDKDLKNVFLSVKDFRTSELDMDSGKGDGDAAHVDPADINSEVKWNYALGLLKSEE

ITELVTKVASNDKLAELLRLTLHNNPAATVIELVSDESKISGASSAKLSKGLILPSQIRYVVVNPEAADADSFFKFFSLG

EDGAPVAAERGPAELLIASSEVTDAAVLERLITLAKPDASILVAVNNKTTAAALSAKAFRVVTSIQDSKSIALYTSKKAP

AADTSKLEAIILKPTTAQPAAQNFASILQKALELQGYSVVSQPWGTDIDVNDAKGKTYISLLELEQPLLDNLSKSDFENL

RAVVLNCERLLWVTAGDNPSFGMVDGFARCIMSEIASTKFQVLHLSAATGLKYGSSLATRILQSDSTDNEYREVDGALQV

ARIFKSYNENESLRHHLEDTTSVVTLADQEDALRLTIGKPGLLDTLKFVPDERMLPPLQDHEVEIQVKATGLNFRDIMAC

MGLIPVRSLGQEASGIVLRTGAKATNFKPGDRVCTMNVGTHATKIRADYRVMTKIPDSMTFEEAASVAVVHTTAYYAFIT

IAKLRKGQSVLIHAAAGGVGQAAIQLAKHLGLITYVTVGTEDKRQLIREQYGIPDEHIFNSRDASFVKGVQRVTNGRGVD

CVLNSLSGELLRASWGCLATFGHFIEIGLRDITNNMRLDMRPFRKSTSFTFINTHTLFEEDPAALGDILNESFKLMFAGA

LTAPSPLNAYPIGQVEEAFRTMQQGKHRGKMVLSFSDDAKAPVLRKAKDSLKLDPDATYLFVGGLGGLGRSLAKEFVASG

ARNIAFLSRSGDTTAQAKAIVDELAGQGIQVKAYRGDIASEASFLQAMEQCSQDLPPVKGVIQMAMVLRDIVFEKMSYDE

WTVPVGPKVQGSWNLHKYFSHERPLDFMVICSSSSGIYGYPSQAQYAAGNTYQDALAHYRRSQGLNAISVNLGIMRDVGV

LAETGTTGNIKLWEEVLGIREPAFHALMKSLINHQQRGSGDYPAQVCTGLGTADIMATHGLARPEYFNDPRFGPLAVTTV

ATDASADGQGSAVSLASRLSKVSTKDEAAEIITDALVNKTADILQMPPSEVDPGRPLYRYGVDSLVALEVRNWITREMKA

NMALLEILAAVPIESFAVKIAEKSKLVTV

>BGC0001067.1|EAL85129.1|MIBiG

MTLTYGHKRLQDAPEPIAIVSAACRLPGHVNGPHKLWELLQSGGTAVSNEVPQSRFSSEGHFDGSGRPGTMKALSGMFIE

DIDPAAFDAAFFNLTRADAIAMDPQQRQLLEVVYECFENGGIPIEKVRGKQIGCYVGSLNGGKSLWMSRWSVADIIRILI

DADYHDMQMRDPEQRVSGHAVGTGRAILSNRISHFFDLRGSSFTIDTACSSGLVGVDVACKNLRAGTLTGAVVAGVNLWL

SPEHTEERGTMRAAYSASGKCHTFDAKADGYCRAEAVNAVYLKRLSDAVRDGDPIRAVIRGTASNSDGWTPGINSPSAQA

QAAMIREAYANAGIDSSEYAETGYLECHGTGTPAGDPTEVKGAASVLAHMRPPASPLIIGSVKSNIGHSEPGAGLSGLIK

AMLVVEEGEIPGNPTFLNPNPAIDFDNLRVYATRIRIPWPKESSHYRRASVNSFGFGGSNAHAVLDNAEHYLGKYWASLE

IPRSHLSSYINLSDMLSLFDGRRSSKTVTRRPQVLVFSANDMDSLKRQISTLSAHLLNPRVKVKLSDLSYTLSERRSRHF

CRAFLLSYPAKSGHASKIAVEEAQFSKISQEATRIGFVFTGQGAQWSQMGLELVRTFPGVVKPILEQLDNVLQELPADLK

SEWSLLQELTEARSSEHLSRPEFSQPLVTALQLAQLAVLQSWGVRAEAVIGHSSGEIAAACSAGLLTPRQAILNAYFRGL

AGKSALATSPKGMMAVGLGAQDVQPYLEGVSADVVIACHNSPASVTLSGSASTLAELEGTIKAAGHFARMLRVEVAYHSP

HMAKIANRYEELLKEHGRLDDGSKTNKRSNRMISTVTEDEVTGAQVCDAAYWKANMLSPVRFDGACNKLLTNTQLAPNFL

IELGPSNTLAGPVTQIARAAKVDNLTYAAANKRGPDESSRAIFDVAGHLFLQNADISLDKVNLGDNTPDKAKPAVIVDLP

NYQWKHSTHYWHESLASKDWRFKKFPSHDLLGSKVIGTLWQSPSWHKMLRLSDVPWLRDHRIGSEILFPAAGYLAMAMEA

VRQAALSTATAEARELLKTRHYRYCLRDVQFPRGLVLEDDAEVHIMLLLVPMAKLGQGWWEYKITSLAESDSVASSSSST

LSPEKWNINSTGLVRLETILEASSSRAPEHTCSLPLDNPTPGQMWYKSLRDAGYSYGPSFQRLVAVESTEGKSATRSLIS

LEPPRSKWEPQSEYPLHPAPLDSVLQSMFPSLHRGNRTKLDQLLVPRGIGELTVSGDIWKSGEAISVTTWNKVSGDASLY

DPASRSLIMQLNSVSFSPMLDGRDSLYMSHVYTQLTWKPDFQLLDTDEKLQQALSGGDGAASSLVQDLLDLAAHKAPNLR

VLEFNLVPGSSESLWLAGHPTPRAVRTALTEFHFAANSADTALAAQEEYAEWPAARTARFSVLDPFSKALAVPAGSSQFD

LVIIRRPQHADLGELDILVGNLRRLTSDGGSVIFYDSKQSSLSGGRGLANGHNHFPAALQRFGLTKVRQTRDGSCIVAEV

SPAQNLSLRNDFRVVIVRFSTARSTIIDHTISQLRQFGWTLTEICIYNESGTGLPQLPPKSTVLVLDELDRPLLATATDH

EWTALQAIIQSECNLLWVTEGSQVRPTAPLKAVAHGIFRTVRAEVPMMRIVTLDVESATTESLGTNASAINMALREITLA

DRSSLPIECEIAERGGLLHVSRIWPDAGVNKRKVEDNAGGAPPVLTNLHDSKSTIRLMASRPGSLEALHFAEQGRDVCSR

QDMGPDDVEVEIFAAGCNSRDIDVAMGDISGDLDGLGLEGAGVVVRVGACVSARCVGQRVAVFGKGCFANRVTVSCKATF

PLPDAMSFEQAATLPIALLTALYAVGRLAHVQGDDRVLVHSPCTDVGIACIRLCQRSGSTPFATVDNLEQRHFLTHELGL

PEDHIFMSEPAAFPRALRHATKGHGLDVIISQPANRNLDNENMRLLAPGGRLIGIANGGADVGNLLPTGSLAPNCSFQRL

DVTALPEKTIESGNSFDSVFLELSRLVTDGSVQPLSPSTLLGYEEIPKALQLLREGTHIGKIVISDPRGTKLAVLTRPAT

TLAQSMINPSHCYLLVGGLKGICGSLAIHLASHGAKNIAVMSRSGGGDQVSQGIARNIRALGCSLDLLQGDVTSISDVRR

AFSQISVPLGGIIQGAAVFRDRTFESMSHEDYHAAVSSKVTGTCNLHTVSLETNQPISFFTMLSSISGVIGQKGQANYAG

GNAFQDAFAEYRRALGLPAISIDLGPVEDVGVIHGNEDLQNRFDGSTLLSINEGLLRRIFDYSILQQHPDPQHRLNVTSQ

GQMITSILVPQPEDSDLLRDCRFRGLRALGEHSPRSRRDPTKDKEIQSLLFLAQSQDPDRAALRAAAITVVGARLAKQLR

LTDAVDPARPLSYYGLDSLAAVELRTWVRMTLAIELTTLDVMNAASLGELCEKVIGKMGFGM

>PspA

MLAQDVEFVDLPPPEATAGAATTDNETSSFNSNPVPTPSEASSIGPPHQLPVPVPDGDQPPLVEPMAICGMAMRLPGGIR

DAEGFWDLLYNKRSGRCRVPKDRYNVENWFGPGKIGHVASEYGYFLDDVDLRNADASFWSMTKQEIEAMDPQQRLSLEVT

YECLQNAGQRPEELRGRKIGVYLGTFEGDWLELDGRDPQHYHMYRLTGYGDYMSANRIHYEFGFMGPSVTIRTACSSSLT

GLYDACHAISAGDCDSAIVACANIIYSPRTSITMQEQGVISPSGFCKTFDANADGYARGEAVSAVYVKKLSDAIRDGDPI

RSVIRSTCINAGGKASTLTAPNTAAHETLIRRGHELAGVTDFSKTAMIECHGTGTAVGDPIETAAVANVFGEHGIYIGSV

KTNLGHSEGASGLASIIKMTLALEHKIIPPNINFTTPNPKIPFERCKLKVPTEPLPWPKDRAELVGVNSFGIGGSNAHVL

LGSAASFGIGSVQQKIIASEQSAEVAMTELTPRLLLFSAKHQQSLERMVANHQAYFLSHPESLDDMAYSLALKREELSHR

SFCVTNGEDDWVPSRTHRTSGRAPPMLIFTFTGQGAQWAQMGKALIDQVPRFRRSIEKLDQVLQALPTPPRWKLIDEIRA

SKKKSRLSEAELSQPCCTAIQIALVDILEHYGIHPDAVIGHSSGEIGAAYASHAISGADAIQIAFYRGLVMCSLNPAERP

GGMAAVGLGAEELTPYLRPGVRVGCENSPNSTTLTGDKVSLEETMKAIKEANPDVFVRALQVDRAYHSHHMETVAPEYVE

LLTNQRVQAMDPSVKFFSSVTGRQVDQSKELGPLYWAKNLVSPVRFSTAMEELVQSLIGPKVFLEIGPHSALAGPIRQIL

QHHKSTDEYFNTLTRGSDSHKDLLKAVGEMWLQNIPVNLTAVLGEGRFLPDLPLYPWHYEEPLWCESRLSKEWRLREFPH

HDILGSRVLESTDQNPSWRNILRLDVVPWIKEHEVAGEIVFPGVGYICMAGEAIRQLTGETGFTARRVHIKAALVMHQGQ

DVEVITQLQRIPLTNAADSKWYNFTVHSYNKGIWVKHIFGQVCAGSDREHQAPSLESLPRQLSRRGWYRKMKEMGLEYGS

RFMGLTDMTAHPIERKTIATVVNDIREGESKYAVHPVSLDCLLQAIVPATFNGLTRRFQHLGIPTYMEEIYVCPPLQPEM

IIEACADEQPTAALSGSIIAVSNGHVTIDIRGLQMSAIGDAADASGQDPHAAVELEWREDINLISDAAKLIHPAKDRTDL

HHLLDRFASASMMDTSTRLRGVEPTRSHLTHYQKWIESTADLIKLGKYPGLQPEDEIVEVSDAERVNIIESLYLSLLETD

AAATATAIYRIWKECQGIFTGETDELELLLEGEVLHSLYDFMQNSEYRVFLELLAHRKPNLRVLEIGAGTGGTTATVLPA

LKSLYGERMYHSYTYTDISAGFFPQAKKRFENYPGLEFATLDISQDPLSQGFEAESFDLIIACNVLHATSTLQDTLTNVR

RLLHPQGRLFLQELSPATKWINYVMGVLPGWWLGEQDGRYPEPYIGIDQWDALLSQSGFSGINLVSHDGYLNNNIVARPA

ADTQRQKRITLLHSCEDSASVTTSISQLLSSAGFGIDLYAIENANIPTPTQQDIVSILDLDRPFFHDLHESLFENLKGLL

SQLRDTDSGILWVTRASQVGCKDPRYAMVNGVARVIRTELNIDFATLELEDFEQETLALIPQVLGEFQQRISEPNINTTT

EWAVVGQKPLISRYHYIQVAEELKNNAVADSSTVKKLEQSRPGLVDTLCWKSMPISHALDENDVLVQVKCVGMNFKDVLI

STGVITEKSSIGRGLGYEGSGLVLQVGSAVHKLSVGDRVIMSSSGSLTTTQQLDQRLCVKMPDSMTYEEGATMSAVYCTA

IHCLLDVGGLRKGQSVLIHSASGGVGIAAMYIAQMVGAEVYATVGSEEKTQSLMSTFNIPRNRIFNSRTSEFLPRIMEET

NGMGVDVVLNSLSGELLHASWKCTAEFGTFVEIGRRDFVGQGLLDMQPFEPNRSFVGFDLLLFSNKRPERIESIMTRAMD

YYRAGFIQPIKPMTMFDAVSIVDAIRYMQRGQHIGKIVITMPENSTELSAEPPRQELALRQDRAYLFVGGLGGLGRSIAT

WLVEHGARHLVFLSRSAGNVPDDDPFVQELAVLGCTTTRISGDVSKLDDVLLAIRASGKPVGGVLQSSMVLRDNSFVDMN

WDEWLGAVQPKVLGTWNLHNALLSEQPEEALDFFFLFSSAGAMSGQWGQANYNAGNTFLDAFVAYRHSLGLPASTVNIGV

IQDIGYVSQNPEILDSLRSTAQYLMREPELLESIELMLHRSSPAESVVDHAVGRYVTRSQIGIGMRSTVPMDAPSNRTIW

RKDPRMLVYRNLEVQSGPVSSSTGSDQVLTQFLREIGSNMTMLKAPETAELLAGEIGRTLFGFLMRADTEVVDLDAPLAS

VGIDSLISIELRNWIRRKIGVEVTVLEIVRADSVRDLGVLAQKKLAEKYEARM
