# Supplementary figures and images for: Evolutionary Histories of Type III Polyketide Synthases in Fungi
Source: Front Microbiol. 2020 Jan 21;10:3018. doi: 10.3389/fmicb.2019.03018 (PMC6985275; doi:10.3389/fmicb.2019.03018)

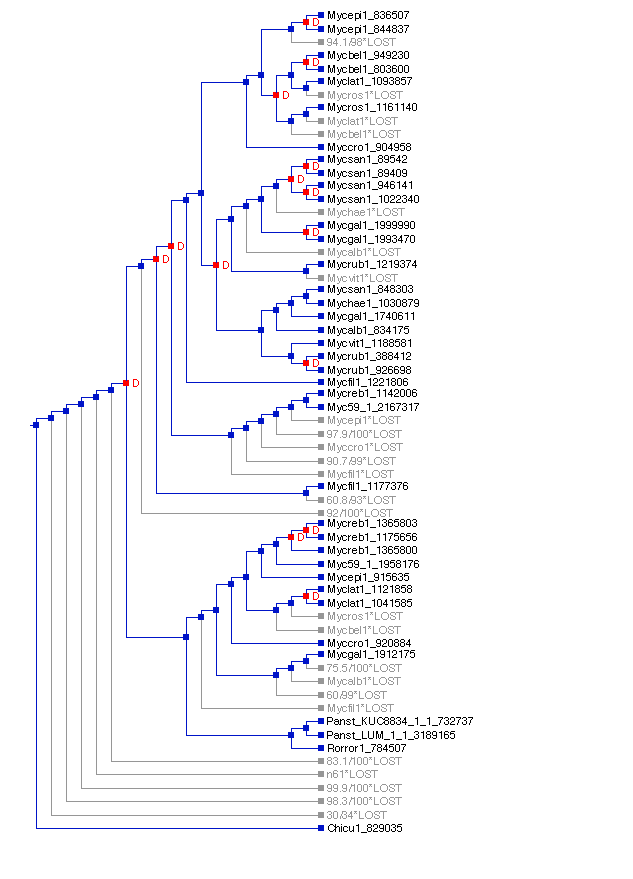

Supplement: MATERIAL S6 — Reconciliation analyses performed with DTL and DL models using NOTUNG. [file Data_Sheet_6.zip › NOTUNG_clade3_DLmodel.png]

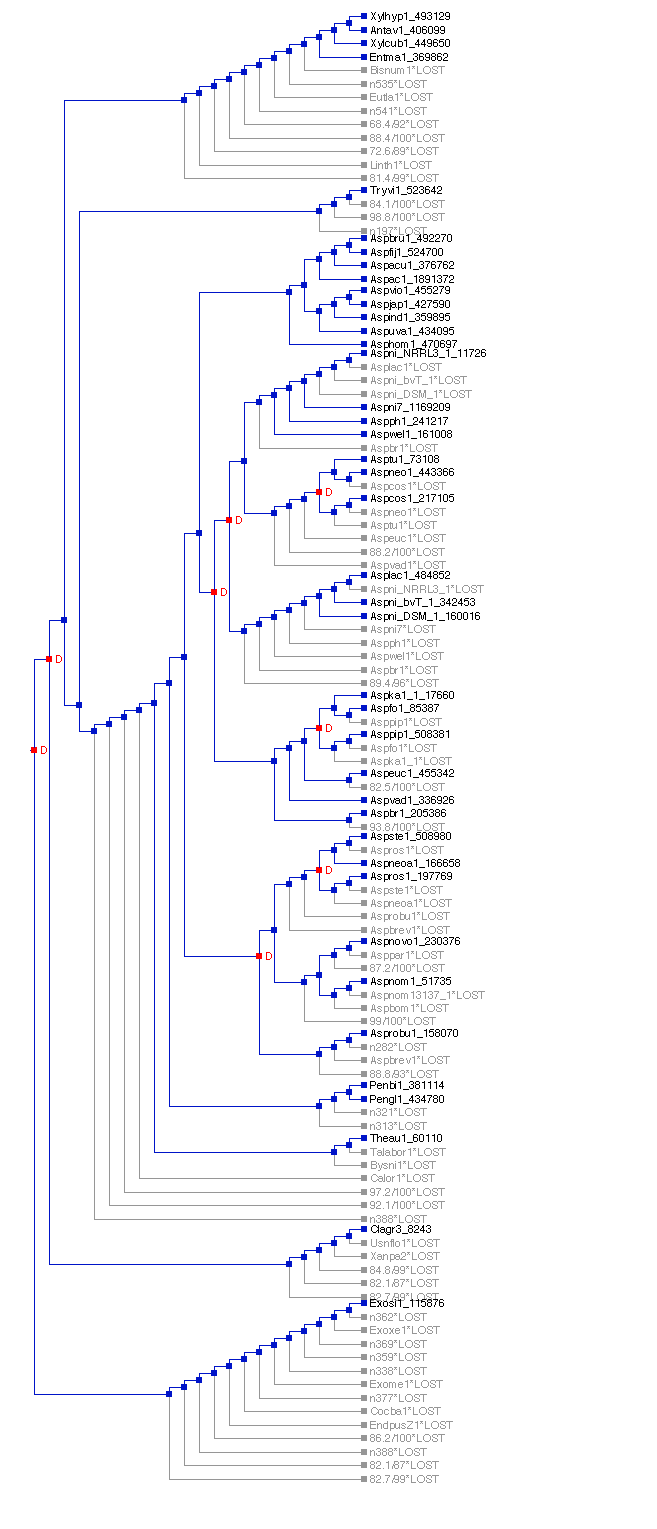

Supplement: MATERIAL S6 — Reconciliation analyses performed with DTL and DL models using NOTUNG. [file Data_Sheet_6.zip › NOTUNG_clade6_DL.png]

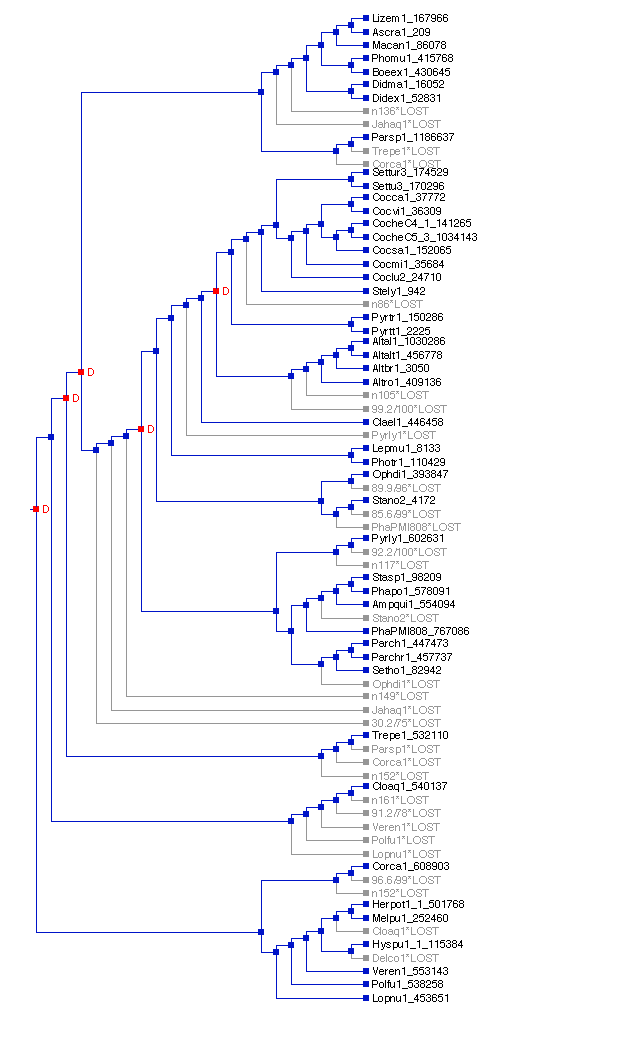

Supplement: MATERIAL S6 — Reconciliation analyses performed with DTL and DL models using NOTUNG. [file Data_Sheet_6.zip › NOTUNG_clade5_DLmodel.png]

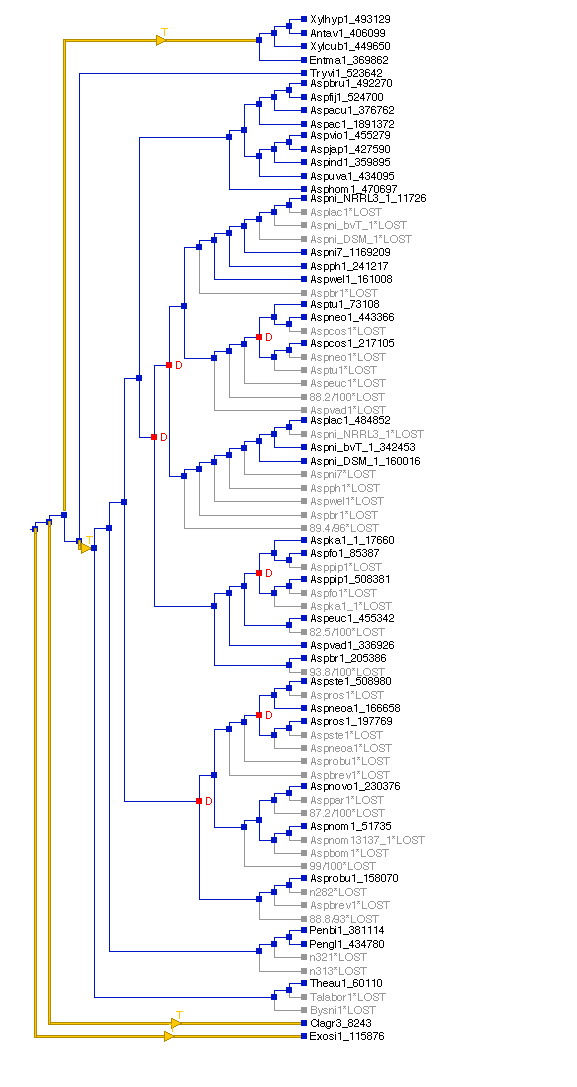

Supplement: MATERIAL S6 — Reconciliation analyses performed with DTL and DL models using NOTUNG. [file Data_Sheet_6.zip › NOTUNG_clade6_DTLmodel.png]

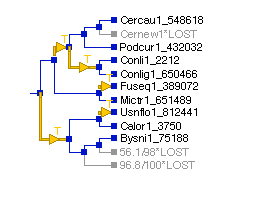

Supplement: MATERIAL S6 — Reconciliation analyses performed with DTL and DL models using NOTUNG. [file Data_Sheet_6.zip › NOTUNG_clade7_DTLmodel.png]

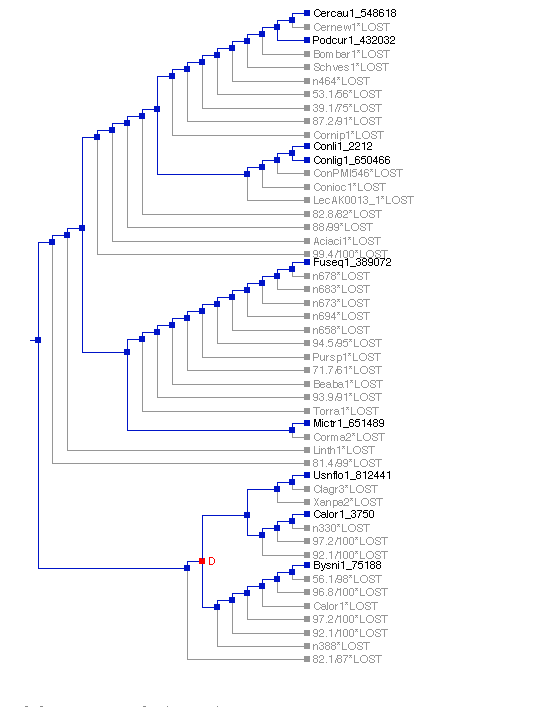

Supplement: MATERIAL S6 — Reconciliation analyses performed with DTL and DL models using NOTUNG. [file Data_Sheet_6.zip › NOTUNG_clade7_DLmodel.png]

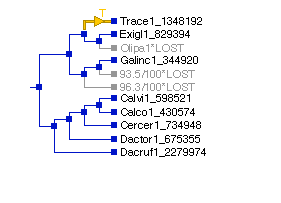

Supplement: MATERIAL S6 — Reconciliation analyses performed with DTL and DL models using NOTUNG. [file Data_Sheet_6.zip › NOTUNG_clade2_DTLmodel.png]

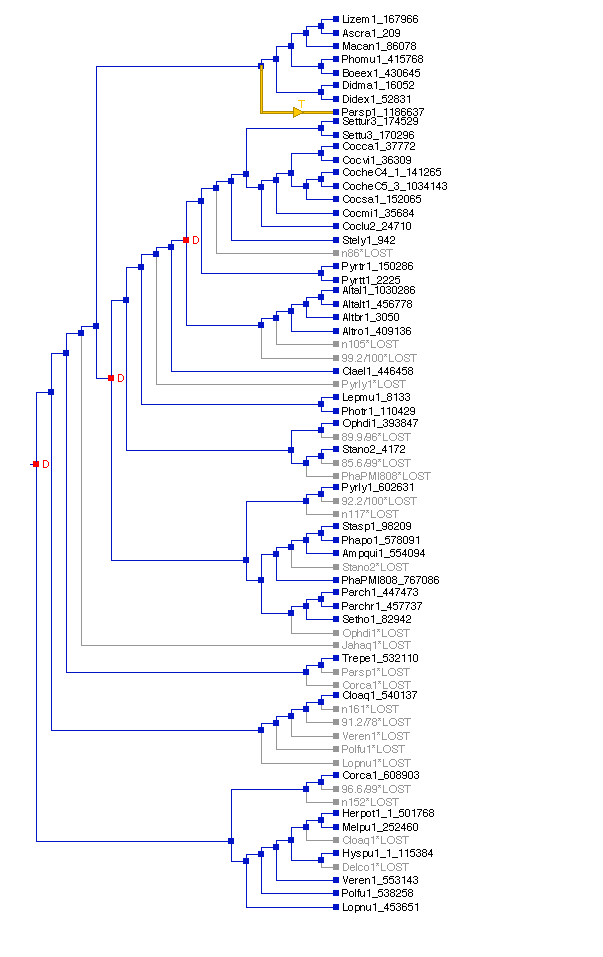

Supplement: MATERIAL S6 — Reconciliation analyses performed with DTL and DL models using NOTUNG. [file Data_Sheet_6.zip › NOTUNG_clade5_DTLmodel.png]

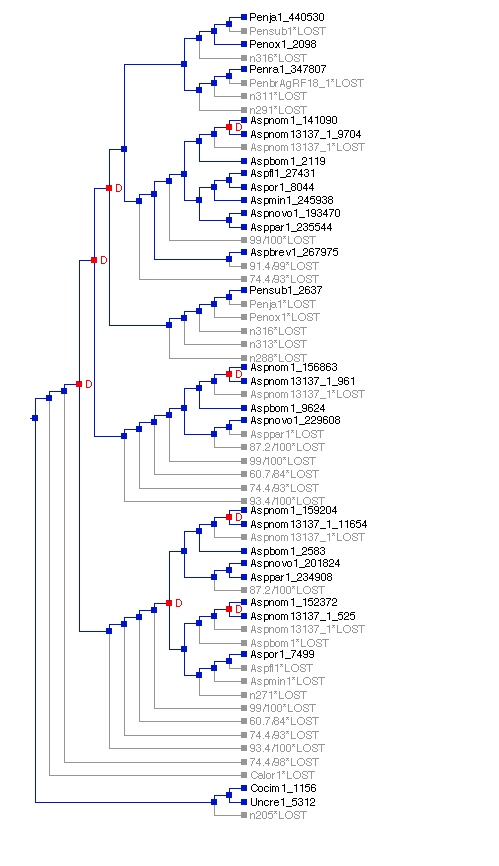

Supplement: MATERIAL S6 — Reconciliation analyses performed with DTL and DL models using NOTUNG. [file Data_Sheet_6.zip › NOTUNG_clade4_DTLmodel.png]

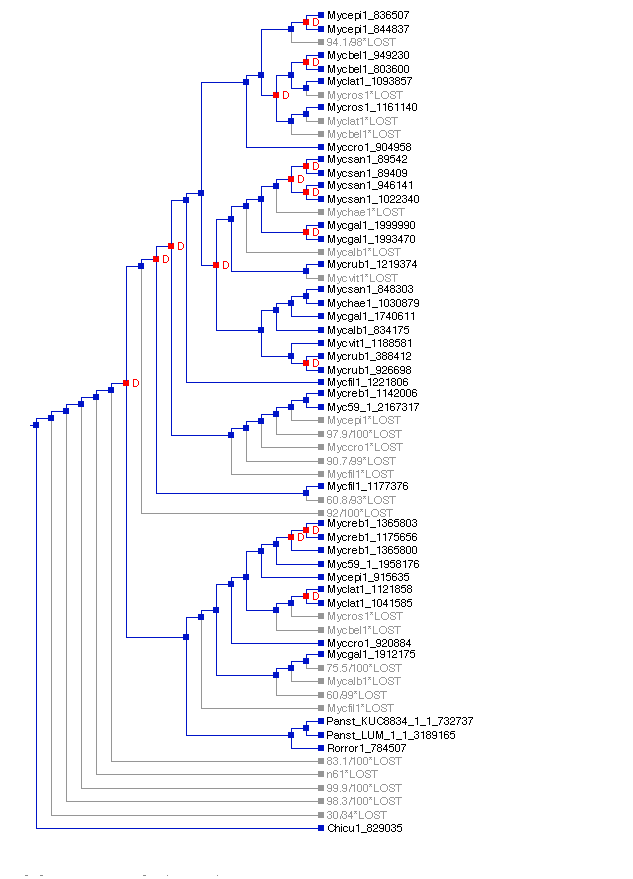

Supplement: MATERIAL S6 — Reconciliation analyses performed with DTL and DL models using NOTUNG. [file Data_Sheet_6.zip › NOTUNG_clade3_DTLmodel.png]

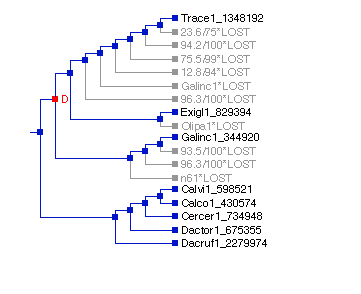

Supplement: MATERIAL S6 — Reconciliation analyses performed with DTL and DL models using NOTUNG. [file Data_Sheet_6.zip › NOTUNG_clade2_DLmodel.png]

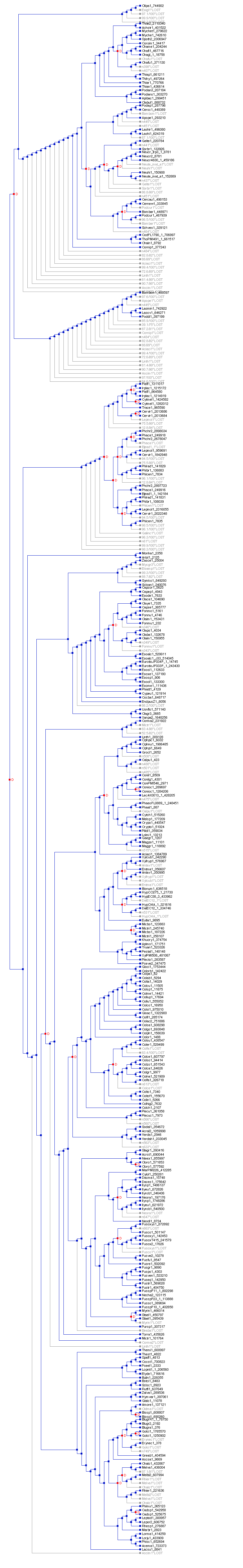

Supplement: MATERIAL S6 — Reconciliation analyses performed with DTL and DL models using NOTUNG. [file Data_Sheet_6.zip › NOTUNG_clade1_DLmodel.png]

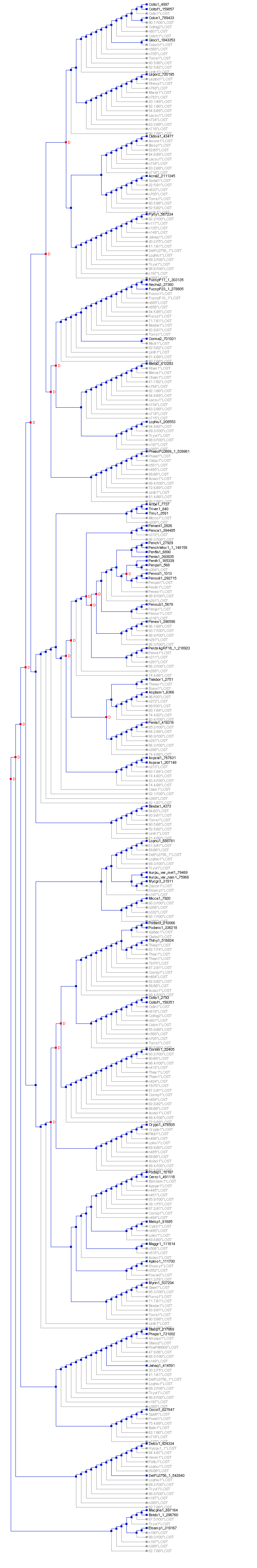

Supplement: MATERIAL S6 — Reconciliation analyses performed with DTL and DL models using NOTUNG. [file Data_Sheet_6.zip › NOTUNG_clade8_DLmodel.png]

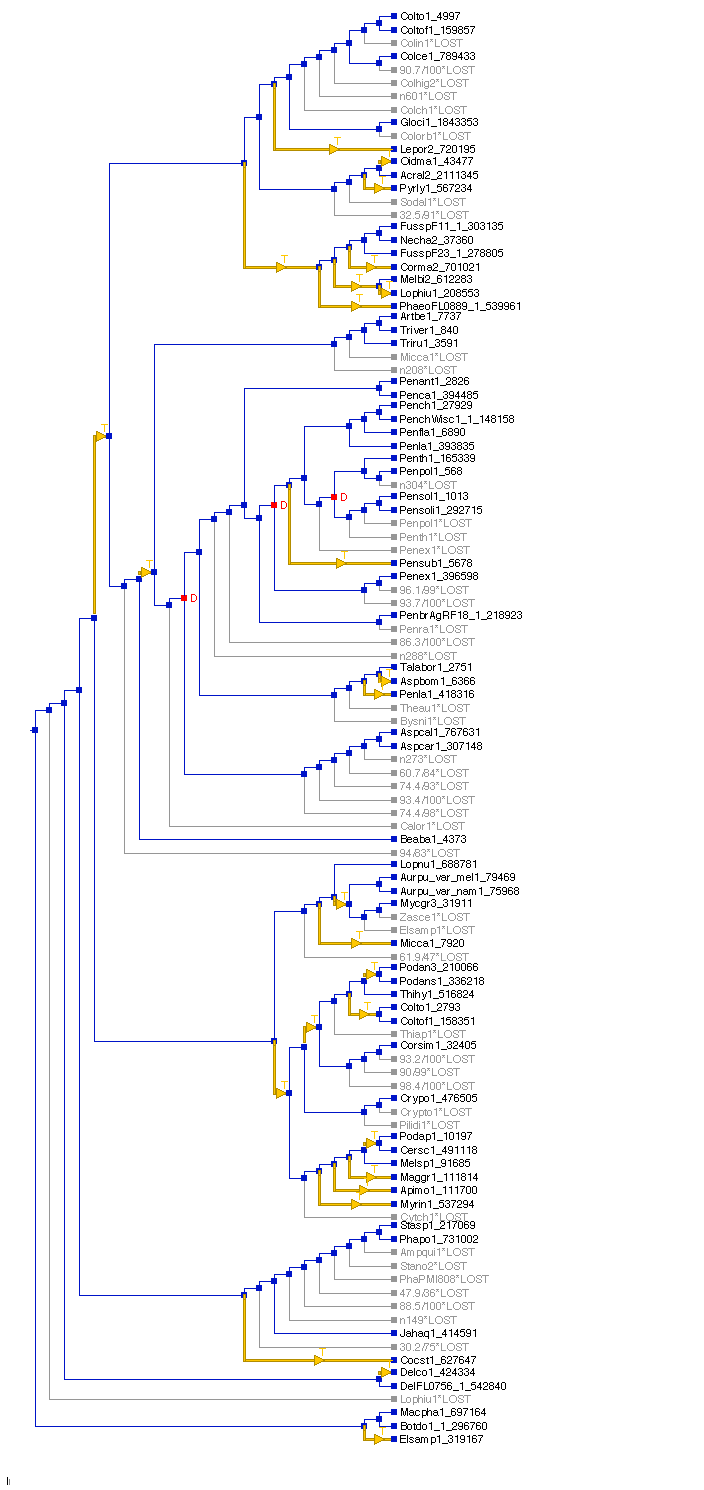

Supplement: MATERIAL S6 — Reconciliation analyses performed with DTL and DL models using NOTUNG. [file Data_Sheet_6.zip › NOTUNG_clade8_DTLmodel.png]
