# Supplementary material for: Evolutionary Histories of Type III Polyketide Synthases in Fungi
Source: Front Microbiol. 2020 Jan 21;10:3018. doi: 10.3389/fmicb.2019.03018 (PMC6985275; doi:10.3389/fmicb.2019.03018)
Supplement: MATERIAL S7 — Paralogy in the fungal type III polyketide synthase phylogenetic tree. [file Data_Sheet_7.pdf]

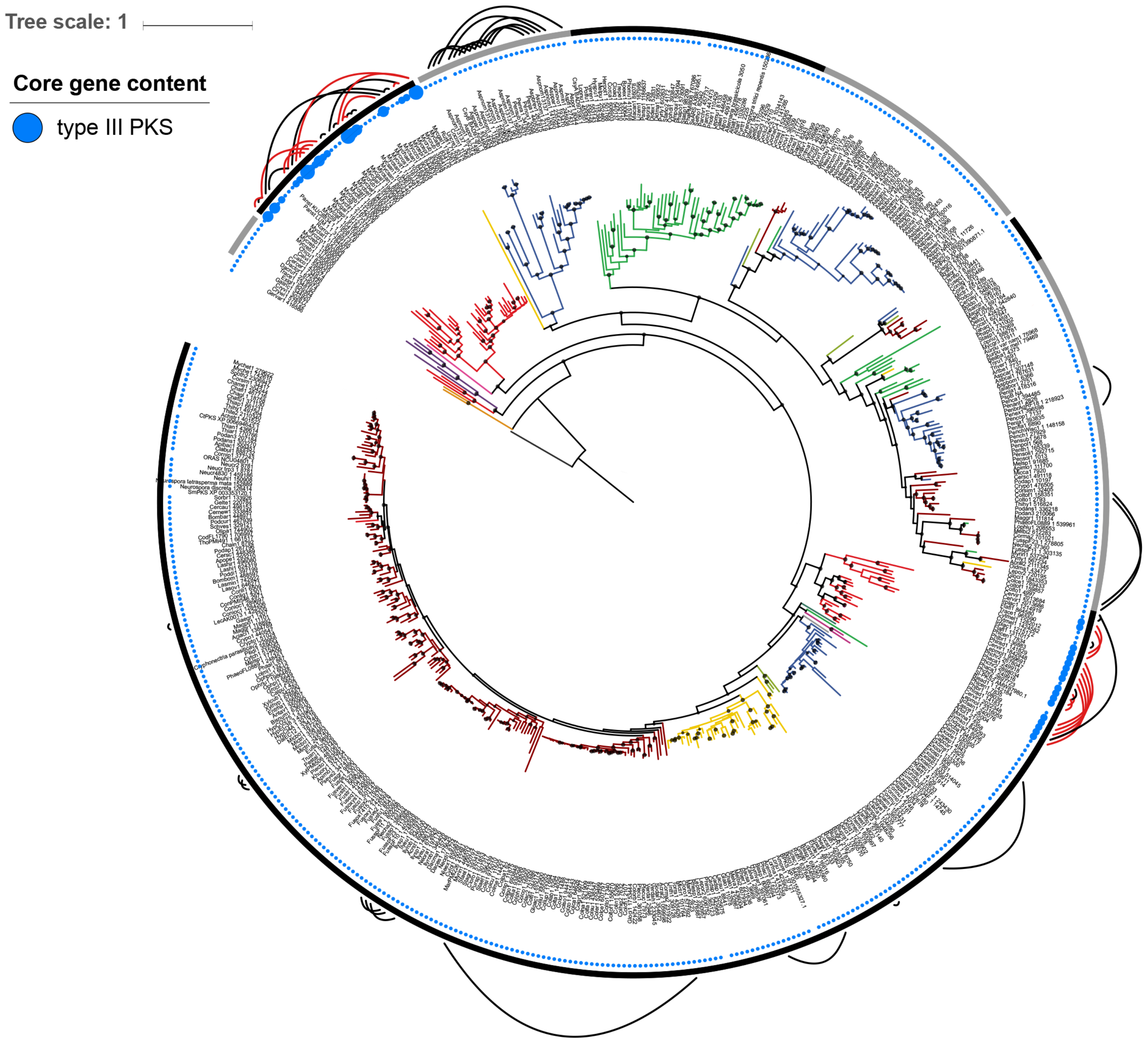

Supplementary material 7. Paralogy in the fungal type III polyketide synthase (PKS) phylogenetic tree.
The number of type III PKS genes at the locus is indicated as in Figure 3. The type III PKS phylogenetic clades are indicated in the middle ring as shown in Figure 3. The black and red lines link type III PKS paralogues in the same species, the red lines indicating those paralogues that are located at the same locus.
