# Supplementary material for: Evolutionary Histories of Type III Polyketide Synthases in Fungi
Source: Front Microbiol. 2020 Jan 21;10:3018. doi: 10.3389/fmicb.2019.03018 (PMC6985275; doi:10.3389/fmicb.2019.03018)
Supplement: MATERIAL S9 — Sequence conservation within fungal, plant and bacterial type III polyketide synthases (PKS) phylogenetic clades. [file Data_Sheet_9.pdf]

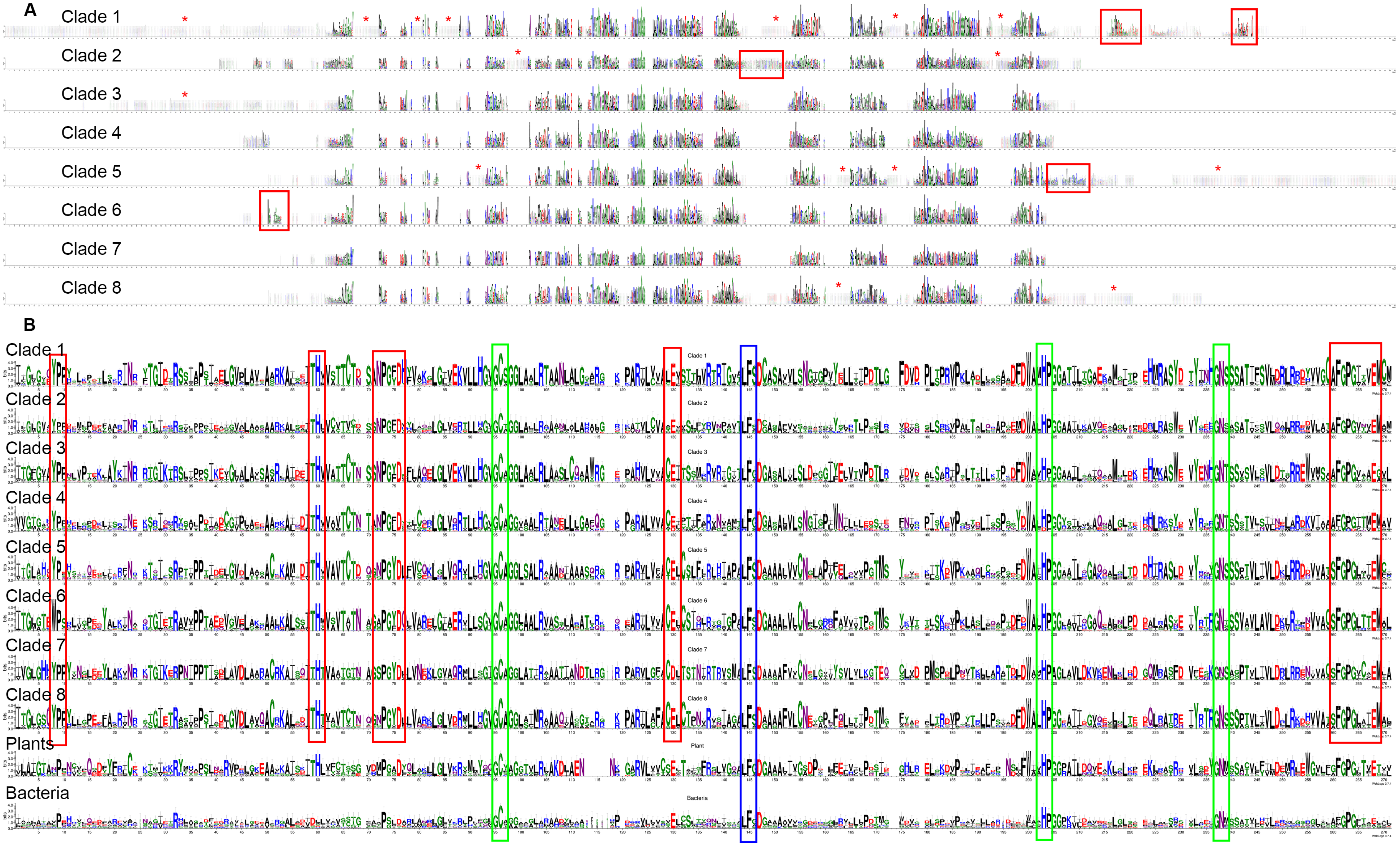

Supplementary material 9. Sequence conservation within fungal, plant and bacterial type III polyketide synthases (PKS) phylogenetic clades.
(A) Sequence logos of fungal type III PKSs from the same untrimmed multiple sequence alignment. The red squares indicate clade-specific conserved motifs and red stars indicate clade-specific sequence insertions.
(B) Sequence logos of fungal, plant and bacterial type III PKSs from the same trimmed multiple sequence alignment. The green squares indicate the conserved Cys-His-Asn catalytic triad found in all type III PKSs. The blue square indicates the conserved Phe residue that is involved in product specificity control (Rubin-Pitel *et al.*, 2008). Red squares indicate conserved amino acids in all fungal clades, and to some extent in plant and bacterial sequences.
